# *CircAASS* alleviates renal injury and fibrosis by regulating mitochondrial homeostasis in tubular epithelial cells

**DOI:** 10.1080/15548627.2025.2581212

**Published:** 2025-11-20

**Authors:** Tongtong Ma, Yanmei Yu, Huasheng Luo, Ziqi Zhang, Miaotao Wei, Chunjie Tian, Xianmou Fan, Zhenyi Yan, Shaowu Zhang, Junfeng Hao, Peng Wang

**Affiliations:** a Department of Anesthesiology, Affiliated Hospital of Guangdong Medical University, Zhanjiang, Guangdong, China; b Guangdong Provincial Key Laboratory of Autophagy and Major Chronic Non-Communicable Diseases, Key Laboratory of Prevention and Management of Chronic Kidney Diseases, Institute of Nephrology, Affiliated Hospital of Guangdong Medical University, Zhanjiang, Guangdong, China; c Department of Otorhinolaryngology, Affiliated Hospital of Guangdong Medical University, Zhanjiang, Guangdong, China; d Department of Plastic Surgery, Affiliated Hospital of Guangdong Medical University, Zhanjiang, Guangdong, China

**Keywords:** Acute kidney injury, Autophagy, Chronic kidney disease, Circular RNA, Mitochondrial dysfunction, Mitophagy

## Abstract

Acute kidney injury (AKI) is characterized by the dysfunction of renal tubular epithelial cells (TECs), often leading to renal fibrosis. Mitochondrial impairment is a common hallmark across various types of AKI. However, the potential role of circular RNAs (circRNAs) in modulating mitochondrial homeostasis during AKI and subsequent renal fibrosis remains underexplored. Our findings reveal a significant reduction of *circAass* levels in the renal cortex across all three AKI models. Mechanistically, *circAASS* mitigates TEC apoptosis and inflammatory responses by promoting mitochondrial homeostasis, thereby attenuating AKI. Specifically, cytoplasmic *circAASS* acts as a competing endogenous RNA (ceRNA) by sequestering *MIR324-3p*, which in turn enhances the expression of *PINK1*, a critical regulator of mitophagy. Additionally, nuclear *circAASS* directly interacts with the PPARGC1A/PGC-1α protein, inhibiting its ubiquitin-mediated degradation and thereby promoting mitochondrial biogenesis. Furthermore, we demonstrated that the RNA-binding protein IGF2BP2 suppresses *circAASS* biogenesis by binding to intronic sequences in the *AASS* pre-mRNA. Restoring *circAass* in AKI mouse models improves both mitochondrial biogenesis and mitophagy, ameliorating pro-inflammatory responses of TECs and thus mitigating renal fibrosis. Decreased *circAASS* expression and its association with impaired mitochondrial function in TECs, followed by more severe renal fibrosis, are observed in AKI patients. Collectively, our results suggest that *circAASS* protects against AKI by regulating mitochondrial homeostasis, highlighting its potential as a therapeutic target for kidney injury.

**Abbreviations:** AAV9: adeno-associated virus serotype 9; AKI: acute kidney injury; BLAST: Basic Local Alignment Search Tool; ceRNA: competing endogenous RNA; circRNA: circular RNA; CKD: chronic kidney disease; CP-AKI: cisplatin-induced AKI; DHE: dihydroethidium; FISH: fluorescence in situ hybridization; HK2: human renal proximal tubular cells; IF: immunofluorescence; H/R: hypoxia-reoxygenation; I/R: ischemia-reperfusion; ISH: in situ hybridization; LPS: lipopolysaccharide; m^6^A: N^6^-methyladenosine; MMP: mitochondrial membrane potential; NC: negative control; ncRNA: non-coding RNA; PAS: periodic acid-schiff staining; PINK1: PTEN induced kinase 1; PRKN: parkin RBR E3 ubiquitin protein ligase; RBPs: RNA-binding proteins; RIP: RNA immunoprecipitation; ROS: reactive oxygen species; RT-qPCR: real-time quantitative polymerase chain reaction; SAKI: septic AKI; Scr: serum creatinine; Seq: sequencing; siRNA: small interfering RNA; TECs: tubular epithelial cells; TEM: transmission electron microscopy.

## Introduction

Renal fibrosis, particularly tubulointerstitial fibrosis, is a hallmark and prevalent pathological feature of various progressive chronic kidney diseases (CKD), significantly contributing to increased patient morbidity and mortality [1,2]. Acute kidney injury (AKI) is a clinical syndrome characterized by acute renal dysfunction, frequently arising from renal ischemia-reperfusion (I/R), sepsis, or nephrotoxic exposures [3]. The exquisite metabolic demands of tubular epithelial cells (TECs) render them exceptionally vulnerable to these insults, triggering a pathological cascade involving mitochondrial dysfunction, inflammatory activation, and maladaptive repair [4,5]. Critically, unresolved tubular injury transforms transient AKI into self-perpetuating inflammation, which in turn drives progressive fibrosis through a process directly correlated with CKD mortality risk [6–8]. This vicious cycle underscores the urgent need for therapies targeting AKI and renal fibrosis.

Circular RNAs (circRNAs) are a unique type of non-coding RNA distinguished by their covalently closed loop structures, which confer resistance to exonucleases, rendering them more stable within cellular environments compared to linear non-coding RNAs. Recently, circRNAs have garnered attention as critical regulators in diverse biological processes and diseases [9,10]. They primarily exert their functions by acting as microRNA sponges, interacting with RNA-binding proteins (RBPs), modulating the expression of parental genes, and serving as templates for protein translation [11,12]. Emerging studies suggest that circRNAs play vital roles in kidney diseases and may contribute to the pathogenesis of renal fibrosis [11]; yet, the specific functions and mechanisms of many circRNAs in this context, particularly following AKI, remain largely uncharted.

The kidneys are among the most mitochondria-dense organs in the body, surpassed only by the heart. Given their high energy demands, maintaining mitochondrial homeostasis is imperative for normal kidney physiology and function. Notably, mitophagy and mitochondrial biogenesis cooperate to achieve this balance. On the one hand, mitochondrial autophagy, primarily regulated by the PINK1-PRKN pathway, is essential for cellular health, as it removes damaged mitochondria and mitigates oxidative stress. On the other hand, mitochondrial biogenesis, primarily driven by PPARGC1A/PGC-1α, ensures homeostasis by balancing mitochondrial production and turnover. Available evidence suggests that selectively enhancing mitophagy is effective in alleviating kidney injury across various AKI models [13–15], and in mitigating renal fibrosis [16–18]. Additionally, promoting mitochondrial biogenesis helps to lessen AKI and its progression [19,20]. While compelling evidence has established the importance of circRNAs in regulating mitochondrial function in pathologies such as cancer [21], liver fibrosis [22], and heart failure [23], the specific functions and mechanisms of circRNAs in mitochondrial dysfunction during AKI and subsequent renal fibrosis remain poorly understood and await further investigation.

In the present study, we identified a specific circRNA, *circAASS*, that is consistently downregulated in the kidney cortex of mice subjected to three distinct types of AKI: I/R-AKI, septic AKI (SAKI), and cisplatin-induced AKI (CP-AKI). Mechanistic studies revealed that overexpression of *circAASS* not only promotes mitophagy *via* the *MIR324-3p-PINK1* axis, but also enhances mitochondrial biogenesis by preventing the ubiquitin-mediated degradation of PPARGC1A/PGC-1α. These dual actions enable *circAASS* to maintain mitochondrial homeostasis, effectively alleviating AKI and preventing subsequent renal fibrosis. Collectively, our findings position *circAASS* as a potential novel diagnostic biomarker and therapeutic target for AKI and its fibrotic sequelae.

## Results

### *circAASS* is decreased in injured TECs in response to I/R-AKI, CP-AKI and SAKI

**Figure 1.**
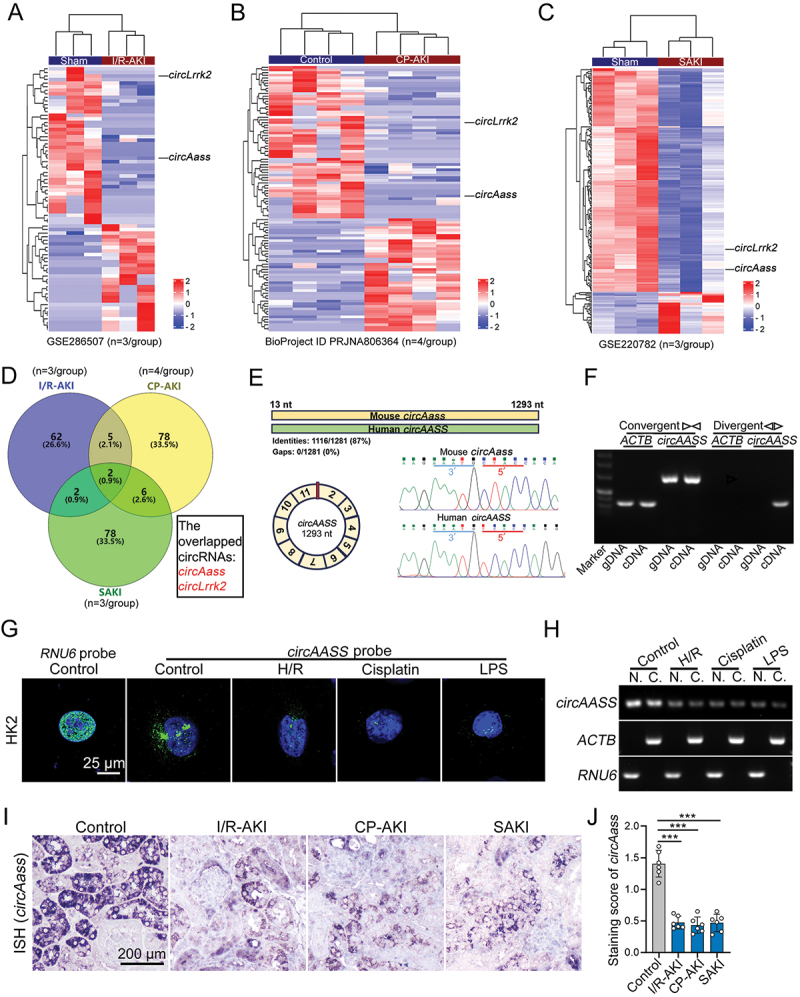
*CircAass* was downregulated in renal cortex of AKI mice. (A) Cluster heat map representing distinct circRNA expression values in I/R-AKI mice compared with Sham controls. (B) Clustered heat map showing the differentially expressed circRNAs in renal cortex between the CP-AKI mice and Control mice. (C) Cluster heat map showing the differences in the expression of circRNA in SAKI mice and Sham mice. (D) Venn diagram showing the two common differentially expressed circRNAs identified by RNA-seq across three distinct AKI models (I/R-AKI, cisplatin-induced AKI, and sepsis-AKI) compared with sham controls (*n* = 3–4 per group). (E) The schematic illustration showed the circularization of *AASS* exons 2–11 to form *circAASS*. The back-splicing junction of *circAASS* was verified by RT-PCR and Sanger sequencing. (F) *CircAASS* expression in TECs was detected by RT-PCR. Agarose gel electrophoresis showed that divergent primers amplified *circAASS* in cDNA but not genomic DNA (gDNA). (G) FISH showing the expression and localization of *circAASS* in HK2. (H) Subcellular fractionation assay and detected by agarose gel electrophoresis. (I and J) representative images of ISH (I) and quantitative analysis (J) of *circAass* expression in kidney from three different mouse models of AKI. Data are presented as the mean ± sd. ***p < 0.001, by 2-tailed Student’s *t* test (J). Scale bars: 25 μm (G) and 200 μm (I).

To identify the dysregulated circRNAs potentially involved in TEC injury in AKI, we analyzed three datasets containing differentially expressed circRNAs in tissues from mouse AKI models induced by I/R (GSE286507), cisplatin treatment (BioProject ID PRJNA806364) [24] and sepsis (GSE220782) [25] (Figure 1(A-C)). By taking the intersection of these datasets, we found two circRNAs (*circAass* and *circLrrk2*) that exhibited decreased expression in the kidney tissue from AKI mice, as compared with that from control mice (Figure 1(D), Table S1). To assess the translational potential, we analyzed the conservation between human and mouse of alternative circRNAs. The sequences of *circAASS* and *circAass* are 87% conserved between human and mouse (Figure 1(E), Figure S1A), while those of *circLRRK2* and *circLrrk2* are 78% (Figure S1A); therefore, we selected *circAASS* for further investigation. *CircAASS* was composed of exons 2 to 11 of the linear transcript of the *AASS* gene with a length of 1,293 nucleotides. Sequencing results confirmed the existence of back-splicing site in divergent primers-amplified PCR product (Figure 1(E)). Accordingly, *circAASS* was validated by PCR amplification using divergent primers from cDNA but not gDNA of HK2 cells (Figure 1(F)). RNase R resistance analysis showed that *circAASS*, but not linear *AASS* mRNA (*lAASS*), was resistant to RNase R digestion (Figure S1B). Also, like other circRNAs, *circAASS* was more stable than its linear counterpart *AASS* mRNA (Figure S1C). An RNA-FISH assay indicated that *circAASS* was located in both the nucleus and cytoplasm of TECs (Figure 1(G)). Similar results were obtained in the subcellular fractionation assay (Figure 1(H)).

We further examined the expression of *circAASS* in TECs and kidney tissues by qPCR. The results showed that *circAASS* expression levels were decreased in injured TECs (Figure S1D) and kidney tissues (Figure S1E). Furthermore, compared to control mice, expression of *circAass* was remarkably downregulated in kidney tissues from I/R-AKI, CP-AKI, and SAKI mice (Figure 1(I,J)). Together, these data suggest that *circAASS* is downregulated in injured TECs in vivo and in vitro.

### *circAASS* attenuates mitochondrial damage and pro-fibrotic responses in injured TECs

Based on the common dysregulated mRNAs in kidney tissues from I/R-AKI (GSE286507), cisplatin-induced AKI (BioProject ID PRJNA806364) and SAKI mice (GSE220812), we performed KEGG and GO analysis. The results showed that the enriched items were associated with metabolic pathways (Figure S2A, Table S2). Indeed, under a transmission electron microscope, the mitochondrion morphology was commonly disorganized in proximal TECs from AKI mice induced by I/R, cisplatin treatment and sepsis (Figure S2B). These results are consistent with previous studies by us and other investigators [26–28]. Given that *circAass* was generally downregulated in TECs from a variety of AKI mouse models, we hypothesized that decreased expression of *circAASS* in TECs was related to the mitochondrial dysfunction. To investigate the role of *circAASS* on mitochondrial dysfunction in injured TECs, *circAASS* was knocked down or overexpressed in TECs in the presence or absence of hypoxia-reoxygenation (H/R) treatment. The knockdown or overexpression efficacy of *circAASS* was evaluated, demonstrating that *circAASS* was specifically knocked down or overexpressed, while the expression of *lAASS* remained unchanged (Figure S2C, D).

**Figure 2.**
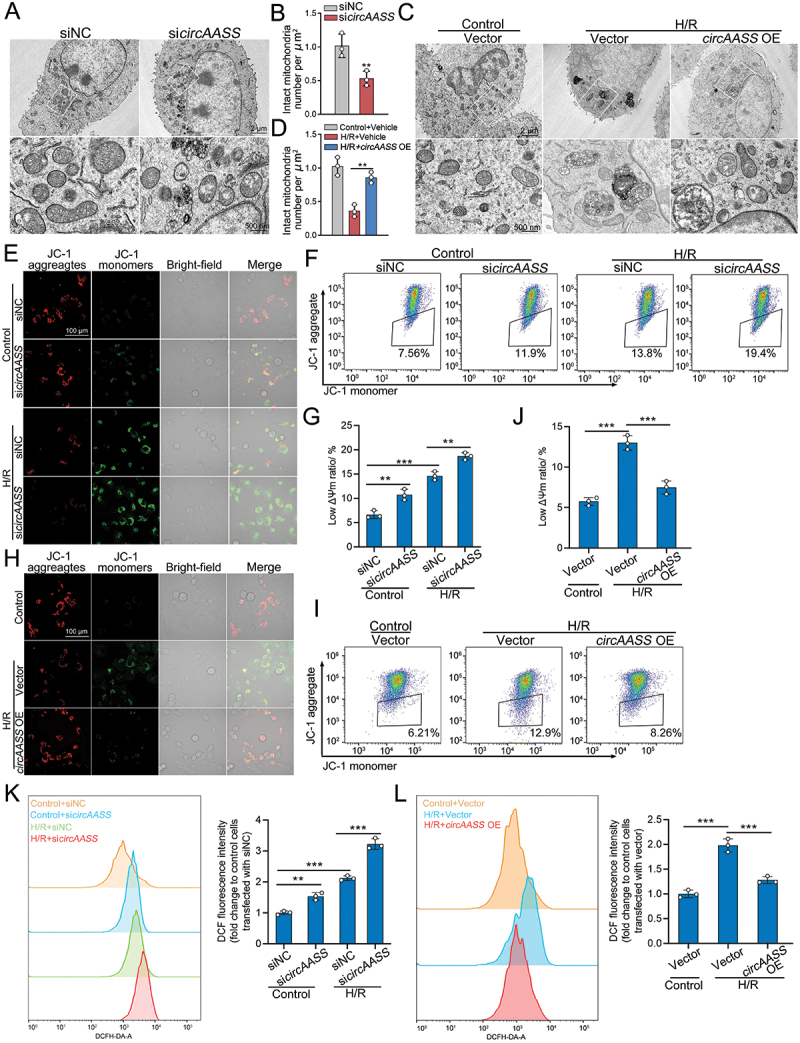
*circAASS* attenuates mitochondrial damage in injured TECs. (A and B) Knockdown of *circAASS* aggravates mitochondrial damage in HK2 cells, as shown by representative TEM images (A) and quantitative analysis of intact mitochondria per μm^2^ (B). (C and D) Overexpression of *circAASS* ameliorates mitochondria in HK2 cells treated with H/R, as shown by representative TEM images (C) and quantitative analysis of intact mitochondria per μm^2^ (D). (E) JC-1 staining reveals that *circAASS* knockdown reduces mitochondrial membrane potential in HK2 cells, both under normal and H/R-treated conditions. (F and G) Flow cytometry analysis shows that knockdown of *circAASS* led to reduced mitochondrial membrane potential in HK2 cells, both under normal conditions and following H/R treatment. (H) JC-1 staining reveals that *circAASS* overexpression elevated mitochondrial membrane potential in HK2 cells, under H/R-treated conditions. (I and J) Flow cytometry analysis shows that overexpressing *circAASS* leads to increased mitochondrial membrane potential in H/R-treated HK2 cells. (K) Flow cytometry analysis shows that knockdown of *circAASS* increased ROS levels in HK2 cells, both under normal conditions and following H/R treatment. (L) Overexpression of *circAASS* reduced the levels of ROS in HK2 cells, under H/R-treated conditions. Data are presented as the mean ± sd. **p < 0.01, by 2-tailed Student’s *t* test (B). **p < 0.01, ***p < 0.001, by 1-way ANOVA with Tukey’s multiple-comparison test (D, G, J, K and L). Scale bars: 2 μm (A and C, upper panel), 500 nm (A and C, lower panel) and 100 μm (E and H).

Loss of function in *circAASS* aggravated mitochondria damage, whereas overexpression of *circAASS* mitigated mitochondria damage (Figure 2(A-D)). Furthermore, we measured JC-1 levels and reactive oxygen species (ROS) levels to further examine mitochondrial function and oxidative stress markers. JC-1 staining showed that mitochondrial membrane potential (MMP) was depolarized in *circAASS*-knockdown HK2 cells (Figure 2(E-G)). Of note, *circAASS* overexpression had the opposite effects (Figure 2(H-J)). Knockdown of *circAASS* significantly elevated ROS levels in HK2 cells (Figure 2(K)), whereas *circAASS* overexpression markedly reduced ROS production (Figure 2(L)).

**Figure 3.**
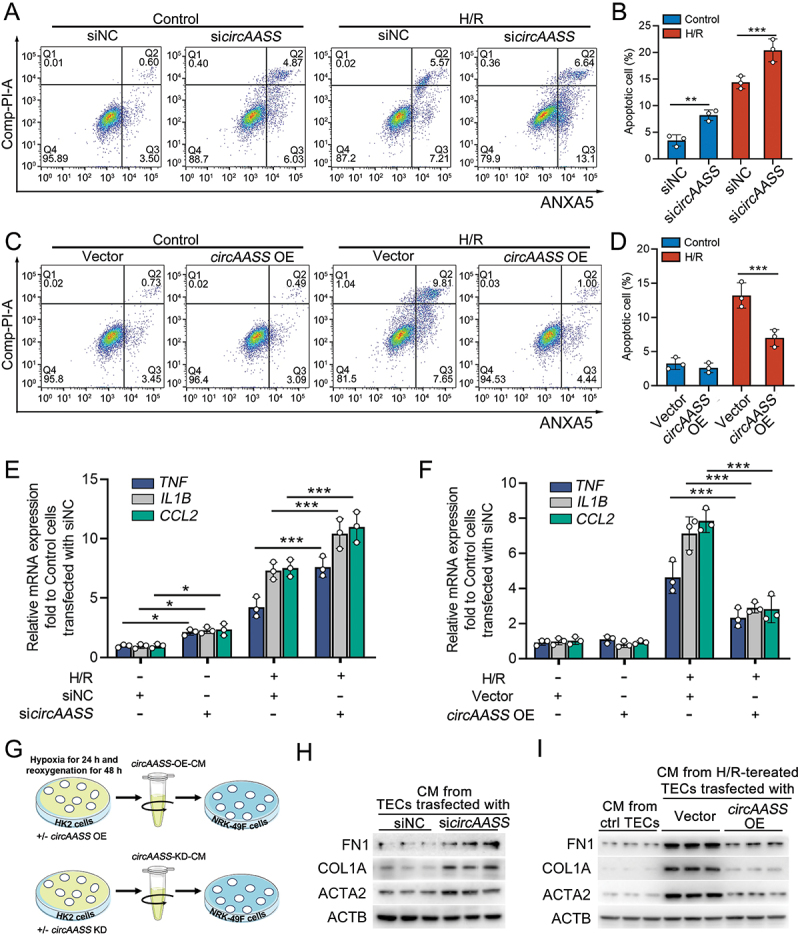
*circAASS* inhibits apoptosis and pro-fibrotic responses in injured TECs. (A and B) The effects of *circAASS* knockdown on apoptosis and the quantification data. Cell apoptosis of HK2 was assayed by co-staining of ANXA5/annexin V and propidium iodide followed by flow cytometric analysis. (C and D) The effects of *circAASS* overexpression on apoptosis and the quantification data. (E) qPCR showing the expression of pro-inflammatory cytokines in HK2 cells transfected with *circAASS* siRNA. (F) qPCR showing the expression of pro-inflammatory cytokines in HK2 cells overexpressed with *circAASS*. (G) Diagram shows the experimental design. (H) Western blotting showing protein levels of FN1, COL1A, and ACTA2/αSMA in NRK49F cells incubated with conditional medium from HK2 cells with or without *circAASS*-KD treatment. (I) Western blotting showing protein levels of FN1, COL1A, and ACTA2/αSMA in NRK49F cells incubated with the supernatant from H/R-treated HK2 cells overexpressed with *circAASS* or empty vector. Data are presented as the mean ± sd. *p < 0.05, **p < 0.01, ***p < 0.001, by 1-way ANOVA with Tukey’s multiple-comparison test (B, D, E and F).

Previous studies showed that mitochondria damage in TECs, triggering apoptosis and pro-inflammatory effects of TECs, is a critical event in the occurrence and development of kidney fibrosis [29–31]. Therefore, we explored the function of *circAASS* on apoptosis and the pro-inflammatory responses in injured TECs. Notably, *circAASS* knockdown increased HK2 cell apoptosis, whereas its overexpression reduced apoptotic rates (Figure 3(A-D)). Consistent with these findings, *circAASS* modulation similarly influenced pro-inflammatory responses, as evidenced by altered expression of *IL1B*, *CCL2*, and *TNF* (Figure 3(E,F)). To observe the role of *circAASS* on the pro-fibrotic effects of HK2 cells, we collected medium from HK2 cells with or without *circAASS*-KD treatment (*circAASS*-KD) and co-cultured with fibroblasts (Figure 3(G)), further analysis showed that the levels of profibrotic factors, including FN1, ACTA2/αSMA and COL1A/collagen I, were significantly increased in fibroblasts cultured with *circAASS*-KD treatment (Figure 3(H)). Inversely, the expression levels of profibrotic factors were reduced in fibroblasts cultured with the supernatant from H/R-treated HK2 cells overexpressed with *circAASS* (Figure 3(I)). Taken together, these data suggested that *circAASS* attenuates mitochondrial damage and pro-fibrotic responses in injured TECs.

### Cytoplasmic *circAASS* modulates *MIR324-3p*/PINK1 axis-regulated mitophagy in TECs

**Figure 4.**
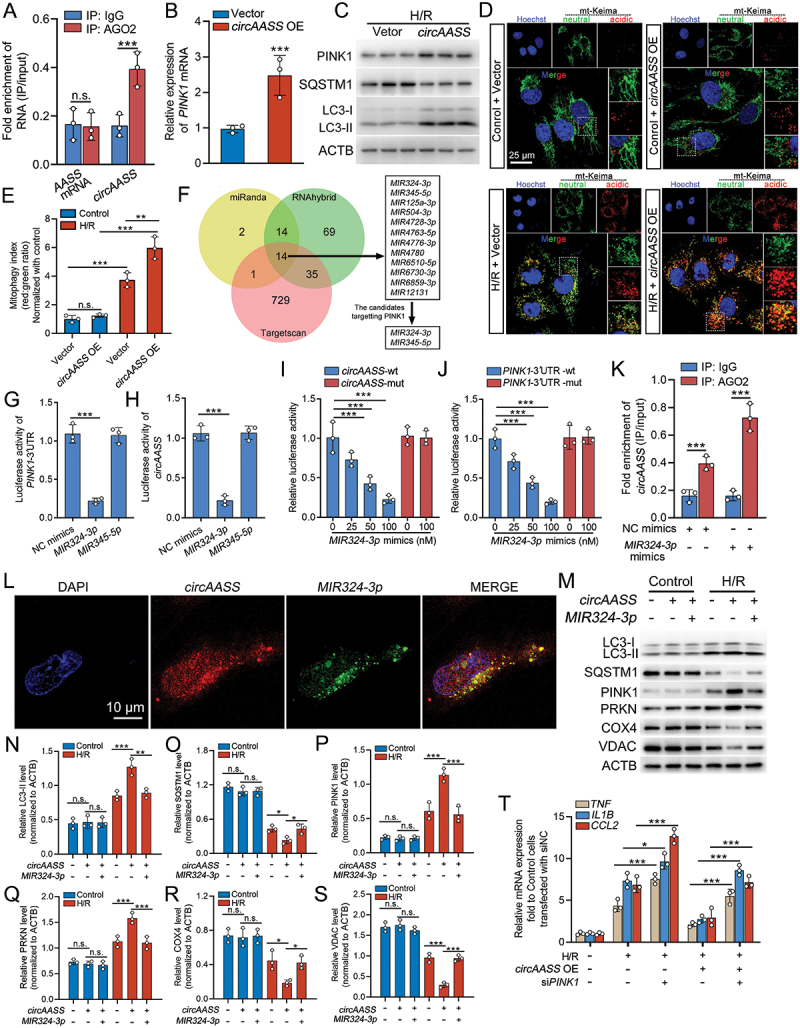
Cytoplasmic *circAASS* modulates *MIR324-3p*/PINK1 axis-regulated mitophagy in TECs. (A) RIP assay confirms the interaction between *circAASS* and AGO2. (B) qPCR analysis showing the expression of PINK1 in *circAASS*-overexpressing HK2 cells. (C) Western blotting showing the protein expression of PINK1, SQSTM1/p62 and LC3 in *circAASS*-overexpressing HK2 cells exposed to H/R treatment. (D and E) mKeima expression in HK2 cells transfected with *circAASS* or empty vector, with or without H/R treatment. Live-cell confocal imaging was performed (excitation 561 nm: red; excitation 488 nm: green). The 561/488 nm excitation ratio of mKeima was quantified; *n* = 3. (F) Integrative analysis of bioinformatics prediction to screen for *circAASS*-binding miRNAs, *MIR324-3p* and *MIR345-5p* are the candidate miRNAs that also target 3‘ UTR of *PINK1*. (G and H) Luciferase reporter assays showing *MIR324-3p* mimics, but not *MIR345-5p* mimics, remarkably inhibited luciferase activity of *PINK1*-3‘ UTR or *circAASS*, as compared to NC mimics. (I and J) Luciferase reporter assays showing mutant *circAASS* or mutant *PINK1*-3’ UTR reversed the effects of *MIR324-3p* mimics on luciferase activity of *PINK1*-3‘UTR or *circAASS*. (K) RIP assay showing that *circAASS* directly bound to *MIR324-3p* in HK2 cells. (L) RNA FISH showing the colocalization of *circAASS* and *MIR324-3p* in the cytoplasm of HK2 cells. (M-S) Western blot and quantification data showing the expression of mitophagy related factors in *circAASS*-overexpressed HK2 cells transfected with NC mimic or *MIR324-3p*. (T) qPCR analysis of pro-inflammatory gene expression (*TNF*, *IL1B*, *CCL2*) in HK2 cells. *PINK1* knockdown markedly exacerbated the inflammatory response induced by H/R and abolished the anti-inflammatory effect of *circAASS* overexpression. Data are presented as the mean ± sd. ***p < 0.001, by 2-tailed Student’s *t* test (A, B and K). ***p < 0.001, by 1-way ANOVA with Tukey’s multiple-comparison test (E, G, H, I, J, N, O, P, Q, R, S, and T). Scale bars: 25 μm (D) and 10 μm (l).

Competing endogenous RNA (ceRNA) is a well-characterized mechanism driven by circRNAs, which modulates the expression of specific targets by sponging miRNAs at post-transcriptional level [32,33]. We then examined whether *circAASS* could function as a miRNA sponge. RNA immunoprecipitation (RIP) assays showed that *circAASS* but not *lAASS* was abundantly enriched in AGO2-containing micro-ribonucleoprotein complexes, compared with control IgG (Figure 4(A)), suggesting the occupancy of AGO2 in the sequence of *circAASS*.

To identify the downstream signaling pathways and target genes that are regulated by *circAASS* in injured cells, we performed RNA sequencing (RNA-seq) on control and *circAASS*-overexpressing HK2 cells subjected to H/R treatment. KEGG analysis showed that mitophagy responded remarkably in the *circAASS*-overexpressing HK2 cells. The differentially expressed genes related with mitophagy, including *PINK1*, were increased significantly in H/R-treated HK2 cells with *circAASS* overexpression (Figure S3A). PINK1 is a key molecule for initiating mitophagy [34]. Previous studies showed that elevating the expression of *PINK1* could attenuate AKI and protect kidney function by protecting mitochondrial function [13,14,35]. *circAASS* overexpression promotes *PINK1* mRNA and protein levels (Figure 4(B,C)). To determine whether *circAASS* promotes mitophagy, we quantified mitophagic flux using the mKeima fluorescent reporter system, which is widely recognized as a specific and quantitative method for monitoring mitophagy based on pH-sensitive fluorescence emission [27]. Under normal conditions, overexpression of *circAASS* did not significantly alter mitophagic flux in TECs. However, under H/R stress, *circAASS* overexpression markedly enhanced mitophagic flux, as indicated by a significant increase in acidic mKeima fluorescence signals (Figure 4(D,E)). These results provide direct evidence that *circAASS* facilitates the full completion of mitophagy rather than leading to an accumulation of mitophagosomes resulting from defective degradation.

To screen the potential miRNAs targets of *circAASS*, three databases (miRanda, RNAhybrid and TargetScan) were used to perform in silico analysis, which jointly predicted that 14 miRNAs may be the potential targets of *circAASS*. Among these candidates, two miRNAs, including *MIR324-3p* and *MIR345-5p* were also predicted to bind with *PINK1* mRNA in TargetScan database (Figure 4(F)), which met the basic precondition of ceRNA theory. Furthermore, *MIR324-3p* was significantly upregulated in H/R- and cisplatin-treated TECs, while *MIR345-5p* expression was unchanged (Figure S3B, S3C). Transfection of *MIR324-3p* mimics together with *circAASS* or *PINK1*-3′ UTR luciferase reporter plasmids into HEK-293T cells showed that *MIR324-3p* mimics, but not *MIR345-5p* mimics, remarkably inhibited luciferase activity of *PINK1*-3′ UTR or *circAASS*, as compared to mimics NC (Figure S3D; Figure 4(G,H)), while these effects were hindered in cells transfected with mutant *circAASS* or mutant *PINK1*-3′ UTR (Figure 4(I,J)).

An affinity-isolation assay using biotinylated *MIR324-3p* showed the binding between *circAASS* and *MIR324-3p* (Figure 4(K)). Consistently, RNA FISH demonstrated the colocalization of *circAASS* with *MIR324-3p* in the cytoplasm of HK2 cells (Figure 4(L)). Therefore, these results indicated that *circAASS*, acting as a sponge, directly bound to *MIR324-3p* in HK2 cells. In the *circAASS*-overexpressed HK2 cells, the enhanced mitophagy was blocked by *MIR324-3p* mimic (Figure 4(M-S)).

To explore the causal relationship between mitophagy and inflammatory signaling, we performed a rescue experiment to determine if *circAASS* suppresses inflammation indirectly through PINK1-mediated mitophagy. Specifically, we knocked down *PINK1* using siRNA in H/R-treated TECs with or without *circAASS* overexpression (Figure S3E). qPCR analysis demonstrated that knockdown of *PINK1* markedly exacerbated H/R-induced inflammation, shown by elevated mRNA levels of *TNF*, *IL1B*, and *CCL2* (Figure 4(T)). Notably, while *PINK1* knockdown significantly attenuated the anti-inflammatory effect of *circAASS*, a substantial protective effect persisted, as inflammatory cytokine expression remained significantly lower in cells with *circAASS* overexpression compared to *PINK1*-knockdown controls lacking *circAASS*.

These findings demonstrate that the anti-inflammatory effect of *circAASS* is significantly mediated through the *MIR324-3p*/*PINK1*/mitophagy axis, but also point to the existence of a complementary, PINK1-independent pathway.

### Nuclear *circAASS* inhibits RNF34-mediated ubiquitination and degradation of PPARGC1A/PGC-1α through direct binding

**Figure 5.**
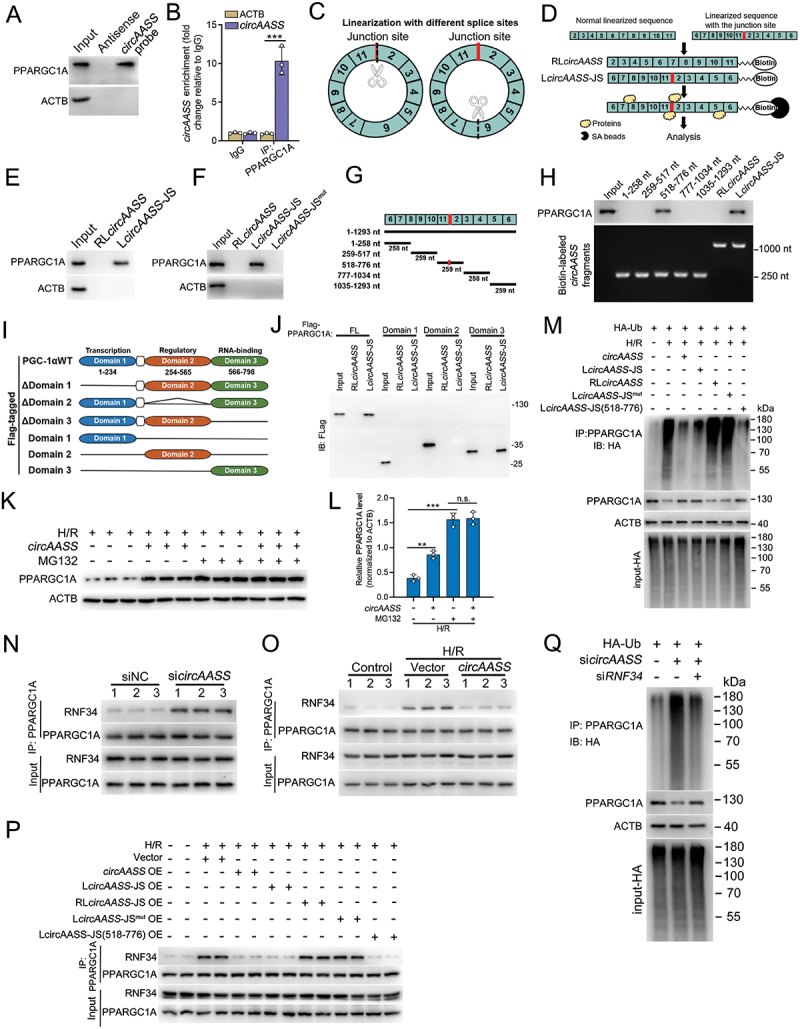
*circAASS* inhibits ubiquitination-proteasome pathways mediated PPARGC1A/PGC-1α degradation by directly binding with PPARGC1A/PGC-1α. (A) PPARGC1A/PGC-1α levels in affinity-isolation assays using biotinylated antisense probes (control) or probes targeting the junction site sequence of *circAASS*. (B) Analysis for *circAASS* enrichment by PPARGC1A/PGC-1α, as revealed by RNA immunoprecipitation assay. (C) Schematic of *circAASS* linearization. (D) The schematic of rna affinity-isolation assay with streptavidin magnetic beads (SA beads) to enriched the proteins interacted with biotinylated RL*circAASS* or L*circAASS*-JS. (E) RNA affinity-isolation assays showing the interaction of L*circAASS*-JS with PPARGC1A/PGC-1α. (F) RNA affinity-isolation assays showing the interaction of L*circAASS*-JS with PPARGC1A/PGC-1α, as compared to L*circAASS*-JS^mut^. (G) The schematic showing sequential truncated mutations of L*circAASS*-JS and the 518–776 nt of L*circAASS*-JS contained the junction sequence. (H) RNA affinity-isolation assay using sequentially truncated L*circAASS*-JS mutants reveals that both full-length L*circAASS*-JS and a truncated fragment (518–776 nt) bind to PPARGC1A/PGC-1α. (I) To test the binding site of PPARGC1A/PGC-1α and *circAASS*, truncated mutants of PPARGC1A/PGC-1α were established and tagged with Flag. (J) L*circAASS*-JS interacts with PPARGC1A/PGC-1α, as shown by RNA affinity-isolation assay detecting binding to both the full-length protein and domain 3. (K and L) Western blotting and quantification data reveal that the promoting role of *circAASS* on PPARGC1A/PGC-1α expression is abrogated by using the proteasome inhibitor (MG132). (M) HA-Ub-expressing HK2 cells were transfected with *circAASS*, L*circAASS*-JS, RL*circAASS*, L*circAASS*-JS^mut^ or L*circAASS*-JS (518–776) to test their role on the ubiquitination of PPARGC1A/PGC-1α. (N) Co-IP assay demonstrates increased interaction between RNF34 and PPARGC1A/PGC-1α upon *circAASS* knockdown in HK2 cells. (O) Co-IP assay shows decreased interaction between RNF34 and PPARGC1A/PGC-1α upon *circAASS* overexpression in H/R-treated HK2 cells. (P) Co-IP assay reveals RNF34-PPARGC1A/PGC-1α interaction in H/R-treated HK2 cells overexpressing *circAASS* variants (L*circAASS*-JS, RL*circAASS*, L*circAASS*-JS^mut^, or L*circAASS*-JS 518–776). (Q) Western blot analysis demonstrates that RNF34 knockdown abolishes *circAASS* knockdown-induced PPARGC1A/PGC-1α ubiquitination. Data are presented as the mean ± sd. **p < 0.01, ***p < 0.001, by 1-way ANOVA with Tukey’s multiple-comparison test (L).

To better explore the function of *circAASS*, we performed RNA affinity-isolation assays of *circAASS* using biotinylated probes targeting the *circAASS* back-spliced sequence (Figure S4A), and the precipitates were subjected to mass spectrometry analysis. Among the RNA-binding proteins identified by MS analysis, PPARGC1A/PGC-1α was the most abundant protein among the candidates (Figure S4B, C, Table S3). Accordingly, we verified the binding between *circAASS* and PPARGC1A/PGC-1α using RNA affinity isolation, followed by immunoblotting and RIP assays (Figure 5(A,B)). Moreover, FISH assay of *circAASS*, followed by immunofluorescence for PPARGC1A/PGC-1α, showed that *circAASS* was colocalized with PPARGC1A/PGC-1α in the nucleus of HK2 cells (Figure S4D). These data showed that *circAASS* directly binds to PPARGC1A/PGC-1α.

We and others have demonstrated that non-coding RNAs interact with specific proteins with determined sequences, some truncated mutations might work as well as the full-length sequence [22,36]. Therefore, we next tried to identify the specific sequences within *circAASS* which were responsible for the interaction between *circAASS* and PPARGC1A/PGC-1α. To this end, in vitro transcriptions of linearized *circAASS* were performed, *circAASS* was linearized at two different sites to produce two kinds of linearized products (Figure 5(C)). Of note, linearization through breaking down exon 6 transformed *circAASS* to a linearization sequence containing the identical junction sequence (Figure 5(D)). RNA affinity-isolation assays were performed with biotin-labeled linear transcripts, showing that linearized-*circAASS* with junction site (L*circAASS*-JS) was able to interact with PPARGC1A/PGC-1α while regular linearized-*circAASS* (RL*circAASS*) could not (Figure 5(E)). Replacing the junction site sequence with adenylate in L*circAASS*-JS abrogated the interaction between L*circAASS*-JS and PPARGC1A/PGC-1α (Figure 5(F)), which further verified the essential role of junction site sequence in mediating this interaction. A series of truncated mutations were constructed and RNA affinity-isolation assays demonstrated that the 518–776 nt of L*circAASS*-JS interacted with PPARGC1A/PGC-1α like the full-length construct did (Figure 5(G,H)).

To determine the definite binding sites within PPARGC1A/PGC-1α with *circAASS*, we performed full-length or truncated PPARGC1A/PGC-1α overexpression in TECs (Figure 5(I)). RNA affinity-isolation and RIP assays demonstrated that *circAASS* bound to the RNA-binding domain of PPARGC1A/PGC-1α (Figure 5(J), S4E). CircRNAs regulate the post-translational modification by interacting with proteins [37]. Here, *circAASS* overexpression ameliorated the H/R-induced downregulation of PPARGC1A/PGC-1α expression (Figure S4F), while the mRNA level of *PPARGC1A* was unaffected by overexpressing *circAASS* (Figure S4G), indicating that *circAASS* regulates PPARGC1A/PGC-1α at the post-translational level. Moreover, when a specific proteasome inhibitor (MG132) was used, the enhancing effect of *circAASS* overexpression on PPARGC1A/PGC-1α protein levels was abrogated (Figure 5(K,L)). Further experiments showed that *circAASS* overexpression inhibited the ubiquitination of PPARGC1A/PGC-1α, whereas the overexpression of a *circAASS*-mutant failed to regulate PPARGC1A/PGC-1α protein ubiquitination (Figure 5M). These data demonstrated that *circAASS* stabilizes PPARGC1A/PGC-1α by inhibiting its ubiquitin/proteasome-dependent degradation.

As described previously, PPARGC1A/PGC-1α is degraded by the ubiquitin-proteasome system, and binding of RNF34 to the C-terminal of PPARGC1A/PGC-1α leads to proteasome-dependent degradation [38–40]. Therefore, we hypothesized that *circAASS* protects PPARGC1A/PGC-1α against degradation by binding to PPARGC1A/PGC-1α and masking the binding site of RNF34 on PPARGC1A/PGC-1α, thus protecting PPARGC1A/PGC-1α against ubiquitination and degradation. Indeed, knockdown of *circAASS* promoted the interaction between PPARGC1A/PGC-1α and RNF34 (Figure 5(N)), whereas overexpression of *circAASS* reduced the interaction between PPARGC1A/PGC-1α and RNF34 (Figure 5(O)). Consistently, overexpression of either L*circAASS*-JS or the fragment (518–776 nt) of L*circAASS*-JS could repress the binding of RNF34 to PPARGC1A/PGC-1α (Figure 5(P)). Notably, knocking down RNF34 markedly reverses the stimulatory effect of *circAASS* knockdown on PPARGC1A/PGC-1α ubiquitination (Figure 5(Q)), confirming that decreased *circAASS* expression promotes PPARGC1A/PGC-1α ubiquitination *via* RNF34.

### IGF2BP2 functions as a negative regulator of *circAASS* circularization in TECs

Transcriptomic profiling of I/R-AKI, CP-AKI, and SAKI mouse models (Table S2) revealed no significant changes in linear *AASS* mRNA expression, a result corroborated by RT-qPCR analysis of both mature *AASS* mRNA and its precursor (Figure S5A, B). However, *circAASS* was consistently downregulated in injured kidneys, raising the question of why this occurred despite unchanged *AASS* precursor and mRNA levels. Given that circRNAs and mRNAs share precursor origins [41,42] and that circRNA production can be modulated by RBPs [12,42], we hypothesized that *circAASS* is post-transcriptionally regulated by specific RBPs in AKI pathogenesis.

**Figure 6.**
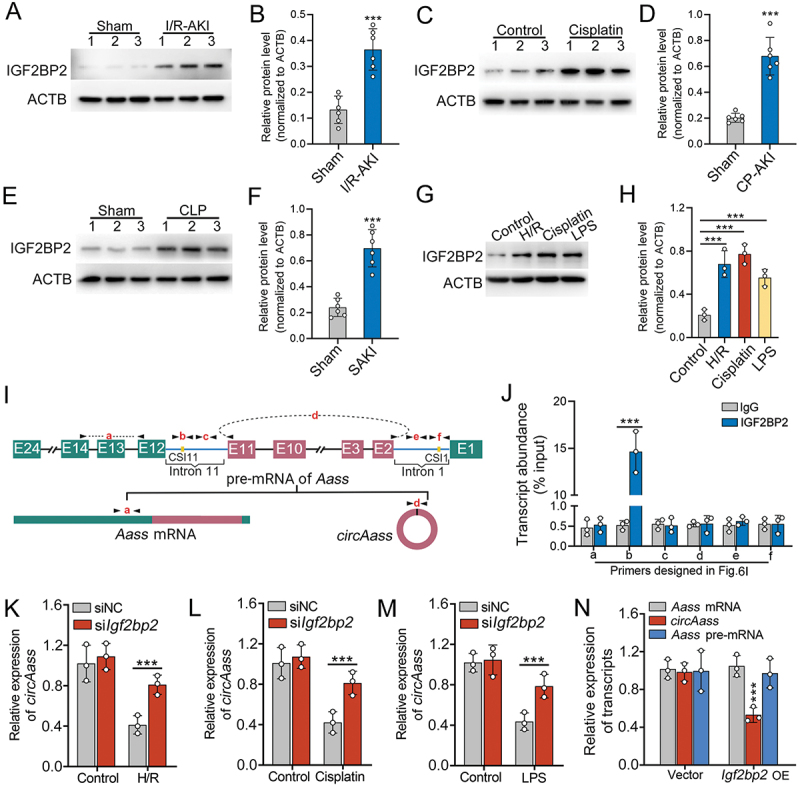
IGF2BP2 functions as a negative regulator of *circAASS* circularization in TECs. (A and B) IGF2BP2 protein expression in kidney tissues from IRI-AKI mice by western blot (A) and quantification analysis (B). (C and D) IGF2BP2 expression in CP-AKI mouse kidney tissues (C, western blot; D, quantification). (E and F) Detection of IGF2BP2 in SAKI mouse kidneys (E, representative blot; F, quantitative data). (G and H) Western blotting showing IGF2BP2 expression in H/R-, cisplatin- or LPS-treated primary TECs, and the quantification data. (I) The primer sets designed in the pre-mRNA of *Aass*. (J) The transcript abundance of amplicons a-f relative to input, detected by rna immunoprecipitation with anti-IGF2BP2 in lysates from primary TECs, followed by qPCR assay. (K-M) The relative levels of *circAass* in primary TECs with *Igf2bp2* knockdown upon H/R (K), cisplatin (L), and LPS (M) treatment. (N) The relative levels of *circAass*, *Aass* mRNA and *Aass* pre-mRNA in primary TECs transfected with *Igf2bp2* overexpression plasmids. Data are presented as the mean ± sd. ***p < 0.001, by 2-tailed Student’s *t* test (B, D, F, J, K, L and M). ***p < 0.001, by 1-way ANOVA with Tukey’s multiple-comparison test (H and N).

To identify candidate RBPs, we screened for *AASS* pre-mRNA interactions using the ENCORI platform [43], which identified 10 RBPs with predicted binding affinity. Of these, TARDBP, ELAVL1, U2AF1, and IGF2BP2 demonstrated particularly strong interactions, each binding to over 10 distinct sites (Figure S5C). Transcriptome analysis in AKI mice revealed IGF2BP2 as the only RBP significantly upregulated, whereas the other three remained unchanged (Table S2). This differential expression was further confirmed in AKI mouse kidney tissues (Figure 6(A-F)). Consistent with these findings, IGF2BP2 upregulation was also observed in primary TECs subjected to H/R, cisplatin, or lipopolysaccharide (LPS) treatment (Figure 6(G,H)).

Consistent with prior reports, the predominant class of human circRNAs derives from internal exons with unusually long flanking introns that characteristically contain inverted complementary sequences. These sequence elements mediate RNA duplex formation that functionally enhances back-splicing, consequently stimulating circRNA biogenesis [44]. We then analyzed the sequence alignment between intron 1 and intron 11 of the *AASS* gene. Notably, we identified highly reverse-complementary regions (71% identity over 180 nucleotides) (Figure S5D). We designated these sequences as CSI1-*AASS* (complementary sequences in intron 1 of *AASS*) and CSI11-*AASS* (complementary sequences in intron 11 of *AASS*), respectively (Figure 6(I)). To confirm IGF2BP2 binding sites on *circAASS* pre-mRNA, we examined the transcript abundance of the corresponding amplicons. The results showed that IGF2BP2 bound to CSI11-*AASS* but not CSI1-*AASS* of *circAASS* pre-mRNA (Figure 6(J)). Strikingly, siRNA-mediated knockdown of *IGF2BP2* in TECs exposed to these injury models restored *circAASS* expression (Figure 6(K-M)). Furthermore, the ectopic expression of *IGF2BP2* downregulated *circAASS* expression in TECs, without affecting the expression levels of *AASS* mRNA and its pre-mRNA (Figure 6(N)). While IGF2BP2 is well-established as a N^6^-methyladenosine (m^6^A) reader that collaborates with the METTL3-METTL14 methyltransferase complexes to mediate m^6^A-dependent RNA regulation [45,46], our findings reveal an m^6^A-independent mechanism for *circAASS* suppression. Knockdown of *METTL3* and *METTL14*, which impairs m^6^A modification on RNA, did not alter *circAASS* expression levels (Figure S5E). This finding provides definitive evidence that the regulation of *circAASS* by IGF2BP2 occurs independently of m^6^A.

### *circAASS* alleviates acute kidney injury and renal inflammation by improving mitochondrial homeostasis in TECs

**Figure 7.**
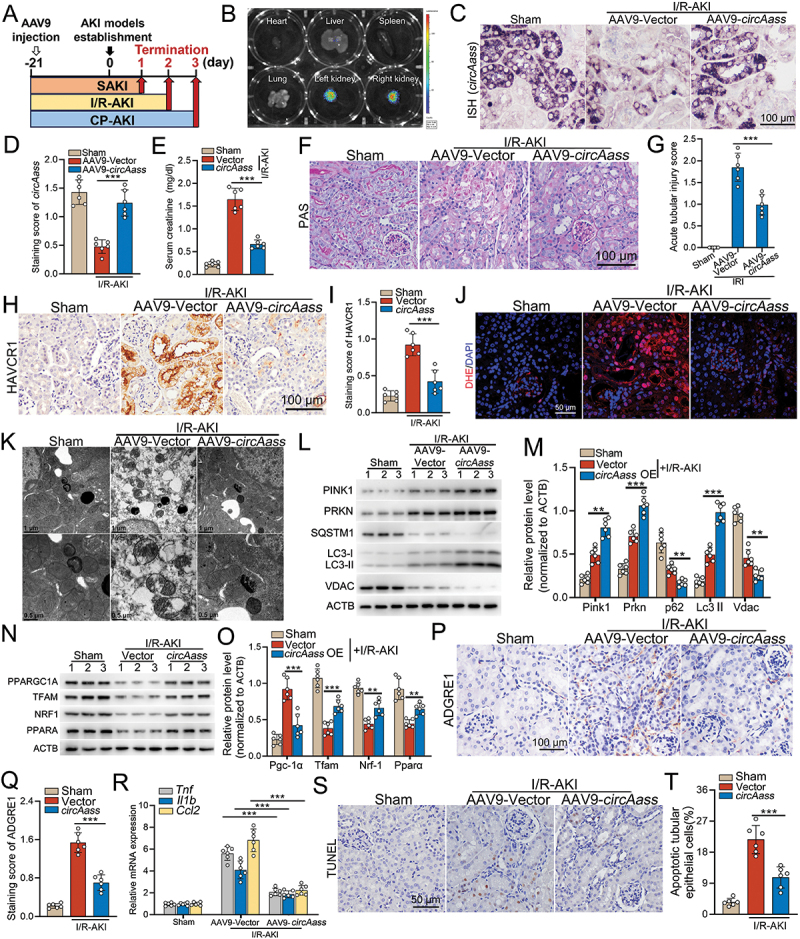
*circAass* alleviates acute kidney injury, renal inflammation by improving mitochondrial homeostasis in TECs. (A) Diagram shows the experimental design. (B) Representative images of luminescent imaging for kidney, heart, liver, lung and spleen from mice treated with AAV9-luciferase-*circAass* through the tail vein. (C and D) Representative images and quantification data of ISH of *circAass* expression in kidneys from mice injected with AAV9-*circAass*. (E) Serum creatinine level in I/R-AKI mice injected with either AAV9-Vector or AAV9-*circAass* 2 days post I/R treatment. (F and G) Representative images and quantification data of PAS staining kidney tissue from mice treated with AAV9-*circAass* 2 days post I/R treatment. (H and I) Representative images and quantification data of IHC staining of HAVCR1/KIM-1 in kidney tissue from mice treated with AAV9-*circAass*, at 2 days post injury. (J) Representative images of DHE staining. DHE nuclear staining indicates the presence of ROS. (K) Representative TEM images showing mitochondrial morphology in TECs from different experimental groups of mice. Higher-magnification insets highlight detailed ultrastructural features of mitochondria, including cristae organization and membrane integrity. (L and M) Western Blotting and quantification data showing the expression levels of PINK1 and other mitophagy-related proteins in kidney cortex. (N and O) Western blotting and quantification data showing the expression levels of mitochondrial biogenesis related proteins in kidney cortex. (P and Q) Representative images and quantification data of IHC staining of ADGRE1/F4/80 in kidney tissue from mice treated with AAV9-*circAass*, at 2 days post injury. (R) qPCR results showing the expression levels of *Tnf*, *Il1b* and *Ccl2* in the kidney cortex. (S and T) Representative images and quantification data of TUNEL staining showed that ectopic expression of *circAass* inhibited TEC apoptosis of AKI mice. Data are presented as the mean ± sd. **p < 0. 01, ***p < 0.001, by 1-way ANOVA with Tukey’s multiple-comparison test (D, E, G, L, M, O and R). ***p < 0.001, by 2-tailed Student’s *t* test (Q and T). Scale bars: 100 μm (C, F, H and P), 50 μm (J and S), 1 μm (K, upper panel) and 500 nm (K, lower panel).

To explore whether *circAASS* regulates mitochondrial homeostasis to alleviate TECs injury, we employed an adeno-associated virus serotype 9 (AAV9) system to overexpress *circAass* under a *Cdh16/Ksp-cadherin* promoter for specifically targeting TECs. Also, a luciferase reporter was co-expressed with ectopic *circAass* to evaluate the delivery efficacy in the kidneys. I/R-AKI mice, CP-AKI or SAKI mice were treated with AAV9 carrying *circAass* three weeks before the establishment of these three models, mice were euthanized at the indicated days post the treatments (Figure 7(A)). The quantified luminescent signals demonstrated that the overexpressed *circAass* were mainly located in the kidney, while a few signals were observed in the liver (Figure 7(B)). Consistently, we found that *circAass* expression in TECs was rescued in I/R-treated mice treated with AAV9-*circAass* injection, as evidenced by *in* *situ* hybridization (ISH) (Figure 7(C,D)).

AAV9-*circAass* injected mice exhibited improved renal function and lower acute tubular injury scores across all three AKI models, as compared to AAV9-Vector controls (Figure 7(E-I)). Mitochondrial function in TECs, as assessed by dihydroethidium (DHE) staining for ROS, was improved in AAV9-*circAass* injected I/R-AKI mice, as compared to the I/R-AKI mice injected with AAV9-Vector (Figure 7(J)). Consistently, restoring *circAass* expression alleviated the mitochondrial damage in TECs from I/R-AKI mice (Figure 7(K)). Also, elevated expression of PINK1 and other mitophagy-associated proteins were observed in the kidneys from I/R-AKI mice overexpressed with *circAass* (Figure 7(L,M)). In addition, I/R-treated mice overexpressing *circAass* showed restored PPARGC1A/PGC-1α expression and its downstream effectors of mitochondrial biogenesis in the kidneys compared to levels in I/R-treated mice administered the AAV9-empty vector (Figure 7(N,O)). Consistently, ectopic expression of *circAass* decreased the infiltration of macrophages and inhibited the expression of pro-inflammatory molecules (*Il1b*, *Tnf* and *Ccl2*) (Figure 7(P-R)), inhibited the apoptosis of TECs in renal cortex from I/R-treated mice administered the AAV9-*circAass* (Figure 7(S-T)). Similar results about exogenous *circAass*-mediated mitochondrial homeostasis improvement and anti-apoptotic effect in injured TECs were found in AAV9-*circAass* injected mice subjected to CP-AKI or SAKI (Figure S6A-L).

### Ectopic expression of *circAass* inhibits tubulointerstitial fibrosis post I/R- or CP-induced AKI

**Figure 8.**
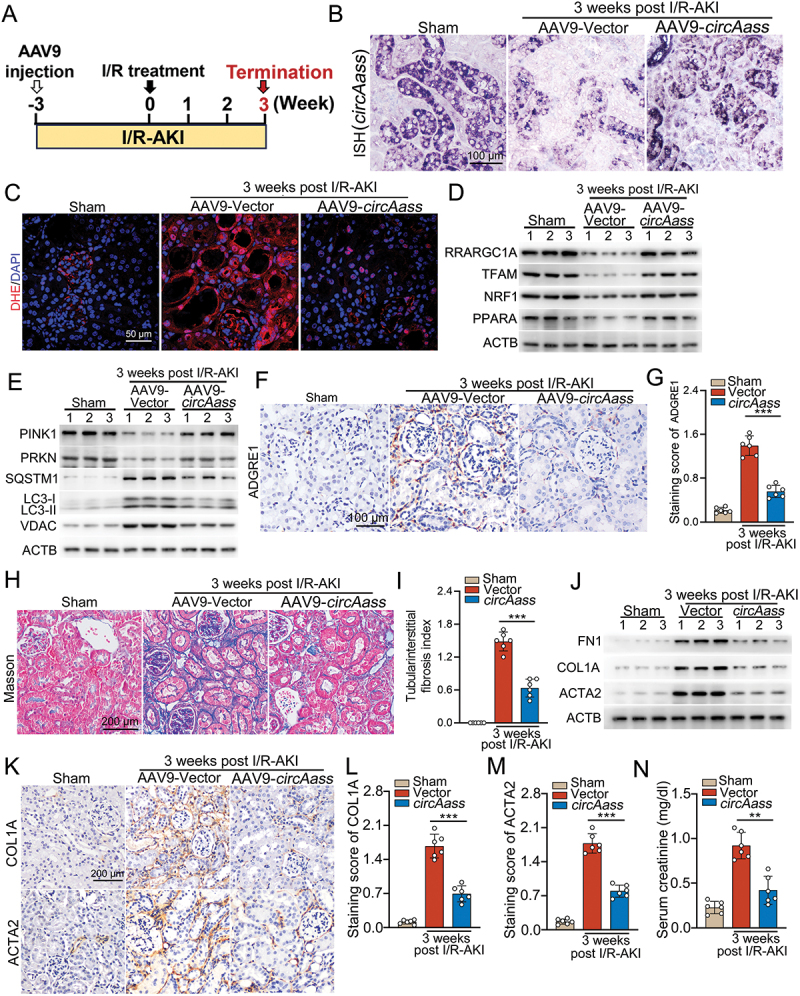
Ectopic expression of *circAass* inhibits tubulointerstitial fibrosis post I/R- or CP-induced AKI. (A) Diagram shows the experimental design. (B) Representative images of ISH of *circAass* expression in kidneys from mice treated with AAV9-Vector or AAV9-*circAass* 3 weeks post-I/R treatment. (C) DHE staining shows that overexpression of *circAass* decreased the levels of ROS 3 weeks post-I/R treatment. (D) Western blotting shows that overexpression of *circAass* decreased the expression of PPARGC1A/PGC-1α and other mitochondrial biogenesis-associated molecules 3 weeks post-I/R treatment. (E) Western blotting shows that overexpression of *circAass* decreased the expression of PINK1 and other mitophagy-associated molecules 3 weeks post-I/R treatment. (F and G) Representative images and quantification data of IHC staining of ADGRE1/F4/80 in kidney tissue from I/R mice treated with AAV9-Vector or AAV9-*circAass* 3 weeks post-I/R treatment. (H and I) Representative images and quantification data of Masson’s trichrome staining in I/R mice injected with either AAV9-Vector or AAV9-*circAass* 3 weeks post-I/R treatment. (J) Western blot shows that overexpression of *circAass* decreased the expression of profibrotic factors 3 weeks post-I/R treatment. (K-M) Representative images and quantification data of IHC staining in I/R mice injected with either AAV9-Vector or AAV9-*circAass* 3 weeks post-I/R treatment. (N) Serum creatinine level in I/R-AKI mice injected with either AAV9-Vector or AAV9-*circAass* 3 weeks post I/R treatment. Data are presented as the mean ± sd. **p < 0. 01, ***p < 0.001, by 1-way ANOVA with Tukey’s multiple-comparison test (G, I, L, M and N). Scale bars: 100 μm (B and F), 50 μm (C), 200 μm (H and K).

For further exploring the role of ectopically expressed *circAass* in tubulointerstitial fibrosis, I/R-AKI model and repeated low-dose CP-induced AKI model were chosen because these two models were widely used to investigate tubulointerstitial fibrosis post AKI [47], while seldom study on tubulointerstitial fibrosis could be accomplished in SAKI model. To this end, AAV9-*circAass* was injected three weeks before these two animal models were built, mice were euthanized at the indicated days (Figure 8(A), S7A). As shown in Figure 8(B) and Figure S7B, the *circAass* expression was greatly restored in kidney sections from AAV9-*circAass*-treated mice subjected to I/R treatment or CP injection. As reflected by the staining of DHE, the mitochondrial function of TECs were improved in AAV9-*circAass* injected I/R-AKI and CP-AKI mice, as compared to the mice injected with AAV9-Vector (Figure 8(C), S7C). Consistently, the restored expression of PPARGC1A/PGC-1α and other mitochondrial biogenesis-associated molecules in the renal cortex was found in AAV9-*circAass*-treated mice subjected to I/R treatment (Figure 8(D)). Furthermore, western blot analysis of key mitophagy markers revealed that *circAass* overexpression upregulated PINK1 and PRKN, concomitantly increased LC3-II accumulation, and downregulated SQSTM1/p62 in kidneys at 3 weeks post-I/R injury (Figure 8(E)). This coordinated expression pattern of mitophagy-related proteins suggests an enhancement of mitophagic activity. Together, these results demonstrate that *circAass* promotes the molecular signature associated with improved mitochondrial homeostasis. Consistently, *circAass* overexpression ameliorated inflammation (Figure 8(F,G)) and tubulointerstitial fibrosis (Figure 8(H,I); Figure S7D, E), decreased pro-fibrotic molecules (ACTA2/αSMA, FN1 and COLIA) (Figure 8(J-M); Figure S7F-I) and improved renal function in I/R-AKI (Figure 8(N)) and CP-AKI mice.

### Downregulation of *circAASS* associates with disruption of mitochondrial homeostasis in AKI and CKD

To investigate the clinical relevance of *circAASS* in human kidney disease, we conducted a cross-sectional analysis correlating *circAASS* expression levels with parameters of mitochondrial homeostasis in renal biopsies from AKI (*n* = 17) and CKD (*n* = 15) patients. The characteristics of patients at the time of biopsy are summarized in Table S4.

**Figure 9.**
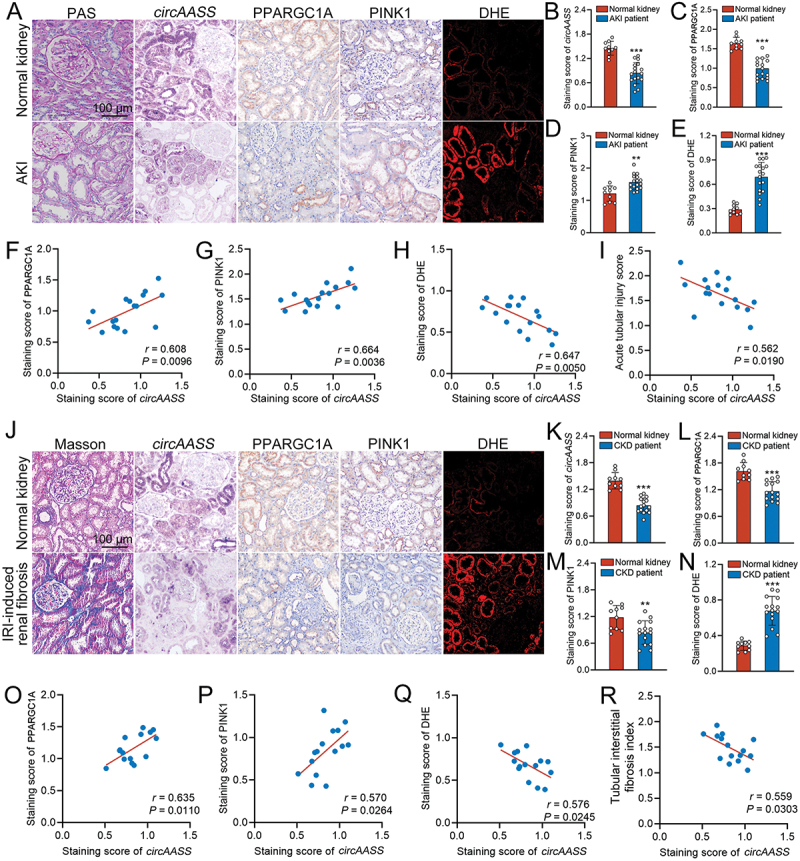
Downregulation of *circAASS* associates with mitochondrial dyshomeostasis in patients with AKI and CKD. (A-E) Representative images of kidney sections staining with PAS staining, ISH for *circAASS*, IHC staining for PPARGC1A/PGC-1α or PINK1, DHE staining in renal biopsy samples from patients with AKI (A), and quantification data (B-E). (F-I) Pearson correlation analyses for association between intrarenal *circAASS* with PPARGC1A/PGC-1α expression score (F) or PINK1 expression score (G) or DHE staining score (H) or acute tubular injury score (I). (J-N) Representative images of kidneys with Masson staining, ISH for *circAASS*, IHC staining for PPARGC1A/PGC-1α or PINK1, DHE staining in renal biopsy samples from patients with renal fibrosis (J), and quantification data (K-N). (O-R) Pearson correlation analyses for association between intrarenal *circAASS* with PPARGC1A/PGC-1α expression score (O) or PINK1 expression score (P) or DHE staining score (Q) or tubular interstitial fibrosis index (R). Data are presented as the mean ± sd. **p < 0. 01, ***p < 0.001, by 2-tailed Student’s *t* test (B, C, D, E, K, L, M and N). Scale bars: 100 μm (A and J).

To observe the involvement of intrarenal *circAASS* in AKI, we evaluated the expression of *circAASS*, PPARGC1A/PGC-1α, and PINK1. Normal kidneys showed abundant expression of *circAASS* and moderate levels of PPARGC1A/PGC-1α, both of which were significantly downregulated in AKI. Meanwhile, PINK1 expression was upregulated under AKI conditions (Figure 9(A-D)). On the other hand, DHE staining indicated elevated ROS levels in TECs of AKI patients, reflecting impaired mitochondrial function (Figure 9(A,E)). Furthermore, correlation analyses within the AKI cohort revealed that *circAASS* expression showed a positive correlation with both PPARGC1A/PGC-1α and PINK1 levels (Figure 9(F,G)). Specifically, the positive correlation with PINK1 suggests that despite the overall downregulation of *circAASS* in disease, its expression levels among patients remain coordinated with those of PINK1. Additionally, *circAASS* expression showed negative correlations with DHE staining scores (Figure 9(H)) and acute tubular injury scores (Figure 9(I)).

We further examined the expression levels of *circAASS*, PPARGC1A/PGC-1α, and PINK1 in renal tissues from CKD patients. Consistent with the observations in AKI patients, CKD patients exhibited reduced expression of *circAASS*, PPARGC1A/PGC-1α, and PINK1, along with elevated ROS levels in TECs (Figure 9(J-N)). Correlation analysis revealed a positive association between *circAASS* expression and both PPARGC1A/PGC-1α and PINK1 levels (Figure 9(O,P)), whereas *circAASS* expression was inversely correlated with DHE staining scores (Figure 9(Q)) and the tubulointerstitial fibrosis index (Figure 9(R)). These findings further support the notion that downregulation of *circAASS* contributes to both AKI and CKD progression by impairing mitochondrial homeostasis in TECs.

## Discussion

CKD is a common kidney disorder that seriously endangers human health and has become a global public health issue. AKI independently predisposes to CKD development, wherein unresolved inflammation and fibrosis secondary to TEC injury constitute the central pathogenic cascade [48–50]. TECs are the main cell type in the renal cortex and among the most severely affected in AKI. An increasing number of studies conclude that TECs are not just victims of incomplete repair after AKI but also initiators driving sustained renal inflammation and progressive fibrosis [12,51,52]. Therefore, protecting TECs from injury is crucial for preventing CKD initiation and progression. Here, our study identifies *circAASS* as a conserved RNA that suppresses mitochondrial dysfunction and apoptosis in renal tubular cells. Importantly, therapeutic restoration of *circAASS* expression demonstrates remarkable efficacy in mitigating tubular injury and renal fibrosis across multiple etiologies of AKI.

With the advancement of high-throughput sequencing and microarray technologies, many dysregulated circRNAs have been identified in various mouse AKI models, providing potential targets for further investigation [24,25]. Comprehensive integration of these dysregulated circRNA datasets aids in discovering candidate circRNAs with broad-spectrum anti-AKI effects. In this study, *circAass* and *circLrrk2* were downregulated in AKI mice with three different etiologies. Due to its higher human-mouse conservation, *circAASS* was selected for further investigation given its clinical translational potential. Considering the central role of mitochondrial dysfunction in tubular injury [27,53], we demonstrated that *circAASS* restoration attenuates apoptosis and inflammation through mitochondrial protection and ROS reduction.

Mechanistically, circRNAs function through diverse pathways including miRNA sponging, RBP interactions, transcriptional regulation, and in some cases, peptide translation [54]. In line with localization-dependent functionality, we found nuclear and cytoplasmic *circAASS* exhibit distinct roles. Specifically, cytoplasmic *circAASS* acts as a *MIR324-3p* sponge to upregulate PINK1, which is a key mitophagy regulator crucial for mitochondrial quality control in AKI [13,35,53]. This *circAASS*/*MIR324-3p*/PINK1 axis constitutes a key cytoprotective pathway in TECs by preserving mitochondrial integrity. Importantly, we further identified that *circAASS* physically interacts with PPARGC1A/PGC-1α, potentially stabilizing it against ubiquitin-proteasomal degradation to enhance mitochondrial biogenesis. These complementary mechanisms, coordinating both mitochondrial clearance (*via* PINK1) and regeneration (*via* PPARGC1A/PGC-1α), establish *circAASS* as a key coordinator of mitochondrial homeostasis in AKI. While our current study demonstrates the therapeutic potential of AAV-mediated *circAass* overexpression in mitigating AKI and renal fibrosis, future studies should directly compare its efficacy with existing therapeutic strategies, such as mitophagy activators or mitochondrial biogenesis enhancers, to better understand where *circAASS*-based therapy fits into future treatment options.

The protective role of the PINK1-PRKN pathway in mitophagy during renal injury, including AKI, has been well established in previous high-quality studies [14,55]. However, the upstream regulatory mechanisms, particularly those governing the post-transcriptional regulation of PINK1 expression, remain poorly understood. In this study, we demonstrate that cytoplasmic *circAASS* upregulates *PINK1* mRNA expression by sequestering *MIR324-3p*. Interestingly, elevated PINK1 levels were observed in H/R-treated TECs, as well as in mouse models of both AKI and CKD and kidney tissues from AKI patients. This widespread upregulation suggests a possible stress-induced compensatory response aimed at mitigating mitochondrial damage. The positive correlation between *circAASS* and PINK1 expression further implies that higher *circAASS* levels may enhance this compensatory capacity.

PPARGC1A/PGC-1α is a short-lived protein whose cellular abundance is tightly regulated by ubiquitination and subsequent proteasomal degradation. We found that nuclear *circAASS* directly interacts with the C-terminal of PPARGC1A/PGC-1α (aa 566–798), thereby inhibiting its degradation. This finding is consistent with previous reports indicating that the N-terminal region (aa 1–565) of PPARGC1A/PGC-1α is resistant to degradation and unaffected by MG132 treatment [56], underscoring the critical role of the C-terminal region in regulating PPARGC1A/PGC-1α stability. Importantly, since RNF34 is known to target this same region [38–40], we propose a competitive binding mechanism whereby *circAASS* stabilizes PPARGC1A/PGC-1α during AKI by displacing RNF34. Supporting this model, we observed that *circAASS* significantly reduced the interaction between PPARGC1A/PGC-1α and RNF34 in injured TECs. Furthermore, rescue experiments demonstrated that knocking down RNF34 abolishes the effects of *circAASS* depletion on PPARGC1A/PGC-1α ubiquitination, providing functional validation of this regulatory mechanism. These findings reveal a competitive RNA-protein interaction in which *circAASS* antagonizes RNF34-mediated ubiquitination to stabilize PPARGC1A/PGC-1α, offering new insights into post-translational regulation during AKI. Pharmacological modulation of this competition may represent a promising renoprotective strategy.

Notably, our study revealed a selective downregulation of *circAASS* in injured TECs, while *AASS* pre-mRNA and mRNA levels remained unchanged. This distinct expression pattern suggests that *circAASS* biogenesis from its parental pre-mRNA may be suppressed through RBP-mediated posttranscriptional regulation [12,57], rather than transcriptional control of the *AASS* gene. Through bioinformatic screening and transcriptomic validation, we identified IGF2BP2 as the candidate regulator. Although IGF2BP2 is best known as an m^6^A reader protein [58,59], we discovered it directly binds intron 11 of *Aass* pre-mRNA to suppress *circAass* formation. To our knowledge, this represents the first report of IGF2BP2 inhibiting circRNA biogenesis, thereby expanding its functional repertoire beyond m^6^A-mediated regulation. Further studies should explore whether this mechanism applies to other circRNAs.

Although several circRNAs have been implicated in kidney pathologies [9,60,61], their specific roles in mitochondrial dysfunction during AKI remain poorly understood. Our study addresses this critical gap by identifying *circAASS* as a key regulator of mitochondrial homeostasis, highlighting its potential as a therapeutic target for attenuating kidney injury and renal fibrosis. To restore *circAASS* expression *in vivo*, we employed an AAV9 system [12,36] with a kidney-specific promoter (*Cdh16/Ksp-cadherin*) [62] to achieve ectopic expression of *circAASS* specifically in TECs, as demonstrated by luminescent imaging (Figure 7B). While short-term interventions showed no signs of acute toxicity, further investigations are needed to evaluate the long-term safety of AAV-mediated *circAASS* overexpression, including potential immune reactions, off-target effects, and organ-specific toxicity. Future clinical studies are also warranted to validate *circAASS* as both a diagnostic biomarker and a therapeutic target. Moreover, interventional studies utilizing *circAASS*-based strategies in large animal models will be essential to facilitate the translation of these preclinical findings into clinical applications.

**Figure 10.**
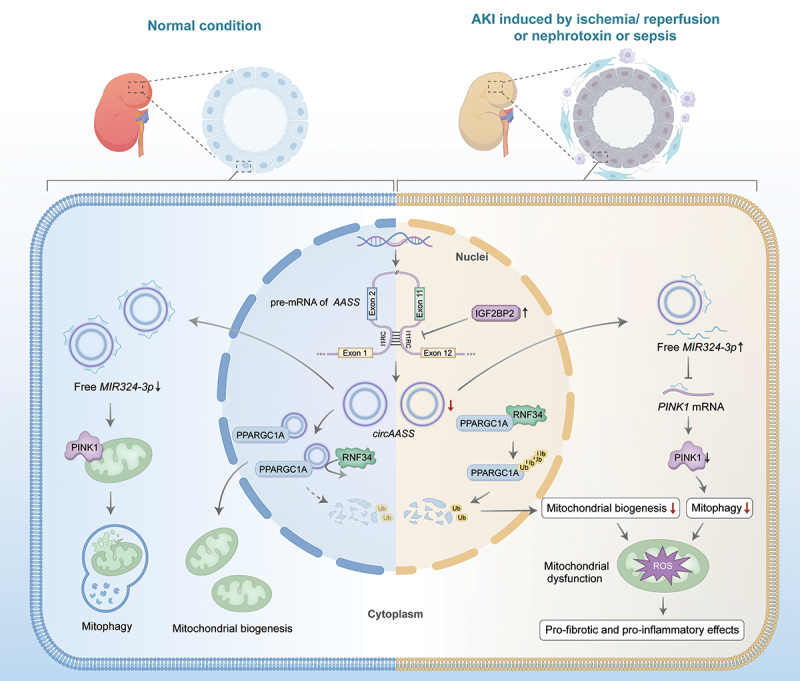
Schematic illustration of the mechanism whereby *circAASS* regulates mitochondrial homeostasis in kidney injury and renal fibrosis. Under normal conditions, cytoplasm-localized *circAASS* acts as a ceRNA by sequestering *MIR324-3p*, thus maintaining the expression of PINK1 and promoting mitophagy; nucleus-localized *circAASS* directly interact with the PPARGC1A/PGC-1α protein, inhibiting its ubiquitin-mediated degradation and thereby facilitating post-translational modifications that promote mitochondrial biogenesis. Under Aki conditions, low-expression of *circAASS* inhibits mitophagy and mitochondrial biogenesis, thereby causing pro-fibrotic and pro-inflammatory effects of TECs.

In summary, we demonstrate that cytoplasmic *circAASS* sustains PINK1 expression by acting as a molecular sponge for *MIR324-3p*. Additionally, nuclear *circAASS* functions as a previously unrecognized natural agonist that inhibits RNF34-mediated degradation of PPARGC1A/PGC-1α in TECs (Figure 10). Collectively, our findings demonstrate that *circAASS* restoration enhances renal resilience against injury by coordinating mitochondrial homeostasis, thereby highlighting its potential as a novel therapeutic strategy.

## Materials and methods

### Human study participants and human tissue samples

The observational experiment of this study was approved by the Clinical Research Ethics Committee of the Affiliated Hospital of Guangdong Medical University (Approval no. PJKT2025-071). Patients with biopsy-confirmed AKI or subsequent fibrosis attributable to ischemic reperfusion injury were enrolled. Detailed clinical characteristics and underlying causes, such as septic shock or postsurgical complications, are summarized in Table S4.

Eligibility for enrollment required a diagnosis of AKI according to KDIGO criteria, along with biopsy-confirmed acute tubular injury for the AKI group or tubulointerstitial fibrosis for the fibrosis group, both attributable to IRI. Patients were excluded if they had preexisting or concurrent renal diseases unrelated to IRI, such as diabetic nephropathy or glomerulonephritis. A total of 17 patients were included in the IRI-AKI group and 15 in the IRI-fibrosis group. Normal control tissues were obtained from non-tumorous regions adjacent to renal cell carcinomas in patients with clear cell renal cell carcinoma (*n* = 10). Written informed consent was provided by all participants.

### Retrieving dysregulated circRNAs from publicly available data

To identify commonly dysregulated circular RNAs (circRNAs) in renal cortical tissues from three AKI mouse models, we conducted a comprehensive analysis incorporating our own datasets (I/R-AKI: GSE286507; SAKI: GSE220782) alongside one publicly available dataset: CP-AKI (BioProject ID PRJNA806364).

Initially, we retrieved raw circRNA expression profiles from these datasets, which were generated through high-throughput technologies, including both sequencing and microarray analysis. After data acquisition, we performed quality control to filter out low-quality samples and ensure the integrity of the datasets. We then normalized the circRNA expression data using appropriate statistical methods to account for batch effects and variations across different studies.

Subsequently, we compared the precursor mRNA gene names associated with the identified circRNAs, as well as their lengths, to establish a framework for identifying common dysregulated circRNAs. We employed differential expression analysis using statistical software, applying stringent criteria that included a false discovery rate (FDR) adjusted p-value of < 0.05 and a fold change of ≥2. Our analysis revealed that *circAASS* and *circLRRK2* were consistently dysregulated across all three datasets. This systematic approach not only highlighted the commonalities in circRNA expression profiles among the different AKI models but also provided insights into the potential functional roles of these circRNAs in the context of AKI.

### Cell culture and treatment

Human renal proximal tubular epithelial cell line (HK2, CRL-2190), human embryonic kidney 293 cells (HEK-293, CRL-1573), and fibroblasts (NRK49F, CRL-1570) were commercially available from American Type Culture Collection. For cell cultivation, HK2 cells were cultured in Dulbecco’s modified Eagle’s medium (DMEM)-Ham’s F12 medium (Invitrogen, 11320033), which contained 10% FBS (Invitrogen, 10099141C). HEK-293 cells were maintained in complete medium composed of basic DMEM (Invitrogen, 11965092) and 10% FBS. These cells were maintained in a Thermo Fisher Scientific incubator under standard normal condition (5% oxygen and 5% carbon dioxide).

Primary mouse renal tubular cells were isolated *via* enzymatic digestion and density gradient centrifugation. Renal cortices were dissected into 1–2 mm^3^ fragments and digested with 0.75 mg/ml collagenase type I (Thermo Fisher Scientific, 17104019) at 37°C for 40 min. The suspension was filtered through 100 μm and 40 μm strainers (Fisherbrand, 22–363-549 and 22–363-547) and centrifuged with 31% Percoll (GE Healthcare, 17–0891-01) at 800×g for 20 min. After PBS (Boster, AR0030) washing, cells were cultured in collagen-coated dishes (Thermo Fisher Scientific, A1142801) with DMEM containing 10% FBS, antibiotics, and Insulin-Transferrin-Selenium (Thermo Fisher Scientific, 41,400,045), maintained at 37°C, 5% CO₂. Medium was renewed every 48 h initially, then triweekly. Cultures reached 60–80% confluency within 4–8 days.

This study utilized three distinct treatments to develop an injured tubular epithelial cells (TECs) model that mimics AKI induced by I/R, cisplatin, or sepsis. Briefly, after overnight starvation in DMEM/F12 basic medium containing 0.5% FBS, TECs were subjected to hypoxia for 24 h and reoxygenation for 12 h, or treated with cisplatin (Selleck, S1166; 20 μM) or LPS (Sigma-Aldrich, L2630, 10 μg/ml) for the indicated time points. For the observation of profibrotic effects of TECs, the reoxygenation duration in the H/R model was extended to 48 h post hypoxia. Also, the duration for cisplatin treatment was extended to 48 h.

### Cell transfection

Plasmids, siRNAs and miRNA mimics were synthesized by Shanghai GenePharma Biotechnology Co., Ltd. HK2 cells were cultured in 6-well plates overnight and transfected with Lipofectamine 2000 (Invitrogen, 11668019) according to the manufacturer’s instructions. The plasmids, siRNA sequences and miRNA mimics used in this study are listed in Table S5.

### *circAASS* overexpression

To achieve *circAASS* overexpression, the full-length sequence of *circAASS* was cloned into the pLO5-ciR vector (Geneseed Biotech, GS0107) at the EcoRI and BamHI restriction sites. Lentivirus production was conducted in HEK-293T cells co-transfected with the pLO5-ciR-*circAASS* vector and packaging plasmids. After 60 h, the harvested viral supernatant was filtered through 0.45-μm filters (Merck Millipore, HAWG047500) to remove debris and obtain infectious lentivirus.

Subsequently, target cell lines were infected with the lentivirus for 24 h, followed by selection with 2 μg/ml puromycin for one week to isolate stable positive clones expressing *circAASS*. Following transfection, cells were cultured for 48 hours before analysis to assess the overexpression efficiency of *circAASS*.

### Animal models of AKI

All animal experiments were performed according to the Guide for the Care and Use of Laboratory Animals. In the *in vivo* experiments, only male mice were used to minimize variability associated with the female reproductive cycle, as previously described [63]. Male C57BL/6J mice (6–8 weeks old, approximately 20 to 22 g) were provided by SPF (Beijing) biotechnology. All animal experiments were approved by the Institutional Animal Care and Use Committee of the Affiliated Hospital of Guangdong Medical University (Approval No. 202209-0052).

For the induction of I/R-induced AKI, renal I/R surgery was performed as previously described [12]. In brief, the mice were anesthetized and placed on a thermostat plate to maintain body temperature at 37–38°C. Bilateral renal pedicles were clamped for 35 min using microaneurysm clamps, followed by reperfusion. Sham mice were subjected to the identical surgical procedures without renal pedicle clamping. All mice were euthanized under anesthesia two days post-I/R for tissue and blood collection.

For CP-AKI, the mice were intraperitoneally injected with a single dose of cisplatin (20 mg/kg body weight). Control mice received an intraperitoneal injection of an equivalent volume of 0.9% saline solution. All animals were euthanized under anesthesia three days after injection for subsequent analyses.

For the sepsis-induced AKI model, mice underwent cecal ligation and puncture (CLP) surgery as previously described [25]. Sham mice underwent the same surgery, with the exception of ligation or puncture. All mice were euthanized under anesthesia 1 day post CLP treatment for biological sample collection.

### Animal models of renal fibrosis post AKI

To investigate the role of *circAASS* on renal fibrosis post AKI, I/R-AKI and CP-AKI were conducted in C57BL/6J mice. Briefly, for studying renal fibrosis post I/R-AKI, bilateral renal pedicles were clamped for 35 min using microaneurysm clamps, and the mice were euthanized at day 21 following I/R injury. Sham mice were subjected to the same surgery except clamping the bilateral renal pedicles. For studying renal fibrosis post CP-AKI, repeated low-dose cisplatin injection was conducted, as described previously [64], with minor modification. Mice were injected intraperitoneally with 8 mg/kg cisplatin or saline as control, once a week for 4 consecutive weeks. Mice were euthanized four weeks after the last treatment with cisplatin.

### Measurement of serum creatinine

Serum creatinine was detected with Creatinine (Cr) Assay Kit (C011-2–1) purchased from Nanjing Jiancheng Bioengineering Institute according to the manufacturer’s instructions. The levels of serum creatinine were expressed as milligrams per deciliter.

### Periodic acid-schiff (PAS) staining and acute tubular injury score

Periodic Acid Schiff (PAS) staining was performed with a PAS staining kit (Solarbio, G1280) to evaluate the degree of renal tubular injury at the renal cortex in mouse kidney sections, as previously reported [65]. All assessments were done by two investigators blinded to experimental conditions.

### Masson staining and degree of tubulointerstitial fibrosis

The kidney sections (3 μm) were stained with Masson’s trichrome (Jiancheng Bioengineering Institute, D026-1–3) according to the manufacturer’s protocol and scored by an experienced pathologist who was blinded to the grouping. The degree of tubulointerstitial fibrosis for each sample was scored according to the fibrotic area: 0, no evidence of tubulointerstitial fibrosis; 1, < 25% involvement; 2, 25–50% involvement; and 3, > 50% involvement. The score of each kidney sample was presented as the mean of at least 10 random high-power (400×) fields.

### TUNEL assay

For terminal deoxynucleotidyl transferase dUTP nick end labeling (TUNEL) staining, a DeadEnd Fluorometric TUNEL System was applied according to manufacturer’s instructions (Beyotime, C1098). The degree of TECs apoptosis for each sample was quantified by an experienced pathologist with Image-Pro Plus software and presented as the average percentage of at least 10 random high-power fields.

### Immunohistochemistry

Renal tissue samples from mice or humans were fixed in 4% paraformaldehyde and embedded in paraffin blocks. The samples were treated with specific antibodies against ADGRE1/F4/80 (Cell Signaling Technology [CST], 70076S), PPARGC1A/PGC-1α (NOVUS, NBP1-04676), PINK1 (NOVUS, BC100-494), COL1A/collagen I (Abcam, ab316222), ACTA2/αSMA (Abcam, ab7817) and HAVCR1/KIM-1 (Abcam, ab78494) overnight at 4°C. Then, the samples were incubated with a secondary antibody (ZSGB-Bio, PV-6001; PV-6002) for 1 h. The results were visualized using the optical microscope (Olympus). For protein staining quantification, we evaluated at least 10 random visual fields per sample and computed the score using the formula: 3×high% + 2×positive% + 1×low%, with results expressed as mean values.

### Dihydroethidium (DHE) staining

ROS levels in renal tissues were measured using the fluorescent probe DHE (YEASEN, 50102ES25). Frozen sections were prepared from harvested kidneys and incubated with DHE working solution at 37°C for 60 min in the dark. Intracellular ROS production was simultaneously assessed using the DCFH-DA probe (Beyotime, S0034M). After DAPI staining and mounting, images were acquired with a confocal laser scanning microscope (Olympus, Japan). For quantification of DHE staining score, we evaluated at least 10 random visual fields per sample and computed the score using the formula: 3×high% + 2×positive% + 1×low%, with results expressed as mean values.

### Spatiotemporal tracking of *circAASS* via luminescent imaging

To assess the success of *Cdh16/Ksp-cadherin* promoter-driven *circAass* overexpression in the kidney, we constructed a *Cdh16/Ksp-cadherin*-Nanoluc-*circAass* vector and packaged it into AAV9. The recombinant AAV9-Nanoluc-*circAass* was delivered systemically *via* tail vein injection in mice. Three weeks post-injection, mice were intraperitoneally administered D-Luciferin (YEASEN, 40902ES02) 10 min prior to euthanasia. Organ-specific bioluminescence signals in the kidney, heart, liver, spleen, and lung were then quantified using an IVIS Lumina III Imaging System (Caliper Life Science, USA).

### Transmission electron microscopy (TEM)

Transmission electron microscopy (TEM) was performed as follows. Fresh kidneys and HK2 cells were harvested and cut into 1-mm^3^ pieces, prefixed in 2% glutaraldehyde, and then fixed in 1% osmium tetroxide. Subsequently, after dehydration in ethanol with 3% uranyl acetate, samples were embedded in epoxy resin (Aladdin, R303195) and propylene oxide (Aladdin, P109311) overnight, and polymerized. After slicing into 70-nm-thick sections and staining with lead citrate, the sections were examined by JEM-1400HC transmission electron microscope (JEOL, Japan). For quantitative analysis, the number of intact mitochondria per square micrometer was calculated. For each experimental group, at least six random fields of view were analyzed per independent experiment, and the average value was determined. This process was repeated for three independent experiments.

### Western blot analysis

Renal tissue or cells were lysed in RIPA (Beyotime, P0013B) containing protease and phosphatase inhibitors (Thermo Fisher Scientific, 78446). After the quantification of protein concentrations, equal amounts of protein were electrophoresed on 10% SDS-PAGE gels and transferred onto PVDF membranes (Millipore, ISEQ00010). The membranes were blocked with 5% defatted milk at room temperature for 1 h, then incubated with primary antibodies overnight at 4°C. After incubation with the indicated HRP-labeled secondary antibodies (Proteintech, SA00001-1; SA00001-2) for 1 h, the membranes were detected by Western ECL substrate (Bio-Rad, 1705061). The primary antibodies used were as follows: anti-FN1/fibronectin 1 (Sigma-Aldrich, F3648), anti-COL1A/collagen I (CST, 72026S; Abcam, ab34710), anti-ACTA2/αSMA (CST, 19245S), anti-PINK1 (CST, 6946S), anti-PPRGC1A/PGC-1α (Novus Biologicals, NBP1-04676), anti-SQSTM1/p62 (Abcam, ab56416), anti-LC3-I/II (MBL, PM036), anti-PRKN (CST, 4211S), anti-COX4/Cox IV(CST, 4850S), anti-VDAC1 (Abcam, ab14734), anti-TFAM (Abcam, ab307302), anti-PPARA/PPARα (Novus Biologicals, NB300-537), anti-NRF1 (Abcam, ab175932), anti-RNF34 (Novus Biologicals, NBP2-56413), anti-ACTB/β-actin (CST, 4970S), anti-ubiquitin (Santa Cruz Biotechnology, sc-8017HRP). The ImageJ program was used to analyze images quantitatively.

### RNA extraction and quantitative real-time polymerase chain reaction

Total RNA was extracted from tissues and cells by Trizol reagent (Invitrogen, 15596018CN). The Prime Script RT Master Mix (Takara, RR036A) was used to reverse transcription of RNA into cDNA. Then qPCR was performed on Cobas Z480 qPCR (Roche) using qPCR SYBR Green Master Mix (Vazyme, Q711-03). All primers used in this study were synthesized by Sangon Biotech (Shanghai, China), and the sequences of primers are listed in Table S5. Each sample was replicated at least three times and data were analyzed by the 2^−ΔΔCT^ statistical method.

### Agarose gel electrophoresis

Agarose gel electrophoresis was performed using 1.2% agarose gels prepared in 1×TAE buffer (Biosharp, BL533A). DNA samples were electrophoresed at 140 V for 25 min, and DNA bands were visualized using an ultraviolet gel imaging system (Bio-Rad, ChemiDoc XRS+).

### RNase R and actinomycin D treatment

For RNase R treatment, total RNA (2 μg) extracted from HK2 cells was incubated for 20 min at 37°C with 3 U/μg of RNase R (Epicenter Technologies, LGCRNR07250). For the actinomycin D assay, HK2 cells were treated with 2 μg/ml actinomycin D (Sigma-Aldrich, A4262) for the indicated time, then RNA was collected at the indicated time points. After treatment with actinomycin D and RNase R, RNA expression levels of *circAASS* and *AASS* mRNA were detected by qPCR.

### Flow cytometric analysis

The mitochondrial membrane potential (MMP) was determined with a JC-1 assay kit (Beyotime, C2006). In addition, ROS were analyzed using a ROS assay kit (Beyotime, S0033S). Apoptosis was assessed using the Annexin V-FITC/PI Apoptosis Kit (MULTISCIENCES, AT101). These experiments were performed according to the manufacturer’s instructions. Stained cells ( > 10,000) were examined by using a FACS flow cytometer (Beckman CytoFLEX, Beckman Coulter Life Sciences, USA). Flow Jo software was used to analyze the experimental data.

### Dual-luciferase reporter system

To investigate whether *circAASS* and PINK1 are regulated by *MIR324-3p* or *MIR345-5p*, we performed luciferase reporter assays. The full-length sequence of *circAASS* and the 3’ UTR of PINK1 were cloned into the psi-CHECK2 dual-luciferase reporter vector (Promega, C8021). To further validate the specific binding of *MIR324-3p* to these sequences, we generated mutant constructs with altered *MIR324-3p* binding sites and similarly cloned them into the psi-CHECK2 vector. All luciferase assays were performed according to the manufacturer’s protocol (Promega, N1610).

### RNA fluorescent in *in situ* (RNA-FISH) and RNA *in* *situ* hybridization (RNA-ISH)

#### RNA FISH in cultured cells

To detect the expression of *circAASS* in HK2 cells, LNA-modified probes specifically targeting *circAASS* were synthesized based on the sequences provided in Table S5. The main procedures are as follows: HK2 cells were washed with PBS (Boster, AR0030), followed by fixation in 4% formaldehyde. To ensure access for the probe, permeabilization was carried out using PBS containing 0.1% Triton X-100 (Boster, AR0205). Cells on coverslips were then hybridized with the *circAASS* probes within a humid chamber at 52°C for 16 h in a hybridization solution with a 1:1000 dilution of the probe. After stringent washes in a 25% deionized formamide, 2×SSC solution, cells were treated with fluorescein-conjugated anti-digoxin antibody (Roche, 11207741910; dilution 1:100) at 4°C overnight, followed by counterstaining with DAPI for nuclear visualization.

#### RNA ISH in kidney tissue

RNA ISH assay in renal cortex specimens was performed as previously described [12,36,65], paraffin-embedded kidney tissues were first digested with trypsin and fixed in 4% paraformaldehyde. Sections were hybridized with digoxin-labeled LNA-modified *circAASS* probe (Sangon Biotech, Shanghai) at 52°C for 16 h. Following washing, sections were incubated overnight at 4°C with an anti-digoxigenin monoclonal antibody (Abcam, ab419; dilution 1:200) to facilitate detection. Subsequently, sections were treated with alkaline phosphatase streptavidin (Beyotime, A0312) followed by chromogenic development using nitro blue tetrazolium-5-bromo-4-chloro-3-indolylphosphate solution (Beyotime, C3206). For quantitative analysis of *circAASS* by ISH, we evaluated at least 10 random visual fields per sample and computed the score using the formula: 3×high% + 2×positive% + 1×low%, with results expressed as mean values.

### Immunofluorescence

Cells were seeded on a confocal dish, fixed with 4% paraformaldehyde, permeabilized with 0.3% Triton X-100, sealed with 5% BSA (Sigma-Aldrich, V900933) for 2 h, and incubated overnight with the appropriate primary antibody. Then, the cells were incubated with allogeneic secondary antibody (Thermo Fisher Scientific, A-11008) in the dark for 1 h. Finally, nuclei were stained with DAPI, and images were collected by confocal microscopy (Olympus, FV3000).

### Mitophagic flux analysis

HK2 cells were infected with mitochondria-targeted Keima (mKeima) adenovirus (customized by GeneChem) for 6 h. 48 h after mKeima transfection, mKeima fluorescence density was observed using a Olympus confocal microscope. The mKeima protein can be used to detect lysosomal mitochondrial degradation as an indicator to evaluate mitophagic flux. The sensitivity of mKeima to pH can be examined whether the mitochondria is in an acidic lysosome (excitation: 561 nm, red) or neutral compartments (excitation: 488 nm, green). Pairs of images for ratiometric analysis of mKeima fluorescence were sequentially collected in 488 nm (green) and 561 nm (red) excitation. The nuclei were labeled with Hoechst 33342. Fluorescence density of mKeima was calculated as the ratio of e × 561 nm/488 nm fluorescence integrated density in each 630× fields using ImageJ.

### RNA affinity-isolation

RNA affinity-isolation assays were conducted using a Magnetic RNA-Protein Pull-Down Kit (Thermo Fisher Scientific, 20164) following established protocols. Initially, 2 × 10^7^ HK2 cells were cross-linked with 1% formaldehyde (Sigma-Aldrich, 47608) for 30 min at room temperature. After fixation, the cells were thoroughly washed with ice-cold PBS and lysed in 500 μL of IP lysis buffer (Thermo Fisher Scientific, 87787), supplemented with phosphatase and protease inhibitors, as well as an RNase inhibitor (Invitrogen, N8080119). The cell lysates were incubated with 100 nM biotin-labeled probes targeting endogenous *circAASS* or control probes synthesized by Sangon Biotech. This incubation occurred at 37°C overnight on a rotator. Subsequently, Nucleic-Acid Compatible Streptavidin Magnetic Beads (Thermo Fisher Scientific, 20164) were added to the RNA-protein complexes and incubated at 37°C for 2 h to facilitate binding.

The beads were then washed extensively with cold IP lysis buffer to remove unbound materials. Co-precipitated proteins were analyzed either by mass spectrometry or western blotting to identify RNA-binding proteins. The sequences of the probes utilized in this study are detailed in Table S5.

### The construction of Flag-tagged PPARGC1A truncated mutations

The construction of Flag-tagged PPARGC1A truncated mutations was performed through a series of molecular cloning techniques. Initially, the full-length *PPARGC1A* cDNA was amplified from a human cDNA library using high-fidelity PCR. Specific primers were designed to include restriction sites for cloning into the pCMV-Tag2B vector (NovoPro Bioscience, V011240), which contains a Flag-tag for protein detection.

Truncated mutations were generated using site-directed mutagenesis with the QuikChange II XL kit (Agilent, 200522), introducing stop codons at predetermined positions within the *PPARGC1A* sequence. Each mutation was confirmed by Sanger sequencing to ensure accuracy. The Flag-tagged constructs were assembled using Gibson Assembly (Thermo Fisher Scientific, A46627), allowing for seamless insertion into the vector. Following transformation into competent *E. coli*, positive clones were screened *via* colony PCR and verified by restriction enzyme analysis. The constructs were then transfected into HEK293T cells using Lipofectamine 3000. The expression of the Flag-tagged truncated proteins was assessed by western blotting with anti-Flag antibodies (Sigma-Aldrich, F9291), confirming the successful expression and integrity of the truncated variants.

### RNA-protein immunoprecipitation (RIP) assay

RIP assays were performed using Magna RIP™ RNA-Binding Protein Immunoprecipitation Kit (Millipore, 71–700) following the manufacturer’s instructions. Briefly, cells were cross-linked with 1% formaldehyde for 30 min at room temperature and then incubated with RIP lysis buffer containing RNase inhibitor and protease inhibitor. The RNAs in the lysates were fragmented by sonication. Magnetic beads were pre-incubated with the anti-AGO2 antibody (Abcam, ab186733), anti-IGF2BP2 (Novus Biologicals, NBP3-30626) or normal rabbit IgG (Abcam, ab125938) for 1 h at room temperature, and lysates were immunoprecipitated with beads at 4°C overnight. The immunoprecipitated RNA complex were then purified and detected by qPCR.

### Co-immunoprecipitation (co-IP) assay

The proteins extracted from HK2 cells were incubated with anti-PGC-1α at 4°C on a rotator overnight. In order to capture antibody-protein complexes, the protein A/G beads (Thermo Fisher Scientific, 88803) was used to incubate with the lysates for 2 h. The complexes were then detected and analyzed by western blot analysis.

### Mass spectrometry analysis

Protein samples were separated by SDS-PAGE, then the SDS-PAGE gels were visualized by silver staining using Pierce Sliver Stain Kit (Thermo Fisher Scientific, 24600) according to the instructions. The target gel bands were cut, then LC-MS was performed by Shanghai Bioprofile Technology Co., Ltd. as described previously [12].

### mRNA sequencing and functional enrichment analysis

To investigate the functional enrichment of differentially expressed mRNAs between *circAASS*-OE and wild-type cells, total RNA was extracted using the TRIzol reagent and assessed for quality with an Agilent Bioanalyzer. RNA-sequencing libraries were constructed using the KAPA Stranded RNA-Sequencing Library Prep Kit (KAPA Biosystems, KK8401) and sequenced on the Illumina NovaSeq platform.

Raw sequencing reads were processed with FastQC to ensure quality control, followed by trimming of low-quality sequences with Trimmomatic. Clean reads were aligned to the human reference genome GRCh37 utilizing HISAT2, and gene expression levels were quantified using the RSEM software package. Differential expression analysis was performed using the edgeR package, with a focus on mRNAs exhibiting a fold change ≥2 and a *p* value < 0.05 between *circAASS*-OE and wild-type cells. The identified differentially expressed mRNAs were subjected to Gene Ontology (GO) enrichment analysis using the Database for Annotation Visualization and Integrated Discovery (DAVID). Biological processes with a *p* value < 0.05 were considered significantly enriched, providing insights into the functional implications of the observed gene expression changes.

### Bioinformatics analysis

The target miRNAs of *circAASS* were predicted by circular RNA interactome (https://circinteractome.nia.nih.gov). Target genes were predicted by TargetScan (https://www.targetscan.org/), miRbase (https://www.mirbase.org/), and miRDB (https://www.mirdb.org/).

### In situ injection of AAV9-Cdh16/Ksp-cadherin-*circAass* into C57BL/6 J mice

In order to induce *circAass* overexpression in mice, we utilized AAV9 vectors to introduce *circAass* into the kidney with *in* *situ* injection. Specifically, we injected AAV9 vectors carrying *circAass* or an empty control, both under the control of the proximal tubule-specific *Cdh16/Ksp-cadherin* promoter (Shanghai Genechem), via a single tail vein injection (200 μL of 1 × 10^12^ vg/mL in saline). As previously described [66], 1,341 bp of the 5’ flanking region (nucleotides 2,430–770 of GenBank accession no. AF118228) of *Cdh16/Ksp-cadherin* (a unique, tissue-specific member of the cadherin family that is exclusively expressed in TECs) was used as the upstream promoter of *circAass*. Throughout the study, no evidence of acute toxicity, including weight loss, behavioral abnormalities, or impaired liver function, was observed in the mice.

### Statistical analysis

Statistical analysis was performed using GraphPad Prism 7.0 and SPSS 22.0 software. Mean ± sd was used to express continuous variables. Student’s *t*-test was used to analyze the differences of data between two groups. ANOVA was used to analyze the differences between multiple groups. Pearson’s correlation test was performed to assess the associations between *circAASS* expression levels and each of the following parameters: PINK1, PPARGC1A/PGC-1α, acute tubular injury score, and tubular interstitial fibrosis index. All experiments were independently performed in triplicate. Statistical significance was defined as *p* < 0.05.

## Supplementary Material

Supplementary Data 20251014.docx

## Data Availability

All data associated with this study are available in the main text or the Supplemental materials. To identify the dysregulated circRNAs, GEO datasets (GSE286507 and GSE220782) and BioProject ID PRJNA806364 were used. To analyze the common dysregulated mRNAs in kidney tissues from three models of AKI, GEO datasets (GSE286507 and GSE220812) and BioProject ID PRJNA806364 were used.
