# Supplementary material for: CircAASS alleviates renal injury and fibrosis by regulating mitochondrial homeostasis in tubular epithelial cells
Source: Autophagy. 2025 Nov 20;22(1):182–206. doi: 10.1080/15548627.2025.2581212 (PMC12758326; doi:10.1080/15548627.2025.2581212)
Supplement: Supplementary Data 20251014.docx [file KAUP_A_2581212_SM7025.docx]

***CircAASS* alleviates renal injury and fibrosis by regulating mitochondrial homeostasis in tubular epithelial cells**

Tongtong Ma^a^, Yanmei Yu^b^, Huasheng Luo^b^, Ziqi Zhang^b^, Miaotao Wei^b^, Chunjie Tian^c^, Xianmou Fan^d^, Zhenyi Yan^a^, Shaowu Zhang^b^, Junfeng Hao^b^ and Peng Wang^b^

^a^Department of Anesthesiology, Affiliated Hospital of Guangdong Medical University, Zhanjiang 524000, Guangdong, China.

^b^Guangdong Provincial Key Laboratory of Autophagy and Major Chronic Non-communicable Diseases, Key Laboratory of Prevention and Management of Chronic Kidney Diseases of Zhanjiang City, Institute of Nephrology, Affiliated Hospital of Guangdong Medical University, Zhanjiang 524000, Guangdong, China.

^c^Department of Otorhinolaryngology, Affiliated Hospital of Guangdong Medical University, Zhanjiang 524000, Guangdong, China.

^d^Department of Plastic Surgery, Affiliated Hospital of Guangdong Medical University, Zhanjiang 524000, Guangdong, China.

**Contact** Peng Wang [wangpeng@gdmu.edu.cn](mailto:wangpeng@gdmu.edu.cn) Guangdong Provincial Key Laboratory of Autophagy and Major Chronic Non-communicable Diseases, Key Laboratory of Prevention and Management of Chronic Kidney Diseases of Zhanjiang City, Institute of Nephrology, Affiliated Hospital of Guangdong Medical University, No. 57, South Renmin Avenue, Xiashan District, Zhanjiang, Guangdong 524023, China


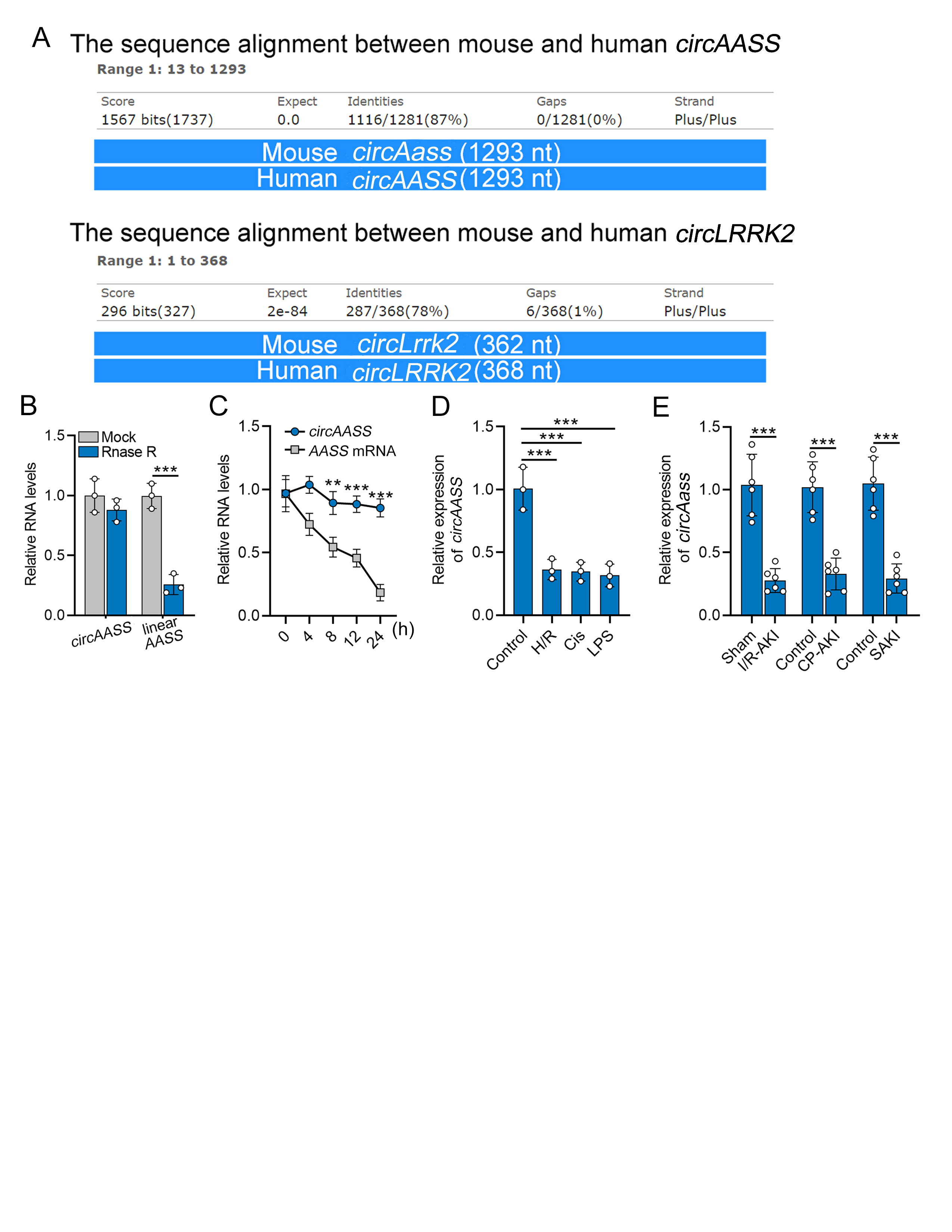


**Figure** **S1.** *CircAASS*, a highly conserved circular RNA between humans and mice, is downregulated in injured renal tubular epithelial cells. (**A**) Sequence alignment analysis demonstrates that *circAASS/circAass* exhibits higher human-mouse homology (87%) than *circLRRK2/circLrrk2* (78%). (**B**) The relative RNA levels of *circAASS* and linear *AASS* in HK2 with RNase R treatment. (**C**) The relative RNA levels of *circAASS* and *AASS* mRNA in HK2 with actinomycin D treatment for the indicated time points. (**D**) qRT-PCR analysis showing the expression of *circAASS* in injured TECs, triggered by H/R, cisplatin treatment and LPS treatment. (**E**) qRT-PCR analysis showing the expression of *circAass* in the renal cortex of AKI mice. Data are presented as the mean ± SD. ****p*<0.001, by 2-tailed Student’s *t* test (**B** and **E**). ***p*<0.01, ****p*<0.001, by 2-way ANOVA with Tukey’s multiple-comparison test (**C**). ****p*<0.001, by 1-way ANOVA with Tukey’s multiple-comparison test (**D**).


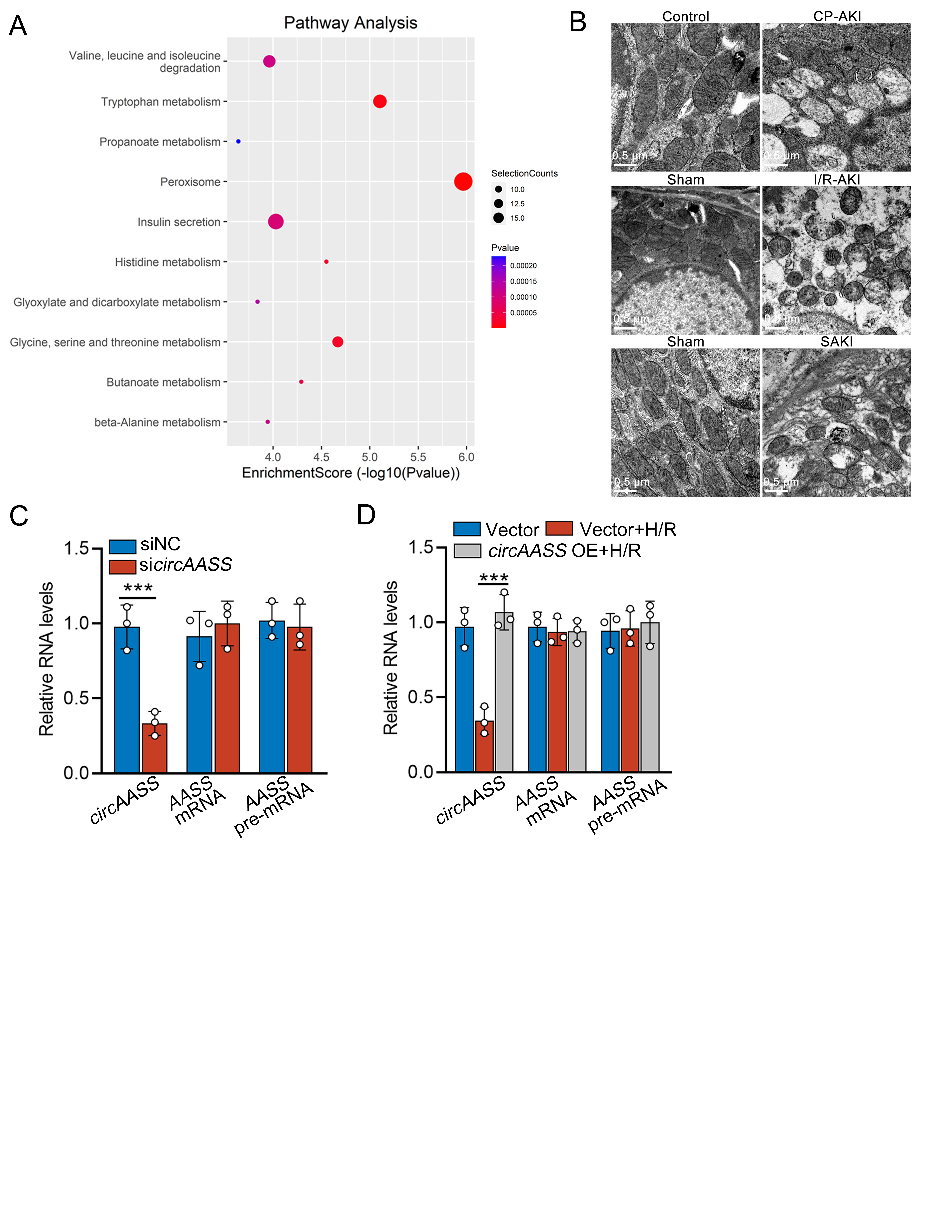


**Figure S2.** Mitochondrial damage in renal TECs is a common feature across multiple AKI models, including I/R-AKI, cisplatin-AKI, and septic AKI. (**A**) KEGG pathway analysis of the common dysregulated mRNAs in kidney tissues from I/R-AKI, cisplatin-induced AKI and SAKI mice, three datasets were analyzed (GSE286507, BioProject ID PRJNA806364 and GSE220782). (**B**) Representative TEM images of mitochondria damage in the TECs from different models of AKI mice. (**C**) qRT-PCR analysis to verify siRNA efficiency of the *circAASS* knockdown. (**D**) qRT-PCR analysis to verify overexpression efficiency of *circAASS* overexpression in HK2 cells treated with H/R. Data are presented as the mean ± SD. ****p*<0.001, by 2-tailed Student’s *t* test (**C**). ****p*<0.001, by 1-way ANOVA with Tukey’s multiple-comparison test (**D**). Scale bar: 500 nm (B).

**
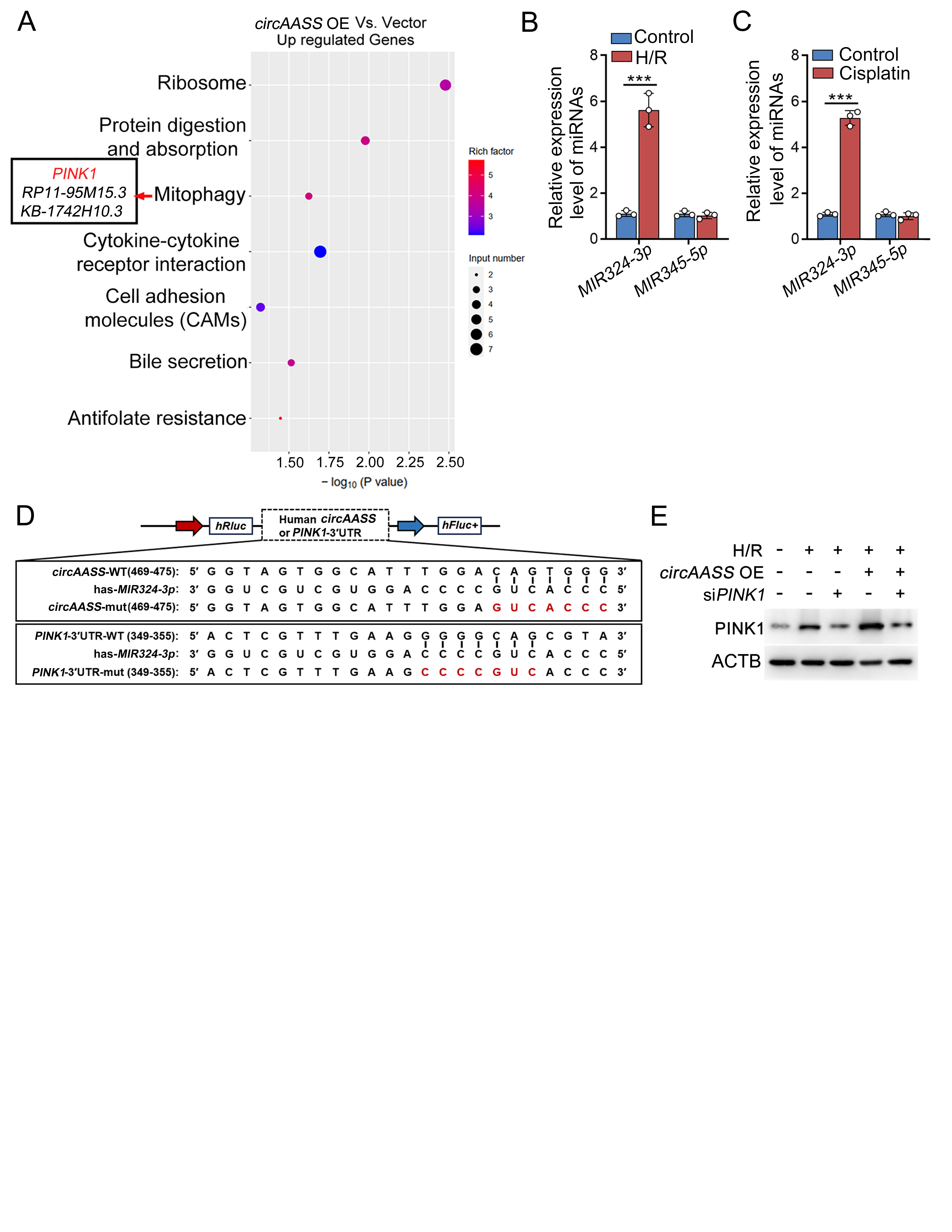
**

**Figure S3.** *CircAASS* regulates mitophagy in TECs via *MIR324*-*3p*/*PINK1* axis. (**A**) KEGG pathway analysis of the genes in HK2 overexpressed with *circAASS*, as compared to HK2 transfected with empty vector. (**B** and **C**) qRT-PCR assay showing the expression of *MIR324-3p* and *MIR345-5p* in H/R- or cisplatin- treated HK2 cells. (**D**) Schematic diagram of human *circAASS* or *PINK1* 3’UTR luciferase reporter gene construction. (**E**) Western blot analysis demonstrates that *circAASS* overexpression upregulates PINK1 expression in H/R-treated TECs, while *PINK1*-targeting siRNA effectively suppresses its protein expression. Data are presented as the mean ± SD. ****p*<0.001, by 2-tailed Student’s *t* test (**B** and **C**).

**
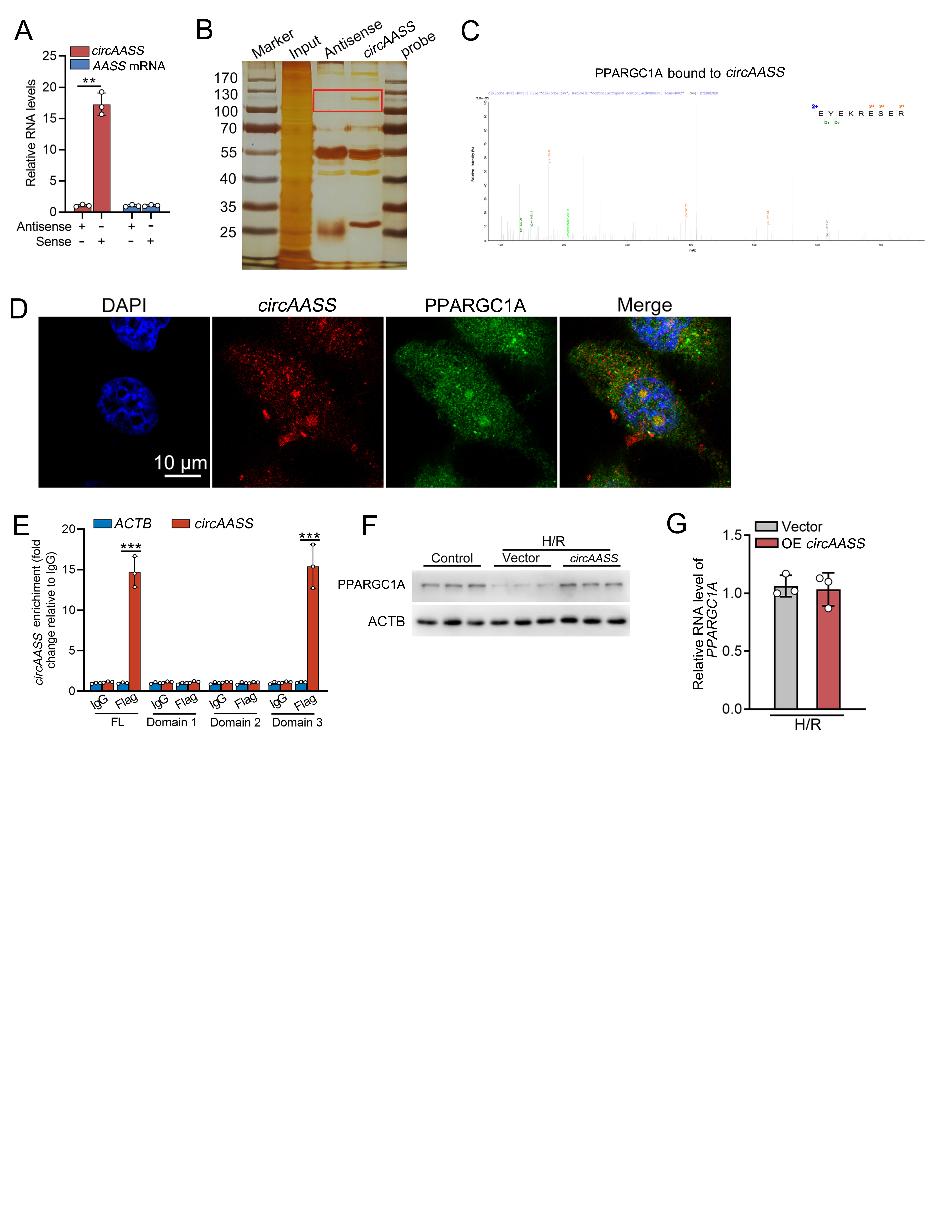
**

**Figure S4.** Nuclear *circAASS* directly interacts with PPARGC1A/PGC-1α. (**A**) qRT-PCR assay after RNA affinity isolation with biotinylated Sense probe or Antisense probe showing the enrichment of *circAASS* by using Sense probe. (**B**) Biotinylated sense or antisense probe was incubated with HK2 total cell lysates for RNA affinity isolation assays. The sense-specific bands were excised and analyzed using mass spectrometry. (**C**) Mass spectrometry identified PPARGC1A/PGC-1α, which was pulled down from HK2 lysates by biotin-labeled oligo probes for *circAASS*. (**D**) Immunofluorescence detected the colocalization of *circAASS* (red) and PPARGC1A/PGC-1α (green) in HK2. Nuclei were stained with DAPI. Scale bar: 10 μm. (**E**) Flag-PGC-1α interacts with *circAASS* through its full-length form and domain 3, as shown by RIP. (**F**) Western blotting showing PPARGC1A/PGC-1α expression in H/R-treated HK2 cells transfected with *circAASS* plasmids or empty vector. (**G**) qRT-PCR assay reveals that overexpressing *circAASS* had no effect on the expression of *PPARGC1A* mRNA. Data are presented as the mean ± SD. ***p*<0.01, ****p*<0.001, by 2-tailed Student’s *t* test (**A**, **E** and **G**). Scale bar: 10 μm (D).

**
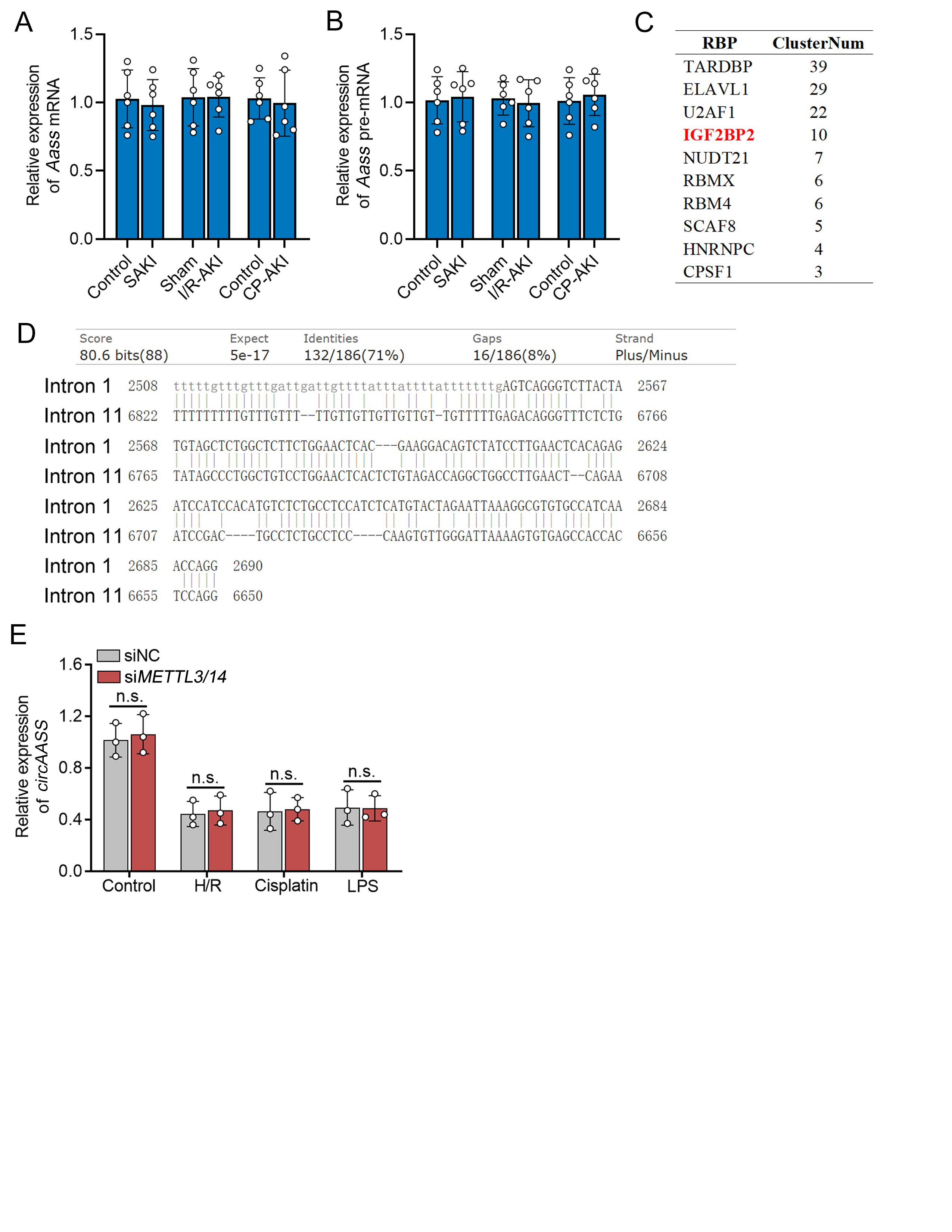
**

**Figure S5.** IGF2BP2 inhibits the biogenesis of *circAASS.* (**A** and **B**) qRT-PCR showing the relative expression of mature *Aass* mRNA and its precursor in I/R-AKI, CP-AKI, and SAKI mouse models. (**C**) RNA-Binding proteins exhibiting binding affinity to *AASS* pre-mRNA identified *via* ENCORI Platform Screening. (**D**) The sequence alignment between intron 1 and intron 11 of the *Aass* gene. (**E**) qRT-PCR analysis demonstrates that dual knockdown of *METTL3* and *METTL14* does not alter *circAASS* expression levels in HK2 cells under either basal conditions or following H/R injury, cisplatin treatment, or LPS stimulation. Data are presented as the mean ± SD. n.s. not significant, by 2-tailed Student’s *t* test (**A**, **B** and **E**).

**
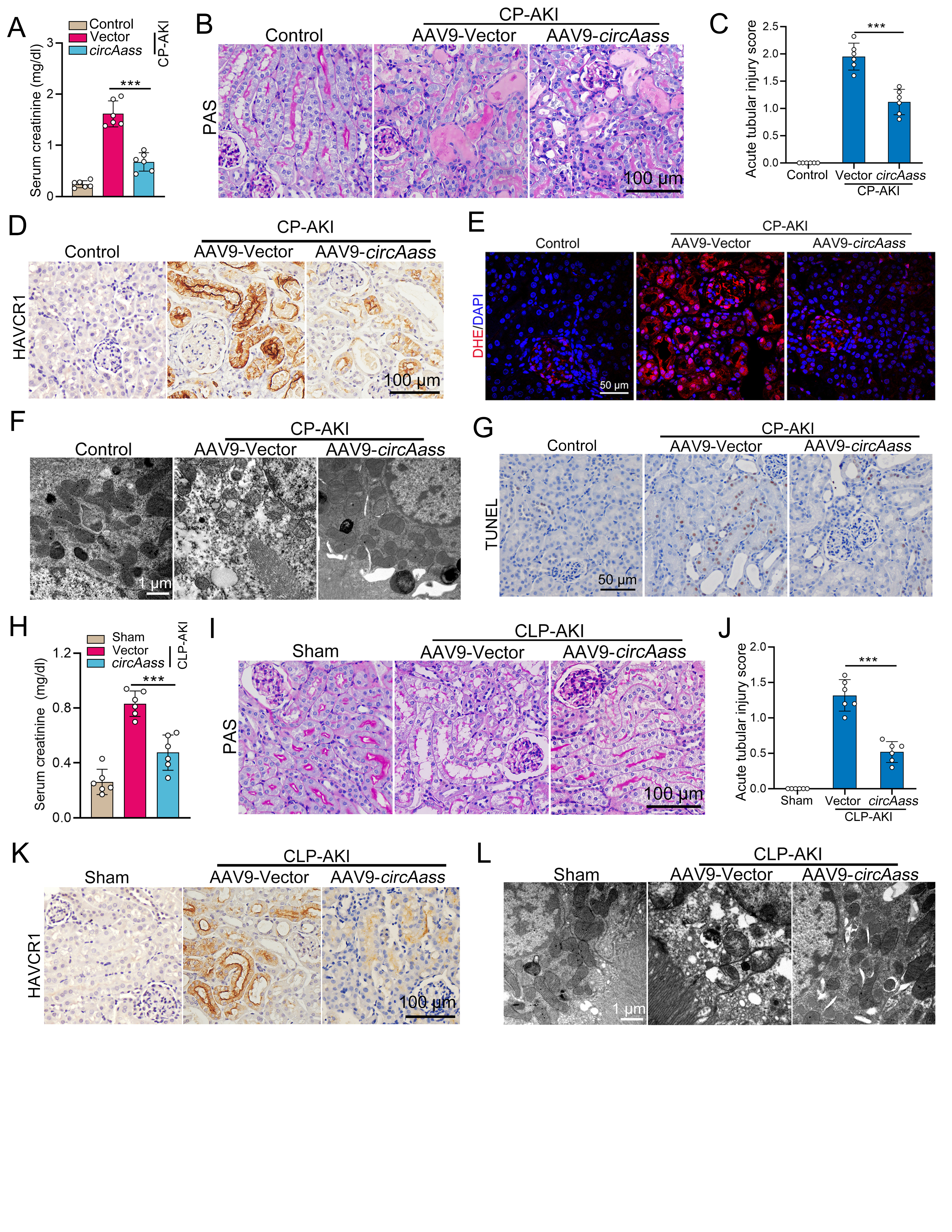
**

**Figure S6.** C*ircAass* alleviates AKI induced by cisplatin or sepsis. (**A**) Serum creatinine level in cisplatin (CP)-AKI mice injected with either AAV9-Vector or AAV9-*circAass* 3 days post CP treatment. (**B**&**C**) Representative images and quantification data of PAS staining kidney tissue from mice treated with AAV9-*circAass* 3 days post CP treatment. (**D**) Representative images and quantification data of IHC staining of HAVCR1/Kim-1 in kidney tissue from mice treated with AAV9-*circAass*, at 3 days post CP-AKI. (**E**) Representative images of DHE staining. DHE nuclear staining indicates the presence of ROS. (**F**) Representative TEM images of mitochondria in the TECs from CP-AKI mice. (**G**) Representative images of TUNEL staining of TEC apoptosis of CP-AKI mice injected with AAV9-*circAass*. (**H**) Serum creatinine level in CLP-AKI mice injected with either AAV9-Vector or AAV9-*circAass* 1 day post CLP treatment. (**I** and **J**) Representative images and quantification data of PAS staining kidney tissue from mice treated with AAV9-*circAass* 1 day post CLP treatment. (**K**) Representative images of IHC staining of HAVCR1/Kim-1 in kidney tissue from mice treated with AAV9-*circAass*, at 1 day post CLP-AKI. (**L**) Representative TEM images of mitochondria in the TECs from CLP-AKI mice. Data are presented as the mean ± SD. ****p*<0.001, by 1-way ANOVA with Tukey’s multiple-comparison test (**A**, **C**, **H** and **J**). Scale bars: 100 μm (B, D, I and K), 50 μm (E and G) and 1 μm (F and L).


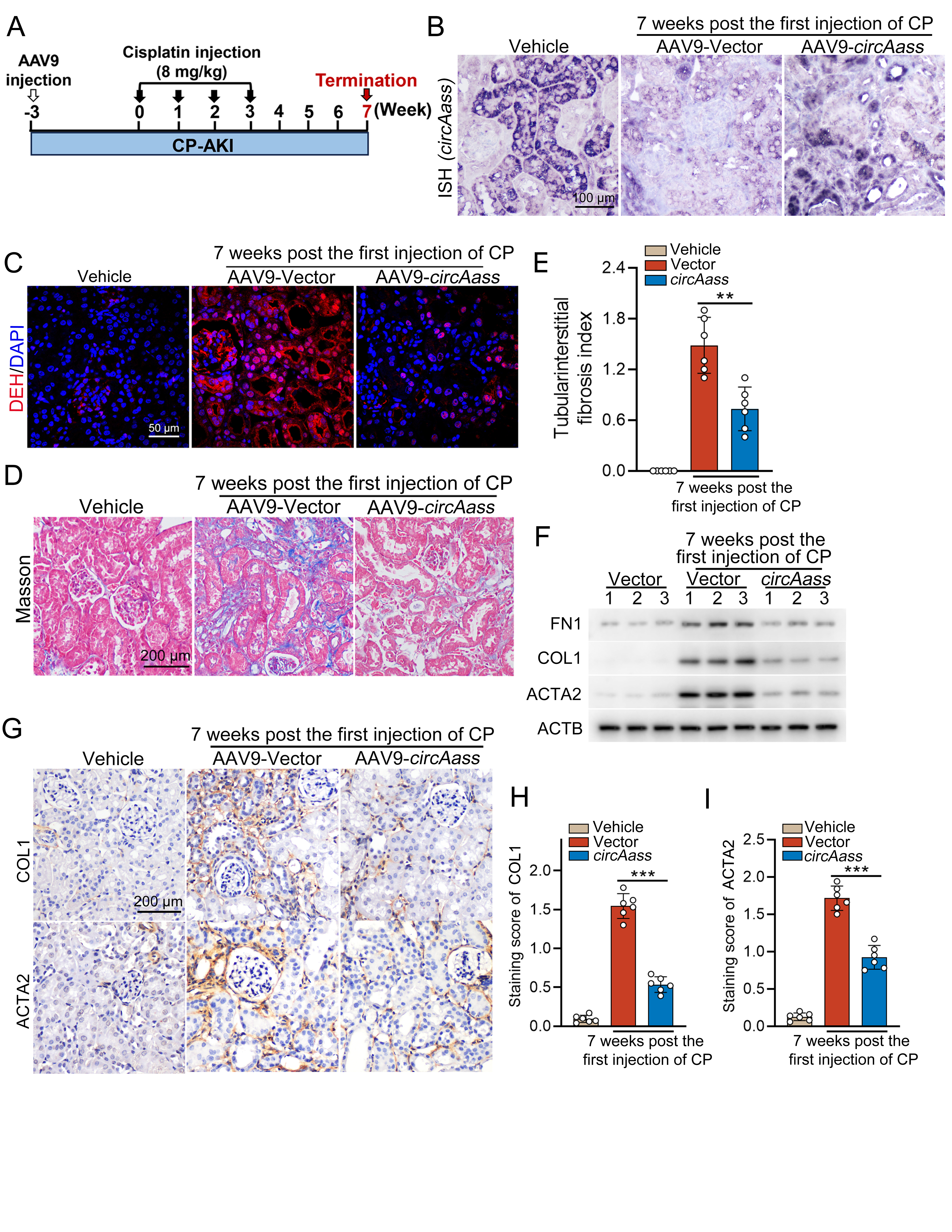


**Figure S7.** Ectopic expression of *circAass* inhibits tubulointerstitial fibrosis post cisplatin-induced AKI. (**A**) Diagram shows the experimental design. (**B**) Representative images of ISH of *circAass* expression in kidneys from mice treated with AAV9-Vector or AAV9-*circAass* 7 weeks post-CP treatment. (**C**) Representative images of dihydroethidium staining. DHE staining showed that overexpression of *circAass* decreased the levels of ROS 7 weeks post the first injection of cisplatin. (**D** and **E**) Representative images and quantification data of Masson’s trichrome staining in CP mice injected with either AAV9-Vector or AAV9-*circAass* 7 weeks post the first injection of cisplatin. (**F**) Western blot showed that overexpression of *circAass* decreased the expression of profibrotic factors. (**G**-**I**) Representative images and quantification data of IHC staining of COLI and ACTA2/αSMA in CP-AKI mice injected with either AAV9-Vector or AAV9-*circAass* 7 weeks post the first injection of cisplatin. Data are presented as the mean ± SD. ***p*<0.01, ****p*<0.001, by 1-way ANOVA with Tukey’s multiple-comparison test (**E**, **H** and **I**). Scale bars: 100 μm (B), 50 μm (C), 200 μm (D and G).

**Table S1.** Differentially expressed circRNAs of three AKI mice models.

| **I/R-AKI** | | **CP-AKI** | | **SAKI** | |
| --- | --- | --- | --- | --- | --- |
| **CircRNA-ID** | **Regulation** | **CircRNA-ID** | **Regulation** | **CircRNA-ID** | **Regulation** |
| chr5:111346483-111349574+ | Up | circRNA_0270\|Chr1:157286731_157299264_- | Up | mmu_circRNA_19427 | Up |
| chr17:80653862-80655921- | Up | circRNA_0278\|Chr1:162285997_162313696_- | Up | mmu_circRNA_41361 | Up |
| chr4:126814256-126816474+ | Up | circRNA_0308\|Chr1:179784090_179786131_- | Up | mmu_circRNA_36330 | Up |
| chr5:73510886-73522744+ | Up | circRNA_0340\|Chr10:7152864_7154485_- | Up | mmu_circRNA_42341 | Up |
| chr3:35809357-35820112+ | Up | circRNA_0388\|Chr10:42196466_42197901_- | Up | mmu_circRNA_36265 | Up |
| chr16:16474227-16504216- | Up | circRNA_0421\|Chr10:67157907_67189849_+ | Up | mmu_circRNA_30665 | Up |
| chr2:78913674-78920209+ | Up | circRNA_0475\|Chr10:103137107_103146293_- | Up | mmu_circRNA_43861 | Up |
| chr1:140362949-140383095+ | Up | circRNA_0500\|Chr10:117701565_117705229_- | Up | mmu_circRNA_44192 | Up |
| chr1:139085883-139110485+ | Up | circRNA_0595\|Chr11:26534211_26550075_- | Up | mmu_circRNA_31626 | Up |
| chr6:108376762-108418000+ | Up | circRNA_0599\|Chr11:29705535_29708770_+ | Up | mmu_circRNA_31169 | Up |
| chr13:64243634-64248466- | Up | circRNA_0601\|Chr11:30219579_30219772_- | Up | mmu_circRNA_19314 | Up |
| chr13:119437969-119470161+ | Up | circRNA_0746\|Chr11:96322111_96323445_+ | Up | mmu_circRNA_38232 | Up |
| chr1:20209161-20288261- | Up | circRNA_0765\|Chr11:104423922_104426468_- | Up | mmu_circRNA_38234 | Up |
| chr4:121170618-121190258- | Up | circRNA_1004\|Chr12:104393733_104412484_+ | Up | mmu_circRNA_24044 | Up |
| chr13:32127000-32128099- | Up | circRNA_1065\|Chr13:25102333_25119406_+ | Up | mmu_circRNA_26188 | Up |
| chr19:5801232-5801822- | Up | circRNA_1383\|Chr14:105287617_105294892_+ | Up | mmu_circRNA_29102 | Up |
| chr16:94419398-94424187+ | Up | circRNA_1388\|Chr14:118654150_118669107_- | Up | mmu_circRNA_19469 | Up |
| chr11:30219579-30219772- | Up | circRNA_1391\|Chr14:120324794_120325412_+ | Up | mmu_circRNA_42044 | Up |
| chr11:74679458-74699520- | Up | circRNA_1478\|Chr15:62107148_62107465_+ | Up | mmu_circRNA_42255 | Up |
| chr5:17811181-17829549- | Up | circRNA_1599\|Chr16:17015898_17018475_+ | Up | mmu_circRNA_43178 | Up |
| chr2:156745835-156749356+ | Up | circRNA_1684\|Chr16:93847288_93853217_+ | Up | mmu_circRNA_21478 | Up |
| chr14:27475695-27480091+ | Up | circRNA_1766\|Chr17:32544883_32629156_+ | Up | mmu_circRNA_33862 | Up |
| chr12:21394965-21398364- | Up | circRNA_1821\|Chr17:63471408_63490887_- | Up | mmu_circRNA_38013 | Up |
| chr10:30689704-30704680- | Up | circRNA_1830\|Chr17:65592291_65595054_- | Up | mmu_circRNA_006355 | Up |
| chr2:126810163-126813408- | Up | circRNA_1891\|Chr17:88462713_88463263_+ | Up | mmu_circRNA_30395 | Up |
| chr5:103885907-103886290- | Up | circRNA_2140\|Chr19:28665753_28666236_- | Up | mmu_circRNA_41496 | Up |
| chr2:147024715-147026439+ | Up | circRNA_2154\|Chr19:34587061_34607998_+ | Up | mmu_circRNA_36266 | Up |
| chr2:156264281-156276668+ | Up | circRNA_2635\|Chr3:27620605_27643089_- | Up | mmu_circRNA_44123 | Up |
| chr16:55448590-55454066- | Up | circRNA_2755\|Chr3:103310309_103311513_+ | Up | mmu_circRNA_19034 | Up |
| chr17:42845326-42852528- | Up | circRNA_2820\|Chr3:135655500_135669339_- | Up | mmu_circRNA_25474 | Up |
| chr9:123593831-123594669- | Down | circRNA_3102\|Chr4:143131710_143142234_- | Up | mmu_circRNA_013351 | Up |
| chrX:85754332-85761407- | Down | circRNA_3528\|Chr6:40724571_40750981_+ | Up | mmu_circRNA_19130 | Down |
| chr9:20473028-20476478- | Down | circRNA_3699\|Chr6:140633689_140648085_+ | Up | mmu_circRNA_34880 | Down |
| chr5:4081184-4081349- | Down | circRNA_3873\|Chr7:104315301_104348403_- | Up | mmu_circRNA_000610 | Down |
| chr12:4961218-4978588+ | Down | circRNA_3907\|Chr7:121052087_121057399_+ | Up | mmu_circRNA_016702 | Down |
| chr15:91705106-91712862+ | Down | circRNA_4002\|Chr8:31946984_31968185_- | Up | mmu_circRNA_43557 | Down |
| chr13:107769560-107788009- | Down | circRNA_4010\|Chr8:33834304_33866404_- | Up | mmu_circRNA_43940 | Down |
| chr11:62419598-62438515- | Down | circRNA_4011\|Chr8:33862986_33866404_- | Up | mmu_circRNA_39531 | Down |
| chr8:77409548-77413627- | Down | circRNA_4157\|Chr8:111715283_111715776_- | Up | mmu_circRNA_22903 | Down |
| chr1:16463035-16509408- | Down | circRNA_4231\|Chr9:40413297_40456165_- | Up | mmu_circRNA_19001 | Down |
| chr6:142086453-142103142- | Down | circRNA_4347\|Chr9:77775079_77779635_+ | Up | mmu_circRNA_35900 | Down |
| chr2:18101458-18126229+ | Down | circRNA_4469\|Chr9:123632110_123664693_- | Up | mmu_circRNA_19196 | Down |
| chr4:150500003-150510038+ | Down | circRNA_4574\|ChrY:90739142_90793417_+ | Up | mmu_circRNA_011137 | Down |
| chr8:25640577-25649703+ | Down | circRNA_0153\|Chr1:82455723_82456895_- | Down | mmu_circRNA_011852 | Down |
| chr3:146463185-146493843- | Down | circRNA_0161\|Chr1:84912196_84929564_- | Down | mmu_circRNA_014628 | Down |
| chr14:57444355-57450741+ | Down | circRNA_0466\|Chr10:89473752_89483524_- | Down | mmu_circRNA_19495 | Down |
| chr16:31977680-31990465- | Down | circRNA_0494\|Chr10:116953785_116964118_- | Down | mmu_circRNA_18953 | Down |
| chr2:156096826-156103429- | Down | circRNA_0523\|Chr10:128084304_128084452_+ | Down | mmu_circRNA_009056 | Down |
| chr2:166834471-166836457+ | Down | circRNA_0933\|Chr12:77323845_77332329_+ | Down | mmu_circRNA_38020 | Down |
| chr3:95784214-95787384- | Down | circRNA_0948\|Chr12:83990494_84009966_+ | Down | mmu_circRNA_003983 | Down |
| chr16:17348715-17356216- | Down | circRNA_1056\|Chr13:17644781_17672602_- | Down | mmu_circRNA_33758 | Down |
| chr12:77323845-77365359+ | Down | circRNA_1238\|Chr14:21626719_21664748_+ | Down | mmu_circRNA_017443 | Down |
| chr13:54523970-54529223+ | Down | circRNA_1306\|Chr14:57303593_57313129_- | Down | mmu_circRNA_41806 | Down |
| chr6:22132229-22145687+ | Down | circRNA_1345\|Chr14:75770171_75771533_- | Down | mmu_circRNA_26829 | Down |
| chr7:90160448-90165616+ | Down | circRNA_1421\|Chr15:9306075_9351214_+ | Down | mmu_circRNA_33495 | Down |
| chrX:144346682-144349419+ | Down | circRNA_1422\|Chr15:9311749_9365377_+ | Down | mmu_circRNA_19679 | Down |
| chr5:124391922-124398509- | Down | circRNA_1424\|Chr15:9321419_9367467_+ | Down | mmu_circRNA_28880 | Down |
| chr19:57378549-57379175+ | Down | circRNA_1468\|Chr15:50822105_50846942_- | Down | mmu_circRNA_000155 | Down |
| chr7:144179755-144192530+ | Down | circRNA_1537\|Chr15:91705106_91712862_+ | Down | mmu_circRNA_015633 | Down |
| chr5:100475689-100480321- | Down | circRNA_2103\|Chr19:12650257_12714633_+ | Down | mmu_circRNA_009396 | Down |
| chr9:54378714-54382721- | Down | circRNA_2118\|Chr19:23063340_23064443_- | Down | mmu_circRNA_34688 | Down |
| chr6:65869181-65901859+ | Down | circRNA_2240\|Chr2:5923869_5932542_- | Down | mmu_circRNA_012180 | Down |
| chr6:142145638-142161112- | Down | circRNA_2299\|Chr2:31183580_31188813_+ | Down | mmu_circRNA_29236 | Down |
| chr3:129735802-129740041- | Down | circRNA_2378\|Chr2:69533453_69538061_- | Down | mmu_circRNA_28135 | Down |
| chr1:20198018-20288261- | Down | circRNA_2408\|Chr2:76584461_76586251_+ | Down | mmu_circRNA_27501 | Down |
| chr1:53256629-53282092- | Down | circRNA_2439\|Chr2:105297726_105297968_+ | Down | mmu_circRNA_36141 | Down |
| chr3:51308050-51326035- | Down | circRNA_2493\|Chr2:126567747_126581003_+ | Down | mmu_circRNA_39001 | Down |
| chr7:99935162-99955540- | Down | circRNA_2750\|Chr3:96850228_96851740_+ | Down | mmu_circRNA_27503 | Down |
| chr8:79118175-79136663+ | Down | circRNA_2781\|Chr3:121228310_121235764_- | Down | mmu_circRNA_45581 | Down |
| chr18:79086551-79087180- | Down | circRNA_2783\|Chr3:121765001_121772366_- | Down | mmu_circRNA_19195 | Down |
| chr13:97929411-97936861- | Down | circRNA_2799\|Chr3:129735802_129740041_- | Down | mmu_circRNA_001848 | Down |
| chr8:41076232-41084840- | Down | circRNA_2804\|Chr3:131599865_131607463_+ | Down | mmu_circRNA_003780 | Down |
| chr5:106618071-106638635- | Down | circRNA_2812\|Chr3:132904658_132917553_- | Down | mmu_circRNA_18967 | Down |
| chr6:23107140-23122737- | Down | circRNA_2899\|Chr4:49536800_49538282_+ | Down | mmu_circRNA_40807 | Down |
|  |  | circRNA_2947\|Chr4:84291693_84293662_- | Down | mmu_circRNA_41032 | Down |
|  |  | circRNA_2976\|Chr4:96641272_96641448_- | Down | mmu_circRNA_30953 | Down |
|  |  | circRNA_3023\|Chr4:115524248_115606772_+ | Down | mmu_circRNA_004868 | Down |
|  |  | circRNA_3026\|Chr4:115569709_115606772_+ | Down | mmu_circRNA_013422 | Down |
|  |  | circRNA_3145\|Chr5:3755594_3772787_- | Down | mmu_circRNA_19253 | Down |
|  |  | circRNA_3273\|Chr5:89084642_89135820_+ | Down | mmu_circRNA_35496 | Down |
|  |  | circRNA_3284\|Chr5:89197649_89199816_+ | Down | mmu_circRNA_27696 | Down |
|  |  | circRNA_3300\|Chr5:100805898_100812202_- | Down | mmu_circRNA_000353 | Down |
|  |  | circRNA_3336\|Chr5:109751595_109805077_- | Down | mmu_circRNA_002886 | Down |
|  |  | circRNA_3348\|Chr5:113175205_113193907_- | Down | mmu_circRNA_26715 | Down |
|  |  | circRNA_3467\|Chr6:23107140_23122737_- | Down | mmu_circRNA_29065 | Down |
|  |  | circRNA_3500\|Chr6:38067737_38083002_- | Down | mmu_circRNA_001407 | Down |
|  |  | circRNA_3527\|Chr6:40714975_40750266_+ | Down | mmu_circRNA_19155 | Down |
|  |  | circRNA_3589\|Chr6:84129471_84152409_+ | Down | mmu_circRNA_27357 | Down |
|  |  | circRNA_3667\|Chr6:124914408_124914686_- | Down | mmu_circRNA_28143 | Down |
|  |  | circRNA_3669\|Chr6:124914408_124914704_- | Down | mmu_circRNA_017696 | Down |
|  |  | circRNA_3706\|Chr6:142145638_142167388_- | Down | mmu_circRNA_007027 | Down |
|  |  | circRNA_3843\|Chr7:87360728_87376282_+ | Down | mmu_circRNA_017947 | Down |
|  |  | circRNA_4016\|Chr8:41082614_41084840_- | Down | mmu_circRNA_37007 | Down |
|  |  | circRNA_4053\|Chr8:77344640_77384796_- | Down | mmu_circRNA_44963 | Down |
|  |  | circRNA_4120\|Chr8:93271831_93328531_- | Down | mmu_circRNA_006097 | Down |
|  |  | circRNA_4228\|Chr9:32181842_32208265_+ | Down | mmu_circRNA_24050 | Down |
|  |  | circRNA_4241\|Chr9:44651337_44684725_+ | Down | mmu_circRNA_45583 | Down |
|  |  | circRNA_4340\|Chr9:73930597_73933809_- | Down | mmu_circRNA_19091 | Down |
|  |  | circRNA_4473\|ChrMT:5760_5954_- | Down | mmu_circRNA_43788 | Down |
|  |  | circRNA_4480\|ChrMT:13917_14141_- | Down | mmu_circRNA_20265 | Down |
|  |  | circRNA_4526\|ChrX:85746225_85761407_- | Down | mmu_circRNA_009519 | Down |
|  |  |  |  | mmu_circRNA_45580 | Down |
|  |  |  |  | mmu_circRNA_31616 | Down |
|  |  |  |  | mmu_circRNA_002139 | Down |
|  |  |  |  | mmu_circRNA_018017 | Down |
|  |  |  |  | mmu_circRNA_011975 | Down |
|  |  |  |  | mmu_circRNA_005048 | Down |
|  |  |  |  | mmu_circRNA_42217 | Down |
|  |  |  |  | mmu_circRNA_28137 | Down |
|  |  |  |  | mmu_circRNA_013774 | Down |
|  |  |  |  | mmu_circRNA_42567 | Down |
|  |  |  |  | mmu_circRNA_19201 | Down |
|  |  |  |  | mmu_circRNA_22786 | Down |
|  |  |  |  | mmu_circRNA_43976 | Down |
|  |  |  |  | mmu_circRNA_29499 | Down |
|  |  |  |  | mmu_circRNA_33079 | Down |
|  |  |  |  | mmu_circRNA_19353 | Down |
|  |  |  |  | mmu_circRNA_43975 | Down |
|  |  |  |  | mmu_circRNA_32011 | Down |
|  |  |  |  | mmu_circRNA_017081 | Down |
|  |  |  |  | mmu_circRNA_19252 | Down |
|  |  |  |  | mmu_circRNA_29185 | Down |
|  |  |  |  | mmu_circRNA_010385 | Down |
|  |  |  |  | mmu_circRNA_26276 | Down |
|  |  |  |  | mmu_circRNA_18949 | Down |
|  |  |  |  | mmu_circRNA_19194 | Down |
|  |  |  |  | mmu_circRNA_000453 | Down |
|  |  |  |  | mmu_circRNA_43596 | Down |
|  |  |  |  | mmu_circRNA_23788 | Down |
|  |  |  |  | mmu_circRNA_42260 | Down |
|  |  |  |  | mmu_circRNA_007066 | Down |
|  |  |  |  | mmu_circRNA_001009 | Down |
|  |  |  |  | mmu_circRNA_017446 | Down |
|  |  |  |  | mmu_circRNA_44846 | Down |
|  |  |  |  | mmu_circRNA_31400 | Down |
|  |  |  |  | mmu_circRNA_30340 | Down |
|  |  |  |  | mmu_circRNA_018626 | Down |
|  |  |  |  | mmu_circRNA_24811 | Down |
|  |  |  |  | mmu_circRNA_37175 | Down |
|  |  |  |  | mmu_circRNA_34341 | Down |
|  |  |  |  | mmu_circRNA_39902 | Down |
|  |  |  |  | mmu_circRNA_24813 | Down |
|  |  |  |  | mmu_circRNA_31221 | Down |
|  |  |  |  | mmu_circRNA_001096 | Down |
|  |  |  |  | mmu_circRNA_28239 | Down |
|  |  |  |  | mmu_circRNA_005949 | Down |
|  |  |  |  | mmu_circRNA_42446 | Down |
|  |  |  |  | mmu_circRNA_009521 | Down |
|  |  |  |  | mmu_circRNA_000787 | Down |
|  |  |  |  | mmu_circRNA_41204 | Down |
|  |  |  |  | mmu_circRNA_37793 | Down |
|  |  |  |  | mmu_circRNA_34464 | Down |
|  |  |  |  | mmu_circRNA_37186 | Down |
|  |  |  |  | mmu_circRNA_43789 | Down |
|  |  |  |  | mmu_circRNA_29238 | Down |
|  |  |  |  | mmu_circRNA_28595 | Down |
|  |  |  |  | mmu_circRNA_35040 | Down |
|  |  |  |  | mmu_circRNA_29237 | Down |
|  |  |  |  | mmu_circRNA_19164 | Down |
|  |  |  |  | mmu_circRNA_19510 | Down |
|  |  |  |  | mmu_circRNA_005202 | Down |
|  |  |  |  | mmu_circRNA_007495 | Down |
|  |  |  |  | mmu_circRNA_22326 | Down |
|  |  |  |  | mmu_circRNA_43578 | Down |
|  |  |  |  | mmu_circRNA_19218 | Down |
|  |  |  |  | mmu_circRNA_29284 | Down |
|  |  |  |  | mmu_circRNA_40181 | Down |
|  |  |  |  | mmu_circRNA_007507 | Down |
|  |  |  |  | mmu_circRNA_001914 | Down |
|  |  |  |  | mmu_circRNA_20332 | Down |
|  |  |  |  | mmu_circRNA_35001 | Down |
|  |  |  |  | mmu_circRNA_23582 | Down |
|  |  |  |  | mmu_circRNA_40314 | Down |
|  |  |  |  | mmu_circRNA_012214 | Down |
|  |  |  |  | mmu_circRNA_26830 | Down |
|  |  |  |  | mmu_circRNA_006636 | Down |
|  |  |  |  | mmu_circRNA_23578 | Down |
|  |  |  |  | mmu_circRNA_014997 | Down |
|  |  |  |  | mmu_circRNA_43597 | Down |
|  |  |  |  | mmu_circRNA_015986 | Down |
|  |  |  |  | mmu_circRNA_21193 | Down |
|  |  |  |  | mmu_circRNA_017050 | Down |
|  |  |  |  | mmu_circRNA_37689 | Down |
|  |  |  |  | mmu_circRNA_011031 | Down |
|  |  |  |  | mmu_circRNA_44181 | Down |
|  |  |  |  | mmu_circRNA_012091 | Down |
|  |  |  |  | mmu_circRNA_23442 | Down |
|  |  |  |  | mmu_circRNA_002959 | Down |
|  |  |  |  | mmu_circRNA_21801 | Down |
|  |  |  |  | mmu_circRNA_32437 | Down |
|  |  |  |  | mmu_circRNA_017246 | Down |
|  |  |  |  | mmu_circRNA_19481 | Down |
|  |  |  |  | mmu_circRNA_33912 | Down |
|  |  |  |  | mmu_circRNA_19450 | Down |
|  |  |  |  | mmu_circRNA_36352 | Down |
|  |  |  |  | mmu_circRNA_000903 | Down |
|  |  |  |  | mmu_circRNA_26989 | Down |

**Table S2.** Differentially expressed mRNAs of three AKI mice models.

| **I/R-AKI** | | **CP-AKI** | | **SAKI** | |
| --- | --- | --- | --- | --- | --- |
| **gene_name** | **regulation** | **gene_name** | **regulation** | **gene_name** | **regulation** |
| *Fut1* | Up | *1110008P14Rik* | Up | *Akap12* | Up |
| *Gm10375* | Up | *1500009L16Rik* | Up | *Adgb* | Up |
| *Xirp2* | Up | *1700001F09Rik* | Up | *Tnfaip3* | Up |
| *1700001F09Rik* | Up | *1700001K19Rik* | Up | *Sgk1* | Up |
| *Gm5150* | Up | *1700001P01Rik* | Up | *Vnn3* | Up |
| *Mmp8* | Up | *1700007K09Rik* | Up | *Vnn1* | Up |
| *Gm3486* | Up | *1700007K13Rik* | Up | *Arg1* | Up |
| *BC061237* | Up | *1700011E24Rik* | Up | *Cd24a* | Up |
| *Mmp27* | Up | *1700016K19Rik* | Up | *Lilrb4a* | Up |
| *Retnlg* | Up | *1700017B05Rik* | Up | *Ddit4* | Up |
| *Sprr2f* | Up | *1700024P16Rik* | Up | *Spock2* | Up |
| *Gm3573* | Up | *1700047I17Rik2* | Up | *Col13a1* | Up |
| *Saa1* | Up | *1700056E22Rik* | Up | *Hkdc1* | Up |
| *Pla2g2e* | Up | *1700080G18Rik* | Up | *Egr2* | Up |
| *Crct1* | Up | *1700092M07Rik* | Up | *4930533K18Rik* | Up |
| *Trem1* | Up | *1700109H08Rik* | Up | *Mif* | Up |
| *Spaca7* | Up | *1810011H11Rik* | Up | *Derl3* | Up |
| *Il1f6* | Up | *1810011O10Rik* | Up | *Cstb* | Up |
| *Havcr1* | Up | *1810055G02Rik* | Up | *Plpp2* | Up |
| *Serpina6* | Up | *1810062G17Rik* | Up | *Cdc34* | Up |
| *Prss22* | Up | *2010003K11Rik* | Up | *Fstl3* | Up |
| *A530064D06Rik* | Up | *2200002D01Rik* | Up | *Prss57* | Up |
| *Aqp8* | Up | *2610528A11Rik* | Up | *Abca7* | Up |
| *Tmem145* | Up | *2810417H13Rik* | Up | *Sbno2* | Up |
| *Cxcl17* | Up | *2810459M11Rik* | Up | *Cbarp* | Up |
| *Cyp21a1* | Up | *4833427G06Rik* | Up | *Pcsk4* | Up |
| *Clca4* | Up | *4921507P07Rik* | Up | *Reep6* | Up |
| *Gm3676* | Up | *4921536K21Rik* | Up | *Gadd45b* | Up |
| *Wnt7a* | Up | *4930427A07Rik* | Up | *Tle2* | Up |
| *Fgb* | Up | *4930438A08Rik* | Up | *Sirt6* | Up |
| *Uox* | Up | *4930503B20Rik* | Up | *Aldh1l2* | Up |
| *Btbd17* | Up | *4930523C07Rik* | Up | *Btg1* | Up |
| *Casp14* | Up | *4930539E08Rik* | Up | *Slc35e3* | Up |
| *Mb* | Up | *4930579G24Rik* | Up | *Irak3* | Up |
| *Tssk6* | Up | *4931409K22Rik* | Up | *Srgap1* | Up |
| *Grp* | Up | *4931423N10Rik* | Up | *Mettl1* | Up |
| *Slc26a9* | Up | *4932438H23Rik* | Up | *Agap2* | Up |
| *Ms4a8a* | Up | *4933428G20Rik* | Up | *B4galnt1* | Up |
| *Olr1* | Up | *5730507C01Rik* | Up | *Kif5a* | Up |
| *Ptprn* | Up | *5730559C18Rik* | Up | *Mbd6* | Up |
| *6430531B16Rik* | Up | *6030419C18Rik* | Up | *Ddit3* | Up |
| *Fgg* | Up | *6430531B16Rik* | Up | *Mars* | Up |
| *Gm5784* | Up | *8430408G22Rik* | Up | *Stac3* | Up |
| *Gsta1* | Up | *9030624G23Rik* | Up | *Gpr182* | Up |
| *Vgf* | Up | *9230104L09Rik* | Up | *Mmp19* | Up |
| *Vstm2b* | Up | *9530053A07Rik* | Up | *Tmem198b* | Up |
| *Gpr84* | Up | *A3galt2* | Up | *Itga7* | Up |
| *Popdc3* | Up | *A430105I19Rik* | Up | *Sec14l4* | Up |
| *Nlrp12* | Up | *A4galt* | Up | *Lif* | Up |
| *Ccdc36* | Up | *A530064D06Rik* | Up | *Polm* | Up |
| *Stfa2l1* | Up | *A630095N17Rik* | Up | *Ddx56* | Up |
| *Clec4e* | Up | *A730017C20Rik* | Up | *Upp1* | Up |
| *S100a3* | Up | *AA414768* | Up | *Stc2* | Up |
| *Fosl1* | Up | *AA467197* | Up | *Fgf18* | Up |
| *Reg3g* | Up | *AB124611* | Up | *Foxi1* | Up |
| *1700016K19Rik* | Up | *AI413582* | Up | *Timd2* | Up |
| *Irg1* | Up | *AI607873* | Up | *Havcr1* | Up |
| *Rab44* | Up | *Aaas* | Up | *D930048N14Rik* | Up |
| *Cd40lg* | Up | *Aard* | Up | *P4ha2* | Up |
| *Ceacam20* | Up | *Abcb11* | Up | *Tnip1* | Up |
| *Gm9898* | Up | *Abcb1b* | Up | *Zfp692* | Up |
| *Il1r2* | Up | *Abcc3* | Up | *Rasd1* | Up |
| *Lrrc72* | Up | *Abcc8* | Up | *Adora2b* | Up |
| *Has2* | Up | *Abcd2* | Up | *Cenpv* | Up |
| *Lrrc15* | Up | *Abcg4* | Up | *Trpv2* | Up |
| *Areg* | Up | *Abhd15* | Up | *Pik3r5* | Up |
| *Pcsk2* | Up | *Abhd4* | Up | *Mfsd6l* | Up |
| *Serpina10* | Up | *Acat3* | Up | *Per1* | Up |
| *Lilra6* | Up | *Acer2* | Up | *Chrnb1* | Up |
| *Gjb4* | Up | *Ackr4* | Up | *Cldn7* | Up |
| *Cd177* | Up | *Acod1* | Up | *Dvl2* | Up |
| *Fgf18* | Up | *Acot10* | Up | *Slc16a13* | Up |
| *Il1f9* | Up | *Acot5* | Up | *Eno3* | Up |
| *Prss16* | Up | *Acsbg1* | Up | *Smtnl2* | Up |
| *Cma1* | Up | *Acsl4* | Up | *Spns2* | Up |
| *Padi4* | Up | *Acsl5* | Up | *Ctns* | Up |
| *Arg1* | Up | *Acsl6* | Up | *Efcab5* | Up |
| *Dpf1* | Up | *Acta2* | Up | *Coro6* | Up |
| *Trem3* | Up | *Actn1* | Up | *Gm10277* | Up |
| *Cxcr2* | Up | *Ada* | Up | *Wsb1* | Up |
| *Dppa3* | Up | *Adam11* | Up | *Ccl2* | Up |
| *Chrm4* | Up | *Adam23* | Up | *Nle1* | Up |
| *Gm21677* | Up | *Adam28* | Up | *Slfn4* | Up |
| *Rfx8* | Up | *Adam8* | Up | *Wfdc21* | Up |
| *Csf3* | Up | *Adamts1* | Up | *Vmp1* | Up |
| *Akap4* | Up | *Adamts12* | Up | *Ypel2* | Up |
| *Serpina7* | Up | *Adamts2* | Up | *Gdpd1* | Up |
| *Raet1d* | Up | *Adamts4* | Up | *Lpo* | Up |
| *Kcnk10* | Up | *Adamts8* | Up | *RP23-220F20.2* | Up |
| *Vwa5b1* | Up | *Adamts9* | Up | *Epn3* | Up |
| *Gzmc* | Up | *Adamtsl2* | Up | *Itga3* | Up |
| *Krt20* | Up | *Adamtsl4* | Up | *Slc35b1* | Up |
| *Lif* | Up | *Add2* | Up | *Hoxb4* | Up |
| *Cldn14* | Up | *Adgra1* | Up | *Pcgf2* | Up |
| *Gm5269* | Up | *Adgre4* | Up | *Krt20* | Up |
| *2310005G13Rik* | Up | *Adgrf1* | Up | *Krt14* | Up |
| *Gpr150* | Up | *Adgrf3* | Up | *Hap1* | Up |
| *Dppa1* | Up | *Adgrg2* | Up | *Stat3* | Up |
| *Tmem59l* | Up | *Adgrg6* | Up | *Aoc2* | Up |
| *Gm3383* | Up | *Adgrg7* | Up | *Vat1* | Up |
| *Gm3252* | Up | *Adh4* | Up | *Arl4d* | Up |
| *Cdh9* | Up | *Adm* | Up | *Grn* | Up |
| *Gm14548* | Up | *Adora1* | Up | *Adam11* | Up |
| *Timp1* | Up | *Adora2b* | Up | *Wnt9b* | Up |
| *Hfm1* | Up | *Aen* | Up | *Itgb3* | Up |
| *Ccl20* | Up | *Agap2* | Up | *Apoh* | Up |
| *D630011A20Rik* | Up | *Ager* | Up | *Sox9* | Up |
| *Serpina3i* | Up | *Agrn* | Up | *Cd300e* | Up |
| *Adamts4* | Up | *Agt* | Up | *Cd300lf* | Up |
| *Sirpb1c* | Up | *Ahnak* | Up | *Armc7* | Up |
| *Cd300lb* | Up | *Ahnak2* | Up | *Nt5c* | Up |
| *Gm8232* | Up | *Ahsg* | Up | *Llgl2* | Up |
| *Cxcl2* | Up | *Aida* | Up | *Sphk1* | Up |
| *Tnfsf18* | Up | *Aim1l* | Up | *Rhbdf2* | Up |
| *Muc13* | Up | *Akap12* | Up | *1810032O08Rik* | Up |
| *Gm22* | Up | *Akap2* | Up | *Tha1* | Up |
| *Ptpn5* | Up | *Akap4* | Up | *Socs3* | Up |
| *Gm101* | Up | *Akr1b8* | Up | *Pgs1* | Up |
| *9530053A07Rik* | Up | *Akr1c12* | Up | *Dnah17* | Up |
| *Ankrd7* | Up | *Aldh18a1* | Up | *Cbr2* | Up |
| *B430306N03Rik* | Up | *Aldh1a1* | Up | *Hmga1-rs1* | Up |
| *Slpi* | Up | *Aldh1a2* | Up | *Slc16a3* | Up |
| *Il1rn* | Up | *Aldh1a7* | Up | *Mfsd2b* | Up |
| *Clec4n* | Up | *Alkbh2* | Up | *Sdc1* | Up |
| *Ms4a6d* | Up | *Alox5* | Up | *Dus4l* | Up |
| *Mmp7* | Up | *Alox5ap* | Up | *Bzw2* | Up |
| *Fgf21* | Up | *Aloxe3* | Up | *Ifrd1* | Up |
| *Serpinb9b* | Up | *Amh* | Up | *Gm7008* | Up |
| *Tubb3* | Up | *Amica1* | Up | *Nfkbia* | Up |
| *Egr2* | Up | *Amigo2* | Up | *Rpl36al* | Up |
| *S100a9* | Up | *Amigo3* | Up | *Rab15* | Up |
| *Chi3l3* | Up | *Ampd1* | Up | *Arg2* | Up |
| *Il11* | Up | *Ampd3* | Up | *Zfp36l1* | Up |
| *Gpr123* | Up | *Angpt4* | Up | *Plekhd1* | Up |
| *Fut2* | Up | *Angptl8* | Up | *Acot2* | Up |
| *Ccl12* | Up | *Ankk1* | Up | *Acot1* | Up |
| *BC021614* | Up | *Ankle1* | Up | *Pgf* | Up |
| *2810417H13Rik* | Up | *Ankrd1* | Up | *Fos* | Up |
| *Fam179a* | Up | *Ankrd22* | Up | *Sptlc2* | Up |
| *Gm15448* | Up | *Ankrd7* | Up | *Tshr* | Up |
| *Aldh1a2* | Up | *Anln* | Up | *Ifi27l2b* | Up |
| *Gm13247* | Up | *Ano3* | Up | *Serpina10* | Up |
| *Fga* | Up | *Anxa1* | Up | *Serpina1b* | Up |
| *Serpina3m* | Up | *Anxa2* | Up | *Serpina3f* | Up |
| *Spp1* | Up | *Anxa3* | Up | *Serpina3g* | Up |
| *Msr1* | Up | *Anxa4* | Up | *Serpina3i* | Up |
| *4930503E14Rik* | Up | *Anxa5* | Up | *Serpina3n* | Up |
| *Cyp2ab1* | Up | *Aoc1* | Up | *Ankrd9* | Up |
| *Clspn* | Up | *Aoc2* | Up | *Trmt61a* | Up |
| *S100a8* | Up | *Aox1* | Up | *Inf2* | Up |
| *Mmp10* | Up | *Ap1s2* | Up | *Zbtb42* | Up |
| *Gm5483* | Up | *Ap3b2* | Up | *Jag2* | Up |
| *Flnc* | Up | *Apaf1* | Up | *Gm6768* | Up |
| *Socs3* | Up | *Apbb1ip* | Up | *Macc1* | Up |
| *Asprv1* | Up | *Apobec3* | Up | *Calml3* | Up |
| *E230025N22Rik* | Up | *Apobr* | Up | *Net1* | Up |
| *Lcn2* | Up | *Apoc1* | Up | *Klf6* | Up |
| *Hsh2d* | Up | *Apoc3* | Up | *Heatr1* | Up |
| *Dok5* | Up | *Apoc4* | Up | *Hfe* | Up |
| *Sox9* | Up | *Apod* | Up | *Slc17a2* | Up |
| *Uhrf1* | Up | *Apold1* | Up | *Dcdc2a* | Up |
| *Mmp20* | Up | *Aqp8* | Up | *Mboat1* | Up |
| *Gad1-ps* | Up | *Aqp9* | Up | *A530084C06Rik* | Up |
| *Ccr1* | Up | *Arc* | Up | *Foxq1* | Up |
| *Fignl1* | Up | *Areg* | Up | *Foxc1* | Up |
| *Gm8229* | Up | *Arf2* | Up | *Serpinb1a* | Up |
| *Mmp9* | Up | *Arg1* | Up | *Tubb2a* | Up |
| *Onecut2* | Up | *Arg2* | Up | *Tubb2b* | Up |
| *Lgi2* | Up | *Arhgap11a* | Up | *Pxdc1* | Up |
| *Cdc6* | Up | *Arhgdig* | Up | *F13a1* | Up |
| *Slc34a2* | Up | *Arhgef37* | Up | *Tfap2a* | Up |
| *Prtn3* | Up | *Arhgef4* | Up | *Edn1* | Up |
| *Kel* | Up | *Arhgef40* | Up | *Gm9817* | Up |
| *Adam8* | Up | *Arid5a* | Up | *Gadd45g* | Up |
| *Tnf* | Up | *Arl11* | Up | *Nfil3* | Up |
| *Akap3* | Up | *Arl14* | Up | *Pdlim7* | Up |
| *Blk* | Up | *Arl4c* | Up | *Dok3* | Up |
| *Gm5799* | Up | *Arl4d* | Up | *Fam193b* | Up |
| *Ccl2* | Up | *Armc12* | Up | *Fbp2* | Up |
| *Tox2* | Up | *Armcx4* | Up | *Ctsl* | Up |
| *Jph4* | Up | *Arntl* | Up | *Cdk20* | Up |
| *Gm17416* | Up | *Arntl2* | Up | *Ftl1-ps1* | Up |
| *Baalc* | Up | *Arpc1b* | Up | *Glrx* | Up |
| *Orc1* | Up | *Arrdc2* | Up | *Vcan* | Up |
| *Eme1* | Up | *Arrdc4* | Up | *F2rl1* | Up |
| *Mefv* | Up | *Art5* | Up | *Gcnt4* | Up |
| *Bub1* | Up | *Artn* | Up | *Enc1* | Up |
| *Hist1h1a* | Up | *Asb11* | Up | *Tmem171* | Up |
| *Rad51* | Up | *Asb4* | Up | *Pik3r1* | Up |
| *Tsr2* | Up | *Asb5* | Up | *Map3k1* | Up |
| *C5ar1* | Up | *Asf1b* | Up | *Fst* | Up |
| *Col17a1* | Up | *Asns* | Up | *Emb* | Up |
| *Zfp365* | Up | *Aspm* | Up | *Kctd6* | Up |
| *Cxcl1* | Up | *Asprv1* | Up | *Comtd1* | Up |
| *Fpr1* | Up | *Atad2* | Up | *Hesx1* | Up |
| *Hist1h2ag* | Up | *Atf3* | Up | *Mustn1* | Up |
| *Spink12* | Up | *Atf4* | Up | *Itih4* | Up |
| *Akr1b8* | Up | *Atf5* | Up | *Itih3* | Up |
| *Klra8* | Up | *Atg4a* | Up | *Gnl3* | Up |
| *Nptx2* | Up | *Atg9b* | Up | *Tnnc1* | Up |
| *Gpbar1* | Up | *Atp10a* | Up | *Sema3g* | Up |
| *Trim30b* | Up | *Atp13a4* | Up | *Galnt15* | Up |
| *Serpine1* | Up | *Atp1a3* | Up | *Anxa8* | Up |
| *Smpdl3b* | Up | *Atp2b3* | Up | *Fam25c* | Up |
| *Clu* | Up | *Atp2c2* | Up | *Sh2d4b* | Up |
| *Aunip* | Up | *Aunip* | Up | *Sftpd* | Up |
| *Slfn4* | Up | *Aurka* | Up | *Gm8127* | Up |
| *Lhx2* | Up | *Aurkb* | Up | *Gch1* | Up |
| *Mfi2* | Up | *Axl* | Up | *Lgals3* | Up |
| *Atf5* | Up | *B3galt1* | Up | *Ndrg2* | Up |
| *Hist1h2ao* | Up | *B3gnt3* | Up | *Zfp219* | Up |
| *Ercc6l* | Up | *B3gnt4* | Up | *Mrpl52* | Up |
| *Mybpc2* | Up | *B3gnt6* | Up | *Mmp14* | Up |
| *4930524J08Rik* | Up | *B430306N03Rik* | Up | *Myh6* | Up |
| *Usp51* | Up | *B4galnt1* | Up | *Ripk3* | Up |
| *Slc25a31* | Up | *B4galnt2* | Up | *Pinx1* | Up |
| *Ska3* | Up | *B4galt2* | Up | *Scara3* | Up |
| *Dusp5* | Up | *B4galt6* | Up | *Clu* | Up |
| *Brca1* | Up | *B930041F14Rik* | Up | *Stc1* | Up |
| *Atf3* | Up | *BC021614* | Up | *Slc39a14* | Up |
| *Gjb3* | Up | *BC021891* | Up | *Reep4* | Up |
| *Rmi2* | Up | *BC024139* | Up | *Fam160b2* | Up |
| *Nlgn3* | Up | *BC030867* | Up | *Tsc22d1* | Up |
| *Defb36* | Up | *BC051019* | Up | *Enox1* | Up |
| *Serpina3n* | Up | *BC061237* | Up | *Bora* | Up |
| *Cenph* | Up | *BC100530* | Up | *Acod1* | Up |
| *Dscc1* | Up | *Baalc* | Up | *C6* | Up |
| *Sgol1* | Up | *Bag2* | Up | *C7* | Up |
| *Ccne1* | Up | *Bak1* | Up | *Osmr* | Up |
| *Acsbg1* | Up | *Basp1* | Up | *Dap* | Up |
| *Treml4* | Up | *Batf* | Up | *Grhl2* | Up |
| *Tnfrsf12a* | Up | *Bax* | Up | *Klf10* | Up |
| *Stil* | Up | *Bbc3* | Up | *Mal2* | Up |
| *Cd300e* | Up | *Bcam* | Up | *Wdyhv1* | Up |
| *Slfn9* | Up | *Bcl10* | Up | *Trib1* | Up |
| *Slc7a11* | Up | *Bcl2a1a* | Up | *Myc* | Up |
| *Il6* | Up | *Bcl2a1b* | Up | *St3gal1* | Up |
| *Cass4* | Up | *Bcl2a1d* | Up | *Them6* | Up |
| *Hmga2* | Up | *Bcl2l1* | Up | *Slurp1* | Up |
| *Cd101* | Up | *Bcl2l14* | Up | *Ly6f* | Up |
| *Tnip3* | Up | *Bcl2l15* | Up | *Nrbp2* | Up |
| *Ndc80* | Up | *Bcl3* | Up | *Smpd5* | Up |
| *Gm8126* | Up | *Bco1* | Up | *Oplah* | Up |
| *Kntc1* | Up | *Bdkrb2* | Up | *Gm35339* | Up |
| *4930427A07Rik* | Up | *Bean1* | Up | *Hgh1* | Up |
| *Crlf1* | Up | *Begain* | Up | *Scx* | Up |
| *Ccl9* | Up | *Best2* | Up | *Slc39a4* | Up |
| *Cxcl10* | Up | *Bex2* | Up | *Gpt* | Up |
| *Bdkrb2* | Up | *Bgn* | Up | *Csf2rb* | Up |
| *Cdh17* | Up | *Bhlha15* | Up | *Maff* | Up |
| *Lrr1* | Up | *Birc2* | Up | *Apobec3* | Up |
| *1810033B17Rik* | Up | *Birc3* | Up | *Atf4* | Up |
| *C3* | Up | *Birc5* | Up | *Mchr1* | Up |
| *Rgs16* | Up | *Blcap* | Up | *Cyp2d22* | Up |
| *Kif2c* | Up | *Blm* | Up | *Cyp2d9* | Up |
| *Tnfaip6* | Up | *Bloc1s2* | Up | *A4galt* | Up |
| *Chtf18* | Up | *Bmp1* | Up | *Prr5* | Up |
| *Tfr2* | Up | *Bmp3* | Up | *Arhgap8* | Up |
| *Ankrd1* | Up | *Bmp8b* | Up | *Alg12* | Up |
| *Nxpe5* | Up | *Bmper* | Up | *Creld2* | Up |
| *Gm9930* | Up | *Bnipl* | Up | *Pim3* | Up |
| *Cpn2* | Up | *Bora* | Up | *Trabd* | Up |
| *Exo1* | Up | *Bpifc* | Up | *Chkb* | Up |
| *Hist1h3c* | Up | *Brca1* | Up | *C730034F03Rik* | Up |
| *Mcm5* | Up | *Bst1* | Up | *Slc38a2* | Up |
| *Tdrd1* | Up | *Bst2* | Up | *Amigo2* | Up |
| *Il1b* | Up | *Btbd17* | Up | *Rnd1* | Up |
| *Tubb6* | Up | *Btg1* | Up | *Fkbp11* | Up |
| *Shcbp1* | Up | *Btg2* | Up | *Ddn* | Up |
| *Glipr2* | Up | *Btg3* | Up | *Tuba1c* | Up |
| *Nxnl2* | Up | *Btn1a1* | Up | *Bcdin3d* | Up |
| *Chaf1b* | Up | *Bub1* | Up | *Aqp2* | Up |
| *Btbd16* | Up | *Bub1b* | Up | *Aqp6* | Up |
| *Gp49a* | Up | *C1qa* | Up | *Csrnp2* | Up |
| *Hist1h3a* | Up | *C1qtnf6* | Up | *Grasp* | Up |
| *Cd44* | Up | *C2cd4b* | Up | *Nr4a1* | Up |
| *Esco2* | Up | *C3* | Up | *Krt7* | Up |
| *Dclk1* | Up | *C330027C09Rik* | Up | *Krt5* | Up |
| *Melk* | Up | *C4bp* | Up | *Krt18* | Up |
| *Ctrl* | Up | *C5ar1* | Up | *Tns2* | Up |
| *Cenpi* | Up | *C6* | Up | *Soat2* | Up |
| *Rgs1* | Up | *C7* | Up | *Itga5* | Up |
| *Sec1* | Up | *C77080* | Up | *Ppl* | Up |
| *Iqgap3* | Up | *C77370* | Up | *Litaf* | Up |
| *Pilra* | Up | *Cabyr* | Up | *Zc3h7a* | Up |
| *E2f8* | Up | *Cad* | Up | *Myh11* | Up |
| *Arg2* | Up | *Calcb* | Up | *Cebpd* | Up |
| *Aoc1* | Up | *Calcr* | Up | *Sdf2l1* | Up |
| *Rrm2* | Up | *Cald1* | Up | *Car15* | Up |
| *Aurkb* | Up | *Calml3* | Up | *Gm20518* | Up |
| *Sprr1a* | Up | *Camk2b* | Up | *Comt* | Up |
| *Frmd7* | Up | *Camkk1* | Up | *Cldn5* | Up |
| *Unc5cl* | Up | *Camkk2* | Up | *Ece2* | Up |
| *Top2a* | Up | *Capg* | Up | *Camk2n2* | Up |
| *Cd300lf* | Up | *Capn2* | Up | *Chrd* | Up |
| *Rad51ap1* | Up | *Capn5* | Up | *Tmem41a* | Up |
| *BC030867* | Up | *Capns2* | Up | *Igf2bp2* | Up |
| *Il5ra* | Up | *Capsl* | Up | *Kng1* | Up |
| *Piwil4* | Up | *Car13* | Up | *St6gal1* | Up |
| *Maff* | Up | *Card14* | Up | *Hes1* | Up |
| *Emr4* | Up | *Card9* | Up | *Apod* | Up |
| *Tnfrsf9* | Up | *Carhsp1* | Up | *Muc4* | Up |
| *Lrg1* | Up | *Casc5* | Up | *Muc20* | Up |
| *2010003K11Rik* | Up | *Casp14* | Up | *Ildr1* | Up |
| *Hist1h3h* | Up | *Casp4* | Up | *Igsf11* | Up |
| *Neil3* | Up | *Casp7* | Up | *Retnlg* | Up |
| *Hells* | Up | *Casq1* | Up | *Nfkbiz* | Up |
| *Cdk1* | Up | *Cast* | Up | *Gm11146* | Up |
| *Gdf15* | Up | *Catsper4* | Up | *Jam2* | Up |
| *Tnc* | Up | *Cav2* | Up | *Adamts1* | Up |
| *Kif14* | Up | *Cav3* | Up | *Eva1c* | Up |
| *Gzma* | Up | *Cbln2* | Up | *Slc5a3* | Up |
| *Samd5* | Up | *Cbr1* | Up | *Kcne1* | Up |
| *F13a1* | Up | *Cbr3* | Up | *Rcan1* | Up |
| *Mcm3* | Up | *Ccbe1* | Up | *Setd4* | Up |
| *Gtse1* | Up | *Ccdc109b* | Up | *Cbr1* | Up |
| *Lgals3* | Up | *Ccdc116* | Up | *Cbr3* | Up |
| *Nsl1* | Up | *Ccdc120* | Up | *Tiam2* | Up |
| *Inhba* | Up | *Ccdc154* | Up | *Acat3* | Up |
| *Ncapg* | Up | *Ccdc168* | Up | *Tcp10b* | Up |
| *4932438H23Rik* | Up | *Ccdc182* | Up | *Spaca6* | Up |
| *Asf1b* | Up | *Ccdc184* | Up | *Fpr1* | Up |
| *Hist1h2ai* | Up | *Ccdc63* | Up | *Fpr2* | Up |
| *Kif15* | Up | *Ccdc69* | Up | *Tnfrsf12a* | Up |
| *Nek5* | Up | *Ccdc71l* | Up | *E4f1* | Up |
| *Pilrb1* | Up | *Ccdc86* | Up | *Noxo1* | Up |
| *Hist1h2bj* | Up | *Ccl11* | Up | *Tbl3* | Up |
| *Itgam* | Up | *Ccl17* | Up | *Rpl3l* | Up |
| *Hdc* | Up | *Ccl2* | Up | *Msrb1* | Up |
| *Mt2* | Up | *Ccl20* | Up | *Lmf1* | Up |
| *Medag* | Up | *Ccl24* | Up | *Msln* | Up |
| *Clec5a* | Up | *Ccl28* | Up | *Rgs11* | Up |
| *Raet1e* | Up | *Ccl6* | Up | *Luc7l* | Up |
| *Chek1* | Up | *Ccl7* | Up | *Hmga1* | Up |
| *Tnfrsf23* | Up | *Ccl9* | Up | *AI413582* | Up |
| *Bmp8b* | Up | *Ccna2* | Up | *Scube3* | Up |
| *Pbk* | Up | *Ccnb1* | Up | *Fkbp5* | Up |
| *Ska1* | Up | *Ccnb2* | Up | *Mapk13* | Up |
| *Fos* | Up | *Ccne1* | Up | *4930539E08Rik* | Up |
| *Lilrb4* | Up | *Ccne2* | Up | *Cdkn1a* | Up |
| *Pole* | Up | *Ccnf* | Up | *Pi16* | Up |
| *Gins2* | Up | *Ccng1* | Up | *Pim1* | Up |
| *Tcf19* | Up | *Ccno* | Up | *Rrp1b* | Up |
| *Diap3* | Up | *Ccp110* | Up | *Notch3* | Up |
| *Nuf2* | Up | *Ccr1* | Up | *Cyp4f17* | Up |
| *Tmem173* | Up | *Ccsap* | Up | *Zfp811* | Up |
| *Hist1h2bm* | Up | *Cd109* | Up | *Pram1* | Up |
| *Steap1* | Up | *Cd14* | Up | *Angptl4* | Up |
| *Krt4* | Up | *Cd151* | Up | *Col11a2* | Up |
| *Fgr* | Up | *Cd163* | Up | *Ager* | Up |
| *Bcl3* | Up | *Cd177* | Up | *Hspa1b* | Up |
| *Car13* | Up | *Cd244* | Up | *Hspa1a* | Up |
| *Kif18b* | Up | *Cd248* | Up | *Gm20481* | Up |
| *Eps8l3* | Up | *Cd24a* | Up | *Hspa1l* | Up |
| *Hist1h1b* | Up | *Cd274* | Up | *Lsm2* | Up |
| *P2ry13* | Up | *Cd276* | Up | *Tnf* | Up |
| *Mthfd2* | Up | *Cd2ap* | Up | *Ier3* | Up |
| *Spdl1* | Up | *Cd300e* | Up | *Flot1* | Up |
| *Cd14* | Up | *Cd300lb* | Up | *Nrm* | Up |
| *Cdkn1a* | Up | *Cd300ld* | Up | *Mrps18b* | Up |
| *Rnd1* | Up | *Cd300lf* | Up | *Trim39* | Up |
| *Anxa3* | Up | *Cd33* | Up | *Pla2g7* | Up |
| *Bcl2a1a* | Up | *Cd44* | Up | *Slc25a27* | Up |
| *Ppm1j* | Up | *Cd68* | Up | *Rcan2* | Up |
| *Slc25a24* | Up | *Cd80* | Up | *Tmem151b* | Up |
| *Ifitm6* | Up | *Cd82* | Up | *Foxp4* | Up |
| *Pemt* | Up | *Cd9* | Up | *Mocs1* | Up |
| *Egr1* | Up | *Cd93* | Up | *Stap2* | Up |
| *Ptger2* | Up | *Cdc20* | Up | *Plin4* | Up |
| *Gm7233* | Up | *Cdc25c* | Up | *Plin5* | Up |
| *Tnfsf8* | Up | *Cdc42bpg* | Up | *Lrg1* | Up |
| *Ugt1a9* | Up | *Cdc42ep4* | Up | *Sema6b* | Up |
| *Cdca5* | Up | *Cdc6* | Up | *Ticam1* | Up |
| *Hesx1* | Up | *Cdc7* | Up | *Plin3* | Up |
| *Ncaph* | Up | *Cdca2* | Up | *C3* | Up |
| *Ckap2* | Up | *Cdca3* | Up | *Vav1* | Up |
| *Atp1a3* | Up | *Cdca4* | Up | *Myom1* | Up |
| *Ccne2* | Up | *Cdca5* | Up | *Emilin2* | Up |
| *Dnmt3l* | Up | *Cdca7* | Up | *Xdh* | Up |
| *4930539E08Rik* | Up | *Cdca8* | Up | *Gm10309* | Up |
| *Ttk* | Up | *Cdcp1* | Up | *Gm10184* | Up |
| *Col3a1* | Up | *Cdh17* | Up | *Map3k8* | Up |
| *Ifitm10* | Up | *Cdh24* | Up | *Kctd1* | Up |
| *D17H6S56E-5* | Up | *Cdh3* | Up | *B4galt6* | Up |
| *Akr1c20* | Up | *Cdh6* | Up | *Slc39a6* | Up |
| *Lig1* | Up | *Cdhr1* | Up | *Celf4* | Up |
| *Nlrp3* | Up | *Cdk1* | Up | *4933408B17Rik* | Up |
| *Gins1* | Up | *Cdk5r1* | Up | *Egr1* | Up |
| *Hist1h3g* | Up | *Cdk5r2* | Up | *Tmem173* | Up |
| *Selp* | Up | *Cdk6* | Up | *Hbegf* | Up |
| *Ncapg2* | Up | *Cdkn1a* | Up | *Slc4a9* | Up |
| *Fbxo48* | Up | *Cdkn2b* | Up | *Eif4ebp3* | Up |
| *Mis18bp1* | Up | *Cdkn3* | Up | *E230025N22Rik* | Up |
| *Gls2* | Up | *Cdr2l* | Up | *Cd14* | Up |
| *Slco5a1* | Up | *Cdsn* | Up | *Npy6r* | Up |
| *Cyp4f18* | Up | *Cdt1* | Up | *Eif1a* | Up |
| *Fcgr1* | Up | *Cebpb* | Up | *Lox* | Up |
| *Relb* | Up | *Cebpd* | Up | *Ctxn3* | Up |
| *Nrm* | Up | *Celf4* | Up | *Smim3* | Up |
| *Vtcn1* | Up | *Celf5* | Up | *Synpo* | Up |
| *Junb* | Up | *Celsr3* | Up | *Arhgef37* | Up |
| *Ptrh1* | Up | *Cenpa* | Up | *Pcyox1l* | Up |
| *Anxa2* | Up | *Cenpf* | Up | *Sh3tc2* | Up |
| *Adam12* | Up | *Cenph* | Up | *Tubb6* | Up |
| *Dcdc2a* | Up | *Cenpi* | Up | *Ptpn2* | Up |
| *C4b* | Up | *Cenpk* | Up | *Mapk4* | Up |
| *Ankle1* | Up | *Cenpm* | Up | *Mbd1* | Up |
| *Hist1h4a* | Up | *Cenpt* | Up | *Cfap53* | Up |
| *Tmem95* | Up | *Cenpu* | Up | *Slc14a2* | Up |
| *Bard1* | Up | *Cenpw* | Up | *Chka* | Up |
| *Ckap2l* | Up | *Cep170* | Up | *Doc2g* | Up |
| *Cdca7* | Up | *Cep55* | Up | *Gstp3* | Up |
| *4930579G24Rik* | Up | *Cercam* | Up | *Cdk2ap2* | Up |
| *Pilrb2* | Up | *Cerk* | Up | *Clcf1* | Up |
| *Cd244* | Up | *Ces2e* | Up | *Ssh3* | Up |
| *Bin2* | Up | *Ces2f* | Up | *Syt12* | Up |
| *Arl11* | Up | *Cfap52* | Up | *2010003K11Rik* | Up |
| *Ch25h* | Up | *Cfap53* | Up | *Rce1* | Up |
| *Lrp8* | Up | *Cfh* | Up | *Rin1* | Up |
| *E2f7* | Up | *Cfp* | Up | *Fosl1* | Up |
| *Recql4* | Up | *Cgn* | Up | *Snx15* | Up |
| *Cep55* | Up | *Ch25h* | Up | *Ppp1r14b* | Up |
| *Tmem253* | Up | *Chac1* | Up | *Wdr74* | Up |
| *Dusp8* | Up | *Chaf1b* | Up | *RP23-62O7.9* | Up |
| *Rad51b* | Up | *Chek1* | Up | *Fth1* | Up |
| *Wfdc3* | Up | *Chil1* | Up | *Ms4a4a* | Up |
| *Cd80* | Up | *Chil3* | Up | *Ms4a6d* | Up |
| *Mab21l3* | Up | *Chit1* | Up | *Dtx4* | Up |
| *1300002K09Rik* | Up | *Chka* | Up | *Psat1* | Up |
| *Trip13* | Up | *Chmp4c* | Up | *Trpm6* | Up |
| *Fanci* | Up | *Chrm4* | Up | *Aldh1a1* | Up |
| *Espl1* | Up | *Chrnb1* | Up | *Aldh1a7* | Up |
| *Ccnf* | Up | *Chrnb4* | Up | *Zfand5* | Up |
| *Kifc1* | Up | *Chrnd* | Up | *Gda* | Up |
| *Ung* | Up | *Chst13* | Up | *Fas* | Up |
| *Rad54l* | Up | *Chsy3* | Up | *Ch25h* | Up |
| *Kcnn4* | Up | *Chtf18* | Up | *Ankrd1* | Up |
| *Col5a3* | Up | *Cidec* | Up | *Rbp4* | Up |
| *Asns* | Up | *Cilp2* | Up | *Slc35g1* | Up |
| *Eif4ebp1* | Up | *Ckap2* | Up | *Plce1* | Up |
| *Slc15a3* | Up | *Ckap2l* | Up | *Aldh18a1* | Up |
| *Rad54b* | Up | *Ckap4* | Up | *Entpd1* | Up |
| *Gm6169* | Up | *Cklf* | Up | *Rrp12* | Up |
| *Hspb1* | Up | *Cks1b* | Up | *Ankrd2* | Up |
| *St8sia2* | Up | *Clca3a1* | Up | *Pdzd7* | Up |
| *Rrad* | Up | *Clca3a2* | Up | *Kcnip2* | Up |
| *Naip1* | Up | *Clca3b* | Up | *Nfkb2* | Up |
| *Hist1h2bl* | Up | *Clcf1* | Up | *Psd* | Up |
| *Slc11a1* | Up | *Cldn1* | Up | *Wbp1l* | Up |
| *Tmem156* | Up | *Cldn14* | Up | *Dusp5* | Up |
| *Sfn* | Up | *Cldn23* | Up | *Plekhs1* | Up |
| *Trem2* | Up | *Cldn3* | Up | *Sulf1* | Up |
| *Nefm* | Up | *Cldn4* | Up | *Ly96* | Up |
| *Apitd1* | Up | *Cldn6* | Up | *Ptp4a1* | Up |
| *Tpx2* | Up | *Cldn7* | Up | *Bag2* | Up |
| *A2m* | Up | *Clec2f* | Up | *Arid5a* | Up |
| *Pkmyt1* | Up | *Clec2g* | Up | *Ankrd23* | Up |
| *Xkr4* | Up | *Clec4d* | Up | *Gm42417* | Up |
| *Ptafr* | Up | *Clec4e* | Up | *Ankrd39* | Up |
| *Il34* | Up | *Clec4n* | Up | *Npas2* | Up |
| *Fam111a* | Up | *Clec5a* | Up | *Il1r2* | Up |
| *Ctxn1* | Up | *Clic1* | Up | *Slc9a4* | Up |
| *Traip* | Up | *Clspn* | Up | *Als2cr12* | Up |
| *Myc* | Up | *Clu* | Up | *Mpp4* | Up |
| *Hmox1* | Up | *Cmklr1* | Up | *Nop58* | Up |
| *Glipr1* | Up | *Cmtm3* | Up | *Cxcr2* | Up |
| *Cd68* | Up | *Cnfn* | Up | *Slc11a1* | Up |
| *Gm6133* | Up | *Cnga3* | Up | *Slc23a3* | Up |
| *Cpe* | Up | *Cnih2* | Up | *Tuba4a* | Up |
| *Hk3* | Up | *Cnih3* | Up | *Slc4a3* | Up |
| *Clcf1* | Up | *Cnksr1* | Up | *A630001G21Rik* | Up |
| *Col1a1* | Up | *Cnp* | Up | *B3gnt7* | Up |
| *Trib3* | Up | *Cntn1* | Up | *Arl4c* | Up |
| *Rtkn2* | Up | *Cntn2* | Up | *Sh3bp4* | Up |
| *Hist1h2ah* | Up | *Cobl* | Up | *Gpr35* | Up |
| *Nt5c1a* | Up | *Col11a2* | Up | *2310007B03Rik* | Up |
| *Emilin2* | Up | *Col12a1* | Up | *Inhbb* | Up |
| *Ripk3* | Up | *Col13a1* | Up | *Tmem37* | Up |
| *Lat* | Up | *Col15a1* | Up | *Marco* | Up |
| *Dnaaf3* | Up | *Col17a1* | Up | *Gpr39* | Up |
| *Saa4* | Up | *Col18a1* | Up | *Lypd1* | Up |
| *Csf3r* | Up | *Col1a1* | Up | *AA986860* | Up |
| *Depdc1a* | Up | *Col1a2* | Up | *Pigr* | Up |
| *Chrnb1* | Up | *Col3a1* | Up | *Mapkapk2* | Up |
| *Npas2* | Up | *Col4a1* | Up | *Ikbke* | Up |
| *Cdc7* | Up | *Col4a2* | Up | *Slc45a3* | Up |
| *Edn1* | Up | *Col5a1* | Up | *Nuak2* | Up |
| *Mcm4* | Up | *Col5a3* | Up | *Tmem81* | Up |
| *2200002D01Rik* | Up | *Col6a1* | Up | *Ren1* | Up |
| *Sirpb1b* | Up | *Col6a2* | Up | *Btg2* | Up |
| *Mki67* | Up | *Col7a1* | Up | *Mybph* | Up |
| *5730507C01Rik* | Up | *Col8a1* | Up | *Adora1* | Up |
| *Mall* | Up | *Col9a3* | Up | *Syt2* | Up |
| *Gm216* | Up | *Colec12* | Up | *Elf3* | Up |
| *Lgals1* | Up | *Coro2a* | Up | *Shisa4* | Up |
| *Aldh1a7* | Up | *Cox6b2* | Up | *Tnnt2* | Up |
| *Chac1* | Up | *Cp* | Up | *5730559C18Rik* | Up |
| *Acat3* | Up | *Cpa2* | Up | *Rgs2* | Up |
| *Sh2b2* | Up | *Cpe* | Up | *Ptgs2* | Up |
| *Zwilch* | Up | *Cpn2* | Up | *Lamc2* | Up |
| *Ulbp1* | Up | *Cpne2* | Up | *Ier5* | Up |
| *Alox5* | Up | *Cpne5* | Up | *Qsox1* | Up |
| *Hmga1* | Up | *Cpne8* | Up | *Tdrd5* | Up |
| *Polq* | Up | *Cpt1c* | Up | *Nphs2* | Up |
| *Tifa* | Up | *Crabp1* | Up | *Sele* | Up |
| *Tlr13* | Up | *Crct1* | Up | *Selp* | Up |
| *Krt8* | Up | *Creb3l3* | Up | *Creg1* | Up |
| *Mvp* | Up | *Creb5* | Up | *Fcer1g* | Up |
| *Plek* | Up | *Crip1* | Up | *Adamts4* | Up |
| *Trpv6* | Up | *Crip2* | Up | *B4galt3* | Up |
| *S100a10* | Up | *Crisp1* | Up | *Nectin4* | Up |
| *Nfkbiz* | Up | *Crispld2* | Up | *Cd244* | Up |
| *Casc5* | Up | *Crlf1* | Up | *Kcnj10* | Up |
| *Peg12* | Up | *Crlf2* | Up | *Grem2* | Up |
| *Gpnmb* | Up | *Crmp1* | Up | *Gm1305* | Up |
| *Ccsap* | Up | *Crocc2* | Up | *Mfsd7b* | Up |
| *Rin1* | Up | *Cryab* | Up | *Atf3* | Up |
| *Ctsw* | Up | *Crybb1* | Up | *Plxna2* | Up |
| *Treml2* | Up | *Csf1* | Up | *Proser2* | Up |
| *Mmp19* | Up | *Csf2* | Up | *4930412O13Rik* | Up |
| *Dusp10* | Up | *Csf2rb* | Up | *Pfkfb3* | Up |
| *Kif11* | Up | *Csf2rb2* | Up | *Hacd1* | Up |
| *Pla2g4c* | Up | *Csf3* | Up | *Nebl* | Up |
| *Capg* | Up | *Csf3r* | Up | *Il1f6* | Up |
| *Chaf1a* | Up | *Csn3* | Up | *Il1rn* | Up |
| *Aldh18a1* | Up | *Csnk1e* | Up | *Fam166a* | Up |
| *Foxm1* | Up | *Csrnp1* | Up | *Ptgds* | Up |
| *Sorcs1* | Up | *Csrp1* | Up | *Bmyc* | Up |
| *Runx1* | Up | *Cst6* | Up | *Card9* | Up |
| *Ccnb1* | Up | *Cstb* | Up | *Agpat2* | Up |
| *Fgf2* | Up | *Ctgf* | Up | *Adamtsl2* | Up |
| *Hk2* | Up | *Ctla2a* | Up | *Pkn3* | Up |
| *Ypel4* | Up | *Ctla2b* | Up | *Zdhhc12* | Up |
| *Prc1* | Up | *Ctps* | Up | *Endog* | Up |
| *Lyz2* | Up | *Ctrb1* | Up | *Kyat1* | Up |
| *Ms4a4c* | Up | *Ctrl* | Up | *Phyhd1* | Up |
| *Piwil2* | Up | *Ctsc* | Up | *Ier5l* | Up |
| *1110002E22Rik* | Up | *Ctsd* | Up | *1110008P14Rik* | Up |
| *Rsph4a* | Up | *Ctsk* | Up | *Lcn2* | Up |
| *4930486L24Rik* | Up | *Ctss* | Up | *Slc25a25* | Up |
| *Fndc4* | Up | *Cttnbp2nl* | Up | *Tor2a* | Up |
| *Card9* | Up | *Ctxn1* | Up | *Ptrh1* | Up |
| *4632434I11Rik* | Up | *Cuzd1* | Up | *Cfap157* | Up |
| *Cdt1* | Up | *Cxadr* | Up | *Hspa5* | Up |
| *Anln* | Up | *Cxcl1* | Up | *Traf1* | Up |
| *Klrc2* | Up | *Cxcl10* | Up | *Crb2* | Up |
| *Creb5* | Up | *Cxcl13* | Up | *Lypd6b* | Up |
| *Mms22l* | Up | *Cxcl17* | Up | *Kcnh7* | Up |
| *Nlrp1a* | Up | *Cxcl2* | Up | *Grb14* | Up |
| *Itgb2* | Up | *Cxcl3* | Up | *Myo3b* | Up |
| *Ccna2* | Up | *Cxcl5* | Up | *Olfr1033* | Up |
| *Cldn1* | Up | *Cxcr2* | Up | *Ptprj* | Up |
| *Cdc20* | Up | *Cxcr4* | Up | *C1qtnf4* | Up |
| *Jazf1* | Up | *Cyb5r2* | Up | *Gm13889* | Up |
| *Wisp1* | Up | *Cybrd1* | Up | *Gm10800* | Up |
| *Inhbb* | Up | *Cyp1b1* | Up | *Ehf* | Up |
| *Gm10355* | Up | *Cyp21a1* | Up | *Nat10* | Up |
| *Crlf2* | Up | *Cyp26b1* | Up | *Wt1* | Up |
| *Plp2* | Up | *Cyp2b10* | Up | *Scg5* | Up |
| *Wdhd1* | Up | *Cyp2d10* | Up | *Pak6* | Up |
| *Gpr176* | Up | *Cyp2s1* | Up | *A430105I19Rik* | Up |
| *Hist1h2ab* | Up | *Cyp3a13* | Up | *Chac1* | Up |
| *Dhrs9* | Up | *Cyp4f16* | Up | *Chp1* | Up |
| *Cenpn* | Up | *Cyp4f17* | Up | *Gm28042* | Up |
| *Vat1* | Up | *Cyp4f18* | Up | *Pla2g4b* | Up |
| *Mmp12* | Up | *Cyp4v3* | Up | *Catsper2* | Up |
| *Atad2* | Up | *Cyr61* | Up | *AA467197* | Up |
| *Csf1* | Up | *Cysltr1* | Up | *Slc24a5* | Up |
| *Cks1b* | Up | *Cyyr1* | Up | *Dusp2* | Up |
| *Icos* | Up | *D17H6S56E-5* | Up | *Zfp661* | Up |
| *Kif4* | Up | *D830031N03Rik* | Up | *Mal* | Up |
| *Gprc5a* | Up | *DXBay18* | Up | *Slc20a1* | Up |
| *Hist1h2ak* | Up | *Dap* | Up | *Il1b* | Up |
| *BC055324* | Up | *Dapp1* | Up | *Nop56* | Up |
| *Nek6* | Up | *Dbf4* | Up | *Ap5s1* | Up |
| *Cd300ld* | Up | *Dbndd1* | Up | *Srxn1* | Up |
| *Psrc1* | Up | *Dcdc2a* | Up | *Trib3* | Up |
| *Litaf* | Up | *Dclk1* | Up | *Bcl2l1* | Up |
| *Tubb2b* | Up | *Dcn* | Up | *Hck* | Up |
| *Gpr35* | Up | *Dctd* | Up | *Mmp24* | Up |
| *Ncf4* | Up | *Ddias* | Up | *BC029722* | Up |
| *Mb21d1* | Up | *Ddit4* | Up | *Eif6* | Up |
| *Sgol2* | Up | *Ddit4l* | Up | *Dlgap4* | Up |
| *Cldn4* | Up | *Ddx39* | Up | *Sla2* | Up |
| *Fam83e* | Up | *Def6* | Up | *Lbp* | Up |
| *Vim* | Up | *Defb28* | Up | *Snhg11* | Up |
| *Plac8* | Up | *Defb36* | Up | *Mafb* | Up |
| *Pea15a* | Up | *Defb42* | Up | *Mybl2* | Up |
| *Plekho1* | Up | *Dennd2a* | Up | *Wfdc2* | Up |
| *Trim72* | Up | *Dennd2c* | Up | *Acot8* | Up |
| *Rnf39* | Up | *Depdc1a* | Up | *Cebpb* | Up |
| *Tubb5* | Up | *Dhh* | Up | *Pmepa1* | Up |
| *Ccr5* | Up | *Dhrs9* | Up | *Lama5* | Up |
| *Apbb1ip* | Up | *Dhx58* | Up | *Slco4a1* | Up |
| *Cstb* | Up | *Diaph3* | Up | *Slc17a9* | Up |
| *Mcm2* | Up | *Dio2* | Up | *Col20a1* | Up |
| *Kdelr3* | Up | *Dio3* | Up | *Car13* | Up |
| *Cfi* | Up | *Dkkl1* | Up | *Sirpb1a* | Up |
| *Icam1* | Up | *Dlgap4* | Up | *Sirpb1b* | Up |
| *Rhoh* | Up | *Dlgap5* | Up | *Cp* | Up |
| *Cd69* | Up | *Dlk2* | Up | *Slc7a11* | Up |
| *Npl* | Up | *Dmkn* | Up | *Noct* | Up |
| *Ccl6* | Up | *Dnaaf3* | Up | *Ccnl1* | Up |
| *Bcl2a1d* | Up | *Dnah1* | Up | *Fgg* | Up |
| *Foxj1* | Up | *Dnah10* | Up | *Fga* | Up |
| *Cdca8* | Up | *Dnah6* | Up | *Fgb* | Up |
| *Runx3* | Up | *Dnajb13* | Up | *Tlr2* | Up |
| *Coro1a* | Up | *Dnajb3* | Up | *Gpatch4* | Up |
| *Cyr61* | Up | *Dnase1l2* | Up | *Syt11* | Up |
| *Osmr* | Up | *Dntt* | Up | *Rusc1* | Up |
| *Myof* | Up | *Doc2a* | Up | *Pklr* | Up |
| *Rasd1* | Up | *Dok1* | Up | *Hcn3* | Up |
| *Ccdc18* | Up | *Donson* | Up | *Muc1* | Up |
| *Hpcal4* | Up | *Dpcr1* | Up | *Trim46* | Up |
| *Adamts1* | Up | *Dpep2* | Up | *Krtcap2* | Up |
| *Card14* | Up | *Dpep3* | Up | *Efna4* | Up |
| *Sbno2* | Up | *Dppa3* | Up | *S100a8* | Up |
| *Fancd2* | Up | *Dpt* | Up | *S100a9* | Up |
| *Tpm4* | Up | *Dpysl3* | Up | *Sprr2g* | Up |
| *Col8a1* | Up | *Draxin* | Up | *S100a11* | Up |
| *Hist1h4m* | Up | *Drc1* | Up | *S100a10* | Up |
| *Sdc1* | Up | *Drd1* | Up | *Celf3* | Up |
| *Fosb* | Up | *Drd3* | Up | *Psmd4* | Up |
| *Sell* | Up | *Dsc2* | Up | *Adamtsl4* | Up |
| *F2rl3* | Up | *Dsc3* | Up | *Plekho1* | Up |
| *Kcnh1* | Up | *Dscaml1* | Up | *Hmgcs2* | Up |
| *Samsn1* | Up | *Dscc1* | Up | *Vtcn1* | Up |
| *Cd84* | Up | *Dsel* | Up | *Mab21l3* | Up |
| *Golm1* | Up | *Dsg1b* | Up | *Slc16a1* | Up |
| *Klf6* | Up | *Dsg2* | Up | *Ppm1j* | Up |
| *Flot1* | Up | *Dsp* | Up | *Rhoc* | Up |
| *Col5a1* | Up | *Dspp* | Up | *Wdr77* | Up |
| *Nrg1* | Up | *Dtl* | Up | *Ovgp1* | Up |
| *Mboat1* | Up | *Dtx4* | Up | *Chil3* | Up |
| *Tmsb10* | Up | *Dusp10* | Up | *Dennd2d* | Up |
| *Ccr2* | Up | *Dusp13* | Up | *Csf1* | Up |
| *Il21r* | Up | *Dusp14* | Up | *Amy1* | Up |
| *Timeless* | Up | *Dusp5* | Up | *S1pr1* | Up |
| *Neto2* | Up | *Dusp8* | Up | *Lrrc39* | Up |
| *Msn* | Up | *Dynll1* | Up | *Slc44a3* | Up |
| *Lingo2* | Up | *Dyrk3* | Up | *Tifa* | Up |
| *Emp3* | Up | *E030030I06Rik* | Up | *Clca3a1* | Up |
| *Nyx* | Up | *E130012A19Rik* | Up | *Clca3a2* | Up |
| *Prr11* | Up | *E230025N22Rik* | Up | *Cyr61* | Up |
| *Mak* | Up | *E2f7* | Up | *Mcoln3* | Up |
| *Myo1g* | Up | *E2f8* | Up | *Ptgfr* | Up |
| *Rap2b* | Up | *Eaf1* | Up | *Gem* | Up |
| *Gpr153* | Up | *Ear2* | Up | *Decr1* | Up |
| *Bpifc* | Up | *Ecel1* | Up | *Ttpa* | Up |
| *Actn1* | Up | *Ecscr* | Up | *Unc13b* | Up |
| *Hn1l* | Up | *Eda2r* | Up | *Rusc2* | Up |
| *Basp1* | Up | *Edaradd* | Up | *Zcchc7* | Up |
| *Itga5* | Up | *Edn1* | Up | *Stra6l* | Up |
| *Lpo* | Up | *Ednrb* | Up | *Plppr1* | Up |
| *Ccl5* | Up | *Eef1a2* | Up | *Acnat2* | Up |
| *6030419C18Rik* | Up | *Efcab11* | Up | *Susd1* | Up |
| *Kif22* | Up | *Efcab5* | Up | *Hsdl2* | Up |
| *Tigd3* | Up | *Efemp2* | Up | *Slc31a2* | Up |
| *Nfam1* | Up | *Efhd2* | Up | *Rnf183* | Up |
| *Ntm* | Up | *Efna3* | Up | *Ambp* | Up |
| *Parp3* | Up | *Efna4* | Up | *Col27a1* | Up |
| *Arhgap11a* | Up | *Efna5* | Up | *Orm1* | Up |
| *Pdlim7* | Up | *Efs* | Up | *Orm2* | Up |
| *Tagln2* | Up | *Egr1* | Up | *Pappa* | Up |
| *Cd53* | Up | *Egr2* | Up | *Plin2* | Up |
| *Ydjc* | Up | *Ehd2* | Up | *Acer2* | Up |
| *Mcm7* | Up | *Ehd4* | Up | *Jun* | Up |
| *Gxylt2* | Up | *Ehf* | Up | *1700024P16Rik* | Up |
| *Fen1* | Up | *Eid3* | Up | *Cyp4a12a* | Up |
| *Ms4a6c* | Up | *Eif1a* | Up | *Cyp4a12b* | Up |
| *Jun* | Up | *Eif4ebp1* | Up | *Cyp4a14* | Up |
| *Itgax* | Up | *Eif4ebp3* | Up | *Cyp4a10* | Up |
| *Cdh3* | Up | *Eif6* | Up | *Tspan1* | Up |
| *Dtx4* | Up | *Elf3* | Up | *Hpdl* | Up |
| *Pvr* | Up | *Elf4* | Up | *Plk3* | Up |
| *Mns1* | Up | *Ell* | Up | *Ccdc24* | Up |
| *Izumo1* | Up | *Elmod1* | Up | *Slc2a1* | Up |
| *Mdga1* | Up | *Elmsan1* | Up | *Ctps* | Up |
| *S100a4* | Up | *Eme1* | Up | *Hpcal4* | Up |
| *Hist1h2bb* | Up | *Emilin1* | Up | *Rspo1* | Up |
| *Hist1h2bh* | Up | *Emilin2* | Up | *Zc3h12a* | Up |
| *Mlkl* | Up | *Emp1* | Up | *Csf3r* | Up |
| *Snx10* | Up | *Emp2* | Up | *A3galt2* | Up |
| *Lmnb1* | Up | *Emp3* | Up | *Pef1* | Up |
| *Gpx7* | Up | *Enc1* | Up | *Tinagl1* | Up |
| *Cdca2* | Up | *Enkur* | Up | *Serinc2* | Up |
| *Tuba1b* | Up | *Eno2* | Up | *Med18* | Up |
| *Gadd45a* | Up | *Enthd1* | Up | *Smpdl3b* | Up |
| *Dpep2* | Up | *Entpd1* | Up | *Fgr* | Up |
| *Gpr141* | Up | *Entpd3* | Up | *Map3k6* | Up |
| *Pcsk9* | Up | *Epcam* | Up | *Fam46b* | Up |
| *Col15a1* | Up | *Epha2* | Up | *Sfn* | Up |
| *Tnfaip8l3* | Up | *Ephx1* | Up | *Aim1l* | Up |
| *Mthfd1l* | Up | *Ephx3* | Up | *Sh3bgrl3* | Up |
| *Krt18* | Up | *Ephx4* | Up | *Cep85* | Up |
| *Tyrobp* | Up | *Epn3* | Up | *Zfp593* | Up |
| *Plin2* | Up | *Eppk1* | Up | *E130218I03Rik* | Up |
| *Timd2* | Up | *Eps8l3* | Up | *Grrp1* | Up |
| *Fblim1* | Up | *Eqtn* | Up | *Gale* | Up |
| *Mastl* | Up | *Eras* | Up | *Epha2* | Up |
| *Adamts12* | Up | *Ercc1* | Up | *Hspb7* | Up |
| *Pik3r5* | Up | *Ercc5* | Up | *Tmem82* | Up |
| *Tmem45a* | Up | *Ercc6l* | Up | *Slc25a34* | Up |
| *Slitrk4* | Up | *Ereg* | Up | *Efhd2* | Up |
| *Spns2* | Up | *Esco2* | Up | *Fhad1* | Up |
| *Fbn1* | Up | *Espl1* | Up | *Pdpn* | Up |
| *Klra2* | Up | *Esyt1* | Up | *Miip* | Up |
| *Nlrp10* | Up | *Esyt2* | Up | *Mad2l2* | Up |
| *Spred3* | Up | *Ets2* | Up | *Fbxo6* | Up |
| *Ptpn7* | Up | *Etv2* | Up | *Fbxo2* | Up |
| *Trpv2* | Up | *Etv3l* | Up | *Srm* | Up |
| *C5ar2* | Up | *Etv4* | Up | *Pgd* | Up |
| *Cyp4a12b* | Up | *Eva1c* | Up | *Spsb1* | Up |
| *Hist1h3b* | Up | *Exo1* | Up | *H6pd* | Up |
| *Aurka* | Up | *F10* | Up | *Ccnl2* | Up |
| *Gpr65* | Up | *F13a1* | Up | *9430015G10Rik* | Up |
| *Ccdc88b* | Up | *F2* | Up | *Plekhn1* | Up |
| *Hspb8* | Up | *F2r* | Up | *Samd11* | Up |
| *Psd4* | Up | *F2rl1* | Up | *1700109H08Rik* | Up |
| *Birc5* | Up | *F3* | Up | *Cfap69* | Up |
| *Nanos1* | Up | *Faim2* | Up | *Steap1* | Up |
| *Ecscr* | Up | *Fam110a* | Up | *Steap4* | Up |
| *Cdc45* | Up | *Fam111a* | Up | *Sema3e* | Up |
| *Casp4* | Up | *Fam124a* | Up | *Gm9758* | Up |
| *Rhoc* | Up | *Fam124b* | Up | *Gm10354* | Up |
| *Sacs* | Up | *Fam129a* | Up | *Gm17019* | Up |
| *Slc13a1* | Up | *Fam129b* | Up | *Nos3* | Up |
| *Tubb2a* | Up | *Fam132b* | Up | *Smarcd3* | Up |
| *Stx11* | Up | *Fam159a* | Up | *Il6* | Up |
| *Ppp1r42* | Up | *Fam159b* | Up | *Selenoi* | Up |
| *Spi1* | Up | *Fam167a* | Up | *Mapre3* | Up |
| *Arpc1b* | Up | *Fam174b* | Up | *Tcf23* | Up |
| *Fxyd5* | Up | *Fam189a1* | Up | *Krtcap3* | Up |
| *Rpa2* | Up | *Fam196a* | Up | *Fndc4* | Up |
| *Tnfrsf10b* | Up | *Fam198b* | Up | *Fosl2* | Up |
| *Pola1* | Up | *Fam20a* | Up | *Tnip2* | Up |
| *Slc16a3* | Up | *Fam212b* | Up | *Mfsd10* | Up |
| *H2-Q2* | Up | *Fam214b* | Up | *Bst1* | Up |
| *Ywhah* | Up | *Fam229a* | Up | *Fgfbp1* | Up |
| *Eda2r* | Up | *Fam26e* | Up | *Lgi2* | Up |
| *Rasgrp4* | Up | *Fam46b* | Up | *Slc34a2* | Up |
| *Galnt6* | Up | *Fam49b* | Up | *Tlr1* | Up |
| *Gm6377* | Up | *Fam57a* | Up | *Uchl1* | Up |
| *Xkr9* | Up | *Fam64a* | Up | *Shisa3* | Up |
| *Ckap4* | Up | *Fam71a* | Up | *Cwh43* | Up |
| *Fhl3* | Up | *Fam71f2* | Up | *Sult1e1* | Up |
| *Ptpn22* | Up | *Fam83a* | Up | *Gc* | Up |
| *Mlf1ip* | Up | *Fam83b* | Up | *Rassf6* | Up |
| *Dpysl3* | Up | *Fam83d* | Up | *Cxcl5* | Up |
| *S100a11* | Up | *Fam83e* | Up | *Cxcl1* | Up |
| *Hck* | Up | *Fam83f* | Up | *Cxcl2* | Up |
| *Adam11* | Up | *Fam89a* | Up | *Areg* | Up |
| *Sntg2* | Up | *Fancd2* | Up | *Parm1* | Up |
| *Rad18* | Up | *Fanci* | Up | *Sowahb* | Up |
| *Mroh3* | Up | *Fas* | Up | *Cxcl13* | Up |
| *Smc2* | Up | *Fat1* | Up | *Plac8* | Up |
| *E2f1* | Up | *Fat2* | Up | *Slc10a6* | Up |
| *Foxs1* | Up | *Faxc* | Up | *Dspp* | Up |
| *Lpcat2* | Up | *Fblim1* | Up | *Spp1* | Up |
| *Panx1* | Up | *Fbln1* | Up | *Plcxd1* | Up |
| *Wbscr17* | Up | *Fbn1* | Up | *Tmem119* | Up |
| *C3ar1* | Up | *Fbn2* | Up | *Gm10401* | Up |
| *Hmmr* | Up | *Fbp2* | Up | *Hspb8* | Up |
| *Efna2* | Up | *Fbxl13* | Up | *P2rx4* | Up |
| *Ccdc154* | Up | *Fbxo2* | Up | *Hpd* | Up |
| *F2rl1* | Up | *Fbxw9* | Up | *Dnah10* | Up |
| *Tacc3* | Up | *Fcer1g* | Up | *Ncf1* | Up |
| *Ccdc109b* | Up | *Fcgbp* | Up | *Cldn4* | Up |
| *Pcna* | Up | *Fcgr1* | Up | *Cldn3* | Up |
| *Cenpf* | Up | *Fcgr3* | Up | *Hspb1* | Up |
| *Phgdh* | Up | *Fcrlb* | Up | *Lrwd1* | Up |
| *Tvp23a* | Up | *Fen1* | Up | *Serpine1* | Up |
| *Pold1* | Up | *Fetub* | Up | *Ache* | Up |
| *Snai1* | Up | *Ffar4* | Up | *Pop7* | Up |
| *Mnda* | Up | *Fga* | Up | *Fbxo24* | Up |
| *Tfpi2* | Up | *Fgb* | Up | *Gm20605* | Up |
| *Cd276* | Up | *Fgd3* | Up | *Sap25* | Up |
| *Hpgds* | Up | *Fgf2* | Up | *Pilra* | Up |
| *Tk1* | Up | *Fgf23* | Up | *Cyp3a13* | Up |
| *Hist2h2bb* | Up | *Fgf7* | Up | *Prkar1b* | Up |
| *Knstrn* | Up | *Fgfbp3* | Up | *Rsph10b* | Up |
| *Sphk1* | Up | *Fgg* | Up | *Medag* | Up |
| *Parpbp* | Up | *Fgr* | Up | *Hepacam2* | Up |
| *Tonsl* | Up | *Fhad1* | Up | *Asb4* | Up |
| *Fjx1* | Up | *Fhdc1* | Up | *Pdk4* | Up |
| *Col6a3* | Up | *Fhl2* | Up | *Asns* | Up |
| *Ctps* | Up | *Fibin* | Up | *Slc13a1* | Up |
| *Cdkn3* | Up | *Fignl1* | Up | *Akr1b8* | Up |
| *Arid5a* | Up | *Fjx1* | Up | *Akr1b7* | Up |
| *B3gnt9* | Up | *Fkbp10* | Up | *Svopl* | Up |
| *Mgl2* | Up | *Fkbp5* | Up | *Klrg2* | Up |
| *Wdr62* | Up | *Fkbp7* | Up | *Clec5a* | Up |
| *Bub1b* | Up | *Flna* | Up | *Trpv6* | Up |
| *Slamf8* | Up | *Flnc* | Up | *Trpv5* | Up |
| *Tmem158* | Up | *Flot1* | Up | *1700034O15Rik* | Up |
| *Mcm6* | Up | *Flt1* | Up | *Aoc1* | Up |
| *Eno2* | Up | *Fmnl2* | Up | *Nfe2l3* | Up |
| *Ifi205* | Up | *Fn1* | Up | *Snx10* | Up |
| *Pim1* | Up | *Fndc4* | Up | *Hoxa4* | Up |
| *Lcp2* | Up | *Fos* | Up | *Pde1c* | Up |
| *Dck* | Up | *Fosb* | Up | *Tnip3* | Up |
| *Itgal* | Up | *Fosl1* | Up | *Gadd45a* | Up |
| *Pf4* | Up | *Fosl2* | Up | *Tacstd2* | Up |
| *Ppp1r14b* | Up | *Foxf1* | Up | *Fabp1* | Up |
| *Cp* | Up | *Foxg1* | Up | *Capg* | Up |
| *Pcdha2* | Up | *Foxj1* | Up | *Elmod3* | Up |
| *Pgbd5* | Up | *Foxl2* | Up | *Reg3g* | Up |
| *Stat4* | Up | *Foxm1* | Up | *Hk2* | Up |
| *Slc20a1* | Up | *Foxs1* | Up | *Wdr54* | Up |
| *Ncf2* | Up | *Fpr1* | Up | *Mthfd2* | Up |
| *Tuba1a* | Up | *Fpr2* | Up | *Nat8f5* | Up |
| *Gm8787* | Up | *Frk* | Up | *Nat8f2* | Up |
| *Rcn1* | Up | *Frmd5* | Up | *Sec61a1* | Up |
| *Hist1h2bk* | Up | *Fscn2* | Up | *Tmem43* | Up |
| *Myo1f* | Up | *Fst* | Up | *Slc6a6* | Up |
| *S1pr2* | Up | *Fstl1* | Up | *Gxylt2* | Up |
| *Slc7a5* | Up | *Fstl3* | Up | *Setmar* | Up |
| *Isg15* | Up | *Fut1* | Up | *Bhlhe40* | Up |
| *Mt1* | Up | *Fut2* | Up | *Cidec* | Up |
| *Hn1* | Up | *Fut4* | Up | *Tamm41* | Up |
| *Atad5* | Up | *Fxyd3* | Up | *Rassf4* | Up |
| *Aif1* | Up | *Fxyd5* | Up | *8430408G22Rik* | Up |
| *Stat3* | Up | *Fzd10* | Up | *Tuba8* | Up |
| *Apex1* | Up | *G6pdx* | Up | *Clec4a1* | Up |
| *Incenp* | Up | *Gabrp* | Up | *Clec4d* | Up |
| *Pstpip1* | Up | *Gadd45a* | Up | *Clec4e* | Up |
| *Adam19* | Up | *Gadd45b* | Up | *Ltbr* | Up |
| *Trank1* | Up | *Gal* | Up | *Scnn1a* | Up |
| *Tsc22d1* | Up | *Gale* | Up | *Tnfrsf1a* | Up |
| *Mgst1* | Up | *Galnt12* | Up | *Cd9* | Up |
| *Zfp239* | Up | *Galnt16* | Up | *Styk1* | Up |
| *Clic1* | Up | *Galnt6* | Up | *Ybx3* | Up |
| *5330417C22Rik* | Up | *Galr3* | Up | *Gprc5a* | Up |
| *Prrg4* | Up | *Garnl3* | Up | *Ptpro* | Up |
| *Pirb* | Up | *Gas2l3* | Up | *Mgst1* | Up |
| *Micall2* | Up | *Gas7* | Up | *Rergl* | Up |
| *Blm* | Up | *Gast* | Up | *Slco1a4* | Up |
| *Efhd2* | Up | *Gata6* | Up | *Gm6614* | Up |
| *Cenpe* | Up | *Gbp3* | Up | *Arntl2* | Up |
| *Cebpb* | Up | *Gbx2* | Up | *Tarm1* | Up |
| *Tes* | Up | *Gc* | Up | *Pirb* | Up |
| *Psmc3ip* | Up | *Gch1* | Up | *Leng8* | Up |
| *Pdpn* | Up | *Gclc* | Up | *Ppp1r12c* | Up |
| *Nr4a3* | Up | *Gcnt4* | Up | *Cox6b2* | Up |
| *Sash3* | Up | *Gdap1l1* | Up | *Zfp579* | Up |
| *Loxl4* | Up | *Gdf15* | Up | *Zfp772* | Up |
| *Bspry* | Up | *Gdf3* | Up | *Vmn2r29* | Up |
| *Igsf6* | Up | *Gdf9* | Up | *Gltscr1* | Up |
| *Ctss* | Up | *Gdnf* | Up | *Slc1a5* | Up |
| *Serpina3g* | Up | *Gdpd1* | Up | *Hif3a* | Up |
| *Oip5* | Up | *Gem* | Up | *Fosb* | Up |
| *Fkbp10* | Up | *Gfi1b* | Up | *Ercc1* | Up |
| *Mdfi* | Up | *Gfpt2* | Up | *Relb* | Up |
| *Il1rl2* | Up | *Ggn* | Up | *Apoc2* | Up |
| *Them6* | Up | *Ggt5* | Up | *Gm44805* | Up |
| *Arl4c* | Up | *Ggta1* | Up | *Nectin2* | Up |
| *Pole2* | Up | *Gins2* | Up | *Bcam* | Up |
| *Il18rap* | Up | *Gja1* | Up | *Bcl3* | Up |
| *F3* | Up | *Gjb3* | Up | *Pvr* | Up |
| *Tacstd2* | Up | *Gjb4* | Up | *Plaur* | Up |
| *Lrrc32* | Up | *Gjb5* | Up | *Cd177* | Up |
| *Vav1* | Up | *Gla* | Up | *Grik5* | Up |
| *Aspm* | Up | *Gli3* | Up | *Pou2f2* | Up |
| *Tlr1* | Up | *Glipr2* | Up | *Pafah1b3* | Up |
| *Col1a2* | Up | *Gls2* | Up | *Lipe* | Up |
| *Hist1h2bf* | Up | *Gm10256* | Up | *Cxcl17* | Up |
| *Pklr* | Up | *Gm10375* | Up | *Cyp2b10* | Up |
| *Tpm3-rs7* | Up | *Gm10377* | Up | *Cyp2a5* | Up |
| *Asb4* | Up | *Gm1045* | Up | *Rab4b* | Up |
| *Syt12* | Up | *Gm11100* | Up | *Gm21983* | Up |
| *Zfp593* | Up | *Gm11545* | Up | *Itpkc* | Up |
| *Gvin1* | Up | *Gm11938* | Up | *Blvrb* | Up |
| *Procr* | Up | *Gm12536* | Up | *Zfp36* | Up |
| *Ptp4a1* | Up | *Gm13067* | Up | *Lgals4* | Up |
| *Tnfrsf22* | Up | *Gm13139* | Up | *Spred3* | Up |
| *Tgfb1* | Up | *Gm13251* | Up | *2200002D01Rik* | Up |
| *Srxn1* | Up | *Gm13283* | Up | *Aplp1* | Up |
| *Epha2* | Up | *Gm13889* | Up | *Nphs1* | Up |
| *Tnfrsf1b* | Up | *Gm14025* | Up | *Proser3* | Up |
| *Ier5* | Up | *Gm14137* | Up | *Hspb6* | Up |
| *Cx3cr1* | Up | *Gm14548* | Up | *Igflr1* | Up |
| *Vmp1* | Up | *Gm16486* | Up | *Sbsn* | Up |
| *Cd24a* | Up | *Gm17359* | Up | *Lsr* | Up |
| *Pla2g5* | Up | *Gm17455* | Up | *Gramd1a* | Up |
| *Nckap1l* | Up | *Gm19345* | Up | *Siglece* | Up |
| *B4galnt1* | Up | *Gm21154* | Up | *Klk1b3* | Up |
| *Gale* | Up | *Gm21188* | Up | *Klk1b5* | Up |
| *Dsn1* | Up | *Gm21677* | Up | *Klk1* | Up |
| *Katnal2* | Up | *Gm21693* | Up | *Mybpc2* | Up |
| *Opn3* | Up | *Gm21704* | Up | *Atf5* | Up |
| *Calcr* | Up | *Gm21708* | Up | *Rras* | Up |
| *H2-DMb2* | Up | *Gm22* | Up | *Snrnp70* | Up |
| *Ctsc* | Up | *Gm2237* | Up | *Ftl1* | Up |
| *Bmyc* | Up | *Gm2240* | Up | *Ppp1r15a* | Up |
| *Rtn4* | Up | *Gm2511* | Up | *Fgf21* | Up |
| *Pik3ap1* | Up | *Gm26637* | Up | *Fut1* | Up |
| *Plch1* | Up | *Gm29733* | Up | *Mamstr* | Up |
| *Col4a1* | Up | *Gm29779* | Up | *Fut2* | Up |
| *Rims3* | Up | *Gm29801* | Up | *Sult2b1* | Up |
| *Rras* | Up | *Gm29804* | Up | *Lmtk3* | Up |
| *Eif6* | Up | *Gm30599* | Up | *Grin2d* | Up |
| *Sertad4* | Up | *Gm30732* | Up | *Saa3* | Up |
| *Ube2t* | Up | *Gm31493* | Up | *Saa4* | Up |
| *Nfkbie* | Up | *Gm31532* | Up | *Saa1* | Up |
| *Clec2i* | Up | *Gm3336* | Up | *Saa2* | Up |
| *Cpn1* | Up | *Gm3376* | Up | *Zdhhc13* | Up |
| *Tacc2* | Up | *Gm35078* | Up | *Mctp2* | Up |
| *Tgm2* | Up | *Gm3636* | Up | *Ntrk3* | Up |
| *Kif18a* | Up | *Gm36566* | Up | *Rhcg* | Up |
| *Dock2* | Up | *Gm3776* | Up | *Pex11a* | Up |
| *Kif20a* | Up | *Gm38525* | Up | *Sema4b* | Up |
| *Serpinh1* | Up | *Gm38655* | Up | *Cpeb1* | Up |
| *Lgmn* | Up | *Gm38958* | Up | *Cemip* | Up |
| *Bcmo1* | Up | *Gm39701* | Up | *Rab30* | Up |
| *Mas1* | Up | *Gm39972* | Up | *Usp35* | Up |
| *Dnajb3* | Up | *Gm40453* | Up | *Tsku* | Up |
| *Sptlc2* | Up | *Gm40514* | Up | *Chrdl2* | Up |
| *Clec4a3* | Up | *Gm40525* | Up | *Ucp3* | Up |
| *Epb4.2* | Up | *Gm4064* | Up | *Ucp2* | Up |
| *C330027C09Rik* | Up | *Gm4070* | Up | *Trim6* | Up |
| *Fam26e* | Up | *Gm40835* | Up | *Adm* | Up |
| *Smox* | Up | *Gm40991* | Up | *Ampd3* | Up |
| *Il4ra* | Up | *Gm41885* | Up | *Lyve1* | Up |
| *Cerkl* | Up | *Gm42031* | Up | *Arntl* | Up |
| *Prim1* | Up | *Gm42035* | Up | *Pde3b* | Up |
| *Mad2l1* | Up | *Gm42166* | Up | *Gprc5b* | Up |
| *Lcp1* | Up | *Gm42226* | Up | *Gp2* | Up |
| *Trim47* | Up | *Gm42368* | Up | *Pdilt* | Up |
| *Psat1* | Up | *Gm44504* | Up | *Igsf6* | Up |
| *Rfc3* | Up | *Gm4832* | Up | *Scnn1g* | Up |
| *Sox4* | Up | *Gm4943* | Up | *Scnn1b* | Up |
| *Kctd1* | Up | *Gm5150* | Up | *Il4ra* | Up |
| *Sec14l2* | Up | *Gm5431* | Up | *Nupr1* | Up |
| *Racgap1* | Up | *Gm5483* | Up | *Sult1a1* | Up |
| *Cad* | Up | *Gm5544* | Up | *Doc2a* | Up |
| *Rell1* | Up | *Gm5640* | Up | *Mvp* | Up |
| *Alox5ap* | Up | *Gm5737* | Up | *Tmem265* | Up |
| *Clec12a* | Up | *Gm5741* | Up | *Hsd3b7* | Up |
| *Aim1l* | Up | *Gm5767* | Up | *Itgam* | Up |
| *Apobr* | Up | *Gm5878* | Up | *Tacc2* | Up |
| *Abcg3* | Up | *Gm6034* | Up | *Mapk1ip1* | Up |
| *Fcgr3* | Up | *Gm6306* | Up | *Adgra1* | Up |
| *Rhbdf2* | Up | *Gm6614* | Up | *Adam8* | Up |
| *Tnfrsf1a* | Up | *Gm7233* | Up | *Fuom* | Up |
| *Lair1* | Up | *Gm7972* | Up | *Urah* | Up |
| *Birc3* | Up | *Gm8094* | Up | *RP24-243J4.5* | Up |
| *2810459M11Rik* | Up | *Gm8229* | Up | *Ifitm2* | Up |
| *Smyd5* | Up | *Gm8232* | Up | *Taldo1* | Up |
| *Fcer1g* | Up | *Gm8256* | Up | *Slc25a22* | Up |
| *Cftr* | Up | *Gm8439* | Up | *Pnpla2* | Up |
| *Dap* | Up | *Gm9047* | Up | *Muc6* | Up |
| *Mmp14* | Up | *Gm9573* | Up | *Dusp8* | Up |
| *Scimp* | Up | *Gml* | Up | *Ifitm10* | Up |
| *Mical2* | Up | *Gml2* | Up | *RP23-145I16.3* | Up |
| *Cpne7* | Up | *Gna15* | Up | *Ctsd* | Up |
| *Map3k1* | Up | *Gnal* | Up | *Kcnq1* | Up |
| *Cdk6* | Up | *Gnat1* | Up | *Cdkn1c* | Up |
| *Mpeg1* | Up | *Gngt2* | Up | *Cars* | Up |
| *Apobec1* | Up | *Gnl3* | Up | *Tnfrsf23* | Up |
| *Cdca3* | Up | *Golga7b* | Up | *Tpcn2* | Up |
| *Mmd* | Up | *Golm1* | Up | *Mcemp1* | Up |
| *Arntl2* | Up | *Got1l1* | Up | *Irs2* | Up |
| *3110007F17Rik* | Up | *Gpbar1* | Up | *F10* | Up |
| *Ccdc120* | Up | *Gpha2* | Up | *Defb1* | Up |
| *Cbr3* | Up | *Gpm6b* | Up | *Nek5* | Up |
| *Gch1* | Up | *Gpnmb* | Up | *Plat* | Up |
| *Enkur* | Up | *Gpr141* | Up | *Plpp5* | Up |
| *Hcls1* | Up | *Gpr152* | Up | *Eif4ebp1* | Up |
| *Lrfn4* | Up | *Gpr153* | Up | *Nrg1* | Up |
| *Lhfpl2* | Up | *Gpr156* | Up | *Msr1* | Up |
| *Rnh1* | Up | *Gpr161* | Up | *Ankrd37* | Up |
| *Itih3* | Up | *Gpr171* | Up | *Pbx4* | Up |
| *Comt* | Up | *Gpr176* | Up | *Ell* | Up |
| *Ly9* | Up | *Gpr182* | Up | *Gdf15* | Up |
| *Rhou* | Up | *Gpr27* | Up | *Arrdc2* | Up |
| *Adora2b* | Up | *Gpr35* | Up | *Pgls* | Up |
| *Rac2* | Up | *Gpr39* | Up | *Fam129c* | Up |
| *Rfc4* | Up | *Gpr50* | Up | *Jak3* | Up |
| *C1qa* | Up | *Gpr75* | Up | *Hmox1* | Up |
| *Cd4* | Up | *Gpr84* | Up | *2210011C24Rik* | Up |
| *Dnmt1* | Up | *Gpr85* | Up | *Mri1* | Up |
| *Gpr183* | Up | *Gprc5a* | Up | *Ier2* | Up |
| *Dok4* | Up | *Gpt* | Up | *Dnase2a* | Up |
| *Fam212b* | Up | *Gpx2* | Up | *Mast1* | Up |
| *Pask* | Up | *Gpx7* | Up | *Junb* | Up |
| *Tgif1* | Up | *Gpx8* | Up | *Tox3* | Up |
| *Flna* | Up | *Grasp* | Up | *Chd9* | Up |
| *Parvg* | Up | *Grem1* | Up | *Mt2* | Up |
| *5730559C18Rik* | Up | *Grem2* | Up | *Mt1* | Up |
| *Efcab5* | Up | *Grhl1* | Up | *Adgrg1* | Up |
| *Rdh12* | Up | *Grin1os* | Up | *Adgrg3* | Up |
| *Plec* | Up | *Grk1* | Up | *Rrad* | Up |
| *Pgd* | Up | *Grk5* | Up | *Hsd11b2* | Up |
| *Ankrd6* | Up | *Grp* | Up | *Cenpt* | Up |
| *Vcam1* | Up | *Grrp1* | Up | *Ddx28* | Up |
| *Ammecr1* | Up | *Gsdma* | Up | *Slc7a6* | Up |
| *Padi2* | Up | *Gsdmd* | Up | *Prmt7* | Up |
| *Gas2l3* | Up | *Gsta1* | Up | *Cdh3* | Up |
| *Tnfsf12* | Up | *Gstm6* | Up | *Txnl4b* | Up |
| *Zfp947* | Up | *Gtse1* | Up | *Hp* | Up |
| *Rasl12* | Up | *Guca1a* | Up | *Il34* | Up |
| *Rdh10* | Up | *Gvin1* | Up | *Slc7a5* | Up |
| *Creb3l1* | Up | *Gxylt2* | Up | *Banp* | Up |
| *Arhgap30* | Up | *Gzmm* | Up | *Ctu2* | Up |
| *Tagln* | Up | *H2-M9* | Up | *Piezo1* | Up |
| *Sco2* | Up | *H2-Q1* | Up | *Aprt* | Up |
| *Ttc39a* | Up | *H2afx* | Up | *Rhou* | Up |
| *Asic2* | Up | *H60b* | Up | *Irf2bp2* | Up |
| *Ptger4* | Up | *H6pd* | Up | *Mmp8* | Up |
| *Mapk6* | Up | *Hap1* | Up | *Mmp7* | Up |
| *Csf2rb* | Up | *Hapln3* | Up | *Birc2* | Up |
| *Cmtm3* | Up | *Has1* | Up | *Birc3* | Up |
| *Rhbdl2* | Up | *Has2* | Up | *A230050P20Rik* | Up |
| *Serpinb6b* | Up | *Havcr1* | Up | *Angptl6* | Up |
| *Isg20* | Up | *Havcr2* | Up | *Ppan* | Up |
| *Oasl1* | Up | *Hbegf* | Up | *Icam1* | Up |
| *Masp1* | Up | *Hc* | Up | *Angptl8* | Up |
| *Col18a1* | Up | *Hcar2* | Up | *Plppr2* | Up |
| *Fermt3* | Up | *Hcrtr1* | Up | *Zfp653* | Up |
| *Adamts2* | Up | *Hdac7* | Up | *Cnn1* | Up |
| *Lsr* | Up | *Hdc* | Up | *St14* | Up |
| *Abcb1b* | Up | *Hdx* | Up | *Fam118b* | Up |
| *Ctsd* | Up | *Hebp2* | Up | *Vsig2* | Up |
| *Mex3a* | Up | *Hectd2* | Up | *Zfp202* | Up |
| *Gpr64* | Up | *Heg1* | Up | *Nectin1* | Up |
| *Trmt61a* | Up | *Hells* | Up | *Hyou1* | Up |
| *Ifngr2* | Up | *Helz2* | Up | *Amica1* | Up |
| *Tlr8* | Up | *Hesx1* | Up | *Apoa1* | Up |
| *Nr2c2ap* | Up | *Heyl* | Up | *Apoc3* | Up |
| *Was* | Up | *Hfm1* | Up | *Zbtb16* | Up |
| *Gpr161* | Up | *Hgf* | Up | *Htr3a* | Up |
| *Tmem43* | Up | *Hhipl1* | Up | *Rpl10-ps3* | Up |
| *Card11* | Up | *Hhipl2* | Up | *Plet1* | Up |
| *Ablim3* | Up | *Hif3a* | Up | *Neil1* | Up |
| *Sema6b* | Up | *Hilpda* | Up | *Sema7a* | Up |
| *Nek2* | Up | *Hipk4* | Up | *Kif23* | Up |
| *Actb* | Up | *Hist1h1b* | Up | *Fbxl22* | Up |
| *Tlr2* | Up | *Hist1h1d* | Up | *Aldh1a2* | Up |
| *Pcdhb21* | Up | *Hist1h2ab* | Up | *Gsta1* | Up |
| *Arhgap9* | Up | *Hist1h2ac* | Up | *Gm3776* | Up |
| *Cenpt* | Up | *Hist1h2ae* | Up | *Ibtk* | Up |
| *Slc16a1* | Up | *Hist1h2af* | Up | *Il20rb* | Up |
| *Pdgfb* | Up | *Hist1h2ag* | Up | *Gm20425* | Up |
| *Irf4* | Up | *Hist1h2ai* | Up | *Trf* | Up |
| *Plscr1* | Up | *Hist1h2bb* | Up | *Alas1* | Up |
| *Rgs19* | Up | *Hist1h2be* | Up | *Poc1a* | Up |
| *Fosl2* | Up | *Hist1h2bf* | Up | *Manf* | Up |
| *Ypel2* | Up | *Hist1h2bg* | Up | *Cish* | Up |
| *Ier2* | Up | *Hist1h2bh* | Up | *Hyal1* | Up |
| *Atp10a* | Up | *Hist1h2bj* | Up | *Nat6* | Up |
| *Sparc* | Up | *Hist1h2bl* | Up | *Sema3f* | Up |
| *Ppap2c* | Up | *Hist1h2bm* | Up | *Lamb2* | Up |
| *Mtap7d3* | Up | *Hist1h2bn* | Up | *Slc25a20* | Up |
| *Evi2a* | Up | *Hist1h2bp* | Up | *Ip6k2* | Up |
| *Gc* | Up | *Hist1h3a* | Up | *Spink8* | Up |
| *Gmds* | Up | *Hist1h3b* | Up | *Ngp* | Up |
| *Gm9396* | Up | *Hist1h3c* | Up | *Ltf* | Up |
| *Mcam* | Up | *Hist1h3g* | Up | *Acaa1b* | Up |
| *Shisa4* | Up | *Hist1h3h* | Up | *Myd88* | Up |
| *Mob3a* | Up | *Hist1h4a* | Up | *Csrnp1* | Up |
| *Kif1a* | Up | *Hist1h4j* | Up | *Myrip* | Up |
| *Il1r1* | Up | *Hk2* | Up | *Tmem158* | Up |
| *Lrrc20* | Up | *Hkdc1* | Up | *Limd1* | Up |
| *Tcf7* | Up | *Hmga1* | Up | *Ccr1* | Up |
| *Rrm1* | Up | *Hmga1-rs1* | Up | *Foxp3* | Up |
| *Clec4a2* | Up | *Hmga2* | Up | *Ccdc22* | Up |
| *Slc35e4* | Up | *Hmmr* | Up | *Plp2* | Up |
| *Gm2237* | Up | *Hmox1* | Up | *Ccdc120* | Up |
| *Nfkbid* | Up | *Hn1* | Up | *Maoa* | Up |
| *Ifitm3* | Up | *Hn1l* | Up | *Maob* | Up |
| *Nav3* | Up | *Homer3* | Up | *Timp1* | Up |
| *D16Ertd472e* | Up | *Hoxa1* | Up | *Cfp* | Up |
| *Cdr2l* | Up | *Hoxb9* | Up | *Gabre* | Up |
| *Hist1h2bn* | Up | *Hoxc13* | Up | *Gm6377* | Up |
| *Loxl1* | Up | *Hoxd13* | Up | *Tceal3* | Up |
| *Il27ra* | Up | *Hp* | Up | *Zcchc18* | Up |
| *Igf2bp2* | Up | *Hpca* | Up | *Ripply1* | Up |
| *Palb2* | Up | *Hpcal4* | Up | *Acsl4* | Up |
| *Themis2* | Up | *Hpgds* | Up | *Adgrg2* | Up |
| *Cxcl16* | Up | *Hprt* | Up | *Nhs* | Up |
| *Syt15* | Up | *Hpse* | Up | *Ap1s2* | Up |
| *Ctsl* | Up | *Hr* | Up | *Asb11* | Up |
| *A430078G23Rik* | Up | *Hrct1* | Up | *Gm21887* | Up |
| *Anxa1* | Up | *Hs3st1* | Up | *Erdr1* | Up |
| *Maoa* | Up | *Hsh2d* | Up | *Gm17711* | Up |
| *Rdh11* | Up | *Hspa1l* | Up | *Gm23130* | Up |
| *Btk* | Up | *Hspb1* | Up | *RP24-254O21.2* | Up |
| *Ctsb* | Up | *Hspb2* | Up | *Gm17134* | Up |
| *2610524H06Rik* | Up | *Hspb8* | Up | *1810014B01Rik* | Up |
| *Gdpd1* | Up | *Htr1d* | Up | *Gm15344* | Up |
| *Topbp1* | Up | *Htr2a* | Up | *AC117232.1* | Up |
| *Ran* | Up | *Hyal1* | Up | *Mir3060* | Up |
| *Mapk4* | Up | *Hyal6* | Up | *Snhg15* | Up |
| *Ect2* | Up | *Icam1* | Up | *Gm12121* | Up |
| *Cdh24* | Up | *Ier2* | Up | *Snord96a* | Up |
| *Hilpda* | Up | *Ier5* | Up | *Gm26542* | Up |
| *Ubd* | Up | *Iffo2* | Up | *Gm25082* | Up |
| *Rbm11* | Up | *Ifi202b* | Up | *RP23-263C23.7* | Up |
| *Tipin* | Up | *Ifi204* | Up | *9430098F02Rik* | Up |
| *Prkcb* | Up | *Ifi205* | Up | *Mir3061* | Up |
| *Brsk1* | Up | *Ifi27l2a* | Up | *Gm12240* | Up |
| *Csf2rb2* | Up | *Ifi27l2b* | Up | *4933439C10Rik* | Up |
| *Zfp36l1* | Up | *Ifi30* | Up | *Gm12264* | Up |
| *Plekho2* | Up | *Ifi35* | Up | *Map2k3os* | Up |
| *Fancb* | Up | *Ifit1* | Up | *A530017D24Rik* | Up |
| *Hrasls* | Up | *Ifit3* | Up | *Gm26964* | Up |
| *Mtfr2* | Up | *Ifit3b* | Up | *2410006H16Rik* | Up |
| *Marcksl1* | Up | *Ifitm10* | Up | *Gm24029* | Up |
| *Il2rb* | Up | *Ifitm3* | Up | *C030037D09Rik* | Up |
| *Pgm1* | Up | *Ifitm5* | Up | *Gm53* | Up |
| *Pcdhb16* | Up | *Ifitm6* | Up | *Mir196a-1* | Up |
| *Cep170* | Up | *Ifitm7* | Up | *Gm11537* | Up |
| *Emp2* | Up | *Ifngr1* | Up | *Gm20659* | Up |
| *Pros1* | Up | *Ifngr2* | Up | *Arhgap27os1* | Up |
| *Ssbp4* | Up | *Ifnk* | Up | *1700052K11Rik* | Up |
| *Lmna* | Up | *Ifnlr1* | Up | *Gm11703* | Up |
| *Lat2* | Up | *Ifrd1* | Up | *Snhg20* | Up |
| *Myh9* | Up | *Iglon5* | Up | *Gm17178* | Up |
| *Gins3* | Up | *Igsf6* | Up | *Gm4419* | Up |
| *Rpl12* | Up | *Igsf9b* | Up | *Gm26669* | Up |
| *Kif21b* | Up | *Ikbke* | Up | *Gm28370* | Up |
| *Rcan1* | Up | *Il11* | Up | *Gm17139* | Up |
| *Pprc1* | Up | *Il17rc* | Up | *Gm26698* | Up |
| *Gbp2* | Up | *Il18bp* | Up | *Gm28933* | Up |
| *Slc7a1* | Up | *Il18r1* | Up | *Gm28875* | Up |
| *Cldn7* | Up | *Il18rap* | Up | *Gm17111* | Up |
| *AB124611* | Up | *Il1a* | Up | *A230087F16Rik* | Up |
| *Map1a* | Up | *Il1b* | Up | *Hsp25-ps1* | Up |
| *Zfand4* | Up | *Il1f6* | Up | *Mir3074-1* | Up |
| *Emilin1* | Up | *Il1f9* | Up | *Mir24-1* | Up |
| *Brip1* | Up | *Il1r1* | Up | *A430057M04Rik* | Up |
| *Cdk2* | Up | *Il1r2* | Up | *Gm26772* | Up |
| *Ptpre* | Up | *Il1rl1* | Up | *Mir3076* | Up |
| *Itgb3* | Up | *Il1rl2* | Up | *Gm15601* | Up |
| *Gbp8* | Up | *Il1rn* | Up | *Gm15222* | Up |
| *Cfl1* | Up | *Il20rb* | Up | *Mir6948* | Up |
| *Akap12* | Up | *Il23a* | Up | *Gm16973* | Up |
| *Fam198b* | Up | *Il24* | Up | *Gm37847* | Up |
| *Serpinb6a* | Up | *Il31ra* | Up | *Snora31* | Up |
| *Il2rg* | Up | *Il33* | Up | *Gm17066* | Up |
| *Vasp* | Up | *Il34* | Up | *Mir17hg* | Up |
| *Havcr2* | Up | *Il3ra* | Up | *Gm16136* | Up |
| *Brca2* | Up | *Il4ra* | Up | *Gm17035* | Up |
| *P2ry6* | Up | *Il5ra* | Up | *Gm26621* | Up |
| *Gm7173* | Up | *Il6* | Up | *Mirt2* | Up |
| *Anxa5* | Up | *Il6ra* | Up | *Gm23935* | Up |
| *Cx3cl1* | Up | *Il7* | Up | *1300002E11Rik* | Up |
| *Tax1bp3* | Up | *Impdh1* | Up | *Mir99ahg* | Up |
| *Sh3bgrl3* | Up | *Impg2* | Up | *4930404I05Rik* | Up |
| *Hist1h2bg* | Up | *Incenp* | Up | *Gm15964* | Up |
| *Myd88* | Up | *Inf2* | Up | *Gm16310* | Up |
| *Bend4* | Up | *Inhba* | Up | *3300005D01Rik* | Up |
| *4930506M07Rik* | Up | *Inhbb* | Up | *Gm26130* | Up |
| *Hist1h4b* | Up | *Iqgap1* | Up | *Snora20* | Up |
| *Nusap1* | Up | *Iqgap3* | Up | *Mir5125* | Up |
| *Prim2* | Up | *Irak3* | Up | *E230001N04Rik* | Up |
| *Rcc2* | Up | *Irf7* | Up | *Gm26885* | Up |
| *Tmem98* | Up | *Isg15* | Up | *Gm20507* | Up |
| *Cyp3a13* | Up | *Isg20* | Up | *Mir6972* | Up |
| *Sh2d4b* | Up | *Itga2* | Up | *1110038B12Rik* | Up |
| *Rel* | Up | *Itga3* | Up | *Gm16279* | Up |
| *Cdh11* | Up | *Itga5* | Up | *Rn18s-rs5* | Up |
| *Coro1c* | Up | *Itga6* | Up | *Gm42418* | Up |
| *Nr4a1* | Up | *Itgam* | Up | *AY036118* | Up |
| *Cars* | Up | *Itgav* | Up | *1700071M16Rik* | Up |
| *Nop58* | Up | *Itgb1* | Up | *Gm38220* | Up |
| *Ranbp1* | Up | *Itgb2* | Up | *Epb41l4aos* | Up |
| *1700016C15Rik* | Up | *Itgb3* | Up | *Snhg4* | Up |
| *Hist1h4n* | Up | *Itgb4* | Up | *Cd63-ps* | Up |
| *Bag2* | Up | *Itgb6* | Up | *A930001C03Rik* | Up |
| *Rnf150* | Up | *Itih3* | Up | *Malat1* | Up |
| *Cchcr1* | Up | *Itih4* | Up | *Gm20417* | Up |
| *Tmeff1* | Up | *Itpkc* | Up | *Gm27694* | Up |
| *Tap1* | Up | *Itpr3* | Up | *Gm37376* | Up |
| *Cd300a* | Up | *Itprip* | Up | *Neat1* | Up |
| *H2-DMb1* | Up | *Itpripl2* | Up | *Gm27533* | Up |
| *Lxn* | Up | *Ivl* | Up | *Gm27505* | Up |
| *Zc3hav1* | Up | *Izumo1* | Up | *Gm45248* | Up |
| *Btg2* | Up | *Jak3* | Up | *Gm26460* | Up |
| *Slc38a2* | Up | *Jam2* | Up | *Snhg1* | Up |
| *Samd14* | Up | *Jazf1* | Up | *Gm24452* | Up |
| *Ybx3* | Up | *Jsrp1* | Up | *Snord22* | Up |
| *Pcdhb7* | Up | *Jun* | Up | *1700039E22Rik* | Up |
| *Pknox2* | Up | *Junb* | Up | *Gm16277* | Up |
| *H2-DMa* | Up | *Kbtbd6* | Up | *Snhg6* | Up |
| *Mrpl33* | Up | *Kcnd1* | Up | *Gm37335* | Up |
| *Ptprc* | Up | *Kcnh6* | Up | *Gm15834* | Up |
| *Abca1* | Up | *Kcnip2* | Up | *Gm20257* | Up |
| *Lrmp* | Up | *Kcnj4* | Up | *Eif4a-ps4* | Up |
| *Vash1* | Up | *Kcnk1* | Up | *Snora75* | Up |
| *Gpm6b* | Up | *Kcnk10* | Up | *Gm37967* | Up |
| *Nop56* | Up | *Kcnk12* | Up | *Gm37447* | Up |
| *Elf4* | Up | *Kcnmb1* | Up | *Gm7785* | Up |
| *Atic* | Up | *Kcnq2* | Up | *Gm28857* | Up |
| *Fbl* | Up | *Kcnq4* | Up | *Gm26642* | Up |
| *Dynll1* | Up | *Kcnv2* | Up | *Gm37539* | Up |
| *Comtd1* | Up | *Kctd1* | Up | *Gm22357* | Up |
| *Gstm5* | Up | *Kctd10* | Up | *Snord78* | Up |
| *Psmb10* | Up | *Kctd11* | Up | *Gm16548* | Up |
| *Mxra7* | Up | *Kctd4* | Up | *Gm37490* | Up |
| *Cd180* | Up | *Kctd5* | Up | *Gm37033* | Up |
| *Cd37* | Up | *Kdelr3* | Up | *Pdcd5-ps* | Up |
| *Ipo4* | Up | *Kel* | Up | *Gm15867* | Up |
| *Sh3pxd2b* | Up | *Kif11* | Up | *Mir29c* | Up |
| *Bak1* | Up | *Kif14* | Up | *Gm13194* | Up |
| *Itga6* | Up | *Kif15* | Up | *Gm13375* | Up |
| *Jak3* | Up | *Kif17* | Up | *A230005M16Rik* | Up |
| *Adcyap1r1* | Up | *Kif18a* | Up | *Snhg7* | Up |
| *3110082I17Rik* | Up | *Kif18b* | Up | *Snora43* | Up |
| *Dysf* | Up | *Kif1a* | Up | *Gm16534* | Up |
| *Col4a2* | Up | *Kif20a* | Up | *Snora65* | Up |
| *Ikzf1* | Up | *Kif22* | Up | *Gm44291* | Up |
| *Phlda3* | Up | *Kif23* | Up | *Gm13552* | Up |
| *Spcs3* | Up | *Kif26b* | Up | *Gm13567* | Up |
| *Casp12* | Up | *Kif2c* | Up | *Gm13652* | Up |
| *Fscn1* | Up | *Kif4* | Up | *Gm13868* | Up |
| *Nova1* | Up | *Kifc1* | Up | *Rpl10-ps1* | Up |
| *Tgfbr2* | Up | *Kifc5b* | Up | *9830144P21Rik* | Up |
| *Lrguk* | Up | *Kirrel* | Up | *Gm26841* | Up |
| *Tpm3* | Up | *Kirrel2* | Up | *Gm16098* | Up |
| *Nup107* | Up | *Klc3* | Up | *Gm20412* | Up |
| *Spsb1* | Up | *Klf10* | Up | *Zfas1* | Up |
| *Gm21807* | Up | *Klf14* | Up | *1200007C13Rik* | Up |
| *Armcx6* | Up | *Klf2* | Up | *Gm14319* | Up |
| *Axl* | Up | *Klf4* | Up | *Ctcflos* | Up |
| *Il33* | Up | *Klf5* | Up | *Gm16685* | Up |
| *P2ry10* | Up | *Klf6* | Up | *Gm38197* | Up |
| *Fam114a1* | Up | *Klf7* | Up | *Gm30097* | Up |
| *Dusp14* | Up | *Klhdc8a* | Up | *Gm16069* | Up |
| *Ncf1* | Up | *Klhl26* | Up | *Gm42941* | Up |
| *Phf19* | Up | *Klhl29* | Up | *Gm43328* | Up |
| *Gm13248* | Up | *Klk10* | Up | *Gm42890* | Up |
| *Fam115c* | Up | *Klra2* | Up | *Gm42940* | Up |
| *Ptgfrn* | Up | *Klrb1b* | Up | *A530020G20Rik* | Up |
| *Sirpb1a* | Up | *Klrg2* | Up | *Snora24* | Up |
| *Kctd11* | Up | *Kndc1* | Up | *Gm42876* | Up |
| *Irf1* | Up | *Kng1* | Up | *Gm43088* | Up |
| *BC052040* | Up | *Knstrn* | Up | *Gm34866* | Up |
| *Ppa1* | Up | *Kntc1* | Up | *Gm43618* | Up |
| *Vstm4* | Up | *Krt12* | Up | *Gm24494* | Up |
| *Zfp367* | Up | *Krt14* | Up | *AI838599* | Up |
| *Clmp* | Up | *Krt16* | Up | *9430025C20Rik* | Up |
| *Ppp1r18* | Up | *Krt17* | Up | *Gm11827* | Up |
| *Siglec1* | Up | *Krt18* | Up | *Junos* | Up |
| *Hist1h2ac* | Up | *Krt19* | Up | *Gm17354* | Up |
| *Ptpn23* | Up | *Krt20* | Up | *Ttc39aos1* | Up |
| *Numbl* | Up | *Krt23* | Up | *Foxd2os* | Up |
| *Carhsp1* | Up | *Krt4* | Up | *4933421A08Rik* | Up |
| *D030056L22Rik* | Up | *Krt5* | Up | *Ftl2-ps* | Up |
| *Slc39a1* | Up | *Krt7* | Up | *Gm12940* | Up |
| *Lsp1* | Up | *Krt75* | Up | *Snora44* | Up |
| *Tuba1c* | Up | *Krt76* | Up | *Snora61* | Up |
| *Slc40a1* | Up | *Krt78* | Up | *2700016F22Rik* | Up |
| *Ms4a6b* | Up | *Krt79* | Up | *AI507597* | Up |
| *B3gnt3* | Up | *Krt8* | Up | *Gm13123* | Up |
| *Neurl3* | Up | *Krtap3-2* | Up | *Gm37795* | Up |
| *Tmem194* | Up | *Krtap4-16* | Up | *Gm37090* | Up |
| *Sh2d1b1* | Up | *Krtap5-4* | Up | *AI506816* | Up |
| *Gpatch4* | Up | *L3mbtl1* | Up | *Gm15587* | Up |
| *Ngf* | Up | *LOC100038947* | Up | *Abhd1* | Up |
| *Cd151* | Up | *LOC101056159* | Up | *Gm42798* | Up |
| *Gm13251* | Up | *LOC102634333* | Up | *Gm25767* | Up |
| *Btla* | Up | *LOC102635566* | Up | *Gm42858* | Up |
| *Cdk20* | Up | *LOC102639037* | Up | *Gm42614* | Up |
| *9130401M01Rik* | Up | *LOC102639700* | Up | *C230096K16Rik* | Up |
| *Gm4070* | Up | *LOC105244150* | Up | *Gm42732* | Up |
| *Mesdc1* | Up | *LOC105245043* | Up | *5430416N02Rik* | Up |
| *Serpinb9* | Up | *LOC105245453* | Up | *Gm27997* | Up |
| *Alg8* | Up | *LOC105246496* | Up | *Gm28030* | Up |
| *Pcolce2* | Up | *LOC105247125* | Up | *Gm27286* | Up |
| *Bst2* | Up | *LOC108167368* | Up | *Gm42793* | Up |
| *Mapkapk2* | Up | *LOC108167412* | Up | *Snord21* | Up |
| *Akap2* | Up | *LOC108168067* | Up | *Gm43118* | Up |
| *Exosc8* | Up | *LOC108168152* | Up | *Gm17122* | Up |
| *Adcy7* | Up | *LOC108168155* | Up | *Mir7028* | Up |
| *Cnn2* | Up | *LOC108168162* | Up | *Gm43637* | Up |
| *Uchl4* | Up | *LOC108168411* | Up | *Gm9754* | Up |
| *Ifi204* | Up | *LOC108168644* | Up | *Tbx3os1* | Up |
| *Zdhhc2* | Up | *LOC108168734* | Up | *2510016D11Rik* | Up |
| *1600002H07Rik* | Up | *LOC108168884* | Up | *Gm43813* | Up |
| *Trib1* | Up | *LOC108168925* | Up | *Gm37939* | Up |
| *Cd52* | Up | *LOC108169013* | Up | *Mir702* | Up |
| *Gpr157* | Up | *LOC108169021* | Up | *Gm36266* | Up |
| *AI607873* | Up | *LOC108169054* | Up | *B230303O12Rik* | Up |
| *Sars* | Up | *LOC108169093* | Up | *Gm34248* | Up |
| *Oas1a* | Up | *LOC108169150* | Up | *Gm15406* | Up |
| *Elf3* | Up | *LOC108169171* | Up | *Gm15411* | Up |
| *Tapbpl* | Up | *Lama5* | Up | *Gm43196* | Up |
| *Psmg3* | Up | *Lamb3* | Up | *Gm26809* | Up |
| *Tpbg* | Up | *Lamc1* | Up | *Gm20186* | Up |
| *Selplg* | Up | *Lamc2* | Up | *Gm13857* | Up |
| *Impdh2* | Up | *Lat2* | Up | *E230016M11Rik* | Up |
| *Pfn1* | Up | *Lbp* | Up | *Gm45053* | Up |
| *Nipal1* | Up | *Lcat* | Up | *1600020E01Rik* | Up |
| *Aplnr* | Up | *Lce1d* | Up | *Gm26636* | Up |
| *Lsm2* | Up | *Lce1e* | Up | *Gm44170* | Up |
| *Npm1* | Up | *Lce1f* | Up | *Gm15492* | Up |
| *Phlda1* | Up | *Lce1g* | Up | *Gm26982* | Up |
| *Hdx* | Up | *Lce3c* | Up | *Gm44103* | Up |
| *Car5a* | Down | *Lce3d* | Up | *Gm26728* | Up |
| *Cyp4f15* | Down | *Lce3f* | Up | *Gm26770* | Up |
| *Nrk* | Down | *Lce6a* | Up | *Gm15987* | Up |
| *Actc1* | Down | *Lcn2* | Up | *Gm15720* | Up |
| *Rspo2* | Down | *Lepr* | Up | *Gm15929* | Up |
| *Tbx20* | Down | *Lgals1* | Up | *Gm16175* | Up |
| *Alx4* | Down | *Lgals3* | Up | *Gm15567* | Up |
| *Slc6a2* | Down | *Lgals3bp* | Up | *Gm44641* | Up |
| *Adrb3* | Down | *Lgals7* | Up | *Gm44709* | Up |
| *Actn2* | Down | *Lgals9* | Up | *Tmem147os* | Up |
| *Nr2e3* | Down | *Lgi2* | Up | *RP24-63G4.14* | Up |
| *Pou2f3* | Down | *Lgi4* | Up | *Gm4673* | Up |
| *Serpina3c* | Down | *Lgsn* | Up | *Gm26790* | Up |
| *Orm1* | Down | *Lhfp* | Up | *Gm10109* | Up |
| *Gjb6* | Down | *Lhfpl2* | Up | *Snord35a* | Up |
| *Prok2* | Down | *Lhx2* | Up | *Gm22133* | Up |
| *Zcchc12* | Down | *Lhx4* | Up | *Gm10252* | Up |
| *Slc7a10* | Down | *Lhx5* | Up | *A030001D20Rik* | Up |
| *Tbx5* | Down | *Lhx9* | Up | *Gm45503* | Up |
| *Cox8b* | Down | *Lif* | Up | *Gm26856* | Up |
| *Adamdec1* | Down | *Lilr4b* | Up | *Gm44829* | Up |
| *Ucma* | Down | *Lilra6* | Up | *Gm27252* | Up |
| *Slit1* | Down | *Lilrb4a* | Up | *Gm45170* | Up |
| *Gm6878* | Down | *Liph* | Up | *Gm44751* | Up |
| *Pirt* | Down | *Litaf* | Up | *4632427E13Rik* | Up |
| *Cbln1* | Down | *Lmna* | Up | *Gm10603* | Up |
| *Ppapdc1a* | Down | *Lmnb1* | Up | *B430319F04Rik* | Up |
| *Dpysl4* | Down | *Lox* | Up | *AC122844.1* | Up |
| *Gpr33* | Down | *Loxl1* | Up | *Gm44876* | Up |
| *Mab21l2* | Down | *Loxl2* | Up | *AC136146.1* | Up |
| *Kcnt1* | Down | *Loxl3* | Up | *Gm28198* | Up |
| *Chrm5* | Down | *Loxl4* | Up | *9430064I24Rik* | Up |
| *Ccdc81* | Down | *Lpar1* | Up | *Gm44891* | Up |
| *Gm26616* | Down | *Lpcat2* | Up | *Kcnq1ot1* | Up |
| *Grm4* | Down | *Lpcat4* | Up | *Gm44732* | Up |
| *Pon1* | Down | *Lpin3* | Up | *Gm44785* | Up |
| *Fam47e* | Down | *Lrfn4* | Up | *Gm17491* | Up |
| *Npy5r* | Down | *Lrg1* | Up | *Gm26578* | Up |
| *Necab1* | Down | *Lrp1* | Up | *Gm42031* | Up |
| *Gm16181* | Down | *Lrp8* | Up | *Gm3235* | Up |
| *Map3k19* | Down | *Lrr1* | Up | *RP23-98C9.5* | Up |
| *Slitrk2* | Down | *Lrrc15* | Up | *Gm15889* | Up |
| *AI118078* | Down | *Lrrc20* | Up | *Gm15890* | Up |
| *Aldh3a1* | Down | *Lrrc25* | Up | *Gm45663* | Up |
| *Optc* | Down | *Lrrc32* | Up | *Ces2d-ps* | Up |
| *Eef1a2* | Down | *Lrrc59* | Up | *Gm16156* | Up |
| *Fam163b* | Down | *Lrrc73* | Up | *Gm15895* | Up |
| *4930447C04Rik* | Down | *Lrrc8e* | Up | *RP23-115O21.9* | Up |
| *Gm20661* | Down | *Lrrfip1* | Up | *RP23-312B17.3* | Up |
| *Bves* | Down | *Lrrn2* | Up | *AV064505* | Up |
| *Upk3a* | Down | *Lrrn4cl* | Up | *RP23-350M23.5* | Up |
| *Gm6327* | Down | *Lrrtm2* | Up | *RP23-110E20.5* | Up |
| *Apba2* | Down | *Lsmem1* | Up | *Gm37474* | Up |
| *Hemgn* | Down | *Lsr* | Up | *RP23-114G13.4* | Up |
| *Rgs22* | Down | *Ltb4r1* | Up | *RP23-114G13.1* | Up |
| *Nccrp1* | Down | *Ltb4r2* | Up | *RP23-162P10.8* | Up |
| *En2* | Down | *Ltbp2* | Up | *Gm10684* | Up |
| *Kcnmb3* | Down | *Ltc4s* | Up | *Gm10680* | Up |
| *Pin1rt1* | Down | *Lxn* | Up | *Plet1os* | Up |
| *Gucy2g* | Down | *Ly6c1* | Up | *RP23-320D23.6* | Up |
| *4930455H04Rik* | Down | *Ly6e* | Up | *RP23-297P22.1* | Up |
| *H2-M1* | Down | *Ly6f* | Up | *Gm26377* | Up |
| *Gm17482* | Down | *Ly6g* | Up | *Snhg5* | Up |
| *Klk10* | Down | *Ly6g6c* | Up | *RP23-368K22.5* | Up |
| *Cadps* | Down | *Ly6g6e* | Up | *4930524O07Rik* | Up |
| *Itih2* | Down | *Ly6i* | Up | *Gm17040* | Up |
| *Dyrk4* | Down | *Lypd1* | Up | *Gm17041* | Up |
| *Pomc* | Down | *Lypd3* | Up | *Gm37963* | Up |
| *Ppp3r2* | Down | *Lypd6b* | Up | *E530011L22Rik* | Up |
| *Gm5546* | Down | *Lyve1* | Up | *mt-Rnr1* | Up |
| *Tenm1* | Down | *Lyz1* | Up | *mt-Tv* | Up |
| *Fam178b* | Down | *Lyz2* | Up | *mt-Rnr2* | Up |
| *Rph3a* | Down | *Mab21l1* | Up | *mt-Tl1* | Up |
| *Otof* | Down | *Mab21l3* | Up | *mt-Tw* | Up |
| *Cdhr4* | Down | *Macc1* | Up | *mt-Ta* | Up |
| *Pax1* | Down | *Mad2l2* | Up | *mt-Tn* | Up |
| *Hs6st3* | Down | *Mafa* | Up | *mt-Tc* | Up |
| *Egf* | Down | *Maff* | Up | *mt-Tt* | Up |
| *Adprhl1* | Down | *Mafk* | Up | *Gm36995* | Up |
| *Gm10032* | Down | *Mak* | Up | *9530027J09Rik* | Up |
| *E230008N13Rik* | Down | *Mak16* | Up | *AW822252* | Up |
| *Tssk1* | Down | *Mal2* | Up | *Gm21986* | Up |
| *Dfnb59* | Down | *Mall* | Up | *Gm15241* | Up |
| *Tram1l1* | Down | *Mamstr* | Up | *Gm29650* | Up |
| *Fmod* | Down | *Man1c1* | Up | *Iyd* | Down |
| *Kcng1* | Down | *Map1a* | Up | *Ccdc170* | Down |
| *Mrgprh* | Down | *Map3k14* | Up | *Esr1* | Down |
| *Gfra3* | Down | *Map3k6* | Up | *Myct1* | Down |
| *1700011E24Rik* | Down | *Map3k8* | Up | *Epm2a* | Down |
| *Lrit3* | Down | *Map3k9* | Up | *Map7* | Down |
| *Slc5a4b* | Down | *Map4k4* | Up | *Tcf21* | Down |
| *A230052G05Rik* | Down | *Map6* | Up | *Enpp3* | Down |
| *Shisa9* | Down | *Map7d1* | Up | *Arhgap18* | Down |
| *2310016G11Rik* | Down | *Mapk11* | Up | *Lama2* | Down |
| *Acvr1c* | Down | *Mapk13* | Up | *Tspyl4* | Down |
| *Ralyl* | Down | *Mapk6* | Up | *Marcks* | Down |
| *Mep1b* | Down | *Mapkapk3* | Up | *Ak9* | Down |
| *Grik2* | Down | *Marcksl1* | Up | *Prdm1* | Down |
| *Col6a6* | Down | *Masp1* | Up | *Gm26741* | Down |
| *Cntn6* | Down | *Mast4* | Up | *Slc35f1* | Down |
| *Kcns1* | Down | *Mastl* | Up | *Fabp7* | Down |
| *Fbxw24* | Down | *Matn3* | Up | *Gcc2* | Down |
| *Gm21786* | Down | *Mb* | Up | *Chst3* | Down |
| *Dbh* | Down | *Mb21d1* | Up | *Adamts14* | Down |
| *Mapk8ip2* | Down | *Mboat1* | Up | *Gm5424* | Down |
| *Gm13298* | Down | *Mc2r* | Up | *Tet1* | Down |
| *Trim31* | Down | *Mc5r* | Up | *Dnajc12* | Down |
| *A4gnt* | Down | *Mcam* | Up | *Cdk1* | Down |
| *Vmn1r20* | Down | *Mcemp1* | Up | *Rab36* | Down |
| *Megf10* | Down | *Mcm3* | Up | *Lrrc75b* | Down |
| *Gpa33* | Down | *Mcm4* | Up | *Susd2* | Down |
| *Tmem207* | Down | *Mcm5* | Up | *Mmp11* | Down |
| *Gpr81* | Down | *Mcm7* | Up | *Zfp280b* | Down |
| *Dmrtc1a* | Down | *Mcmdc2* | Up | *Slc5a4a* | Down |
| *Tmem132c* | Down | *Mctp2* | Up | *Ybey* | Down |
| *C8a* | Down | *Mdfi* | Up | *Spatc1l* | Down |
| *Sgcz* | Down | *Mdga1* | Up | *Col6a2* | Down |
| *Cd209c* | Down | *Mdm2* | Up | *Col6a1* | Down |
| *Glt8d2* | Down | *Medag* | Up | *Gm10941* | Down |
| *Sprr2a2* | Down | *Mefv* | Up | *Gamt* | Down |
| *Grpr* | Down | *Megf11* | Up | *Tbxa2r* | Down |
| *Gm17349* | Down | *Meig1* | Up | *Smim24* | Down |
| *Vmn1r19* | Down | *Meiob* | Up | *1500009L16Rik* | Down |
| *Upk2* | Down | *Meis3* | Up | *Btbd11* | Down |
| *Myh6* | Down | *Melk* | Up | *Stab2* | Down |
| *Vpreb1* | Down | *Mesdc1* | Up | *Pah* | Down |
| *Dpp6* | Down | *Mesp2* | Up | *Igf1* | Down |
| *Myl1* | Down | *Mettl21a* | Up | *Slc5a8* | Down |
| *Elmod1* | Down | *Mex3a* | Up | *Ano4* | Down |
| *Lrfn2* | Down | *Mex3c* | Up | *Plxnc1* | Down |
| *Trdn* | Down | *Mfap5* | Up | *Alx1* | Down |
| *Ceacam19* | Down | *Mfge8* | Up | *Lrriq1* | Down |
| *Tmeff2* | Down | *Mfi2* | Up | *Lin7a* | Down |
| *S100b* | Down | *Mfsd10* | Up | *Osbpl8* | Down |
| *Mpped1* | Down | *Mfsd7b* | Up | *Cpm* | Down |
| *Klkb1* | Down | *Mgarp* | Up | *Ndufa4l2* | Down |
| *4930578G10Rik* | Down | *Mgat4c* | Up | *Shmt2* | Down |
| *Smcp* | Down | *Mgl2* | Up | *Rdh16* | Down |
| *Slc7a13* | Down | *Mgmt* | Up | *BC089597* | Down |
| *Ccdc37* | Down | *Mgp* | Up | *Osbp2* | Down |
| *Abca6* | Down | *Mical2* | Up | *Sec14l3* | Down |
| *Myo3a* | Down | *Micall1* | Up | *Nefh* | Down |
| *Wnt11* | Down | *Micall2* | Up | *Emid1* | Down |
| *Lrtm1* | Down | *Mid1* | Up | *Abca13* | Down |
| *Alpk3* | Down | *Mid1ip1* | Up | *Gm6899* | Down |
| *2310042D19Rik* | Down | *Milr1* | Up | *Chac2* | Down |
| *Mgst2* | Down | *Mis18bp1* | Up | *Nsg2* | Down |
| *Ppp1r1b* | Down | *Mki67* | Up | *Pank3* | Down |
| *Corin* | Down | *Mlf1* | Up | *Hmmr* | Down |
| *Coch* | Down | *Mlkl* | Up | *C1qtnf2* | Down |
| *Upk1a* | Down | *Mlph* | Up | *Adam19* | Down |
| *A530016L24Rik* | Down | *Mmp10* | Up | *Trim7* | Down |
| *Sptssb* | Down | *Mmp12* | Up | *Olfr1393* | Down |
| *Popdc2* | Down | *Mmp14* | Up | *Col23a1* | Down |
| *Nobox* | Down | *Mmp15* | Up | *Tcf7* | Down |
| *Pnpla3* | Down | *Mmp19* | Up | *9530068E07Rik* | Down |
| *Slc22a7* | Down | *Mmp20* | Up | *Pdlim4* | Down |
| *Akr1c14* | Down | *Mmp24* | Up | *Gjc2* | Down |
| *BC049762* | Down | *Mmp27* | Up | *Lrrc48* | Down |
| *Cyp7b1* | Down | *Mmp3* | Up | *Ndel1* | Down |
| *Npy1r* | Down | *Mmp7* | Up | *Chd3* | Down |
| *Tnn* | Down | *Mmp8* | Up | *Gm17305* | Down |
| *Slc6a12* | Down | *Mmp9* | Up | *Mgl2* | Down |
| *Wfdc15b* | Down | *Mmrn1* | Up | *Nlrp1b* | Down |
| *Itm2a* | Down | *Mnda* | Up | *Wscd1* | Down |
| *Marc1* | Down | *Mns1* | Up | *Atp2a3* | Down |
| *Dusp15* | Down | *Mob3a* | Up | *Gsg2* | Down |
| *Fam107a* | Down | *Morf4l2* | Up | *Aspa* | Down |
| *Slc12a1* | Down | *Moxd1* | Up | *Hic1* | Down |
| *Hpd* | Down | *Mrap* | Up | *Serpinf2* | Down |
| *Mfrp* | Down | *Mrc2* | Up | *Pipox* | Down |
| *Ano4* | Down | *Mroh2a* | Up | *Car4* | Down |
| *Fam166b* | Down | *Mrpl33* | Up | *Brip1* | Down |
| *Slc22a26* | Down | *Ms4a10* | Up | *Ppm1e* | Down |
| *Gm26596* | Down | *Ms4a4a* | Up | *4-Sep* | Down |
| *Fmo3* | Down | *Ms4a4c* | Up | *Hlf* | Down |
| *Baiap3* | Down | *Ms4a4d* | Up | *Gpr179* | Down |
| *Uroc1* | Down | *Ms4a6b* | Up | *Ppp1r1b* | Down |
| *Apol6* | Down | *Ms4a6c* | Up | *Ikzf3* | Down |
| *Slco4c1* | Down | *Ms4a6d* | Up | *Top2a* | Down |
| *Gm17546* | Down | *Ms4a8a* | Up | *Krt10* | Down |
| *Cyp2c68* | Down | *Msh4* | Up | *Klhl11* | Down |
| *Slc22a19* | Down | *Msh5* | Up | *Tubg2* | Down |
| *Gm15409* | Down | *Msi1* | Up | *Mapt* | Down |
| *Kcnj13* | Down | *Msl3l2* | Up | *Mrc2* | Down |
| *Col23a1* | Down | *Msln* | Up | *Ace* | Down |
| *A930011G23Rik* | Down | *Mslnl* | Up | *Axin2* | Down |
| *Serpina1e* | Down | *Msn* | Up | *Abca9* | Down |
| *Greb1* | Down | *Msr1* | Up | *Map2k6* | Down |
| *Gm10787* | Down | *Mss51* | Up | *Slc9a3r1* | Down |
| *Pex5l* | Down | *Mst1* | Up | *Gm20708* | Down |
| *Ppp2r2b* | Down | *Mt1* | Up | *Tk1* | Down |
| *Gm20695* | Down | *Mt2* | Up | *Afmid* | Down |
| *Islr2* | Down | *Mt3* | Up | *Cbx2* | Down |
| *Colec11* | Down | *Mtfr2* | Up | *Fasn* | Down |
| *Itgbl1* | Down | *Mthfd1l* | Up | *Sectm1b* | Down |
| *Mettl7a2* | Down | *Mthfd2* | Up | *Adcy3* | Down |
| *Fer1l4* | Down | *Mtl5* | Up | *Rnf144a* | Down |
| *Pde6a* | Down | *Muc1* | Up | *Rsad2* | Down |
| *Scd1* | Down | *Muc13* | Up | *Colec11* | Down |
| *Slc22a13* | Down | *Muc15* | Up | *D630036H23Rik* | Down |
| *Upk1b* | Down | *Muc4* | Up | *Ispd* | Down |
| *Aox3* | Down | *Muc5ac* | Up | *Etv1* | Down |
| *Inmt* | Down | *Musk* | Up | *Coch* | Down |
| *Thrsp* | Down | *Mvp* | Up | *Slc25a21* | Down |
| *Gabbr2* | Down | *Mxd3* | Up | *Pole2* | Down |
| *Prss12* | Down | *Mxra7* | Up | *L2hgdh* | Down |
| *Hhip* | Down | *Myadm* | Up | *Frmd6* | Down |
| *1700055N04Rik* | Down | *Myb* | Up | *Mthfd1* | Down |
| *2010002M12Rik* | Down | *Mybl1* | Up | *Tmem229b* | Down |
| *Abca13* | Down | *Mybl2* | Up | *Papln* | Down |
| *Nalcn* | Down | *Mybpc2* | Up | *Aldh6a1* | Down |
| *Tbx18* | Down | *Myc* | Up | *Mfsd7c* | Down |
| *Rnf24* | Down | *Myd88* | Up | *Rps6ka5* | Down |
| *Vipr1* | Down | *Myh3* | Up | *9030617O03Rik* | Down |
| *Adamts20* | Down | *Myh9* | Up | *Ccdc88c* | Down |
| *Elavl3* | Down | *Myl12a* | Up | *Tc2n* | Down |
| *Calb1* | Down | *Myo1g* | Up | *Fbln5* | Down |
| *Ndrg4* | Down | *Myo1h* | Up | *Asb2* | Down |
| *Slc4a1* | Down | *Myof* | Up | *D430019H16Rik* | Down |
| *Rasal1* | Down | *Myoz2* | Up | *Ckb* | Down |
| *AI481877* | Down | *Myrip* | Up | *Kif26a* | Down |
| *Ttc6* | Down | *Nags* | Up | *Igha* | Down |
| *Scube2* | Down | *Naip2* | Up | *Ighm* | Down |
| *Plk5* | Down | *Naip5* | Up | *Cdca7l* | Down |
| *Gm711* | Down | *Nanos1* | Up | *Itgb8* | Down |
| *Thsd7b* | Down | *Nans* | Up | *Akr1c14* | Down |
| *Snap25* | Down | *Nap1l2* | Up | *Akr1c18* | Down |
| *Gm10766* | Down | *Nbl1* | Up | *Akr1c21* | Down |
| *Amdhd1* | Down | *Ncapg* | Up | *Idi1* | Down |
| *Slc22a3* | Down | *Ncapg2* | Up | *Chrm3* | Down |
| *Gucy2f* | Down | *Ncaph* | Up | *Ryr2* | Down |
| *Fxyd2* | Down | *Ncf1* | Up | *Gpr137b* | Down |
| *Higd1b* | Down | *Ncf2* | Up | *Nid1* | Down |
| *Slc22a27* | Down | *Ncf4* | Up | *Epdr1* | Down |
| *Gm973* | Down | *Ncmap* | Up | *Aoah* | Down |
| *Cytl1* | Down | *Ncs1* | Up | *Slc17a3* | Down |
| *Akr1c18* | Down | *Ndc80* | Up | *Cmah* | Down |
| *Rhox6* | Down | *Ndn* | Up | *D130043K22Rik* | Down |
| *Lrrc66* | Down | *Necab2* | Up | *Agtr1a* | Down |
| *C920021L13Rik* | Down | *Nectin2* | Up | *Bphl* | Down |
| *C1qtnf3* | Down | *Nectin4* | Up | *Fam217a* | Down |
| *Egfl6* | Down | *Nefm* | Up | *4933417A18Rik* | Down |
| *Trpm1* | Down | *Neil3* | Up | *Eci3* | Down |
| *Vtn* | Down | *Nek2* | Up | *Tpmt* | Down |
| *Hsd17b14* | Down | *Nek6* | Up | *Id4* | Down |
| *Cyp4f14* | Down | *Neto2* | Up | *Gm906* | Down |
| *Abca8a* | Down | *Neurl1a* | Up | *Gm8765* | Down |
| *AU023871* | Down | *Nfam1* | Up | *Cplx2* | Down |
| *Fndc1* | Down | *Nfasc* | Up | *4833439L19Rik* | Down |
| *Slc22a22* | Down | *Nfe2* | Up | *Unc5a* | Down |
| *9030619P08Rik* | Down | *Nfe2l2* | Up | *Slc34a1* | Down |
| *Slc5a8* | Down | *Nfe2l3* | Up | *Pfn3* | Down |
| *Wfdc16* | Down | *Nfil3* | Up | *Tifab* | Down |
| *Akr1d1* | Down | *Nfkb2* | Up | *Cxcl14* | Down |
| *Msmp* | Down | *Nfkbia* | Up | *Zfp369* | Down |
| *Ido2* | Down | *Nfkbid* | Up | *Zfp759* | Down |
| *Dnah11* | Down | *Nfkbie* | Up | *Srd5a1* | Down |
| *Snx31* | Down | *Nfkbiz* | Up | *Irx1* | Down |
| *Ankrd65* | Down | *Ngf* | Up | *Slc6a19* | Down |
| *Pkhd1l1* | Down | *Nhlh1* | Up | *Nkd2* | Down |
| *Prox1* | Down | *Nid1* | Up | *Tppp* | Down |
| *Sult1a1* | Down | *Ninj2* | Up | *Dhfr* | Down |
| *Trim29* | Down | *Nipal1* | Up | *Dmgdh* | Down |
| *Serpina1d* | Down | *Nipal4* | Up | *Arsb* | Down |
| *Rasgef1a* | Down | *Nkd2* | Up | *Hmgcr* | Down |
| *Cyp2j11* | Down | *Nkpd1* | Up | *Tmem174* | Down |
| *Aadat* | Down | *Nkx2-9* | Up | *Map1b* | Down |
| *Upp2* | Down | *Nlgn2* | Up | *Gtf2h2* | Down |
| *Bhmt* | Down | *Nlrp10* | Up | *Cenph* | Down |
| *Ogn* | Down | *Nlrp12* | Up | *Ccnb1* | Down |
| *Ctnna2* | Down | *Nlrp3* | Up | *Cd180* | Down |
| *Chrdl1* | Down | *Nnmt* | Up | *Cenpk* | Down |
| *Cnbd1* | Down | *Nod2* | Up | *Skiv2l2* | Down |
| *Ak4* | Down | *Nop56* | Up | *BC067074* | Down |
| *Bche* | Down | *Nop58* | Up | *Hmgcs1* | Down |
| *Gm21955* | Down | *Nos3* | Up | *Acox2* | Down |
| *Trim67* | Down | *Nostrin* | Up | *Fam107a* | Down |
| *Fads6* | Down | *Notch3* | Up | *Cfap70* | Down |
| *Sprr2a1* | Down | *Nov* | Up | *Erc2* | Down |
| *Pck1* | Down | *Noxo1* | Up | *Wnt5a* | Down |
| *Lingo4* | Down | *Npas2* | Up | *Wdfy4* | Down |
| *Etnppl* | Down | *Npdc1* | Up | *Ccser2* | Down |
| *Ppp1r1a* | Down | *Nppa* | Up | *Txndc16* | Down |
| *Nuggc* | Down | *Nppb* | Up | *Dlgap5* | Down |
| *Olfr1034* | Down | *Nppc* | Up | *3632451O06Rik* | Down |
| *Gm4767* | Down | *Nptx1* | Up | *Rnase6* | Down |
| *Lipo2* | Down | *Nptx2* | Up | *Slc7a8* | Down |
| *Cacnb4* | Down | *Nptxr* | Up | *A730061H03Rik* | Down |
| *Esrrb* | Down | *Npy* | Up | *Mipep* | Down |
| *Masp2* | Down | *Nr4a1* | Up | *Gm4131* | Down |
| *Gm11128* | Down | *Nradd* | Up | *Wdfy2* | Down |
| *Ces1g* | Down | *Nras* | Up | *Pbk* | Down |
| *Gpx6* | Down | *Nrcam* | Up | *Cdca2* | Down |
| *Gsdmc3* | Down | *Nrg1* | Up | *9930012K11Rik* | Down |
| *Scube1* | Down | *Nrip2* | Up | *Cysltr2* | Down |
| *Dnah3* | Down | *Nrip3* | Up | *Erich6b* | Down |
| *Klhl38* | Down | *Nrm* | Up | *Ccdc122* | Down |
| *Fabp3-ps1* | Down | *Nsl1* | Up | *Pcdh17* | Down |
| *Pcsk6* | Down | *Nt5c1a* | Up | *Diaph3* | Down |
| *Acot3* | Down | *Nt5dc3* | Up | *Klf12* | Down |
| *Lamc3* | Down | *Ntf5* | Up | *Slitrk6* | Down |
| *Fancd2os* | Down | *Ntm* | Up | *Gpc6* | Down |
| *Myzap* | Down | *Nudt17* | Up | *Pcca* | Down |
| *Art3* | Down | *Nuf2* | Up | *Tmtc4* | Down |
| *Rufy4* | Down | *Numbl* | Up | *Ghr* | Down |
| *G6pc* | Down | *Nup210l* | Up | *Oxct1* | Down |
| *Gpr34* | Down | *Nup62cl* | Up | *Egflam* | Down |
| *Apobec2* | Down | *Nupr1* | Up | *Lmbrd2* | Down |
| *Tmem100* | Down | *Nxnl2* | Up | *Ugt3a1* | Down |
| *Eci3* | Down | *Nxpe5* | Up | *Ugt3a2* | Down |
| *Apoc1* | Down | *Nyx* | Up | *Prlr* | Down |
| *Adcy2* | Down | *Oacyl* | Up | *Gm21973* | Down |
| *Tsc22d3* | Down | *Oas1a* | Up | *Agxt2* | Down |
| *Tbx10* | Down | *Oas1c* | Up | *C1qtnf3* | Down |
| *Reln* | Down | *Oas1g* | Up | *Amacr* | Down |
| *Cd163* | Down | *Oas2* | Up | *Adamts12* | Down |
| *Itih5* | Down | *Oas3* | Up | *Npr3* | Down |
| *Edar* | Down | *Oasl1* | Up | *Pdzd2* | Down |
| *5830473C10Rik* | Down | *Oasl2* | Up | *Trio* | Down |
| *Slc16a7* | Down | *Oip5* | Up | *6-Mar* | Down |
| *Omd* | Down | *Oit1* | Up | *Tspyl5* | Down |
| *Slc16a4* | Down | *Olfr1033* | Up | *Snx31* | Down |
| *Cml1* | Down | *Olfr1442* | Up | *Rrm2b* | Down |
| *Ranbp3l* | Down | *Olfr920* | Up | *Angpt1* | Down |
| *Zcchc16* | Down | *Olr1* | Up | *Trps1* | Down |
| *Slc2a4* | Down | *Omp* | Up | *Dscc1* | Down |
| *Mfsd4* | Down | *Onecut2* | Up | *Col14a1* | Down |
| *Bdh1* | Down | *Opn3* | Up | *Slc22a22* | Down |
| *Proz* | Down | *Orai2* | Up | *Atad2* | Down |
| *Atp1a2* | Down | *Orc1* | Up | *Anxa13* | Down |
| *Epor* | Down | *Orm1* | Up | *Psca* | Down |
| *Spr-ps1* | Down | *Orm2* | Up | *Apol7e* | Down |
| *Tarm1* | Down | *Orm3* | Up | *Pvalb* | Down |
| *Ttc36* | Down | *Osm* | Up | *Kdelr3* | Down |
| *Aldoc* | Down | *Osmr* | Up | *Mgat3* | Down |
| *Bsnd* | Down | *Ovgp1* | Up | *Grap2* | Down |
| *4732465J04Rik* | Down | *Oxct2b* | Up | *Cyp2d12* | Down |
| *Acacb* | Down | *Oxtr* | Up | *Mpped1* | Down |
| *Rorc* | Down | *P2ry12* | Up | *Miox* | Down |
| *Gm10300* | Down | *P2ry13* | Up | *Kif21a* | Down |
| *Aspn* | Down | *P2ry2* | Up | *Slc2a13* | Down |
| *Rapsn* | Down | *Pabpc1l* | Up | *Lrrk2* | Down |
| *Matn4* | Down | *Padi1* | Up | *Cntn1* | Down |
| *Tnfrsf11b* | Down | *Padi4* | Up | *Methig1* | Down |
| *N28178* | Down | *Pak3* | Up | *Cela1* | Down |
| *Chst9* | Down | *Palmd* | Up | *Csad* | Down |
| *Aqp4* | Down | *Panx1* | Up | *Adcy9* | Down |
| *Cxcl12* | Down | *Pappa* | Up | *Rbfox1* | Down |
| *Gys2* | Down | *Parm1* | Up | *Abat* | Down |
| *Lpl* | Down | *Parp14* | Up | *Atf7ip2* | Down |
| *D630036H23Rik* | Down | *Parpbp* | Up | *Tekt5* | Down |
| *Lrrc31* | Down | *Pask* | Up | *Ciita* | Down |
| *Acy3* | Down | *Pax9* | Up | *Snx29* | Down |
| *Rp1* | Down | *Pbk* | Up | *Mpv17l* | Down |
| *Mfsd7c* | Down | *Pbp2* | Up | *Snai2* | Down |
| *Prlr* | Down | *Pbx4* | Up | *Vps8* | Down |
| *Hsd3b4* | Down | *Pcdh9* | Up | *Etv5* | Down |
| *Cyp2j7-ps* | Down | *Pcdhb10* | Up | *Bdh1* | Down |
| *Ces1d* | Down | *Pcsk1n* | Up | *Slc51a* | Down |
| *Higd1c* | Down | *Pcyt1b* | Up | *Iqcg* | Down |
| *C1qtnf2* | Down | *Pdcd1* | Up | *Mylk* | Down |
| *Rasgef1b* | Down | *Pdcd1lg2* | Up | *Eaf2* | Down |
| *Miox* | Down | *Pde1c* | Up | *Fbxo40* | Down |
| *Usp2* | Down | *Pde3b* | Up | *Polq* | Down |
| *Cyp51* | Down | *Pdgfra* | Up | *Stxbp5l* | Down |
| *Agmat* | Down | *Pdgfrb* | Up | *Zbtb20* | Down |
| *Acy1* | Down | *Pdk4* | Up | *Usf3* | Down |
| *Gstm7* | Down | *Pdlim1* | Up | *Ccdc80* | Down |
| *Abca9* | Down | *Pdlim3* | Up | *Zpld1* | Down |
| *Car14* | Down | *Pdlim7* | Up | *Nxpe3* | Down |
| *Thbs4* | Down | *Pdpn* | Up | *Abi3bp* | Down |
| *Abcc9* | Down | *Pea15a* | Up | *Epha3* | Down |
| *Atp4a* | Down | *Peak1* | Up | *Robo1* | Down |
| *Asb11* | Down | *Peg12* | Up | *Robo2* | Down |
| *Kcnn3* | Down | *Perp* | Up | *Adamts5* | Down |
| *Cndp1* | Down | *Pf4* | Up | *Clic6* | Down |
| *Pm20d1* | Down | *Pfkfb1* | Up | *Sh3bgr* | Down |
| *Trim61* | Down | *Pfkfb3* | Up | *B3galt5* | Down |
| *Coro6* | Down | *Pfkp* | Up | *Rps6ka2* | Down |
| *Sbspon* | Down | *Pgf* | Up | *Fndc1* | Down |
| *Pdzrn4* | Down | *Pglyrp1* | Up | *Crebrf* | Down |
| *Efcc1* | Down | *Pgm1* | Up | *Fgd2* | Down |
| *Dpys* | Down | *Phc2* | Up | *Cbs* | Down |
| *Lrrc17* | Down | *Phf19* | Up | *Hsf2bp* | Down |
| *Frmpd4* | Down | *Phgdh* | Up | *Zfp871* | Down |
| *Nap1l5* | Down | *Phlda1* | Up | *Zfp955a* | Down |
| *Clcnka* | Down | *Phlda3* | Up | *Zfp955b* | Down |
| *Gm14446* | Down | *Phox2a* | Up | *Zfp101* | Down |
| *Smim5* | Down | *Pi15* | Up | *H2-Ab1* | Down |
| *Mpv17l* | Down | *Pidd1* | Up | *H2-Aa* | Down |
| *Col19a1* | Down | *Piezo1* | Up | *H2-Eb1* | Down |
| *Gpr112* | Down | *Pif1* | Up | *Ppt2* | Down |
| *Acsm3* | Down | *Pik3r5* | Up | *Apom* | Down |
| *Amacr* | Down | *Pilra* | Up | *H2-M2* | Down |
| *Klhl3* | Down | *Pilrb1* | Up | *Mut* | Down |
| *Dlgap1* | Down | *Pilrb2* | Up | *Mep1a* | Down |
| *Cetn4* | Down | *Pim1* | Up | *Slc22a7* | Down |
| *Henmt1* | Down | *Pin1rt1* | Up | *Ttbk1* | Down |
| *Mkx* | Down | *Pinlyp* | Up | *Daam2* | Down |
| *Serpinf1* | Down | *Pip5kl1* | Up | *Sult1c2* | Down |
| *Fcamr* | Down | *Pira1* | Up | *Chaf1a* | Down |
| *Hif3a* | Down | *Pira2* | Up | *Ndc80* | Down |
| *Cd164l2* | Down | *Pirb* | Up | *Clip4* | Down |
| *Gsdmc2* | Down | *Pitpnm1* | Up | *Lbh* | Down |
| *Slc25a47* | Down | *Piwil2* | Up | *Ehd3* | Down |
| *Cntfr* | Down | *Piwil4* | Up | *Nlrc4* | Down |
| *Dnajc12* | Down | *Pkdrej* | Up | *Ltbp1* | Down |
| *Sfrp1* | Down | *Pkmyt1* | Up | *Rasgrp3* | Down |
| *Sec14l3* | Down | *Pkn3* | Up | *Cdc42ep3* | Down |
| *Tmem25* | Down | *Pla2g2e* | Up | *Galm* | Down |
| *Rap1gap* | Down | *Pla2g4e* | Up | *Slc8a1* | Down |
| *Cyp27b1* | Down | *Plac8* | Up | *Gm11096* | Down |
| *Gli2* | Down | *Plat* | Up | *Haao* | Down |
| *Id4* | Down | *Plaur* | Up | *Slc3a1* | Down |
| *Astn1* | Down | *Plb1* | Up | *Colec12* | Down |
| *Lum* | Down | *Plcd4* | Up | *Greb1l* | Down |
| *Rgs9* | Down | *Plcxd2* | Up | *Abhd3* | Down |
| *Tsku* | Down | *Pld2* | Up | *Ttc39c* | Down |
| *Cbr2* | Down | *Plec* | Up | *Chst9* | Down |
| *Wnt9b* | Down | *Plek* | Up | *Ttr* | Down |
| *Macrod2* | Down | *Plekha4* | Up | *Mep1b* | Down |
| *Smarca2* | Down | *Plekhm3* | Up | *Asxl3* | Down |
| *Dcst1* | Down | *Plekhn1* | Up | *Gypc* | Down |
| *Aspdh* | Down | *Plekhs1* | Up | *Stard4* | Down |
| *Xpnpep2* | Down | *Plet1* | Up | *Nrep* | Down |
| *Slc25a42* | Down | *Plin2* | Up | *Pcdh12* | Down |
| *Gm4450* | Down | *Plin4* | Up | *Fgf1* | Down |
| *Cyp2d26* | Down | *Plk1* | Up | *Nr3c1* | Down |
| *Negr1* | Down | *Plk2* | Up | *Dpysl3* | Down |
| *Rapgefl1* | Down | *Plk3* | Up | *Tnfaip8* | Down |
| *Shd* | Down | *Plk4* | Up | *F830016B08Rik* | Down |
| *Gyk* | Down | *Plod2* | Up | *Cd74* | Down |
| *Ass1* | Down | *Plp2* | Up | *Camk2a* | Down |
| *Afm* | Down | *Plpp5* | Up | *Pde6a* | Down |
| *Pxmp2* | Down | *Plscr1* | Up | *Afap1l1* | Down |
| *Ttyh1* | Down | *Plxna1* | Up | *Apcdd1* | Down |
| *Irx1* | Down | *Plxna2* | Up | *Piezo2* | Down |
| *Aacs* | Down | *Pmaip1* | Up | *Myo5b* | Down |
| *Ces1e* | Down | *Pmm1* | Up | *Pstpip2* | Down |
| *Plch2* | Down | *Pmp22* | Up | *Setbp1* | Down |
| *Vwa1* | Down | *Pnldc1* | Up | *Cndp1* | Down |
| *Scara5* | Down | *Pnma2* | Up | *Cndp2* | Down |
| *Htr1b* | Down | *Pnp2* | Up | *Aldh3b2* | Down |
| *Ntrk2* | Down | *Pold4* | Up | *Acy3* | Down |
| *Fam65c* | Down | *Pole* | Up | *Tbx10* | Down |
| *Cdh19* | Down | *Polk* | Up | *Ptprcap* | Down |
| *Cntd1* | Down | *Polq* | Up | *Tbc1d10c* | Down |
| *Gm10384* | Down | *Pom121l2* | Up | *Pygm* | Down |
| *Kbtbd11* | Down | *Popdc2* | Up | *Flrt1* | Down |
| *Acox2* | Down | *Popdc3* | Up | *Slc22a19* | Down |
| *Cox7a1* | Down | *Pou3f1* | Up | *Slc22a26* | Down |
| *Gpm6a* | Down | *Pou5f2* | Up | *Slc22a28* | Down |
| *Hlf* | Down | *Ppbp* | Up | *Slc22a30* | Down |
| *Tyw3* | Down | *Ppfia3* | Up | *Slc22a8* | Down |
| *Nog* | Down | *Ppl* | Up | *Fads2* | Down |
| *Myo5a* | Down | *Ppm1j* | Up | *Vwce* | Down |
| *Mreg* | Down | *Ppp1r13l* | Up | *Ms4a4b* | Down |
| *Wscd2* | Down | *Ppp1r14b* | Up | *Gm4952* | Down |
| *Esr1* | Down | *Ppp1r14c* | Up | *Keg1* | Down |
| *Cnn1* | Down | *Ppp1r15a* | Up | *Gna14* | Down |
| *Napsa* | Down | *Ppp1r18* | Up | *Gcnt1* | Down |
| *Csad* | Down | *Ppp1r32* | Up | *Nmrk1* | Down |
| *Nlrp6* | Down | *Ppp1r36* | Up | *Apba1* | Down |
| *Cbs* | Down | *Ppp1r3a* | Up | *Smarca2* | Down |
| *Csf1r* | Down | *Ppp1r3b* | Up | *Slc1a1* | Down |
| *Galnt11* | Down | *Ppp1r42* | Up | *4430402I18Rik* | Down |
| *Nrap* | Down | *Ppp2r2c* | Up | *A1cf* | Down |
| *Slc9a8* | Down | *Pqlc3* | Up | *Papss2* | Down |
| *Slc39a8* | Down | *Praf2* | Up | *Lipo3* | Down |
| *Scn4b* | Down | *Pram1* | Up | *Pank1* | Down |
| *Cntnap5a* | Down | *Prc1* | Up | *Kif20b* | Down |
| *Slc26a7* | Down | *Prdm13* | Up | *Cpeb3* | Down |
| *Scnn1a* | Down | *Prelid2* | Up | *Kif11* | Down |
| *Aspg* | Down | *Prg4* | Up | *Cep55* | Down |
| *Nxpe3* | Down | *Prkcdbp* | Up | *Lcor* | Down |
| *Kcnj1* | Down | *Prkx* | Up | *4933411K16Rik* | Down |
| *Ndrg2* | Down | *Prl3d1* | Up | *Abcc2* | Down |
| *Tspan13* | Down | *Procr* | Up | *Cyp2c44* | Down |
| *Dhtkd1* | Down | *Pros1* | Up | *Erlin1* | Down |
| *Lsamp* | Down | *Proser2* | Up | *Scd1* | Down |
| *Nudt19* | Down | *Prph* | Up | *Sh3pxd2a* | Down |
| *Sgk1* | Down | *Prr11* | Up | *Add3* | Down |
| *Slc2a5* | Down | *Prr13* | Up | *Nrap* | Down |
| *Cwh43* | Down | *Prr15* | Up | *Vwa2* | Down |
| *Gamt* | Down | *Prr7* | Up | *Rab11fip2* | Down |
| *Ugt3a1* | Down | *Prrg4* | Up | *Lypla1* | Down |
| *Insrr* | Down | *Prss22* | Up | *Gm37988* | Down |
| *Hsd17b11* | Down | *Prss37* | Up | *Adhfe1* | Down |
| *Bhmt2* | Down | *Prss56* | Up | *Ncoa2* | Down |
| *Oxgr1* | Down | *Prtn3* | Up | *Lactb2* | Down |
| *Pdgfrl* | Down | *Prune2* | Up | *Crispld1* | Down |
| *Akr1c21* | Down | *Prx* | Up | *Col19a1* | Down |
| *Kcna6* | Down | *Psat1* | Up | *Plekhb2* | Down |
| *Rhcg* | Down | *Psd4* | Up | *Aff3* | Down |
| *Ttr* | Down | *Psg16* | Up | *Fhl2* | Down |
| *Gfra1* | Down | *Psg18* | Up | *Gulp1* | Down |
| *Slc22a30* | Down | *Psg29* | Up | *Col3a1* | Down |
| *Cap2* | Down | *Psmb8* | Up | *Ccdc150* | Down |
| *Fmo2* | Down | *Psmd10* | Up | *Satb2* | Down |
| *Slc28a1* | Down | *Psors1c2* | Up | *Spats2l* | Down |
| *Fitm1* | Down | *Pspn* | Up | *Gm28845* | Down |
| *Hopx* | Down | *Psrc1* | Up | *D630023F18Rik* | Down |
| *Ovol1* | Down | *Ptbp3* | Up | *Fn1* | Down |
| *BC049352* | Down | *Ptcra* | Up | *Pecr* | Down |
| *Paqr7* | Down | *Ptgdr2* | Up | *Tmem169* | Down |
| *Chrna2* | Down | *Ptger2* | Up | *Igfbp5* | Down |
| *Prickle4* | Down | *Ptger4* | Up | *Col4a3* | Down |
| *Rhbg* | Down | *Ptgfrn* | Up | *Ngef* | Down |
| *Tmem52b* | Down | *Ptgs2* | Up | *Neu2* | Down |
| *6430571L13Rik* | Down | *Ptk7* | Up | *Col6a3* | Down |
| *Gm13318* | Down | *Ptp4a1* | Up | *Ramp1* | Down |
| *Fam214a* | Down | *Ptp4a2* | Up | *St8sia4* | Down |
| *Ybx2* | Down | *Ptp4a3* | Up | *Acmsd* | Down |
| *Adamtsl2* | Down | *Ptpn12* | Up | *Fcamr* | Down |
| *Grin3a* | Down | *Ptpn22* | Up | *Rab7b* | Down |
| *Ank2* | Down | *Ptpn23* | Up | *Prelp* | Down |
| *Acsm5* | Down | *Ptpn5* | Up | *Ppfia4* | Down |
| *Tldc2* | Down | *Ptpre* | Up | *Lmod1* | Down |
| *Gm5424* | Down | *Ptprh* | Up | *F13b* | Down |
| *Gm6665* | Down | *Ptprn* | Up | *BC026585* | Down |
| *Mogat2* | Down | *Ptrf* | Up | *Tnn* | Down |
| *Abca4* | Down | *Ptrh1* | Up | *Fmo1* | Down |
| *Hmcn2* | Down | *Pvr* | Up | *Fmo2* | Down |
| *Glt1d1* | Down | *Pxdc1* | Up | *Prrx1* | Down |
| *Sfrp2* | Down | *Pycr1* | Up | *F5* | Down |
| *Kcnab3* | Down | *Pyhin1* | Up | *Nuf2* | Down |
| *Hykk* | Down | *Qsox1* | Up | *Nos1ap* | Down |
| *Cyp2d12* | Down | *Rab15* | Up | *Tstd1* | Down |
| *Clnk* | Down | *Rab31* | Up | *Atp1a2* | Down |
| *Zim1* | Down | *Rab3d* | Up | *Cfap45* | Down |
| *Gm9967* | Down | *Rab44* | Up | *Coq8a* | Down |
| *Casr* | Down | *Racgap1* | Up | *Ephx1* | Down |
| *Slc10a5* | Down | *Rad18* | Up | *Lbr* | Down |
| *Tmem163* | Down | *Rad51* | Up | *Bpnt1* | Down |
| *Homer2* | Down | *Rad51ap1* | Up | *Tgfb2* | Down |
| *Atp2b2* | Down | *Rad54b* | Up | *Kcnk2* | Down |
| *Cry1* | Down | *Rad54l* | Up | *Cenpf* | Down |
| *Gnmt* | Down | *Raet1d* | Up | *Ccdc3* | Down |
| *Slc25a35* | Down | *Raet1e* | Up | *Dhtkd1* | Down |
| *Atp6v1b1* | Down | *Rap2b* | Up | *Itih2* | Down |
| *Tshr* | Down | *Raph1* | Up | *Itih5* | Down |
| *Slc6a20b* | Down | *Rasd1* | Up | *Itga8* | Down |
| *Slc13a2* | Down | *Rasip1* | Up | *Pter* | Down |
| *Pah* | Down | *Rasl10a* | Up | *Cubn* | Down |
| *4930556J24Rik* | Down | *Rasl10b* | Up | *Hnmt* | Down |
| *Spon1* | Down | *Rasl11a* | Up | *Slc34a3* | Down |
| *Zc2hc1c* | Down | *Rbm3* | Up | *Fam69b* | Down |
| *Tenm4* | Down | *Rbm38* | Up | *Sardh* | Down |
| *Lnx1* | Down | *Rbmy* | Up | *Col5a1* | Down |
| *Tlr11* | Down | *Rbp1* | Up | *Prrx2* | Down |
| *Tnfrsf19* | Down | *Rbp4* | Up | *Ass1* | Down |
| *Fa2h* | Down | *Rcan1* | Up | *Lamc3* | Down |
| *Ehhadh* | Down | *Rcn1* | Up | *Angptl2* | Down |
| *Mcoln3* | Down | *Rdh11* | Up | *Olfml2a* | Down |
| *Cyfip2* | Down | *Rdh12* | Up | *Scai* | Down |
| *Acss2* | Down | *Recql4* | Up | *Kynu* | Down |
| *Cct6b* | Down | *Reep2* | Up | *Zeb2* | Down |
| *A930039A15Rik* | Down | *Reg3g* | Up | *Csrnp3* | Down |
| *Mylk3* | Down | *Rel* | Up | *Lrp2* | Down |
| *Cmbl* | Down | *Rela* | Up | *Sp5* | Down |
| *Adamts15* | Down | *Relb* | Up | *Agps* | Down |
| *Stpg2* | Down | *Rell1* | Up | *Osbpl6* | Down |
| *Phyhip* | Down | *Relt* | Up | *Aplnr* | Down |
| *Adhfe1* | Down | *Rem2* | Up | *Mdk* | Down |
| *Kl* | Down | *Retnlg* | Up | *Mapk8ip1* | Down |
| *Col25a1* | Down | *Rgcc* | Up | *Lmo2* | Down |
| *Prelp* | Down | *Rgs1* | Up | *4931422A03Rik* | Down |
| *Dpep1* | Down | *Rgs16* | Up | *Mpped2* | Down |
| *Tmem86a* | Down | *Rgs19* | Up | *Slc12a6* | Down |
| *Rgs11* | Down | *Rgs20* | Up | *Gm21985* | Down |
| *Tc2n* | Down | *Rhbdf2* | Up | *Fsip1* | Down |
| *Fbln5* | Down | *Rhbdl2* | Up | *Bub1b* | Down |
| *Gpr179* | Down | *Rhbdl3* | Up | *Knl1* | Down |
| *Tcte2* | Down | *Rhob* | Up | *Nusap1* | Down |
| *Gldc* | Down | *Rhoc* | Up | *Ell3* | Down |
| *Slc16a9* | Down | *Rhod* | Up | *Casc4* | Down |
| *Kcnj10* | Down | *Rhoj* | Up | *Sord* | Down |
| *Pla1a* | Down | *Rhou* | Up | *Gatm* | Down |
| *Tnfaip8* | Down | *Rimbp3* | Up | *Slc12a1* | Down |
| *Pecr* | Down | *Rin1* | Up | *Gm9913* | Down |
| *Upk3b* | Down | *Ripk3* | Up | *Secisbp2l* | Down |
| *Vstm2a* | Down | *Rln1* | Up | *Usp8* | Down |
| *Aqp11* | Down | *Rmi2* | Up | *Itpripl1* | Down |
| *Tmem72* | Down | *Rnd1* | Up | *Kcnip3* | Down |
| *Kcnn1* | Down | *Rnd3* | Up | *Nphp1* | Down |
| *Kmo* | Down | *Rnf125* | Up | *Fbln7* | Down |
| *Akap5* | Down | *Rnf151* | Up | *Ckap2l* | Down |
| *Acadm* | Down | *Rnf19b* | Up | *Cpxm1* | Down |
| *Cck* | Down | *Rnf213* | Up | *Rnf24* | Down |
| *Kcnq3* | Down | *Rnf222* | Up | *Gm44637* | Down |
| *Papss2* | Down | *Rnf223* | Up | *Plcb1* | Down |
| *Capn13* | Down | *Rnf225* | Up | *Macrod2* | Down |
| *Cyp2f2* | Down | *Rnf39* | Up | *Bfsp1* | Down |
| *Armc2* | Down | *Rock2* | Up | *Zfp442* | Down |
| *1700040L02Rik* | Down | *Ros1* | Up | *Tpx2* | Down |
| *Gstm2* | Down | *Rpl12* | Up | *Dusp15* | Down |
| *Glul* | Down | *Rpl36a* | Up | *Tspyl3* | Down |
| *Wdr86* | Down | *Rpp25* | Up | *Ggt7* | Down |
| *Nkx3-1* | Down | *Rprm* | Up | *Acss2* | Down |
| *Pank1* | Down | *Rps12* | Up | *Myh7b* | Down |
| *Rpgrip1* | Down | *Rps27l* | Up | *Myl9* | Down |
| *Ryr2* | Down | *Rptn* | Up | *Rbl1* | Down |
| *Cdc42ep3* | Down | *Rrad* | Up | *Ppp1r16b* | Down |
| *Abhd15* | Down | *Rras* | Up | *Wfdc16* | Down |
| *Slc38a3* | Down | *Rras2* | Up | *Ube2c* | Down |
| *Anxa8* | Down | *Rrm2* | Up | *Slc9a8* | Down |
| *Ccdc3* | Down | *Rs1* | Up | *Fam65c* | Down |
| *Dio1* | Down | *Rsph4a* | Up | *Kcng1* | Down |
| *Hs6st2* | Down | *Rtn4* | Up | *Chrna4* | Down |
| *Lrrc34* | Down | *Rtn4rl2* | Up | *Hnf4g* | Down |
| *Notum* | Down | *Rtp4* | Up | *Fabp5* | Down |
| *Atp2a3* | Down | *Runx1* | Up | *Slc10a5* | Down |
| *Nat8* | Down | *Rusc2* | Up | *Slc7a12* | Down |
| *Zmynd10* | Down | *Ryr1* | Up | *Car3* | Down |
| *Arhgef39* | Down | *S100a10* | Up | *Ect2* | Down |
| *Atp6v1c2* | Down | *S100a11* | Up | *Nceh1* | Down |
| *Ces1f* | Down | *S100a14* | Up | *Tnik* | Down |
| *Acox3* | Down | *S100a2* | Up | *Pex5l* | Down |
| *Irs3* | Down | *S100a3* | Up | *Mccc1* | Down |
| *Car4* | Down | *S100a4* | Up | *Acad9* | Down |
| *Klf15* | Down | *S100a6* | Up | *Plk4* | Down |
| *Hrg* | Down | *S100a8* | Up | *Pcdh18* | Down |
| *Lpin1* | Down | *S100a9* | Up | *Postn* | Down |
| *Park2* | Down | *S1pr1* | Up | *Aadac* | Down |
| *Aspa* | Down | *S1pr2* | Up | *Sucnr1* | Down |
| *Axin2* | Down | *Saa1* | Up | *P2ry1* | Down |
| *Edn3* | Down | *Saa2* | Up | *Mme* | Down |
| *Mycbpap* | Down | *Saa3* | Up | *Mfsd1* | Down |
| *Abat* | Down | *Saa4* | Up | *B3galnt1* | Down |
| *Lypla1* | Down | *Sac3d1* | Up | *Fnip2* | Down |
| *Bco2* | Down | *Sacs* | Up | *Fam198b* | Down |
| *Myh11* | Down | *Samd14* | Up | *Pdgfc* | Down |
| *AA474408* | Down | *Samd4* | Up | *Gucy1a3* | Down |
| *Figf* | Down | *Samd5* | Up | *Lrat* | Down |
| *Fmo5* | Down | *Samsn1* | Up | *Iqgap3* | Down |
| *Fam195a* | Down | *Sapcd2* | Up | *Dcst1* | Down |
| *Unc13c* | Down | *Sbno2* | Up | *Slc27a3* | Down |
| *Slc22a28* | Down | *Sbsn* | Up | *Smcp* | Down |
| *Sv2a* | Down | *Scara3* | Up | *Selenbp2* | Down |
| *Sectm1b* | Down | *Scg5* | Up | *Sema6c* | Down |
| *Sptbn2* | Down | *Schip1* | Up | *Car14* | Down |
| *Eps8l1* | Down | *Scin* | Up | *Ankrd35* | Down |
| *Col27a1* | Down | *Scn1b* | Up | *Cd160* | Down |
| *Slc5a11* | Down | *Scn3a* | Up | *Pdzk1* | Down |
| *Slc16a5* | Down | *Scn3b* | Up | *Gpr89* | Down |
| *Myrip* | Down | *Scn7a* | Up | *Fmo5* | Down |
| *Nov* | Down | *Sct* | Up | *Zfp697* | Down |
| *Fbxo40* | Down | *Scube1* | Up | *Gm4450* | Down |
| *Tbxa2r* | Down | *Sdc1* | Up | *Hsd3b4* | Down |
| *Sord* | Down | *Sdcbp2* | Up | *Gm10681* | Down |
| *Sorcs3* | Down | *Sdk2* | Up | *Hsd3b2* | Down |
| *Chst3* | Down | *Sec1* | Up | *Hsd3b3* | Down |
| *Slc24a3* | Down | *Sec14l2* | Up | *Hao2* | Down |
| *Agps* | Down | *Sec14l4* | Up | *Cd2* | Down |
| *Wasf3* | Down | *Sec14l5* | Up | *Vangl1* | Down |
| *Pde4c* | Down | *Sele* | Up | *Kcnd3* | Down |
| *Ptger3* | Down | *Sell* | Up | *I830077J02Rik* | Down |
| *Gm8923* | Down | *Selp* | Up | *A630076J17Rik* | Down |
| *B3gat2* | Down | *Sema3a* | Up | *Prok1* | Down |
| *Crot* | Down | *Sema3c* | Up | *Slc16a4* | Down |
| *Zfp811* | Down | *Sema3d* | Up | *Gstm4* | Down |
| *C330021F23Rik* | Down | *Sema3e* | Up | *Fam102b* | Down |
| *2410066E13Rik* | Down | *Sema3f* | Up | *Olfm3* | Down |
| *6820408C15Rik* | Down | *Sema4b* | Up | *Synpo2* | Down |
| *Irs1* | Down | *Sema4c* | Up | *Egf* | Down |
| *Sned1* | Down | *Sema4f* | Up | *Papss1* | Down |
| *Rasd2* | Down | *Sema6b* | Up | *Arhgef38* | Down |
| *Pbld2* | Down | *Sema7a* | Up | *Cenpe* | Down |
| *Amy1* | Down | *Sepn1* | Up | *Bank1* | Down |
| *Folr2* | Down | *7-Sep* | Up | *Unc5c* | Down |
| *Sypl2* | Down | *8-Sep* | Up | *Gbp2b* | Down |
| *Tmem117* | Down | *Serinc2* | Up | *Mcoln2* | Down |
| *Zfp879* | Down | *Serinc4* | Up | *Lpar3* | Down |
| *Ptpru* | Down | *Serp2* | Up | *Ak5* | Down |
| *Trpc3* | Down | *Serpina10* | Up | *St6galnac5* | Down |
| *Lctl* | Down | *Serpina1b* | Up | *Tyw3* | Down |
| *Tusc5* | Down | *Serpina3g* | Up | *Lrriq3* | Down |
| *Cdkl1* | Down | *Serpina3i* | Up | *Negr1* | Down |
| *Podn* | Down | *Serpina3m* | Up | *Ankrd13c* | Down |
| *Adcy6* | Down | *Serpina3n* | Up | *Triqk* | Down |
| *Haao* | Down | *Serpina6* | Up | *Runx1t1* | Down |
| *Upb1* | Down | *Serpina7* | Up | *Wwp1* | Down |
| *Cables1* | Down | *Serpinb10* | Up | *Slc7a13* | Down |
| *Ttc39c* | Down | *Serpinb1a* | Up | *Fut9* | Down |
| *Cpeb3* | Down | *Serpinb1b* | Up | *Aco1* | Down |
| *Fibin* | Down | *Serpinb1c* | Up | *Arhgef39* | Down |
| *Frem2* | Down | *Serpinb2* | Up | *Car9* | Down |
| *Wfdc1* | Down | *Serpinb5* | Up | *Reck* | Down |
| *Wscd1* | Down | *Serpinb6a* | Up | *Melk* | Down |
| *Synpo2l* | Down | *Serpinb6b* | Up | *Pax5* | Down |
| *Prok1* | Down | *Serpinb9* | Up | *Ccdc180* | Down |
| *Ipcef1* | Down | *Serpinb9b* | Up | *Col15a1* | Down |
| *Rec8* | Down | *Serpinb9e* | Up | *Smc2* | Down |
| *Wnk4* | Down | *Serpinb9f* | Up | *Svep1* | Down |
| *Khk* | Down | *Serpinb9g* | Up | *Ptgr1* | Down |
| *Slco2b1* | Down | *Serpine1* | Up | *Ptprd* | Down |
| *Gprc5b* | Down | *Serpine2* | Up | *Frem1* | Down |
| *Slc2a13* | Down | *Serping1* | Up | *Bnc2* | Down |
| *8430408G22Rik* | Down | *Serpinh1* | Up | *Cntln* | Down |
| *Bend5* | Down | *Sertad1* | Up | *Adamtsl1* | Down |
| *Hsd3b3* | Down | *Sesn2* | Up | *Cyp2j13* | Down |
| *Neu2* | Down | *Sfn* | Up | *Cyp2j11* | Down |
| *Bbox1* | Down | *Sftpd* | Up | *Cyp2j5* | Down |
| *S100g* | Down | *Sgol1* | Up | *Kank4* | Down |
| *Tox3* | Down | *Sgol2a* | Up | *Ak4* | Down |
| *Dmgdh* | Down | *Sgtb* | Up | *Dnajc6* | Down |
| *Aqp1* | Down | *Sh2b2* | Up | *C8a* | Down |
| *Cdo1* | Down | *Sh2d1b1* | Up | *Dhcr24* | Down |
| *Aifm3* | Down | *Sh2d4b* | Up | *Mroh7* | Down |
| *1810041L15Rik* | Down | *Sh2d5* | Up | *Fam151a* | Down |
| *Rdh16* | Down | *Sh2d6* | Up | *Podn* | Down |
| *Gcgr* | Down | *Sh3bgrl3* | Up | *Bend5* | Down |
| *Mep1a* | Down | *Sh3pxd2b* | Up | *Skint3* | Down |
| *Hgd* | Down | *Shc2* | Up | *Trabd2b* | Down |
| *Kcne4* | Down | *Shc4* | Up | *Cyp4b1* | Down |
| *Srpx* | Down | *Shcbp1* | Up | *Akr1a1* | Down |
| *Daam2* | Down | *Shisa4* | Up | *Mfsd2a* | Down |
| *Fam151a* | Down | *Shox2* | Up | *Mycl* | Down |
| *Ggct* | Down | *Siglec1* | Up | *Clspn* | Down |
| *Lgals4* | Down | *Siglece* | Up | *Gm853* | Down |
| *Sgk2* | Down | *Sirpb1b* | Up | *Sdc3* | Down |
| *Rhov* | Down | *Siva1* | Up | *Myom3* | Down |
| *Rab11fip4* | Down | *Six1* | Up | *E2f2* | Down |
| *Aqp3* | Down | *Ska1* | Up | *Tcea3* | Down |
| *Dhdh* | Down | *Ska3* | Up | *Pla2g5* | Down |
| *Tnnc1* | Down | *Sla* | Up | *Gm21969* | Down |
| *Ftcd* | Down | *Slc10a6* | Up | *Aldh4a1* | Down |
| *Slc34a1* | Down | *Slc11a1* | Up | *Mfap2* | Down |
| *Mgam* | Down | *Slc12a4* | Up | *Ubiad1* | Down |
| *Rdh5* | Down | *Slc14a2* | Up | *Mtor* | Down |
| *Mgll* | Down | *Slc15a3* | Up | *Slc2a5* | Down |
| *Atp7b* | Down | *Slc16a1* | Up | *Per3* | Down |
| *Mccc2* | Down | *Slc16a3* | Up | *Vwa1* | Down |
| *3110021N24Rik* | Down | *Slc16a6* | Up | *Ankrd65* | Down |
| *Prom2* | Down | *Slc19a2* | Up | *Mxra8* | Down |
| *Slc34a3* | Down | *Slc1a5* | Up | *Perm1* | Down |
| *Gm15217* | Down | *Slc20a1* | Up | *Ankib1* | Down |
| *Rtp3* | Down | *Slc22a15* | Up | *Cyp51* | Down |
| *Acad12* | Down | *Slc22a3* | Up | *Fzd1* | Down |
| *Kifc2* | Down | *Slc23a3* | Up | *Gsap* | Down |
| *Ptgds* | Down | *Slc25a2* | Up | *Wdr86* | Down |
| *Art4* | Down | *Slc25a22* | Up | *Galnt11* | Down |
| *Ctsf* | Down | *Slc25a24* | Up | *E130116L18Rik* | Down |
| *Smad9* | Down | *Slc26a9* | Up | *Cenpa* | Down |
| *AI661453* | Down | *Slc27a6* | Up | *Lrpap1* | Down |
| *Cyp2j13* | Down | *Slc2a1* | Up | *Ccdc96* | Down |
| *Rab3a* | Down | *Slc2a6* | Up | *Evc* | Down |
| *Scn7a* | Down | *Slc34a2* | Up | *Zfp518b* | Down |
| *Per3* | Down | *Slc35e4* | Up | *Lap3* | Down |
| *Gpc5* | Down | *Slc37a1* | Up | *Cckar* | Down |
| *Susd2* | Down | *Slc38a2* | Up | *Fam114a1* | Down |
| *Mme* | Down | *Slc39a14* | Up | *Rbm47* | Down |
| *Sim1* | Down | *Slc39a6* | Up | *Corin* | Down |
| *Pygl* | Down | *Slc43a1* | Up | *Lrrc66* | Down |
| *Kank4* | Down | *Slc43a3* | Up | *Ugt2b5* | Down |
| *Hmx2* | Down | *Slc4a11* | Up | *Ugt2b37* | Down |
| *Cntn5* | Down | *Slc5a5* | Up | *Ugt2b38* | Down |
| *Ropn1l* | Down | *Slc5a7* | Up | *Slc4a4* | Down |
| *Lactb2* | Down | *Slc6a18* | Up | *Afm* | Down |
| *Arhgef19* | Down | *Slc6a19os* | Up | *Cxcl9* | Down |
| *Aqp6* | Down | *Slc7a1* | Up | *Art3* | Down |
| *Rasgrp2* | Down | *Slc7a11* | Up | *Stbd1* | Down |
| *Ace* | Down | *Slc7a5* | Up | *Fras1* | Down |
| *Sowaha* | Down | *Slc7a6* | Up | *Arhgap24* | Down |
| *Rassf10* | Down | *Slc9b2* | Up | *Hsd17b11* | Down |
| *Sall3* | Down | *Slco1a5* | Up | *Abcg3* | Down |
| *Tef* | Down | *Slco1c1* | Up | *Gbp8* | Down |
| *Gchfr* | Down | *Slco2a1* | Up | *Gbp10* | Down |
| *Slc12a6* | Down | *Slco5a1* | Up | *Pole* | Down |
| *Arl4d* | Down | *Slfn1* | Up | *Chek2* | Down |
| *Cml3* | Down | *Slfn2* | Up | *Mvk* | Down |
| *Ecm1* | Down | *Slfn4* | Up | *Hnf1a* | Down |
| *Clcnkb* | Down | *Slfn9* | Up | *Cit* | Down |
| *Prodh* | Down | *Slfnl1* | Up | *Aacs* | Down |
| *Dnase1* | Down | *Slit3* | Up | *Por* | Down |
| *Acvr2b* | Down | *Slpi* | Up | *Cux1* | Down |
| *Sardh* | Down | *Smad1* | Up | *Elfn1* | Down |
| *Retsat* | Down | *Smc2* | Up | *Card11* | Down |
| *Ahcy* | Down | *Smim3* | Up | *Zfp853* | Down |
| *Hsd3b2* | Down | *Smoc2* | Up | *Wasf3* | Down |
| *Tmco3* | Down | *Smox* | Up | *Slc46a3* | Down |
| *Susd5* | Down | *Smpd3* | Up | *Kl* | Down |
| *Ivd* | Down | *Smpd5* | Up | *Samd9l* | Down |
| *Fgf9* | Down | *Smpdl3b* | Up | *Gng11* | Down |
| *Isoc2b* | Down | *Smtnl1* | Up | *Col1a2* | Down |
| *Plin5* | Down | *Smurf1* | Up | *Casd1* | Down |
| *Plekhd1* | Down | *Snai3* | Up | *Umad1* | Down |
| *Fbp1* | Down | *Sntg1* | Up | *Ahcyl2* | Down |
| *Per1* | Down | *Snx10* | Up | *Plxna4* | Down |
| *Robo3* | Down | *Snx20* | Up | *Slc13a4* | Down |
| *Nudt11* | Down | *Soat2* | Up | *Ptn* | Down |
| *Col20a1* | Down | *Socs1* | Up | *Akr1d1* | Down |
| *Adssl1* | Down | *Socs2* | Up | *Ttc26* | Down |
| *Cckar* | Down | *Socs3* | Up | *Hipk2* | Down |
| *Ces2h* | Down | *Sorcs2* | Up | *Tbxas1* | Down |
| *Inpp5j* | Down | *Sowahc* | Up | *Tpk1* | Down |
| *Slco1a1* | Down | *Sox11* | Up | *Gimap7* | Down |
| *Prkcq* | Down | *Sox15* | Up | *Tril* | Down |
| *BC030499* | Down | *Sox21* | Up | *Ggct* | Down |
| *St8sia1* | Down | *Sox4* | Up | *Inmt* | Down |
| *Cela1* | Down | *Sox9* | Up | *Herc3* | Down |
| *Tjp3* | Down | *Sp6* | Up | *Nap1l5* | Down |
| *Fam217a* | Down | *Sp8* | Up | *Igkc* | Down |
| *Kcnma1* | Down | *Spaca7* | Up | *Cd8b1* | Down |
| *Gnao1* | Down | *Sparc* | Up | *Ctnna2* | Down |
| *Kcnn2* | Down | *Spata18* | Up | *Nat8f1* | Down |
| *Adtrp* | Down | *Spata20* | Up | *Tprkb* | Down |
| *Slc37a4* | Down | *Spatc1* | Up | *Efcc1* | Down |
| *Adrb2* | Down | *Spcs3* | Up | *H1fx* | Down |
| *Slc51a* | Down | *Spdl1* | Up | *Podxl2* | Down |
| *Kcnj15* | Down | *Specc1* | Up | *Fancd2* | Down |
| *Gm5475* | Down | *Sphk1* | Up | *Fancd2os* | Down |
| *Tpmt* | Down | *Spin4* | Up | *Cand2* | Down |
| *Slc22a8* | Down | *Spink2* | Up | *Mbd4* | Down |
| *Slc15a2* | Down | *Spink8* | Up | *Ift122* | Down |
| *Slc6a13* | Down | *Spire2* | Up | *Alox5* | Down |
| *Acss3* | Down | *Spns2* | Up | *Cxcl12* | Down |
| *Gm4737* | Down | *Spock1* | Up | *Zfp239* | Down |
| *Stxbp4* | Down | *Spock2* | Up | *Wnt5b* | Down |
| *Gstz1* | Down | *Spon1* | Up | *B4galnt3* | Down |
| *Slc27a2* | Down | *Spon2* | Up | *D330020A13Rik* | Down |
| *C1qtnf7* | Down | *Spp1* | Up | *Cecr2* | Down |
| *B3galnt1* | Down | *Spred3* | Up | *Lrrc23* | Down |
| *Abhd14b* | Down | *Sprn* | Up | *Cdca3* | Down |
| *Idh2* | Down | *Sprr1a* | Up | *P3h3* | Down |
| *Tet2* | Down | *Sprr1b* | Up | *Cd4* | Down |
| *Myh14* | Down | *Sprr2d* | Up | *Cd27* | Down |
| *Tmem200b* | Down | *Sprr2e* | Up | *Ntf3* | Down |
| *Dcxr* | Down | *Sprr2f* | Up | *Rad51ap1* | Down |
| *Gpc6* | Down | *Sprr2h* | Up | *Gm44596* | Down |
| *Avpr2* | Down | *Sprr2i* | Up | *Pzp* | Down |
| *Fbxl7* | Down | *Spry1* | Up | *Clec2h* | Down |
| *Tmem221* | Down | *Spsb1* | Up | *Clec7a* | Down |
| *Acss1* | Down | *Spx* | Up | *Klrk1* | Down |
| *Acat1* | Down | *Srd5a1* | Up | *Smco3* | Down |
| *Aldh4a1* | Down | *Srgap1* | Up | *Pik3c2g* | Down |
| *Bcat2* | Down | *Srgn* | Up | *Slco1a1* | Down |
| *Eaf2* | Down | *Srrm4* | Up | *Gys2* | Down |
| *Ntf3* | Down | *Srsf12* | Up | *Abcc9* | Down |
| *Foxq1* | Down | *Srxn1* | Up | *St8sia1* | Down |
| *Ppargc1b* | Down | *Ssbp4* | Up | *Bcat1* | Down |
| *Klk1* | Down | *Ssc5d* | Up | *Sspn* | Down |
| *Hao2* | Down | *Sstr2* | Up | *Mansc4* | Down |
| *Fgf12* | Down | *Sstr4* | Up | *Gm36210* | Down |
| *Colgalt2* | Down | *St14* | Up | *Zfp551* | Down |
| *Dcn* | Down | *St3gal1* | Up | *Dact3* | Down |
| *Gm129* | Down | *St6gal1* | Up | *Ccdc8* | Down |
| *Ngef* | Down | *St8sia2* | Up | *Zfp526* | Down |
| *Tmem30b* | Down | *Stac2* | Up | *Ceacam2* | Down |
| *BC049715* | Down | *Stambpl1* | Up | *Erich4* | Down |
| *Ccbl2* | Down | *Stap2* | Up | *Ltbp4* | Down |
| *Stard8* | Down | *Stat3* | Up | *Ppp1r14a* | Down |
| *Shank2* | Down | *Steap1* | Up | *Cox7a1* | Down |
| *Mgat3* | Down | *Steap4* | Up | *Upk1a* | Down |
| *Cyp2e1* | Down | *Stfa1* | Up | *Clec11a* | Down |
| *Fam228a* | Down | *Stfa2l1* | Up | *Rcn3* | Down |
| *Tm7sf2* | Down | *Stil* | Up | *Dhdh* | Down |
| *Klk1b5* | Down | *Stk32c* | Up | *Dbp* | Down |
| *Isoc2a* | Down | *Stmn4* | Up | *Tmem86a* | Down |
| *Sh3d21* | Down | *Stra6l* | Up | *E2f8* | Down |
| *Pde4d* | Down | *Stx11* | Up | *Ano5* | Down |
| *Sema4g* | Down | *Styk1* | Up | *Gas2* | Down |
| *Srd5a2* | Down | *Sulf1* | Up | *Gabrb3* | Down |
| *Gm10647* | Down | *Sulf2* | Up | *Ndn* | Down |
| *Lipo1* | Down | *Sult1e1* | Up | *Pcsk6* | Down |
| *Cmah* | Down | *Sult2b1* | Up | *Aldh1a3* | Down |
| *Apom* | Down | *Sult4a1* | Up | *Prc1* | Down |
| *Acsm2* | Down | *Sult5a1* | Up | *Slc28a1* | Down |
| *Kif20b* | Down | *Susd1* | Up | *Nox4* | Down |
| *Kng2* | Down | *Svop* | Up | *Mogat2* | Down |
| *Kcnab1* | Down | *Sycp3* | Up | *Hbb-bt* | Down |
| *Tppp* | Down | *Syn1* | Up | *Hbb-bs* | Down |
| *Sptlc3* | Down | *Syndig1* | Up | *Olfml1* | Down |
| *Slc46a3* | Down | *Syne3* | Up | *Mrvi1* | Down |
| *Slc25a29* | Down | *Syngr3* | Up | *Far1* | Down |
| *Acadsb* | Down | *Synm* | Up | *Xylt1* | Down |
| *E130208F15Rik* | Down | *Syt12* | Up | *Acsm5* | Down |
| *Rarres2* | Down | *Syt2* | Up | *Acsm2* | Down |
| *Rgs7bp* | Down | *Syt5* | Up | *Acsm1* | Down |
| *Hpgd* | Down | *Syt8* | Up | *Acsm3* | Down |
| *Gas1* | Down | *Sytl1* | Up | *Slc5a11* | Down |
| *Sirt3* | Down | *Sytl5* | Up | *Itgax* | Down |
| *Tle2* | Down | *Tac1* | Up | *Adam12* | Down |
| *Gal3st1* | Down | *Tac2* | Up | *Mki67* | Down |
| *Nepn* | Down | *Tacc3* | Up | *Tnni2* | Down |
| *Ptgis* | Down | *Tacr3* | Up | *Lsp1* | Down |
| *Slc22a18* | Down | *Tacstd2* | Up | *Ano1* | Down |
| *Bphl* | Down | *Taf1d* | Up | *Shcbp1* | Down |
| *Pdk2* | Down | *Taf4b* | Up | *Slc10a2* | Down |
| *Gm16493* | Down | *Tagln* | Up | *Atp11a* | Down |
| *2210404O07Rik* | Down | *Tagln2* | Up | *Proz* | Down |
| *Fpgs* | Down | *Tap1* | Up | *Tmco3* | Down |
| *Adcy10* | Down | *Tas1r2* | Up | *Fbxo25* | Down |
| *Mertk* | Down | *Tas2r143* | Up | *Angpt2* | Down |
| *Slc12a3* | Down | *Tax1bp3* | Up | *Atp7b* | Down |
| *Tcp10b* | Down | *Tbc1d2* | Up | *Ckap2* | Down |
| *4430402I18Rik* | Down | *Tbc1d8* | Up | *Sfrp1* | Down |
| *Wnk1* | Down | *Tbpl2* | Up | *Enpp6* | Down |
| *Sult1d1* | Down | *Tbrg1* | Up | *Gpm6a* | Down |
| *Ugt3a2* | Down | *Tbx20* | Up | *Hpgd* | Down |
| *Sod2* | Down | *Tcaf2* | Up | *Sap30* | Down |
| *Serping1* | Down | *Tcf19* | Up | *Aadat* | Down |
| *Lepr* | Down | *Tcf23* | Up | *Csgalnact1* | Down |
| *Wnt5b* | Down | *Tcfl5* | Up | *Slc18a1* | Down |
| *Fah* | Down | *Tctex1d4* | Up | *Lzts1* | Down |
| *Fn3k* | Down | *Tead4* | Up | *Mcm5* | Down |
| *Srgap3* | Down | *Tecta* | Up | *Hhip* | Down |
| *Slc25a25* | Down | *Tekt2* | Up | *Mylk3* | Down |
| *Slc2a9* | Down | *Tert* | Up | *Itfg1* | Down |
| *Tmtc1* | Down | *Tes* | Up | *Nkd1* | Down |
| *Acaa2* | Down | *Tex10* | Up | *Mmp2* | Down |
| *Syt3* | Down | *Tex14* | Up | *Ces1d* | Down |
| *Efcab12* | Down | *Tex15* | Up | *Ces1e* | Down |
| *Pik3c2g* | Down | *Tex19.1* | Up | *Ces1f* | Down |
| *Dhrs3* | Down | *Tfap2a* | Up | *Ces1g* | Down |
| *A1cf* | Down | *Tg* | Up | *Cngb1* | Down |
| *Ces2b* | Down | *Tgfa* | Up | *Cdh11* | Down |
| *Fmo1* | Down | *Tgfb1* | Up | *Pdp2* | Down |
| *Plekha6* | Down | *Tgfb3* | Up | *Ces2b* | Down |
| *Adam22* | Down | *Tgfbi* | Up | *Ces2h* | Down |
| *Pfkm* | Down | *Tgfbr2* | Up | *Mtss1l* | Down |
| *Fam13a* | Down | *Tgif1* | Up | *Pdpr* | Down |
| *Anpep* | Down | *Tgm2* | Up | *Cdh13* | Down |
| *Ecm2* | Down | *Thbd* | Up | *Mvd* | Down |
| *Porcn* | Down | *Thbs1* | Up | *Gucy1a2* | Down |
| *Cdh13* | Down | *Them5* | Up | *Gria4* | Down |
| *Slc17a3* | Down | *Themis3* | Up | *Dync2h1* | Down |
| *Bmp15* | Down | *Thyn1* | Up | *Mmp12* | Down |
| *Vwa2* | Down | *Ticam2* | Up | *Cep126* | Down |
| *Lrp2* | Down | *Ticrr* | Up | *4931406C07Rik* | Down |
| *Slit2* | Down | *Tifa* | Up | *Ccdc67* | Down |
| *C1qtnf9* | Down | *Timeless* | Up | *Icam5* | Down |
| *Defb29* | Down | *Timp1* | Up | *Ldlr* | Down |
| *Adck5* | Down | *Tinagl1* | Up | *Ccdc151* | Down |
| *Tbc1d16* | Down | *Tirap* | Up | *Kcnj5* | Down |
| *Spink8* | Down | *Tjp2* | Up | *Clmp* | Down |
| *Susd3* | Down | *Tlcd2* | Up | *Thy1* | Down |
| *1700055D18Rik* | Down | *Tlr13* | Up | *Tmem25* | Down |
| *Syt7* | Down | *Tlr2* | Up | *Ttc36* | Down |
| *Keg1* | Down | *Tlr4* | Up | *Cd3g* | Down |
| *Apcdd1* | Down | *Tlr5* | Up | *Cd3d* | Down |
| *Pkd2l2* | Down | *Tlr6* | Up | *Il10ra* | Down |
| *Slc5a6* | Down | *Tlr8* | Up | *Bco2* | Down |
| *Efhd1* | Down | *Tm4sf20* | Up | *Zc3h12c* | Down |
| *Herpud1* | Down | *Tmbim1* | Up | *Acat1* | Down |
| *1700029J07Rik* | Down | *Tmcc3* | Up | *Hykk* | Down |
| *Cth* | Down | *Tmed5* | Up | *Cplx3* | Down |
| *Best3* | Down | *Tmeff1* | Up | *Islr* | Down |
| *Cep95* | Down | *Tmem100* | Up | *2810417H13Rik* | Down |
| *Zfp286* | Down | *Tmem119* | Up | *Dapk2* | Down |
| *Prdm16* | Down | *Tmem130* | Up | *Lactb* | Down |
| *Ninl* | Down | *Tmem132e* | Up | *Tln2* | Down |
| *P2rx1* | Down | *Tmem145* | Up | *Fam81a* | Down |
| *Dnajc28* | Down | *Tmem154* | Up | *Gm10642* | Down |
| *Pcsk4* | Down | *Tmem156* | Up | *Dyx1c1* | Down |
| *Emx1* | Down | *Tmem158* | Up | *Myo5a* | Down |
| *Ptprr* | Down | *Tmem171* | Up | *Gm17324* | Down |
| *Abcc6* | Down | *Tmem173* | Up | *Filip1* | Down |
| *Hnmt* | Down | *Tmem179* | Up | *Myo6* | Down |
| *Myom1* | Down | *Tmem184b* | Up | *Me1* | Down |
| *Afmid* | Down | *Tmem190* | Up | *Tbc1d2b* | Down |
| *Igfbp4* | Down | *Tmem196* | Up | *Slc9a9* | Down |
| *Cacnb2* | Down | *Tmem252* | Up | *Pcolce2* | Down |
| *Gramd1b* | Down | *Tmem253* | Up | *Pls1* | Down |
| *Tln2* | Down | *Tmem40* | Up | *Gk5* | Down |
| *Hsf2bp* | Down | *Tmem43* | Up | *Clstn2* | Down |
| *Lrriq3* | Down | *Tmem45a* | Up | *Rbp1* | Down |
| *Ckb* | Down | *Tmem59l* | Up | *A4gnt* | Down |
| *Zrsr1* | Down | *Tmem63c* | Up | *Dnajc13* | Down |
| *Pim3* | Down | *Tmem95* | Up | *Cpne4* | Down |
| *Apeh* | Down | *Tmem98* | Up | *Col6a6* | Down |
| *Aff3* | Down | *Tmprss11b* | Up | *Col6a4* | Down |
| *Rilp* | Down | *Tmprss11e* | Up | *Glyctk* | Down |
| *AA415398* | Down | *Tmprss11f* | Up | *Acy1* | Down |
| *Tenm2* | Down | *Tmprss4* | Up | *Zmynd10* | Down |
| *Tmod2* | Down | *Tmsb10* | Up | *Fam212a* | Down |
| *Chd5* | Down | *Tmsb4x* | Up | *Apeh* | Down |
| *Gm6614* | Down | *Tnc* | Up | *Pfkfb4* | Down |
| *Macrod1* | Down | *Tnf* | Up | *Rtp3* | Down |
| *Rgcc* | Down | *Tnfaip2* | Up | *Cmtm6* | Down |
| *Osbpl8* | Down | *Tnfaip3* | Up | *Vill* | Down |
| *Krt23* | Down | *Tnfaip6* | Up | *Cck* | Down |
| *Map2k6* | Down | *Tnfaip8l1* | Up | *Vipr1* | Down |
| *Slc22a5* | Down | *Tnfrsf10b* | Up | *Tcaim* | Down |
| *Abhd14a* | Down | *Tnfrsf12a* | Up | *Kif15* | Down |
| *Pnkd* | Down | *Tnfrsf18* | Up | *Slc6a20b* | Down |
| *Ubiad1* | Down | *Tnfrsf1a* | Up | *Ccr9* | Down |
| *Galnt14* | Down | *Tnfrsf1b* | Up | *Cxcr6* | Down |
| *C8g* | Down | *Tnfrsf22* | Up | *Ccr2* | Down |
| *Slc25a34* | Down | *Tnfrsf23* | Up | *Med14* | Down |
| *Slc52a3* | Down | *Tnfrsf9* | Up | *Gpr34* | Down |
| *Lrtm2* | Down | *Tnfsf13b* | Up | *Chst7* | Down |
| *F13b* | Down | *Tnfsf15* | Up | *Rhox6* | Down |
| *Pipox* | Down | *Tnfsf18* | Up | *Apln* | Down |
| *Angpt2* | Down | *Tnfsf8* | Up | *Xpnpep2* | Down |
| *Cry2* | Down | *Tnip1* | Up | *Hs6st2* | Down |
| *Glyctk* | Down | *Tnni1* | Up | *Smim10l2a* | Down |
| *Aldh8a1* | Down | *Tnnt2* | Up | *Adgrg4* | Down |
| *Izumo4* | Down | *Tomm20l* | Up | *Arhgef6* | Down |
| *Cd59b* | Down | *Tomt* | Up | *Nsdhl* | Down |
| *Snx29* | Down | *Top2a* | Up | *Dusp9* | Down |
| *Slc23a1* | Down | *Tor3a* | Up | *Gk* | Down |
| *Cnnm1* | Down | *Tpbg* | Up | *Pdk3* | Down |
| *Pik3c2g* | Down | *Tpm1* | Up | *Kif4* | Down |
| *Vdr* | Down | *Tpm2* | Up | *Rgag4* | Down |
| *Hmcn1* | Down | *Tpm3* | Up | *Slc16a2* | Down |
| *Tril* | Down | *Tpm4* | Up | *Cysltr1* | Down |
| *Hps5* | Down | *Tpx2* | Up | *Itm2a* | Down |
| *Cdnf* | Down | *Traf1* | Up | *Srpx2* | Down |
| *Gm872* | Down | *Traip* | Up | *Armcx6* | Down |
|  |  | *Trank1* | Up | *Gprasp2* | Down |
|  |  | *Trem1* | Up | *Frmpd3* | Down |
|  |  | *Trem2* | Up | *Nxt2* | Down |
|  |  | *Trem3* | Up | *Chrdl1* | Down |
|  |  | *Treml2* | Up | *Lrch2* | Down |
|  |  | *Treml4* | Up | *Alas2* | Down |
|  |  | *Trf* | Up | *Kctd12b* | Down |
|  |  | *Trib1* | Up | *Reps2* | Down |
|  |  | *Trib3* | Up | *Tmem27* | Down |
|  |  | *Trim10* | Up | *Ace2* | Down |
|  |  | *Trim15* | Up | *Egfl6* | Down |
|  |  | *Trim16* | Up | *RP23-331E5.8* | Down |
|  |  | *Trim30a* | Up | *Gm25596* | Down |
|  |  | *Trim30b* | Up | *Gm26564* | Down |
|  |  | *Trim30d* | Up | *1700094J05Rik* | Down |
|  |  | *Trim59* | Up | *Gm15343* | Down |
|  |  | *Trim6* | Up | *Gm16104* | Down |
|  |  | *Trim69* | Up | *A230060F14Rik* | Down |
|  |  | *Trim72* | Up | *Gm17028* | Down |
|  |  | *Trip13* | Up | *Gm15663* | Down |
|  |  | *Trnp1* | Up | *Gm11945* | Down |
|  |  | *Troap* | Up | *Gm16170* | Down |
|  |  | *Trp53* | Up | *Gm12256* | Down |
|  |  | *Trp53inp1* | Up | *AL603834.1* | Down |
|  |  | *Trp53tg5* | Up | *Slc13a2os* | Down |
|  |  | *Trpv2* | Up | *Adap2os* | Down |
|  |  | *Trpv6* | Up | *1700003D09Rik* | Down |
|  |  | *Tsc22d1* | Up | *Gm11940* | Down |
|  |  | *Tshr* | Up | *Mir8101* | Down |
|  |  | *Tspan11* | Up | *Gm20511* | Down |
|  |  | *Tspan17* | Up | *Gm11651* | Down |
|  |  | *Tspan8* | Up | *Taco1os* | Down |
|  |  | *Tssk4* | Up | *Gm26888* | Down |
|  |  | *Tssk6* | Up | *Gm11788* | Down |
|  |  | *Ttc16* | Up | *A630072L19Rik* | Down |
|  |  | *Ttc21a* | Up | *RP23-193M8.3* | Down |
|  |  | *Ttc22* | Up | *Gpr137b-ps* | Down |
|  |  | *Ttc39a* | Up | *A330033J07Rik* | Down |
|  |  | *Ttc39b* | Up | *1810034E14Rik* | Down |
|  |  | *Ttc9* | Up | *Gm15326* | Down |
|  |  | *Ttk* | Up | *Gm45645* | Down |
|  |  | *Ttpa* | Up | *Mipepos* | Down |
|  |  | *Ttpal* | Up | *Gm6999* | Down |
|  |  | *Tuba1a* | Up | *BC037032* | Down |
|  |  | *Tuba1b* | Up | *AC133189.1* | Down |
|  |  | *Tuba1c* | Up | *Gm26684* | Down |
|  |  | *Tuba8* | Up | *Gm27924* | Down |
|  |  | *Tubb2a* | Up | *1700001L05Rik* | Down |
|  |  | *Tubb2b* | Up | *Gm15879* | Down |
|  |  | *Tubb3* | Up | *Gm23332* | Down |
|  |  | *Tubb4b* | Up | *Gm15725* | Down |
|  |  | *Tubb5* | Up | *Mir568* | Down |
|  |  | *Tubb6* | Up | *Gm15638* | Down |
|  |  | *Tubg2* | Up | *A930006K02Rik* | Down |
|  |  | *Twist2* | Up | *Gm28052* | Down |
|  |  | *Txndc2* | Up | *Gm20460* | Down |
|  |  | *Txnrd1* | Up | *Nudt12os* | Down |
|  |  | *Tyro3* | Up | *Gm26749* | Down |
|  |  | *Tyrobp* | Up | *Gm17315* | Down |
|  |  | *Ubd* | Up | *Gm29417* | Down |
|  |  | *Ube2c* | Up | *RP24-464B16.1* | Down |
|  |  | *Ube2l6* | Up | *BC037039* | Down |
|  |  | *Ube2t* | Up | *C030005K06Rik* | Down |
|  |  | *Uchl1* | Up | *Mir5107* | Down |
|  |  | *Ucn2* | Up | *F830208F22Rik* | Down |
|  |  | *Ucp2* | Up | *Gm15962* | Down |
|  |  | *Ucp3* | Up | *4931403E22Rik* | Down |
|  |  | *Ugt1a10* | Up | *1700018L02Rik* | Down |
|  |  | *Ugt1a9* | Up | *Gm16726* | Down |
|  |  | *Uhrf1* | Up | *1700034P13Rik* | Down |
|  |  | *Ulbp1* | Up | *Gm38319* | Down |
|  |  | *Unc5a* | Up | *Gm26788* | Down |
|  |  | *Ung* | Up | *Gm20646* | Down |
|  |  | *Uox* | Up | *AC125167.1* | Down |
|  |  | *Upk3bl* | Up | *9130024F11Rik* | Down |
|  |  | *Upp1* | Up | *Apol7d* | Down |
|  |  | *Usp18* | Up | *D230017M19Rik* | Down |
|  |  | *Usp27x* | Up | *Gm19582* | Down |
|  |  | *Usp43* | Up | *Gm19461* | Down |
|  |  | *Usp53* | Up | *Gm15851* | Down |
|  |  | *Vars* | Up | *C730036E19Rik* | Down |
|  |  | *Vash2* | Up | *Gm6652* | Down |
|  |  | *Vasp* | Up | *5330438I03Rik* | Down |
|  |  | *Vat1* | Up | *3110045C21Rik* | Down |
|  |  | *Vcam1* | Up | *Gm7804* | Down |
|  |  | *Vcan* | Up | *4933439K11Rik* | Down |
|  |  | *Vgf* | Up | *Ifi203-ps* | Down |
|  |  | *Vgll3* | Up | *Gm26776* | Down |
|  |  | *Vim* | Up | *Gm13267* | Down |
|  |  | *Vit* | Up | *E030013I19Rik* | Down |
|  |  | *Vmp1* | Up | *Gm37824* | Down |
|  |  | *Vopp1* | Up | *Gm13270* | Down |
|  |  | *Vps36* | Up | *A130006I12Rik* | Down |
|  |  | *Vps37b* | Up | *Gm13441* | Down |
|  |  | *Vsig2* | Up | *Mir5129* | Down |
|  |  | *Vtcn1* | Up | *Gm13502* | Down |
|  |  | *Vwa7* | Up | *Gm26899* | Down |
|  |  | *Wbscr17* | Up | *Gm26697* | Down |
|  |  | *Wbscr28* | Up | *Gm14233* | Down |
|  |  | *Wdhd1* | Up | *E130215H24Rik* | Down |
|  |  | *Wdr62* | Up | *Acss2os* | Down |
|  |  | *Wfdc2* | Up | *Gssos2* | Down |
|  |  | *Wfdc21* | Up | *Gssos1* | Down |
|  |  | *Wfdc3* | Up | *Gm14257* | Down |
|  |  | *Wfikkn1* | Up | *Gm14320* | Down |
|  |  | *Wnt10a* | Up | *Gm6300* | Down |
|  |  | *Wnt2* | Up | *Gm42944* | Down |
|  |  | *Wnt7a* | Up | *Sox2ot* | Down |
|  |  | *Wnt9a* | Up | *Gm15952* | Down |
|  |  | *Xaf1* | Up | *Mccc1os* | Down |
|  |  | *Xdh* | Up | *Gm31266* | Down |
|  |  | *Xirp2* | Up | *Gm26850* | Down |
|  |  | *Xkr5* | Up | *Gm36569* | Down |
|  |  | *Ybx3* | Up | *Gm16000* | Down |
|  |  | *Ydjc* | Up | *Mir7010* | Down |
|  |  | *Ypel2* | Up | *Gm10702* | Down |
|  |  | *Ypel4* | Up | *4930442L01Rik* | Down |
|  |  | *Ywhah* | Up | *Gm42820* | Down |
|  |  | *Yy2* | Up | *Gm43190* | Down |
|  |  | *Zak* | Up | *Gm5547* | Down |
|  |  | *Zbed6* | Up | *Gm43158* | Down |
|  |  | *Zbp1* | Up | *Gm43568* | Down |
|  |  | *Zbtb16* | Up | *Gm42697* | Down |
|  |  | *Zbtb38* | Up | *6330410L21Rik* | Down |
|  |  | *Zbtb42* | Up | *Gm43429* | Down |
|  |  | *Zc3h12a* | Up | *Gm26663* | Down |
|  |  | *Zfand2a* | Up | *Gm12454* | Down |
|  |  | *Zfand4* | Up | *Gm12678* | Down |
|  |  | *Zfp185* | Up | *Gm11266* | Down |
|  |  | *Zfp36* | Up | *Atad3aos* | Down |
|  |  | *Zfp365* | Up | *Gm26840* | Down |
|  |  | *Zfp36l1* | Up | *Gm43031* | Down |
|  |  | *Zfp385a* | Up | *4931431C16Rik* | Down |
|  |  | *Zfp385c* | Up | *Gm7816* | Down |
|  |  | *Zfp458* | Up | *C130083M11Rik* | Down |
|  |  | *Zfp473* | Up | *Gm3716* | Down |
|  |  | *Zfp52* | Up | *Gm43775* | Down |
|  |  | *Zfp57* | Up | *Gm43323* | Down |
|  |  | *Zfp575* | Up | *2310040G07Rik* | Down |
|  |  | *Zfp593* | Up | *Gm42604* | Down |
|  |  | *Zfp599* | Up | *Gm33370* | Down |
|  |  | *Zfp688* | Up | *5430427N15Rik* | Down |
|  |  | *Zfp750* | Up | *Gm42620* | Down |
|  |  | *Zfp850* | Up | *Gm43592* | Down |
|  |  | *Zfp9* | Up | *Gm37415* | Down |
|  |  | *Zfp92* | Up | *A430035B10Rik* | Down |
|  |  | *Zfp948* | Up | *Gm13855* | Down |
|  |  | *Zglp1* | Up | *Gm42962* | Down |
|  |  | *Zgrf1* | Up | *Gm16499* | Down |
|  |  | *Zmat3* | Up | *4930533I22Rik* | Down |
|  |  | *Zswim4* | Up | *2310040G24Rik* | Down |
|  |  | *Zwilch* | Up | *Gm44101* | Down |
|  |  | *Zyg11a* | Up | *Gm10069* | Down |
|  |  | *0610011F06Rik* | Down | *Gm17089* | Down |
|  |  | *1110032F04Rik* | Down | *Gm44238* | Down |
|  |  | *1600014C10Rik* | Down | *Lockd* | Down |
|  |  | *1700037C18Rik* | Down | *Gm44140* | Down |
|  |  | *1700040L02Rik* | Down | *Gm30524* | Down |
|  |  | *1700067K01Rik* | Down | *Gm20400* | Down |
|  |  | *1700112E06Rik* | Down | *Gm7457* | Down |
|  |  | *1810024B03Rik* | Down | *Gm26666* | Down |
|  |  | *1810041L15Rik* | Down | *Gm6654* | Down |
|  |  | *1810043G02Rik* | Down | *Gm6288* | Down |
|  |  | *2210408I21Rik* | Down | *Gm44878* | Down |
|  |  | *2310039H08Rik* | Down | *Gm45138* | Down |
|  |  | *2610528J11Rik* | Down | *Gm44963* | Down |
|  |  | *3010026O09Rik* | Down | *B230322F03Rik* | Down |
|  |  | *3632451O06Rik* | Down | *Gm44832* | Down |
|  |  | *4430402I18Rik* | Down | *2310016G11Rik* | Down |
|  |  | *4833439L19Rik* | Down | *Gm45669* | Down |
|  |  | *4930415F15Rik* | Down | *Gm44536* | Down |
|  |  | *4930452B06Rik* | Down | *Gm44810* | Down |
|  |  | *4930502E18Rik* | Down | *Gm45065* | Down |
|  |  | *4930505A04Rik* | Down | *Gm21057* | Down |
|  |  | *4930524N10Rik* | Down | *Far1os* | Down |
|  |  | *4930579F01Rik* | Down | *RP23-306P12.3* | Down |
|  |  | *4931406C07Rik* | Down | *Gm44866* | Down |
|  |  | *4933403O08Rik* | Down | *Gm15503* | Down |
|  |  | *5330417C22Rik* | Down | *Gm15348* | Down |
|  |  | *5830411N06Rik* | Down | *Gm15350* | Down |
|  |  | *5830473C10Rik* | Down | *Gm15347* | Down |
|  |  | *6430550D23Rik* | Down | *2810030D12Rik* | Down |
|  |  | *A1cf* | Down | *9530085L11Rik* | Down |
|  |  | *A4gnt* | Down | *2500002B13Rik* | Down |
|  |  | *A530099J19Rik* | Down | *Gm9725* | Down |
|  |  | *AF529169* | Down | *Gm16096* | Down |
|  |  | *AI464131* | Down | *RP23-444K20.5* | Down |
|  |  | *AI481877* | Down | *Gm9869* | Down |
|  |  | *Aacs* | Down | *Gm15511* | Down |
|  |  | *Aadac* | Down | *A330041J22Rik* | Down |
|  |  | *Aadat* | Down | *BC043934* | Down |
|  |  | *Aamdc* | Down | *Gm16010* | Down |
|  |  | *Aass* | Down | *Gm37563* | Down |
|  |  | *Abat* | Down | *Gm38314* | Down |
|  |  | *Abca13* | Down | *D030055H07Rik* | Down |
|  |  | *Abca3* | Down | *Gm34106* | Down |
|  |  | *Abca4* | Down | *Gm10030* | Down |
|  |  | *Abcb6* | Down | *Gm14584* | Down |
|  |  | *Abcb8* | Down | *Gm14764* | Down |
|  |  | *Abcc6* | Down |  |  |
|  |  | *Abcc9* | Down |  |  |
|  |  | *Abcd4* | Down |  |  |
|  |  | *Abhd14a* | Down |  |  |
|  |  | *Abhd14b* | Down |  |  |
|  |  | *Abhd17c* | Down |  |  |
|  |  | *Abhd18* | Down |  |  |
|  |  | *Abhd3* | Down |  |  |
|  |  | *Acaa1a* | Down |  |  |
|  |  | *Acaa1b* | Down |  |  |
|  |  | *Acaa2* | Down |  |  |
|  |  | *Acad10* | Down |  |  |
|  |  | *Acad12* | Down |  |  |
|  |  | *Acad9* | Down |  |  |
|  |  | *Acadm* | Down |  |  |
|  |  | *Acads* | Down |  |  |
|  |  | *Acadsb* | Down |  |  |
|  |  | *Acat1* | Down |  |  |
|  |  | *Acbd4* | Down |  |  |
|  |  | *Ace* | Down |  |  |
|  |  | *Ace2* | Down |  |  |
|  |  | *Acmsd* | Down |  |  |
|  |  | *Aco1* | Down |  |  |
|  |  | *Aco2* | Down |  |  |
|  |  | *Acot11* | Down |  |  |
|  |  | *Acot13* | Down |  |  |
|  |  | *Acot3* | Down |  |  |
|  |  | *Acot4* | Down |  |  |
|  |  | *Acox2* | Down |  |  |
|  |  | *Acox3* | Down |  |  |
|  |  | *Acp5* | Down |  |  |
|  |  | *Acp6* | Down |  |  |
|  |  | *Acsf3* | Down |  |  |
|  |  | *Acsl3* | Down |  |  |
|  |  | *Acsm1* | Down |  |  |
|  |  | *Acsm2* | Down |  |  |
|  |  | *Acsm3* | Down |  |  |
|  |  | *Acsm5* | Down |  |  |
|  |  | *Acss1* | Down |  |  |
|  |  | *Acss2* | Down |  |  |
|  |  | *Acss3* | Down |  |  |
|  |  | *Actl9* | Down |  |  |
|  |  | *Actn2* | Down |  |  |
|  |  | *Actn3* | Down |  |  |
|  |  | *Acy1* | Down |  |  |
|  |  | *Acy3* | Down |  |  |
|  |  | *Acyp2* | Down |  |  |
|  |  | *Adamts16* | Down |  |  |
|  |  | *Adamts17* | Down |  |  |
|  |  | *Adamtsl3* | Down |  |  |
|  |  | *Adap2* | Down |  |  |
|  |  | *Adck3* | Down |  |  |
|  |  | *Adcy10* | Down |  |  |
|  |  | *Add3* | Down |  |  |
|  |  | *Adgb* | Down |  |  |
|  |  | *Adgrg4* | Down |  |  |
|  |  | *Adh1* | Down |  |  |
|  |  | *Adhfe1* | Down |  |  |
|  |  | *Adipoq* | Down |  |  |
|  |  | *Adk* | Down |  |  |
|  |  | *Adm2* | Down |  |  |
|  |  | *Adra1a* | Down |  |  |
|  |  | *Adra2b* | Down |  |  |
|  |  | *Adra2c* | Down |  |  |
|  |  | *Adrb1* | Down |  |  |
|  |  | *Adtrp* | Down |  |  |
|  |  | *Afm* | Down |  |  |
|  |  | *Afmid* | Down |  |  |
|  |  | *Agbl3* | Down |  |  |
|  |  | *Agbl4* | Down |  |  |
|  |  | *Agmat* | Down |  |  |
|  |  | *Agmo* | Down |  |  |
|  |  | *Agpat3* | Down |  |  |
|  |  | *Agps* | Down |  |  |
|  |  | *Agtr1a* | Down |  |  |
|  |  | *Agtr2* | Down |  |  |
|  |  | *Agxt2* | Down |  |  |
|  |  | *Ahcy* | Down |  |  |
|  |  | *Ahcyl2* | Down |  |  |
|  |  | *Ahrr* | Down |  |  |
|  |  | *Aif1* | Down |  |  |
|  |  | *Aifm1* | Down |  |  |
|  |  | *Aifm3* | Down |  |  |
|  |  | *Ak3* | Down |  |  |
|  |  | *Ak4* | Down |  |  |
|  |  | *Ak9* | Down |  |  |
|  |  | *Akap5* | Down |  |  |
|  |  | *Akr1a1* | Down |  |  |
|  |  | *Akr1c14* | Down |  |  |
|  |  | *Akr1c20* | Down |  |  |
|  |  | *Akr1c21* | Down |  |  |
|  |  | *Akr1c6* | Down |  |  |
|  |  | *Akr1d1* | Down |  |  |
|  |  | *Akr1e1* | Down |  |  |
|  |  | *Akr7a5* | Down |  |  |
|  |  | *Alas2* | Down |  |  |
|  |  | *Alb* | Down |  |  |
|  |  | *Aldh16a1* | Down |  |  |
|  |  | *Aldh1a3* | Down |  |  |
|  |  | *Aldh1l1* | Down |  |  |
|  |  | *Aldh3a2* | Down |  |  |
|  |  | *Aldh3b3* | Down |  |  |
|  |  | *Aldh4a1* | Down |  |  |
|  |  | *Aldh5a1* | Down |  |  |
|  |  | *Aldh6a1* | Down |  |  |
|  |  | *Aldh7a1* | Down |  |  |
|  |  | *Aldh8a1* | Down |  |  |
|  |  | *Aldh9a1* | Down |  |  |
|  |  | *Aldob* | Down |  |  |
|  |  | *Alk* | Down |  |  |
|  |  | *Alpl* | Down |  |  |
|  |  | *Als2cr12* | Down |  |  |
|  |  | *Amacr* | Down |  |  |
|  |  | *Amdhd1* | Down |  |  |
|  |  | *Amdhd2* | Down |  |  |
|  |  | *Amn* | Down |  |  |
|  |  | *Amt* | Down |  |  |
|  |  | *Amz1* | Down |  |  |
|  |  | *Angpt1* | Down |  |  |
|  |  | *Angptl1* | Down |  |  |
|  |  | *Angptl6* | Down |  |  |
|  |  | *Ank2* | Down |  |  |
|  |  | *Ankib1* | Down |  |  |
|  |  | *Ankrd13c* | Down |  |  |
|  |  | *Anks4b* | Down |  |  |
|  |  | *Ano2* | Down |  |  |
|  |  | *Ano4* | Down |  |  |
|  |  | *Anpep* | Down |  |  |
|  |  | *Anxa13* | Down |  |  |
|  |  | *Aoah* | Down |  |  |
|  |  | *Apcs* | Down |  |  |
|  |  | *Apeh* | Down |  |  |
|  |  | *Aph1c* | Down |  |  |
|  |  | *Apln* | Down |  |  |
|  |  | *Aplnr* | Down |  |  |
|  |  | *Apmap* | Down |  |  |
|  |  | *Apoa2* | Down |  |  |
|  |  | *Apol10b* | Down |  |  |
|  |  | *Apol11b* | Down |  |  |
|  |  | *Apol7c* | Down |  |  |
|  |  | *Apol7e* | Down |  |  |
|  |  | *Apom* | Down |  |  |
|  |  | *Apoo* | Down |  |  |
|  |  | *Aqp1* | Down |  |  |
|  |  | *Aqp11* | Down |  |  |
|  |  | *Ar* | Down |  |  |
|  |  | *Arhgap18* | Down |  |  |
|  |  | *Arhgap24* | Down |  |  |
|  |  | *Arhgap42* | Down |  |  |
|  |  | *Arhgef39* | Down |  |  |
|  |  | *Arl6ip1* | Down |  |  |
|  |  | *Arpp19* | Down |  |  |
|  |  | *Arsb* | Down |  |  |
|  |  | *Arsg* | Down |  |  |
|  |  | *Arsk* | Down |  |  |
|  |  | *Art2b* | Down |  |  |
|  |  | *As3mt* | Down |  |  |
|  |  | *Asb13* | Down |  |  |
|  |  | *Asb2* | Down |  |  |
|  |  | *Asb9* | Down |  |  |
|  |  | *Ascc1* | Down |  |  |
|  |  | *Asl* | Down |  |  |
|  |  | *Aspa* | Down |  |  |
|  |  | *Aspdh* | Down |  |  |
|  |  | *Aspg* | Down |  |  |
|  |  | *Ass1* | Down |  |  |
|  |  | *Astn1* | Down |  |  |
|  |  | *Atg10* | Down |  |  |
|  |  | *Atoh8* | Down |  |  |
|  |  | *Atp11a* | Down |  |  |
|  |  | *Atp2b2* | Down |  |  |
|  |  | *Atp4a* | Down |  |  |
|  |  | *Atp5a1* | Down |  |  |
|  |  | *Atp5b* | Down |  |  |
|  |  | *Atp5c1* | Down |  |  |
|  |  | *Atp5f1* | Down |  |  |
|  |  | *Atp5g1* | Down |  |  |
|  |  | *Atp5g3* | Down |  |  |
|  |  | *Atp5s* | Down |  |  |
|  |  | *Atp5sl* | Down |  |  |
|  |  | *Atp6v1a* | Down |  |  |
|  |  | *Atp6v1b1* | Down |  |  |
|  |  | *Atp6v1c2* | Down |  |  |
|  |  | *Atraid* | Down |  |  |
|  |  | *Atrnl1* | Down |  |  |
|  |  | *Auh* | Down |  |  |
|  |  | *Azgp1* | Down |  |  |
|  |  | *B3galnt1* | Down |  |  |
|  |  | *B3galt5* | Down |  |  |
|  |  | *B3gat2* | Down |  |  |
|  |  | *B4galnt3* | Down |  |  |
|  |  | *B4galt5* | Down |  |  |
|  |  | *BC026585* | Down |  |  |
|  |  | *BC049352* | Down |  |  |
|  |  | *BC089597* | Down |  |  |
|  |  | *Baat* | Down |  |  |
|  |  | *Bach2* | Down |  |  |
|  |  | *Baiap2l2* | Down |  |  |
|  |  | *Baiap3* | Down |  |  |
|  |  | *Bank1* | Down |  |  |
|  |  | *Bbox1* | Down |  |  |
|  |  | *Bbs2* | Down |  |  |
|  |  | *Bbs9* | Down |  |  |
|  |  | *Bcas3* | Down |  |  |
|  |  | *Bckdha* | Down |  |  |
|  |  | *Bckdk* | Down |  |  |
|  |  | *Bcl11b* | Down |  |  |
|  |  | *Bco2* | Down |  |  |
|  |  | *Bdh1* | Down |  |  |
|  |  | *Bdh2* | Down |  |  |
|  |  | *Bend5* | Down |  |  |
|  |  | *Best3* | Down |  |  |
|  |  | *Bhmt* | Down |  |  |
|  |  | *Bhmt2* | Down |  |  |
|  |  | *Blk* | Down |  |  |
|  |  | *Bmp15* | Down |  |  |
|  |  | *Bmp5* | Down |  |  |
|  |  | *Bmp7* | Down |  |  |
|  |  | *Bnc2* | Down |  |  |
|  |  | *Boll* | Down |  |  |
|  |  | *Bphl* | Down |  |  |
|  |  | *Bpnt1* | Down |  |  |
|  |  | *Bsnd* | Down |  |  |
|  |  | *Btbd11* | Down |  |  |
|  |  | *Btd* | Down |  |  |
|  |  | *Btnl10* | Down |  |  |
|  |  | *Btnl9* | Down |  |  |
|  |  | *Bzrap1* | Down |  |  |
|  |  | *C030006K11Rik* | Down |  |  |
|  |  | *C1qtnf2* | Down |  |  |
|  |  | *C1qtnf3* | Down |  |  |
|  |  | *C1rb* | Down |  |  |
|  |  | *C1rl* | Down |  |  |
|  |  | *C2cd2l* | Down |  |  |
|  |  | *C2cd4d* | Down |  |  |
|  |  | *C330018D20Rik* | Down |  |  |
|  |  | *C330021F23Rik* | Down |  |  |
|  |  | *C8a* | Down |  |  |
|  |  | *C8g* | Down |  |  |
|  |  | *Cables1* | Down |  |  |
|  |  | *Cabp2* | Down |  |  |
|  |  | *Cacna1e* | Down |  |  |
|  |  | *Cacna2d2* | Down |  |  |
|  |  | *Cacnb4* | Down |  |  |
|  |  | *Cacng4* | Down |  |  |
|  |  | *Cacng8* | Down |  |  |
|  |  | *Cadm2* | Down |  |  |
|  |  | *Calb1* | Down |  |  |
|  |  | *Calml4* | Down |  |  |
|  |  | *Calr3* | Down |  |  |
|  |  | *Camk1g* | Down |  |  |
|  |  | *Capn6* | Down |  |  |
|  |  | *Car12* | Down |  |  |
|  |  | *Car14* | Down |  |  |
|  |  | *Car3* | Down |  |  |
|  |  | *Car4* | Down |  |  |
|  |  | *Car5a* | Down |  |  |
|  |  | *Car7* | Down |  |  |
|  |  | *Car9* | Down |  |  |
|  |  | *Card11* | Down |  |  |
|  |  | *Caskin1* | Down |  |  |
|  |  | *Casr* | Down |  |  |
|  |  | *Cat* | Down |  |  |
|  |  | *Cbr4* | Down |  |  |
|  |  | *Cbs* | Down |  |  |
|  |  | *Ccdc107* | Down |  |  |
|  |  | *Ccdc150* | Down |  |  |
|  |  | *Ccdc151* | Down |  |  |
|  |  | *Ccdc162* | Down |  |  |
|  |  | *Ccdc170* | Down |  |  |
|  |  | *Ccdc171* | Down |  |  |
|  |  | *Ccdc180* | Down |  |  |
|  |  | *Ccdc191* | Down |  |  |
|  |  | *Ccdc39* | Down |  |  |
|  |  | *Ccdc6* | Down |  |  |
|  |  | *Ccdc62* | Down |  |  |
|  |  | *Ccdc67* | Down |  |  |
|  |  | *Ccdc85c* | Down |  |  |
|  |  | *Ccdc91* | Down |  |  |
|  |  | *Cck* | Down |  |  |
|  |  | *Cckar* | Down |  |  |
|  |  | *Ccl19* | Down |  |  |
|  |  | *Ccl4* | Down |  |  |
|  |  | *Ccl8* | Down |  |  |
|  |  | *Ccpg1* | Down |  |  |
|  |  | *Ccpg1os* | Down |  |  |
|  |  | *Ccs* | Down |  |  |
|  |  | *Ccser1* | Down |  |  |
|  |  | *Cct6b* | Down |  |  |
|  |  | *Cd160* | Down |  |  |
|  |  | *Cd19* | Down |  |  |
|  |  | *Cd2* | Down |  |  |
|  |  | *Cd209a* | Down |  |  |
|  |  | *Cd22* | Down |  |  |
|  |  | *Cd226* | Down |  |  |
|  |  | *Cd27* | Down |  |  |
|  |  | *Cd3d* | Down |  |  |
|  |  | *Cd3g* | Down |  |  |
|  |  | *Cd7* | Down |  |  |
|  |  | *Cd74* | Down |  |  |
|  |  | *Cd83* | Down |  |  |
|  |  | *Cd8a* | Down |  |  |
|  |  | *Cd8b1* | Down |  |  |
|  |  | *Cdh13* | Down |  |  |
|  |  | *Cdh4* | Down |  |  |
|  |  | *Cdhr5* | Down |  |  |
|  |  | *Cdkl1* | Down |  |  |
|  |  | *Cdkl4* | Down |  |  |
|  |  | *Cela1* | Down |  |  |
|  |  | *Celsr2* | Down |  |  |
|  |  | *Ces1d* | Down |  |  |
|  |  | *Ces1e* | Down |  |  |
|  |  | *Ces1f* | Down |  |  |
|  |  | *Ces1g* | Down |  |  |
|  |  | *Ces2c* | Down |  |  |
|  |  | *Ces2h* | Down |  |  |
|  |  | *Ces3a* | Down |  |  |
|  |  | *Cetn4* | Down |  |  |
|  |  | *Cfap126* | Down |  |  |
|  |  | *Cfap54* | Down |  |  |
|  |  | *Cfap61* | Down |  |  |
|  |  | *Cfap70* | Down |  |  |
|  |  | *Cfap77* | Down |  |  |
|  |  | *Cfd* | Down |  |  |
|  |  | *Cgref1* | Down |  |  |
|  |  | *Chac2* | Down |  |  |
|  |  | *Chadl* | Down |  |  |
|  |  | *Chchd10* | Down |  |  |
|  |  | *Chchd6* | Down |  |  |
|  |  | *Chd3* | Down |  |  |
|  |  | *Chd5* | Down |  |  |
|  |  | *Chdh* | Down |  |  |
|  |  | *Chp2* | Down |  |  |
|  |  | *Chpt1* | Down |  |  |
|  |  | *Chrna2* | Down |  |  |
|  |  | *Chrna4* | Down |  |  |
|  |  | *Chrna7* | Down |  |  |
|  |  | *Chst3* | Down |  |  |
|  |  | *Chst7* | Down |  |  |
|  |  | *Ciart* | Down |  |  |
|  |  | *Cidea* | Down |  |  |
|  |  | *Cideb* | Down |  |  |
|  |  | *Ciita* | Down |  |  |
|  |  | *Cisd1* | Down |  |  |
|  |  | *Cit* | Down |  |  |
|  |  | *Ckb* | Down |  |  |
|  |  | *Clcn3* | Down |  |  |
|  |  | *Clcnkb* | Down |  |  |
|  |  | *Cldn10* | Down |  |  |
|  |  | *Cldn18* | Down |  |  |
|  |  | *Cldn2* | Down |  |  |
|  |  | *Cldnd2* | Down |  |  |
|  |  | *Clec2h* | Down |  |  |
|  |  | *Clec3b* | Down |  |  |
|  |  | *Clec4b1* | Down |  |  |
|  |  | *Clec9a* | Down |  |  |
|  |  | *Clic3* | Down |  |  |
|  |  | *Clic6* | Down |  |  |
|  |  | *Clip4* | Down |  |  |
|  |  | *Clnk* | Down |  |  |
|  |  | *Clstn2* | Down |  |  |
|  |  | *Clstn3* | Down |  |  |
|  |  | *Clybl* | Down |  |  |
|  |  | *Cmah* | Down |  |  |
|  |  | *Cmbl* | Down |  |  |
|  |  | *Cmss1* | Down |  |  |
|  |  | *Cmya5* | Down |  |  |
|  |  | *Cndp1* | Down |  |  |
|  |  | *Cndp2* | Down |  |  |
|  |  | *Cnnm1* | Down |  |  |
|  |  | *Cnnm2* | Down |  |  |
|  |  | *Cntd1* | Down |  |  |
|  |  | *Cntn3* | Down |  |  |
|  |  | *Cntn5* | Down |  |  |
|  |  | *Cntnap5a* | Down |  |  |
|  |  | *Coa6* | Down |  |  |
|  |  | *Coasy* | Down |  |  |
|  |  | *Col19a1* | Down |  |  |
|  |  | *Col23a1* | Down |  |  |
|  |  | *Col25a1* | Down |  |  |
|  |  | *Col26a1* | Down |  |  |
|  |  | *Col27a1* | Down |  |  |
|  |  | *Col4a4* | Down |  |  |
|  |  | *Col6a5* | Down |  |  |
|  |  | *Col6a6* | Down |  |  |
|  |  | *Colec11* | Down |  |  |
|  |  | *Comp* | Down |  |  |
|  |  | *Coq9* | Down |  |  |
|  |  | *Corin* | Down |  |  |
|  |  | *Cox6a2* | Down |  |  |
|  |  | *Cox7a1* | Down |  |  |
|  |  | *Cox8b* | Down |  |  |
|  |  | *Cpb2* | Down |  |  |
|  |  | *Cpeb3* | Down |  |  |
|  |  | *Cplx2* | Down |  |  |
|  |  | *Cplx3* | Down |  |  |
|  |  | *Cpn1* | Down |  |  |
|  |  | *Cpne4* | Down |  |  |
|  |  | *Cpox* | Down |  |  |
|  |  | *Cpq* | Down |  |  |
|  |  | *Cpt2* | Down |  |  |
|  |  | *Cr2* | Down |  |  |
|  |  | *Crat* | Down |  |  |
|  |  | *Crb1* | Down |  |  |
|  |  | *Crocc* | Down |  |  |
|  |  | *Crot* | Down |  |  |
|  |  | *Cryaa* | Down |  |  |
|  |  | *Cryga* | Down |  |  |
|  |  | *Cryl1* | Down |  |  |
|  |  | *Cryz* | Down |  |  |
|  |  | *Csad* | Down |  |  |
|  |  | *Csgalnact1* | Down |  |  |
|  |  | *Csrnp3* | Down |  |  |
|  |  | *Csrp2* | Down |  |  |
|  |  | *Cst7* | Down |  |  |
|  |  | *Ctbs* | Down |  |  |
|  |  | *Ctcfl* | Down |  |  |
|  |  | *Cth* | Down |  |  |
|  |  | *Ctnna2* | Down |  |  |
|  |  | *Ctnna3* | Down |  |  |
|  |  | *Ctnnbip1* | Down |  |  |
|  |  | *Ctsf* | Down |  |  |
|  |  | *Ctsw* | Down |  |  |
|  |  | *Cttnbp2* | Down |  |  |
|  |  | *Cubn* | Down |  |  |
|  |  | *Cux2* | Down |  |  |
|  |  | *Cwh43* | Down |  |  |
|  |  | *Cxcl12* | Down |  |  |
|  |  | *Cxcr3* | Down |  |  |
|  |  | *Cxcr6* | Down |  |  |
|  |  | *Cxx1c* | Down |  |  |
|  |  | *Cyb561d2* | Down |  |  |
|  |  | *Cyb5a* | Down |  |  |
|  |  | *Cyb5rl* | Down |  |  |
|  |  | *Cyba* | Down |  |  |
|  |  | *Cym* | Down |  |  |
|  |  | *Cyp2a4* | Down |  |  |
|  |  | *Cyp2a5* | Down |  |  |
|  |  | *Cyp2c38* | Down |  |  |
|  |  | *Cyp2c44* | Down |  |  |
|  |  | *Cyp2c68* | Down |  |  |
|  |  | *Cyp2c69* | Down |  |  |
|  |  | *Cyp2d12* | Down |  |  |
|  |  | *Cyp2d26* | Down |  |  |
|  |  | *Cyp2e1* | Down |  |  |
|  |  | *Cyp2f2* | Down |  |  |
|  |  | *Cyp2j11* | Down |  |  |
|  |  | *Cyp2j13* | Down |  |  |
|  |  | *Cyp2j5* | Down |  |  |
|  |  | *Cyp2j8* | Down |  |  |
|  |  | *Cyp4a12a* | Down |  |  |
|  |  | *Cyp4b1* | Down |  |  |
|  |  | *Cyp4f14* | Down |  |  |
|  |  | *Cyp4f15* | Down |  |  |
|  |  | *Cyp51* | Down |  |  |
|  |  | *Cyp7b1* | Down |  |  |
|  |  | *Cys1* | Down |  |  |
|  |  | *D10Jhu81e* | Down |  |  |
|  |  | *D130043K22Rik* | Down |  |  |
|  |  | *D2hgdh* | Down |  |  |
|  |  | *D3Ertd751e* | Down |  |  |
|  |  | *D430019H16Rik* | Down |  |  |
|  |  | *D630003M21Rik* | Down |  |  |
|  |  | *D630023F18Rik* | Down |  |  |
|  |  | *D7Ertd443e* | Down |  |  |
|  |  | *Dab2* | Down |  |  |
|  |  | *Dao* | Down |  |  |
|  |  | *Dars2* | Down |  |  |
|  |  | *Dbp* | Down |  |  |
|  |  | *Dbt* | Down |  |  |
|  |  | *Dcaf8* | Down |  |  |
|  |  | *Dcst1* | Down |  |  |
|  |  | *Ddo* | Down |  |  |
|  |  | *Ddt* | Down |  |  |
|  |  | *Deb1* | Down |  |  |
|  |  | *Def8* | Down |  |  |
|  |  | *Defb19* | Down |  |  |
|  |  | *Defb2* | Down |  |  |
|  |  | *Defb29* | Down |  |  |
|  |  | *Degs2* | Down |  |  |
|  |  | *Dennd1a* | Down |  |  |
|  |  | *Depdc1b* | Down |  |  |
|  |  | *Derl3* | Down |  |  |
|  |  | *Dfna5* | Down |  |  |
|  |  | *Dfnb59* | Down |  |  |
|  |  | *Dgat2* | Down |  |  |
|  |  | *Dgkb* | Down |  |  |
|  |  | *Dgkg* | Down |  |  |
|  |  | *Dgki* | Down |  |  |
|  |  | *Dhcr7* | Down |  |  |
|  |  | *Dhdh* | Down |  |  |
|  |  | *Dhfr* | Down |  |  |
|  |  | *Dhrs3* | Down |  |  |
|  |  | *Dhrs4* | Down |  |  |
|  |  | *Dhtkd1* | Down |  |  |
|  |  | *Dio1* | Down |  |  |
|  |  | *Dis3l2* | Down |  |  |
|  |  | *Dkk2* | Down |  |  |
|  |  | *Dld* | Down |  |  |
|  |  | *Dlec1* | Down |  |  |
|  |  | *Dleu7* | Down |  |  |
|  |  | *Dlg2* | Down |  |  |
|  |  | *Dlgap1* | Down |  |  |
|  |  | *Dlgap3* | Down |  |  |
|  |  | *Dll4* | Down |  |  |
|  |  | *Dmgdh* | Down |  |  |
|  |  | *Dmtn* | Down |  |  |
|  |  | *Dnah11* | Down |  |  |
|  |  | *Dnah17* | Down |  |  |
|  |  | *Dnah2* | Down |  |  |
|  |  | *Dnah3* | Down |  |  |
|  |  | *Dnah5* | Down |  |  |
|  |  | *Dnah7b* | Down |  |  |
|  |  | *Dnah7c* | Down |  |  |
|  |  | *Dnah9* | Down |  |  |
|  |  | *Dnaja1* | Down |  |  |
|  |  | *Dnajc12* | Down |  |  |
|  |  | *Dnajc13* | Down |  |  |
|  |  | *Dnajc19* | Down |  |  |
|  |  | *Dnajc22* | Down |  |  |
|  |  | *Dnajc28* | Down |  |  |
|  |  | *Dnajc30* | Down |  |  |
|  |  | *Dnajc6* | Down |  |  |
|  |  | *Dnase1* | Down |  |  |
|  |  | *Dnase1l3* | Down |  |  |
|  |  | *Dpep1* | Down |  |  |
|  |  | *Dpf3* | Down |  |  |
|  |  | *Dpp10* | Down |  |  |
|  |  | *Dpp6* | Down |  |  |
|  |  | *Dpp7* | Down |  |  |
|  |  | *Dpyd* | Down |  |  |
|  |  | *Dpys* | Down |  |  |
|  |  | *Dqx1* | Down |  |  |
|  |  | *Dtd1* | Down |  |  |
|  |  | *Dtx1* | Down |  |  |
|  |  | *Dusp15* | Down |  |  |
|  |  | *Dusp9* | Down |  |  |
|  |  | *Dync2h1* | Down |  |  |
|  |  | *Dynll2* | Down |  |  |
|  |  | *Dyx1c1* | Down |  |  |
|  |  | *Dzank1* | Down |  |  |
|  |  | *Eaf2* | Down |  |  |
|  |  | *Ebp* | Down |  |  |
|  |  | *Ebpl* | Down |  |  |
|  |  | *Ech1* | Down |  |  |
|  |  | *Echdc1* | Down |  |  |
|  |  | *Echdc2* | Down |  |  |
|  |  | *Echdc3* | Down |  |  |
|  |  | *Echs1* | Down |  |  |
|  |  | *Eci3* | Down |  |  |
|  |  | *Ecsit* | Down |  |  |
|  |  | *Edar* | Down |  |  |
|  |  | *Eddm3b* | Down |  |  |
|  |  | *Edil3* | Down |  |  |
|  |  | *Eefsec* | Down |  |  |
|  |  | *Efcab2* | Down |  |  |
|  |  | *Efcc1* | Down |  |  |
|  |  | *Efhc2* | Down |  |  |
|  |  | *Egf* | Down |  |  |
|  |  | *Egfl6* | Down |  |  |
|  |  | *Ehhadh* | Down |  |  |
|  |  | *Elavl3* | Down |  |  |
|  |  | *Ell3* | Down |  |  |
|  |  | *Elovl2* | Down |  |  |
|  |  | *Eml6* | Down |  |  |
|  |  | *Emx1* | Down |  |  |
|  |  | *Enox1* | Down |  |  |
|  |  | *Enpep* | Down |  |  |
|  |  | *Enpp2* | Down |  |  |
|  |  | *Enpp3* | Down |  |  |
|  |  | *Enpp6* | Down |  |  |
|  |  | *Entpd8* | Down |  |  |
|  |  | *Eny2* | Down |  |  |
|  |  | *Epb41l3* | Down |  |  |
|  |  | *Epha6* | Down |  |  |
|  |  | *Ephb1* | Down |  |  |
|  |  | *Ephx2* | Down |  |  |
|  |  | *Epm2a* | Down |  |  |
|  |  | *Erc2* | Down |  |  |
|  |  | *Erich3* | Down |  |  |
|  |  | *Erich4* | Down |  |  |
|  |  | *Erlin1* | Down |  |  |
|  |  | *Esm1* | Down |  |  |
|  |  | *Esr1* | Down |  |  |
|  |  | *Esr2* | Down |  |  |
|  |  | *Esrra* | Down |  |  |
|  |  | *Esrrg* | Down |  |  |
|  |  | *Etfa* | Down |  |  |
|  |  | *Etfdh* | Down |  |  |
|  |  | *Etnppl* | Down |  |  |
|  |  | *Etv1* | Down |  |  |
|  |  | *Evc* | Down |  |  |
|  |  | *F13b* | Down |  |  |
|  |  | *F5* | Down |  |  |
|  |  | *F8* | Down |  |  |
|  |  | *Faah* | Down |  |  |
|  |  | *Fabp3* | Down |  |  |
|  |  | *Fabp7* | Down |  |  |
|  |  | *Fads2* | Down |  |  |
|  |  | *Fads3* | Down |  |  |
|  |  | *Fads6* | Down |  |  |
|  |  | *Fah* | Down |  |  |
|  |  | *Fahd1* | Down |  |  |
|  |  | *Fahd2a* | Down |  |  |
|  |  | *Fam101a* | Down |  |  |
|  |  | *Fam107a* | Down |  |  |
|  |  | *Fam117a* | Down |  |  |
|  |  | *Fam120c* | Down |  |  |
|  |  | *Fam132a* | Down |  |  |
|  |  | *Fam13a* | Down |  |  |
|  |  | *Fam151a* | Down |  |  |
|  |  | *Fam166a* | Down |  |  |
|  |  | *Fam166b* | Down |  |  |
|  |  | *Fam169b* | Down |  |  |
|  |  | *Fam184a* | Down |  |  |
|  |  | *Fam188b* | Down |  |  |
|  |  | *Fam195a* | Down |  |  |
|  |  | *Fam198a* | Down |  |  |
|  |  | *Fam205a1* | Down |  |  |
|  |  | *Fam210a* | Down |  |  |
|  |  | *Fam213b* | Down |  |  |
|  |  | *Fam214a* | Down |  |  |
|  |  | *Fam217a* | Down |  |  |
|  |  | *Fam228a* | Down |  |  |
|  |  | *Fam228b* | Down |  |  |
|  |  | *Fam229b* | Down |  |  |
|  |  | *Fam26f* | Down |  |  |
|  |  | *Fam46c* | Down |  |  |
|  |  | *Fam69b* | Down |  |  |
|  |  | *Fam71d* | Down |  |  |
|  |  | *Fam78a* | Down |  |  |
|  |  | *Fam81a* | Down |  |  |
|  |  | *Fancd2os* | Down |  |  |
|  |  | *Far1* | Down |  |  |
|  |  | *Fars2* | Down |  |  |
|  |  | *Fastkd1* | Down |  |  |
|  |  | *Fat3* | Down |  |  |
|  |  | *Fbln7* | Down |  |  |
|  |  | *Fbp1* | Down |  |  |
|  |  | *Fbxl2* | Down |  |  |
|  |  | *Fbxl21* | Down |  |  |
|  |  | *Fbxo36* | Down |  |  |
|  |  | *Fbxo40* | Down |  |  |
|  |  | *Fbxo47* | Down |  |  |
|  |  | *Fbxo7* | Down |  |  |
|  |  | *Fbxw15* | Down |  |  |
|  |  | *Fcamr* | Down |  |  |
|  |  | *Fcgrt* | Down |  |  |
|  |  | *Fdxr* | Down |  |  |
|  |  | *Fer1l6* | Down |  |  |
|  |  | *Fgd2* | Down |  |  |
|  |  | *Fgf1* | Down |  |  |
|  |  | *Fgf10* | Down |  |  |
|  |  | *Fgf13* | Down |  |  |
|  |  | *Fgfr4* | Down |  |  |
|  |  | *Fggy* | Down |  |  |
|  |  | *Fhit* | Down |  |  |
|  |  | *Fhod3* | Down |  |  |
|  |  | *Fitm1* | Down |  |  |
|  |  | *Fkbp4* | Down |  |  |
|  |  | *Flrt1* | Down |  |  |
|  |  | *Fmnl1* | Down |  |  |
|  |  | *Fmo1* | Down |  |  |
|  |  | *Fmo2* | Down |  |  |
|  |  | *Fmo4* | Down |  |  |
|  |  | *Fmo5* | Down |  |  |
|  |  | *Fmod* | Down |  |  |
|  |  | *Fn3k* | Down |  |  |
|  |  | *Fndc7* | Down |  |  |
|  |  | *Fnip2* | Down |  |  |
|  |  | *Focad* | Down |  |  |
|  |  | *Folr1* | Down |  |  |
|  |  | *Foxred1* | Down |  |  |
|  |  | *Fras1* | Down |  |  |
|  |  | *Frmd7* | Down |  |  |
|  |  | *Frmpd1* | Down |  |  |
|  |  | *Frmpd3* | Down |  |  |
|  |  | *Frs3* | Down |  |  |
|  |  | *Fsip1* | Down |  |  |
|  |  | *Fuca2* | Down |  |  |
|  |  | *Fut8* | Down |  |  |
|  |  | *Fut9* | Down |  |  |
|  |  | *Fuz* | Down |  |  |
|  |  | *Fxyd2* | Down |  |  |
|  |  | *G0s2* | Down |  |  |
|  |  | *G6b* | Down |  |  |
|  |  | *G6pc* | Down |  |  |
|  |  | *G6pc2* | Down |  |  |
|  |  | *Gaa* | Down |  |  |
|  |  | *Gabarapl1* | Down |  |  |
|  |  | *Gabbr2* | Down |  |  |
|  |  | *Gabrb3* | Down |  |  |
|  |  | *Gabrd* | Down |  |  |
|  |  | *Gabrr2* | Down |  |  |
|  |  | *Galc* | Down |  |  |
|  |  | *Galm* | Down |  |  |
|  |  | *Galns* | Down |  |  |
|  |  | *Galnt11* | Down |  |  |
|  |  | *Galnt14* | Down |  |  |
|  |  | *Gamt* | Down |  |  |
|  |  | *Ganc* | Down |  |  |
|  |  | *Gas1* | Down |  |  |
|  |  | *Gas2* | Down |  |  |
|  |  | *Gata5* | Down |  |  |
|  |  | *Gatb* | Down |  |  |
|  |  | *Gatm* | Down |  |  |
|  |  | *Gatsl2* | Down |  |  |
|  |  | *Gbp10* | Down |  |  |
|  |  | *Gbp2b* | Down |  |  |
|  |  | *Gbp6* | Down |  |  |
|  |  | *Gcat* | Down |  |  |
|  |  | *Gcdh* | Down |  |  |
|  |  | *Gcgr* | Down |  |  |
|  |  | *Gcnt1* | Down |  |  |
|  |  | *Gcsam* | Down |  |  |
|  |  | *Gdf10* | Down |  |  |
|  |  | *Gdf7* | Down |  |  |
|  |  | *Gfm1* | Down |  |  |
|  |  | *Gfm2* | Down |  |  |
|  |  | *Ggact* | Down |  |  |
|  |  | *Ggct* | Down |  |  |
|  |  | *Ggnbp1* | Down |  |  |
|  |  | *Ggt1* | Down |  |  |
|  |  | *Ggt6* | Down |  |  |
|  |  | *Ghr* | Down |  |  |
|  |  | *Gimap7* | Down |  |  |
|  |  | *Gja3* | Down |  |  |
|  |  | *Gjb1* | Down |  |  |
|  |  | *Gjb2* | Down |  |  |
|  |  | *Gk* | Down |  |  |
|  |  | *Glb1* | Down |  |  |
|  |  | *Glb1l* | Down |  |  |
|  |  | *Glis1* | Down |  |  |
|  |  | *Glis2* | Down |  |  |
|  |  | *Glod5* | Down |  |  |
|  |  | *Glt8d2* | Down |  |  |
|  |  | *Gltpd2* | Down |  |  |
|  |  | *Glyat* | Down |  |  |
|  |  | *Glyctk* | Down |  |  |
|  |  | *Gm10639* | Down |  |  |
|  |  | *Gm10681* | Down |  |  |
|  |  | *Gm11127* | Down |  |  |
|  |  | *Gm1123* | Down |  |  |
|  |  | *Gm11837* | Down |  |  |
|  |  | *Gm11992* | Down |  |  |
|  |  | *Gm128* | Down |  |  |
|  |  | *Gm14295* | Down |  |  |
|  |  | *Gm14326* | Down |  |  |
|  |  | *Gm14680* | Down |  |  |
|  |  | *Gm15411* | Down |  |  |
|  |  | *Gm16432* | Down |  |  |
|  |  | *Gm16505* | Down |  |  |
|  |  | *Gm21685* | Down |  |  |
|  |  | *Gm27239* | Down |  |  |
|  |  | *Gm2a* | Down |  |  |
|  |  | *Gm3244* | Down |  |  |
|  |  | *Gm33049* | Down |  |  |
|  |  | *Gm33153* | Down |  |  |
|  |  | *Gm33691* | Down |  |  |
|  |  | *Gm35938* | Down |  |  |
|  |  | *Gm36028* | Down |  |  |
|  |  | *Gm38481* | Down |  |  |
|  |  | *Gm38717* | Down |  |  |
|  |  | *Gm39743* | Down |  |  |
|  |  | *Gm40055* | Down |  |  |
|  |  | *Gm40600* | Down |  |  |
|  |  | *Gm4131* | Down |  |  |
|  |  | *Gm42102* | Down |  |  |
|  |  | *Gm4450* | Down |  |  |
|  |  | *Gm4737* | Down |  |  |
|  |  | *Gm4841* | Down |  |  |
|  |  | *Gm4925* | Down |  |  |
|  |  | *Gm4952* | Down |  |  |
|  |  | *Gm5113* | Down |  |  |
|  |  | *Gm5174* | Down |  |  |
|  |  | *Gm5617* | Down |  |  |
|  |  | *Gm567* | Down |  |  |
|  |  | *Gm5820* | Down |  |  |
|  |  | *Gm6551* | Down |  |  |
|  |  | *Gm6637* | Down |  |  |
|  |  | *Gm6878* | Down |  |  |
|  |  | *Gm7889* | Down |  |  |
|  |  | *Gm853* | Down |  |  |
|  |  | *Gm867* | Down |  |  |
|  |  | *Gm8700* | Down |  |  |
|  |  | *Gm8765* | Down |  |  |
|  |  | *Gm906* | Down |  |  |
|  |  | *Gm973* | Down |  |  |
|  |  | *Gna12* | Down |  |  |
|  |  | *Gna14* | Down |  |  |
|  |  | *Gng4* | Down |  |  |
|  |  | *Gnmt* | Down |  |  |
|  |  | *Gnpda1* | Down |  |  |
|  |  | *Gp2* | Down |  |  |
|  |  | *Gpc4* | Down |  |  |
|  |  | *Gpc5* | Down |  |  |
|  |  | *Gpd1* | Down |  |  |
|  |  | *Gphn* | Down |  |  |
|  |  | *Gpm6a* | Down |  |  |
|  |  | *Gpr12* | Down |  |  |
|  |  | *Gpr135* | Down |  |  |
|  |  | *Gpr137b* | Down |  |  |
|  |  | *Gpr155* | Down |  |  |
|  |  | *Gpr174* | Down |  |  |
|  |  | *Gpr179* | Down |  |  |
|  |  | *Gpr34* | Down |  |  |
|  |  | *Gpr37* | Down |  |  |
|  |  | *Gpr88* | Down |  |  |
|  |  | *Gprasp2* | Down |  |  |
|  |  | *Gpx1* | Down |  |  |
|  |  | *Gpx3* | Down |  |  |
|  |  | *Gpx6* | Down |  |  |
|  |  | *Grb7* | Down |  |  |
|  |  | *Grhpr* | Down |  |  |
|  |  | *Grid1* | Down |  |  |
|  |  | *Grin2c* | Down |  |  |
|  |  | *Grin3a* | Down |  |  |
|  |  | *Grin3b* | Down |  |  |
|  |  | *Grip2* | Down |  |  |
|  |  | *Grpr* | Down |  |  |
|  |  | *Grsf1* | Down |  |  |
|  |  | *Grtp1* | Down |  |  |
|  |  | *Gss* | Down |  |  |
|  |  | *Gstk1* | Down |  |  |
|  |  | *Gsto2* | Down |  |  |
|  |  | *Gstt2* | Down |  |  |
|  |  | *Gstz1* | Down |  |  |
|  |  | *Gtdc1* | Down |  |  |
|  |  | *Gtf2h2* | Down |  |  |
|  |  | *Gtf2i* | Down |  |  |
|  |  | *Gucy1b2* | Down |  |  |
|  |  | *Gucy2f* | Down |  |  |
|  |  | *Gucy2g* | Down |  |  |
|  |  | *Gys2* | Down |  |  |
|  |  | *Gzmb* | Down |  |  |
|  |  | *H2-Aa* | Down |  |  |
|  |  | *H2-Ab1* | Down |  |  |
|  |  | *H2-DMb1* | Down |  |  |
|  |  | *H2-Eb1* | Down |  |  |
|  |  | *H2-Ke6* | Down |  |  |
|  |  | *H2-M2* | Down |  |  |
|  |  | *H2-Oa* | Down |  |  |
|  |  | *H2-Ob* | Down |  |  |
|  |  | *Haao* | Down |  |  |
|  |  | *Habp2* | Down |  |  |
|  |  | *Hacl1* | Down |  |  |
|  |  | *Hadh* | Down |  |  |
|  |  | *Hao2* | Down |  |  |
|  |  | *Hapln1* | Down |  |  |
|  |  | *Hba-a1* | Down |  |  |
|  |  | *Hba-a2* | Down |  |  |
|  |  | *Hbb-bs* | Down |  |  |
|  |  | *Hbb-bt* | Down |  |  |
|  |  | *Hcar1* | Down |  |  |
|  |  | *Hdhd3* | Down |  |  |
|  |  | *Hebp1* | Down |  |  |
|  |  | *Hemk1* | Down |  |  |
|  |  | *Henmt1* | Down |  |  |
|  |  | *Herc4* | Down |  |  |
|  |  | *Herpud1* | Down |  |  |
|  |  | *Hes6* | Down |  |  |
|  |  | *Hexb* | Down |  |  |
|  |  | *Hexdc* | Down |  |  |
|  |  | *Hgd* | Down |  |  |
|  |  | *Hhatl* | Down |  |  |
|  |  | *Hhip* | Down |  |  |
|  |  | *Hibadh* | Down |  |  |
|  |  | *Hibch* | Down |  |  |
|  |  | *Higd1c* | Down |  |  |
|  |  | *Hint2* | Down |  |  |
|  |  | *Hlf* | Down |  |  |
|  |  | *Hmgcs1* | Down |  |  |
|  |  | *Hmx2* | Down |  |  |
|  |  | *Hnf1a* | Down |  |  |
|  |  | *Hnf4a* | Down |  |  |
|  |  | *Hnf4g* | Down |  |  |
|  |  | *Hnmt* | Down |  |  |
|  |  | *Hpd* | Down |  |  |
|  |  | *Hpgd* | Down |  |  |
|  |  | *Hpn* | Down |  |  |
|  |  | *Hps5* | Down |  |  |
|  |  | *Hrasls* | Down |  |  |
|  |  | *Hrg* | Down |  |  |
|  |  | *Hrh3* | Down |  |  |
|  |  | *Hs6st3* | Down |  |  |
|  |  | *Hsd17b11* | Down |  |  |
|  |  | *Hsd3b2* | Down |  |  |
|  |  | *Hsd3b3* | Down |  |  |
|  |  | *Hsd3b4* | Down |  |  |
|  |  | *Hsf2bp* | Down |  |  |
|  |  | *Hsf3* | Down |  |  |
|  |  | *Hspa8* | Down |  |  |
|  |  | *Hspd1* | Down |  |  |
|  |  | *Hspe1* | Down |  |  |
|  |  | *Htr3a* | Down |  |  |
|  |  | *Hunk* | Down |  |  |
|  |  | *Hydin* | Down |  |  |
|  |  | *Hykk* | Down |  |  |
|  |  | *Iah1* | Down |  |  |
|  |  | *Id4* | Down |  |  |
|  |  | *Idh2* | Down |  |  |
|  |  | *Idh3b* | Down |  |  |
|  |  | *Idh3g* | Down |  |  |
|  |  | *Idi1* | Down |  |  |
|  |  | *Ido2* | Down |  |  |
|  |  | *Ifrd2* | Down |  |  |
|  |  | *Ift122* | Down |  |  |
|  |  | *Ift172* | Down |  |  |
|  |  | *Igf1* | Down |  |  |
|  |  | *Igfals* | Down |  |  |
|  |  | *Igfbp1* | Down |  |  |
|  |  | *Igfbp4* | Down |  |  |
|  |  | *Igfbp5* | Down |  |  |
|  |  | *Ikzf3* | Down |  |  |
|  |  | *Il11ra1* | Down |  |  |
|  |  | *Il15* | Down |  |  |
|  |  | *Il17f* | Down |  |  |
|  |  | *Il22ra1* | Down |  |  |
|  |  | *Ildr2* | Down |  |  |
|  |  | *Immp2l* | Down |  |  |
|  |  | *Immt* | Down |  |  |
|  |  | *Inmt* | Down |  |  |
|  |  | *Inpp5j* | Down |  |  |
|  |  | *Insc* | Down |  |  |
|  |  | *Insig1* | Down |  |  |
|  |  | *Insrr* | Down |  |  |
|  |  | *Invs* | Down |  |  |
|  |  | *Ipcef1* | Down |  |  |
|  |  | *Iqcg* | Down |  |  |
|  |  | *Iqsec2* | Down |  |  |
|  |  | *Irs1* | Down |  |  |
|  |  | *Irx1* | Down |  |  |
|  |  | *Isoc2a* | Down |  |  |
|  |  | *Isoc2b* | Down |  |  |
|  |  | *Ispd* | Down |  |  |
|  |  | *Itgad* | Down |  |  |
|  |  | *Itgb8* | Down |  |  |
|  |  | *Itih2* | Down |  |  |
|  |  | *Itih5* | Down |  |  |
|  |  | *Ivd* | Down |  |  |
|  |  | *Iyd* | Down |  |  |
|  |  | *Jmjd8* | Down |  |  |
|  |  | *Kank4* | Down |  |  |
|  |  | *Kansl1l* | Down |  |  |
|  |  | *Kap* | Down |  |  |
|  |  | *Kcne3* | Down |  |  |
|  |  | *Kcng1* | Down |  |  |
|  |  | *Kcnh7* | Down |  |  |
|  |  | *Kcnj1* | Down |  |  |
|  |  | *Kcnj10* | Down |  |  |
|  |  | *Kcnj12* | Down |  |  |
|  |  | *Kcnj13* | Down |  |  |
|  |  | *Kcnj16* | Down |  |  |
|  |  | *Kcnj3* | Down |  |  |
|  |  | *Kcnk2* | Down |  |  |
|  |  | *Kcnk5* | Down |  |  |
|  |  | *Kcnma1* | Down |  |  |
|  |  | *Kcnn1* | Down |  |  |
|  |  | *Kcnn2* | Down |  |  |
|  |  | *Kcnq3* | Down |  |  |
|  |  | *Kcns1* | Down |  |  |
|  |  | *Kcnt1* | Down |  |  |
|  |  | *Kcp* | Down |  |  |
|  |  | *Kdm1b* | Down |  |  |
|  |  | *Keg1* | Down |  |  |
|  |  | *Khk* | Down |  |  |
|  |  | *Kidins220* | Down |  |  |
|  |  | *Kif12* | Down |  |  |
|  |  | *Kif20b* | Down |  |  |
|  |  | *Kif21a* | Down |  |  |
|  |  | *Kif27* | Down |  |  |
|  |  | *Kif6* | Down |  |  |
|  |  | *Kl* | Down |  |  |
|  |  | *Klc4* | Down |  |  |
|  |  | *Klf1* | Down |  |  |
|  |  | *Klf12* | Down |  |  |
|  |  | *Klf15* | Down |  |  |
|  |  | *Klhdc9* | Down |  |  |
|  |  | *Klhl3* | Down |  |  |
|  |  | *Klhl32* | Down |  |  |
|  |  | *Klhl36* | Down |  |  |
|  |  | *Klk1* | Down |  |  |
|  |  | *Klk1b16* | Down |  |  |
|  |  | *Klkb1* | Down |  |  |
|  |  | *Klra8* | Down |  |  |
|  |  | *Klrb1f* | Down |  |  |
|  |  | *Klrg1* | Down |  |  |
|  |  | *Kmo* | Down |  |  |
|  |  | *Krt222* | Down |  |  |
|  |  | *Ksr2* | Down |  |  |
|  |  | *Ky* | Down |  |  |
|  |  | *Kynu* | Down |  |  |
|  |  | *L2hgdh* | Down |  |  |
|  |  | *L3hypdh* | Down |  |  |
|  |  | *L3mbtl4* | Down |  |  |
|  |  | *LOC102633345* | Down |  |  |
|  |  | *LOC102638888* | Down |  |  |
|  |  | *LOC102639505* | Down |  |  |
|  |  | *LOC102639653* | Down |  |  |
|  |  | *LOC105242472* | Down |  |  |
|  |  | *LOC108167886* | Down |  |  |
|  |  | *LOC108168181* | Down |  |  |
|  |  | *LOC108168740* | Down |  |  |
|  |  | *LOC108168923* | Down |  |  |
|  |  | *Lactb* | Down |  |  |
|  |  | *Lactb2* | Down |  |  |
|  |  | *Lama1* | Down |  |  |
|  |  | *Lamc3* | Down |  |  |
|  |  | *Lamtor5* | Down |  |  |
|  |  | *Lap3* | Down |  |  |
|  |  | *Lars2* | Down |  |  |
|  |  | *Lat* | Down |  |  |
|  |  | *Lctl* | Down |  |  |
|  |  | *Ldlrad3* | Down |  |  |
|  |  | *Lekr1* | Down |  |  |
|  |  | *Lgi3* | Down |  |  |
|  |  | *Lgr5* | Down |  |  |
|  |  | *Lgr6* | Down |  |  |
|  |  | *Lhfpl1* | Down |  |  |
|  |  | *Lhx1* | Down |  |  |
|  |  | *Lin7a* | Down |  |  |
|  |  | *Lin7b* | Down |  |  |
|  |  | *Lipa* | Down |  |  |
|  |  | *Lipo2* | Down |  |  |
|  |  | *Lipo3* | Down |  |  |
|  |  | *Lman2l* | Down |  |  |
|  |  | *Lmbrd2* | Down |  |  |
|  |  | *Lmo4* | Down |  |  |
|  |  | *Lmod3* | Down |  |  |
|  |  | *Lonp2* | Down |  |  |
|  |  | *Lpar3* | Down |  |  |
|  |  | *Lpl* | Down |  |  |
|  |  | *Lrat* | Down |  |  |
|  |  | *Lrfn2* | Down |  |  |
|  |  | *Lrp2* | Down |  |  |
|  |  | *Lrp3* | Down |  |  |
|  |  | *Lrpap1* | Down |  |  |
|  |  | *Lrpprc* | Down |  |  |
|  |  | *Lrrc16b* | Down |  |  |
|  |  | *Lrrc19* | Down |  |  |
|  |  | *Lrrc3* | Down |  |  |
|  |  | *Lrrc34* | Down |  |  |
|  |  | *Lrrc3b* | Down |  |  |
|  |  | *Lrrc48* | Down |  |  |
|  |  | *Lrrc52* | Down |  |  |
|  |  | *Lrrc55* | Down |  |  |
|  |  | *Lrrc56* | Down |  |  |
|  |  | *Lrrc61* | Down |  |  |
|  |  | *Lrrc66* | Down |  |  |
|  |  | *Lrriq3* | Down |  |  |
|  |  | *Lrrk2* | Down |  |  |
|  |  | *Lrrn1* | Down |  |  |
|  |  | *Lrtm1* | Down |  |  |
|  |  | *Lsamp* | Down |  |  |
|  |  | *Ltbp4* | Down |  |  |
|  |  | *Lypd6* | Down |  |  |
|  |  | *Lypla1* | Down |  |  |
|  |  | *Lyplal1* | Down |  |  |
|  |  | *Lyrm4* | Down |  |  |
|  |  | *Lztr1* | Down |  |  |
|  |  | *Lzts3* | Down |  |  |
|  |  | *Macrod1* | Down |  |  |
|  |  | *Macrod2* | Down |  |  |
|  |  | *Maf* | Down |  |  |
|  |  | *Magi1* | Down |  |  |
|  |  | *Magix* | Down |  |  |
|  |  | *Malrd1* | Down |  |  |
|  |  | *Man2b1* | Down |  |  |
|  |  | *Mansc4* | Down |  |  |
|  |  | *Map2k6* | Down |  |  |
|  |  | *Map3k15* | Down |  |  |
|  |  | *Map3k7cl* | Down |  |  |
|  |  | *Map7* | Down |  |  |
|  |  | *Mapk10* | Down |  |  |
|  |  | *Mapk15* | Down |  |  |
|  |  | *Mapk8ip1* | Down |  |  |
|  |  | *Mapt* | Down |  |  |
|  |  | *2-Mar* | Down |  |  |
|  |  | *10-Mar* | Down |  |  |
|  |  | *6-Mar* | Down |  |  |
|  |  | *Mat2a* | Down |  |  |
|  |  | *Mbl1* | Down |  |  |
|  |  | *Mbl2* | Down |  |  |
|  |  | *Mblac2* | Down |  |  |
|  |  | *Mccc1* | Down |  |  |
|  |  | *Mccc2* | Down |  |  |
|  |  | *Mcee* | Down |  |  |
|  |  | *Mcf2* | Down |  |  |
|  |  | *Mcur1* | Down |  |  |
|  |  | *Mdh1* | Down |  |  |
|  |  | *Mdh1b* | Down |  |  |
|  |  | *Mdk* | Down |  |  |
|  |  | *Me1* | Down |  |  |
|  |  | *Me3* | Down |  |  |
|  |  | *Meox2* | Down |  |  |
|  |  | *Mep1a* | Down |  |  |
|  |  | *Mep1b* | Down |  |  |
|  |  | *Metrn* | Down |  |  |
|  |  | *Mettl15* | Down |  |  |
|  |  | *Mettl21b* | Down |  |  |
|  |  | *Mettl7a1* | Down |  |  |
|  |  | *Mettl7a2* | Down |  |  |
|  |  | *Mettl7a3* | Down |  |  |
|  |  | *Mettl7b* | Down |  |  |
|  |  | *Mettl8* | Down |  |  |
|  |  | *Mfap3l* | Down |  |  |
|  |  | *Mfsd1* | Down |  |  |
|  |  | *Mfsd4a* | Down |  |  |
|  |  | *Mfsd4b1* | Down |  |  |
|  |  | *Mfsd4b5* | Down |  |  |
|  |  | *Mfsd7a* | Down |  |  |
|  |  | *Mfsd7c* | Down |  |  |
|  |  | *Mfsd9* | Down |  |  |
|  |  | *Mgam* | Down |  |  |
|  |  | *Mgat3* | Down |  |  |
|  |  | *Miox* | Down |  |  |
|  |  | *Mipep* | Down |  |  |
|  |  | *Mkks* | Down |  |  |
|  |  | *Mlycd* | Down |  |  |
|  |  | *Mmab* | Down |  |  |
|  |  | *Mme* | Down |  |  |
|  |  | *Mmp13* | Down |  |  |
|  |  | *Mmp16* | Down |  |  |
|  |  | *Mnd1* | Down |  |  |
|  |  | *Mob3b* | Down |  |  |
|  |  | *Mogat1* | Down |  |  |
|  |  | *Mogat2* | Down |  |  |
|  |  | *Morn2* | Down |  |  |
|  |  | *Morn5* | Down |  |  |
|  |  | *Mpc1* | Down |  |  |
|  |  | *Mpc2* | Down |  |  |
|  |  | *Mpp4* | Down |  |  |
|  |  | *Mppe1* | Down |  |  |
|  |  | *Mpst* | Down |  |  |
|  |  | *Mpv17l* | Down |  |  |
|  |  | *Mrap2* | Down |  |  |
|  |  | *Mrgprh* | Down |  |  |
|  |  | *Mrln* | Down |  |  |
|  |  | *Mrm2* | Down |  |  |
|  |  | *Mrpl12* | Down |  |  |
|  |  | *Mrpl50* | Down |  |  |
|  |  | *Mrps36* | Down |  |  |
|  |  | *Ms4a1* | Down |  |  |
|  |  | *Ms4a14* | Down |  |  |
|  |  | *Ms4a4b* | Down |  |  |
|  |  | *Msmp* | Down |  |  |
|  |  | *Msra* | Down |  |  |
|  |  | *Msrb2* | Down |  |  |
|  |  | *Mterf2* | Down |  |  |
|  |  | *Mtfp1* | Down |  |  |
|  |  | *Mthfd1* | Down |  |  |
|  |  | *Mtif3* | Down |  |  |
|  |  | *Mtor* | Down |  |  |
|  |  | *Mtrf1* | Down |  |  |
|  |  | *Mtss1* | Down |  |  |
|  |  | *Mttp* | Down |  |  |
|  |  | *Mturn* | Down |  |  |
|  |  | *Mtus1* | Down |  |  |
|  |  | *Muc19* | Down |  |  |
|  |  | *Mut* | Down |  |  |
|  |  | *Mvd* | Down |  |  |
|  |  | *Myh14* | Down |  |  |
|  |  | *Myh6* | Down |  |  |
|  |  | *Myh7* | Down |  |  |
|  |  | *Myl6b* | Down |  |  |
|  |  | *Mylk3* | Down |  |  |
|  |  | *Myo5a* | Down |  |  |
|  |  | *Myo6* | Down |  |  |
|  |  | *Myo7b* | Down |  |  |
|  |  | *Myom3* | Down |  |  |
|  |  | *Nadk2* | Down |  |  |
|  |  | *Nadsyn1* | Down |  |  |
|  |  | *Naglu* | Down |  |  |
|  |  | *Nalcn* | Down |  |  |
|  |  | *Nap1l5* | Down |  |  |
|  |  | *Naprt* | Down |  |  |
|  |  | *Narf* | Down |  |  |
|  |  | *Nat1* | Down |  |  |
|  |  | *Nat8* | Down |  |  |
|  |  | *Nat8f1* | Down |  |  |
|  |  | *Nat8f2* | Down |  |  |
|  |  | *Nat8f3* | Down |  |  |
|  |  | *Nat8f5* | Down |  |  |
|  |  | *Nat8f6* | Down |  |  |
|  |  | *Nat8f7* | Down |  |  |
|  |  | *Ncam2* | Down |  |  |
|  |  | *Nccrp1* | Down |  |  |
|  |  | *Ncr1* | Down |  |  |
|  |  | *Ndp* | Down |  |  |
|  |  | *Ndufa10* | Down |  |  |
|  |  | *Ndufa9* | Down |  |  |
|  |  | *Ndufb5* | Down |  |  |
|  |  | *Ndufc2* | Down |  |  |
|  |  | *Ndufs1* | Down |  |  |
|  |  | *Ndufs8* | Down |  |  |
|  |  | *Ndufv1* | Down |  |  |
|  |  | *Ndufv3* | Down |  |  |
|  |  | *Neil2* | Down |  |  |
|  |  | *Nek11* | Down |  |  |
|  |  | *Nepn* | Down |  |  |
|  |  | *Neu1* | Down |  |  |
|  |  | *Neu2* | Down |  |  |
|  |  | *Neurl4* | Down |  |  |
|  |  | *Nfs1* | Down |  |  |
|  |  | *Ngef* | Down |  |  |
|  |  | *Nim1k* | Down |  |  |
|  |  | *Nipsnap1* | Down |  |  |
|  |  | *Nit1* | Down |  |  |
|  |  | *Nit2* | Down |  |  |
|  |  | *Nkain2* | Down |  |  |
|  |  | *Nkg7* | Down |  |  |
|  |  | *Nkx3-1* | Down |  |  |
|  |  | *Nlgn1* | Down |  |  |
|  |  | *Nlrp4f* | Down |  |  |
|  |  | *Nlrp6* | Down |  |  |
|  |  | *Nme3* | Down |  |  |
|  |  | *Nme5* | Down |  |  |
|  |  | *Nme7* | Down |  |  |
|  |  | *Nmrk1* | Down |  |  |
|  |  | *Nnt* | Down |  |  |
|  |  | *Nos1* | Down |  |  |
|  |  | *Nos2* | Down |  |  |
|  |  | *Notum* | Down |  |  |
|  |  | *Nox4* | Down |  |  |
|  |  | *Nr0b2* | Down |  |  |
|  |  | *Nr1d1* | Down |  |  |
|  |  | *Nr1d2* | Down |  |  |
|  |  | *Nr1h4* | Down |  |  |
|  |  | *Nr1i3* | Down |  |  |
|  |  | *Nr2e3* | Down |  |  |
|  |  | *Nrap* | Down |  |  |
|  |  | *Nrep* | Down |  |  |
|  |  | *Nrg2* | Down |  |  |
|  |  | *Nrxn3* | Down |  |  |
|  |  | *Nsdhl* | Down |  |  |
|  |  | *Nsg2* | Down |  |  |
|  |  | *Nt5dc2* | Down |  |  |
|  |  | *Ntf3* | Down |  |  |
|  |  | *Ntng1* | Down |  |  |
|  |  | *Ntrk1* | Down |  |  |
|  |  | *Nubpl* | Down |  |  |
|  |  | *Nudt12* | Down |  |  |
|  |  | *Nudt19* | Down |  |  |
|  |  | *Nudt6* | Down |  |  |
|  |  | *Nuggc* | Down |  |  |
|  |  | *Nus1* | Down |  |  |
|  |  | *Nxf7* | Down |  |  |
|  |  | *Nxpe3* | Down |  |  |
|  |  | *Nxph4* | Down |  |  |
|  |  | *Nxt2* | Down |  |  |
|  |  | *Odc1* | Down |  |  |
|  |  | *Odf4* | Down |  |  |
|  |  | *Ogdh* | Down |  |  |
|  |  | *Ogfod3* | Down |  |  |
|  |  | *Olfm3* | Down |  |  |
|  |  | *Olfm4* | Down |  |  |
|  |  | *Olfr111* | Down |  |  |
|  |  | *Olfr1393* | Down |  |  |
|  |  | *Olfr1396* | Down |  |  |
|  |  | *Olfr195* | Down |  |  |
|  |  | *Olfr78* | Down |  |  |
|  |  | *Omd* | Down |  |  |
|  |  | *Opcml* | Down |  |  |
|  |  | *Osbp2* | Down |  |  |
|  |  | *Osbpl3* | Down |  |  |
|  |  | *Osbpl6* | Down |  |  |
|  |  | *Osbpl8* | Down |  |  |
|  |  | *Osgin1* | Down |  |  |
|  |  | *Osr1* | Down |  |  |
|  |  | *Osr2* | Down |  |  |
|  |  | *Otoa* | Down |  |  |
|  |  | *Otop3* | Down |  |  |
|  |  | *Oxct1* | Down |  |  |
|  |  | *Oxgr1* | Down |  |  |
|  |  | *Oxld1* | Down |  |  |
|  |  | *Oxsm* | Down |  |  |
|  |  | *P2rx3* | Down |  |  |
|  |  | *P2ry1* | Down |  |  |
|  |  | *P3h2* | Down |  |  |
|  |  | *Pacrg* | Down |  |  |
|  |  | *Pah* | Down |  |  |
|  |  | *Pank1* | Down |  |  |
|  |  | *Pappa2* | Down |  |  |
|  |  | *Papss1* | Down |  |  |
|  |  | *Papss2* | Down |  |  |
|  |  | *Paqr7* | Down |  |  |
|  |  | *Paqr9* | Down |  |  |
|  |  | *Park2* | Down |  |  |
|  |  | *Pax5* | Down |  |  |
|  |  | *Pbld1* | Down |  |  |
|  |  | *Pbld2* | Down |  |  |
|  |  | *Pcbd1* | Down |  |  |
|  |  | *Pcca* | Down |  |  |
|  |  | *Pccb* | Down |  |  |
|  |  | *Pck1* | Down |  |  |
|  |  | *Pclo* | Down |  |  |
|  |  | *Pcmtd2* | Down |  |  |
|  |  | *Pcsk5* | Down |  |  |
|  |  | *Pcsk6* | Down |  |  |
|  |  | *Pctp* | Down |  |  |
|  |  | *Pcx* | Down |  |  |
|  |  | *Pcyt2* | Down |  |  |
|  |  | *Pde11a* | Down |  |  |
|  |  | *Pde4c* | Down |  |  |
|  |  | *Pde6a* | Down |  |  |
|  |  | *Pde8a* | Down |  |  |
|  |  | *Pde9a* | Down |  |  |
|  |  | *Pdgfc* | Down |  |  |
|  |  | *Pdk1* | Down |  |  |
|  |  | *Pdk2* | Down |  |  |
|  |  | *Pdk3* | Down |  |  |
|  |  | *Pdp2* | Down |  |  |
|  |  | *Pdpr* | Down |  |  |
|  |  | *Pdss1* | Down |  |  |
|  |  | *Pdzd2* | Down |  |  |
|  |  | *Pdzd3* | Down |  |  |
|  |  | *Pdzd7* | Down |  |  |
|  |  | *Pdzk1* | Down |  |  |
|  |  | *Pdzk1ip1* | Down |  |  |
|  |  | *Pdzrn3* | Down |  |  |
|  |  | *Pdzrn4* | Down |  |  |
|  |  | *Pecr* | Down |  |  |
|  |  | *Peli2* | Down |  |  |
|  |  | *Pepd* | Down |  |  |
|  |  | *Per2* | Down |  |  |
|  |  | *Per3* | Down |  |  |
|  |  | *Perm1* | Down |  |  |
|  |  | *Pex1* | Down |  |  |
|  |  | *Pex11g* | Down |  |  |
|  |  | *Pex5l* | Down |  |  |
|  |  | *Pex7* | Down |  |  |
|  |  | *Pfkm* | Down |  |  |
|  |  | *Pfn4* | Down |  |  |
|  |  | *Pgam2* | Down |  |  |
|  |  | *Phb* | Down |  |  |
|  |  | *Phkb* | Down |  |  |
|  |  | *Phyh* | Down |  |  |
|  |  | *Phyhip* | Down |  |  |
|  |  | *Pi4ka* | Down |  |  |
|  |  | *Pigr* | Down |  |  |
|  |  | *Pigz* | Down |  |  |
|  |  | *Pik3c2g* | Down |  |  |
|  |  | *Pink1* | Down |  |  |
|  |  | *Pip5k1b* | Down |  |  |
|  |  | *Pipox* | Down |  |  |
|  |  | *Pkd2l2* | Down |  |  |
|  |  | *Pkhd1l1* | Down |  |  |
|  |  | *Pla2g2c* | Down |  |  |
|  |  | *Pla2g5* | Down |  |  |
|  |  | *Plcb1* | Down |  |  |
|  |  | *Pld6* | Down |  |  |
|  |  | *Plekha6* | Down |  |  |
|  |  | *Plekhd1* | Down |  |  |
|  |  | *Plg* | Down |  |  |
|  |  | *Plk5* | Down |  |  |
|  |  | *Pllp* | Down |  |  |
|  |  | *Plpp7* | Down |  |  |
|  |  | *Plscr4* | Down |  |  |
|  |  | *Pm20d1* | Down |  |  |
|  |  | *Pmpcb* | Down |  |  |
|  |  | *Pnkd* | Down |  |  |
|  |  | *Pnliprp1* | Down |  |  |
|  |  | *Pnmal2* | Down |  |  |
|  |  | *Pnpla3* | Down |  |  |
|  |  | *Pnpo* | Down |  |  |
|  |  | *Podnl1* | Down |  |  |
|  |  | *Pomgnt2* | Down |  |  |
|  |  | *Ppa2* | Down |  |  |
|  |  | *Ppargc1a* | Down |  |  |
|  |  | *Ppargc1b* | Down |  |  |
|  |  | *Ppil6* | Down |  |  |
|  |  | *Ppm1e* | Down |  |  |
|  |  | *Ppm1l* | Down |  |  |
|  |  | *Ppp1r16b* | Down |  |  |
|  |  | *Ppp1r1a* | Down |  |  |
|  |  | *Ppp1r1b* | Down |  |  |
|  |  | *Ppp1r21* | Down |  |  |
|  |  | *Ppp2r2b* | Down |  |  |
|  |  | *Ppt2* | Down |  |  |
|  |  | *Prdx5* | Down |  |  |
|  |  | *Prickle4* | Down |  |  |
|  |  | *Prima1* | Down |  |  |
|  |  | *Prkcz* | Down |  |  |
|  |  | *Prkd1* | Down |  |  |
|  |  | *Prlr* | Down |  |  |
|  |  | *Proc* | Down |  |  |
|  |  | *Prodh* | Down |  |  |
|  |  | *Prodh2* | Down |  |  |
|  |  | *Prok1* | Down |  |  |
|  |  | *Prox1* | Down |  |  |
|  |  | *Proz* | Down |  |  |
|  |  | *Prrx2* | Down |  |  |
|  |  | *Prss12* | Down |  |  |
|  |  | *Prss8* | Down |  |  |
|  |  | *Psd* | Down |  |  |
|  |  | *Pter* | Down |  |  |
|  |  | *Ptgds* | Down |  |  |
|  |  | *Ptger3* | Down |  |  |
|  |  | *Ptgr2* | Down |  |  |
|  |  | *Pth1r* | Down |  |  |
|  |  | *Ptn* | Down |  |  |
|  |  | *Ptprd* | Down |  |  |
|  |  | *Ptprn2* | Down |  |  |
|  |  | *Pvalb* | Down |  |  |
|  |  | *Pxmp2* | Down |  |  |
|  |  | *Pycrl* | Down |  |  |
|  |  | *Pyroxd2* | Down |  |  |
|  |  | *Pyurf* | Down |  |  |
|  |  | *Pzp* | Down |  |  |
|  |  | *Qdpr* | Down |  |  |
|  |  | *Qprt* | Down |  |  |
|  |  | *R3hdml* | Down |  |  |
|  |  | *Rab11fip3* | Down |  |  |
|  |  | *Rab11fip4* | Down |  |  |
|  |  | *Rab36* | Down |  |  |
|  |  | *Rab3a* | Down |  |  |
|  |  | *Rab3il1* | Down |  |  |
|  |  | *Rab3ip* | Down |  |  |
|  |  | *Rab42* | Down |  |  |
|  |  | *Rab4a* | Down |  |  |
|  |  | *Rad51c* | Down |  |  |
|  |  | *Ralgps2* | Down |  |  |
|  |  | *Ralyl* | Down |  |  |
|  |  | *Ranbp3l* | Down |  |  |
|  |  | *Rap1gap* | Down |  |  |
|  |  | *Rapsn* | Down |  |  |
|  |  | *Rars2* | Down |  |  |
|  |  | *Rasd2* | Down |  |  |
|  |  | *Rasl2-9* | Down |  |  |
|  |  | *Rbfox1* | Down |  |  |
|  |  | *Rbpms2* | Down |  |  |
|  |  | *Rd3* | Down |  |  |
|  |  | *Rdh16* | Down |  |  |
|  |  | *Rdh7* | Down |  |  |
|  |  | *Renbp* | Down |  |  |
|  |  | *Reps2* | Down |  |  |
|  |  | *Retsat* | Down |  |  |
|  |  | *Rfng* | Down |  |  |
|  |  | *Rgl1* | Down |  |  |
|  |  | *Rgs11* | Down |  |  |
|  |  | *Rhbg* | Down |  |  |
|  |  | *Rhcg* | Down |  |  |
|  |  | *Rhobtb1* | Down |  |  |
|  |  | *Rhov* | Down |  |  |
|  |  | *Rida* | Down |  |  |
|  |  | *Rilp* | Down |  |  |
|  |  | *Rmnd1* | Down |  |  |
|  |  | *Rnf152* | Down |  |  |
|  |  | *Rnf182* | Down |  |  |
|  |  | *Rnf212b* | Down |  |  |
|  |  | *Rnf24* | Down |  |  |
|  |  | *Rnf5* | Down |  |  |
|  |  | *Rnls* | Down |  |  |
|  |  | *Ropn1l* | Down |  |  |
|  |  | *Rp1* | Down |  |  |
|  |  | *Rpp40* | Down |  |  |
|  |  | *Rpusd3* | Down |  |  |
|  |  | *Rtn4ip1* | Down |  |  |
|  |  | *Rtp1* | Down |  |  |
|  |  | *Rtp3* | Down |  |  |
|  |  | *Rufy3* | Down |  |  |
|  |  | *Rundc3b* | Down |  |  |
|  |  | *Rwdd2b* | Down |  |  |
|  |  | *Rwdd3* | Down |  |  |
|  |  | *Rxrg* | Down |  |  |
|  |  | *Ryr2* | Down |  |  |
|  |  | *S100g* | Down |  |  |
|  |  | *Sall2* | Down |  |  |
|  |  | *Sall3* | Down |  |  |
|  |  | *Samd3* | Down |  |  |
|  |  | *Sap30* | Down |  |  |
|  |  | *Sardh* | Down |  |  |
|  |  | *Sat2* | Down |  |  |
|  |  | *Scfd2* | Down |  |  |
|  |  | *Scn4a* | Down |  |  |
|  |  | *Scn4b* | Down |  |  |
|  |  | *Scp2* | Down |  |  |
|  |  | *Scrn2* | Down |  |  |
|  |  | *Scrn3* | Down |  |  |
|  |  | *Scube3* | Down |  |  |
|  |  | *Sdc2* | Down |  |  |
|  |  | *Sdha* | Down |  |  |
|  |  | *Sdhb* | Down |  |  |
|  |  | *Sdhc* | Down |  |  |
|  |  | *Sec14l3* | Down |  |  |
|  |  | *Sec62* | Down |  |  |
|  |  | *Sectm1b* | Down |  |  |
|  |  | *Selenbp2* | Down |  |  |
|  |  | *Sema3b* | Down |  |  |
|  |  | *Sema4g* | Down |  |  |
|  |  | *Sema5b* | Down |  |  |
|  |  | *Sephs2* | Down |  |  |
|  |  | *Sepp1* | Down |  |  |
|  |  | *Sepsecs* | Down |  |  |
|  |  | *Serpina1d* | Down |  |  |
|  |  | *Serpinc1* | Down |  |  |
|  |  | *Serpinf2* | Down |  |  |
|  |  | *Sez6* | Down |  |  |
|  |  | *Sez6l* | Down |  |  |
|  |  | *Sfrp1* | Down |  |  |
|  |  | *Sfrp2* | Down |  |  |
|  |  | *Sfrp5* | Down |  |  |
|  |  | *Sfxn1* | Down |  |  |
|  |  | *Sfxn2* | Down |  |  |
|  |  | *Sfxn5* | Down |  |  |
|  |  | *Sgcz* | Down |  |  |
|  |  | *Sgip1* | Down |  |  |
|  |  | *Sgk2* | Down |  |  |
|  |  | *Sgpp1* | Down |  |  |
|  |  | *Sh2d1a* | Down |  |  |
|  |  | *Sh3gl2* | Down |  |  |
|  |  | *Shank2* | Down |  |  |
|  |  | *Shc3* | Down |  |  |
|  |  | *Shd* | Down |  |  |
|  |  | *Shf* | Down |  |  |
|  |  | *Shh* | Down |  |  |
|  |  | *Shmt1* | Down |  |  |
|  |  | *Shmt2* | Down |  |  |
|  |  | *Shpk* | Down |  |  |
|  |  | *Siae* | Down |  |  |
|  |  | *Sigirr* | Down |  |  |
|  |  | *Sirt3* | Down |  |  |
|  |  | *Skiv2l2* | Down |  |  |
|  |  | *Slamf7* | Down |  |  |
|  |  | *Slamf8* | Down |  |  |
|  |  | *Slc10a1* | Down |  |  |
|  |  | *Slc10a2* | Down |  |  |
|  |  | *Slc10a5* | Down |  |  |
|  |  | *Slc11a2* | Down |  |  |
|  |  | *Slc12a1* | Down |  |  |
|  |  | *Slc12a3* | Down |  |  |
|  |  | *Slc12a6* | Down |  |  |
|  |  | *Slc12a7* | Down |  |  |
|  |  | *Slc13a2* | Down |  |  |
|  |  | *Slc13a3* | Down |  |  |
|  |  | *Slc13a4* | Down |  |  |
|  |  | *Slc13a5* | Down |  |  |
|  |  | *Slc14a1* | Down |  |  |
|  |  | *Slc15a2* | Down |  |  |
|  |  | *Slc16a10* | Down |  |  |
|  |  | *Slc16a12* | Down |  |  |
|  |  | *Slc16a13* | Down |  |  |
|  |  | *Slc16a14* | Down |  |  |
|  |  | *Slc16a2* | Down |  |  |
|  |  | *Slc16a4* | Down |  |  |
|  |  | *Slc16a7* | Down |  |  |
|  |  | *Slc16a9* | Down |  |  |
|  |  | *Slc17a1* | Down |  |  |
|  |  | *Slc17a3* | Down |  |  |
|  |  | *Slc18a1* | Down |  |  |
|  |  | *Slc19a1* | Down |  |  |
|  |  | *Slc1a1* | Down |  |  |
|  |  | *Slc1a2* | Down |  |  |
|  |  | *Slc22a1* | Down |  |  |
|  |  | *Slc22a12* | Down |  |  |
|  |  | *Slc22a13* | Down |  |  |
|  |  | *Slc22a17* | Down |  |  |
|  |  | *Slc22a18* | Down |  |  |
|  |  | *Slc22a19* | Down |  |  |
|  |  | *Slc22a2* | Down |  |  |
|  |  | *Slc22a22* | Down |  |  |
|  |  | *Slc22a26* | Down |  |  |
|  |  | *Slc22a27* | Down |  |  |
|  |  | *Slc22a28* | Down |  |  |
|  |  | *Slc22a29* | Down |  |  |
|  |  | *Slc22a30* | Down |  |  |
|  |  | *Slc22a4* | Down |  |  |
|  |  | *Slc22a5* | Down |  |  |
|  |  | *Slc22a6* | Down |  |  |
|  |  | *Slc22a7* | Down |  |  |
|  |  | *Slc22a8* | Down |  |  |
|  |  | *Slc25a10* | Down |  |  |
|  |  | *Slc25a11* | Down |  |  |
|  |  | *Slc25a13* | Down |  |  |
|  |  | *Slc25a15* | Down |  |  |
|  |  | *Slc25a16* | Down |  |  |
|  |  | *Slc25a21* | Down |  |  |
|  |  | *Slc25a23* | Down |  |  |
|  |  | *Slc25a26* | Down |  |  |
|  |  | *Slc25a39* | Down |  |  |
|  |  | *Slc25a42* | Down |  |  |
|  |  | *Slc26a1* | Down |  |  |
|  |  | *Slc26a10* | Down |  |  |
|  |  | *Slc26a4* | Down |  |  |
|  |  | *Slc26a6* | Down |  |  |
|  |  | *Slc26a7* | Down |  |  |
|  |  | *Slc27a2* | Down |  |  |
|  |  | *Slc28a1* | Down |  |  |
|  |  | *Slc29a3* | Down |  |  |
|  |  | *Slc2a12* | Down |  |  |
|  |  | *Slc2a13* | Down |  |  |
|  |  | *Slc2a2* | Down |  |  |
|  |  | *Slc2a4* | Down |  |  |
|  |  | *Slc2a5* | Down |  |  |
|  |  | *Slc2a7* | Down |  |  |
|  |  | *Slc30a2* | Down |  |  |
|  |  | *Slc31a1* | Down |  |  |
|  |  | *Slc34a1* | Down |  |  |
|  |  | *Slc34a3* | Down |  |  |
|  |  | *Slc35a3* | Down |  |  |
|  |  | *Slc35f1* | Down |  |  |
|  |  | *Slc35f3* | Down |  |  |
|  |  | *Slc36a1* | Down |  |  |
|  |  | *Slc37a4* | Down |  |  |
|  |  | *Slc38a4* | Down |  |  |
|  |  | *Slc39a11* | Down |  |  |
|  |  | *Slc39a5* | Down |  |  |
|  |  | *Slc39a8* | Down |  |  |
|  |  | *Slc3a1* | Down |  |  |
|  |  | *Slc44a4* | Down |  |  |
|  |  | *Slc46a1* | Down |  |  |
|  |  | *Slc46a3* | Down |  |  |
|  |  | *Slc47a1* | Down |  |  |
|  |  | *Slc4a1* | Down |  |  |
|  |  | *Slc4a4* | Down |  |  |
|  |  | *Slc4a5* | Down |  |  |
|  |  | *Slc51a* | Down |  |  |
|  |  | *Slc51b* | Down |  |  |
|  |  | *Slc5a11* | Down |  |  |
|  |  | *Slc5a12* | Down |  |  |
|  |  | *Slc5a2* | Down |  |  |
|  |  | *Slc5a6* | Down |  |  |
|  |  | *Slc5a8* | Down |  |  |
|  |  | *Slc5a9* | Down |  |  |
|  |  | *Slc6a13* | Down |  |  |
|  |  | *Slc6a15* | Down |  |  |
|  |  | *Slc6a19* | Down |  |  |
|  |  | *Slc6a20b* | Down |  |  |
|  |  | *Slc6a4* | Down |  |  |
|  |  | *Slc6a7* | Down |  |  |
|  |  | *Slc7a12* | Down |  |  |
|  |  | *Slc7a13* | Down |  |  |
|  |  | *Slc7a7* | Down |  |  |
|  |  | *Slc7a8* | Down |  |  |
|  |  | *Slc7a9* | Down |  |  |
|  |  | *Slc8a1* | Down |  |  |
|  |  | *Slc9a3* | Down |  |  |
|  |  | *Slc9a3r1* | Down |  |  |
|  |  | *Slc9a8* | Down |  |  |
|  |  | *Slco1a1* | Down |  |  |
|  |  | *Slco1a4* | Down |  |  |
|  |  | *Slco1a6* | Down |  |  |
|  |  | *Slco1b2* | Down |  |  |
|  |  | *Slit2* | Down |  |  |
|  |  | *Slitrk1* | Down |  |  |
|  |  | *Slitrk4* | Down |  |  |
|  |  | *Slitrk6* | Down |  |  |
|  |  | *Smad9* | Down |  |  |
|  |  | *Smarca2* | Down |  |  |
|  |  | *Smco3* | Down |  |  |
|  |  | *Smcp* | Down |  |  |
|  |  | *Smim19* | Down |  |  |
|  |  | *Smim22* | Down |  |  |
|  |  | *Smim24* | Down |  |  |
|  |  | *Smim4* | Down |  |  |
|  |  | *Smim5* | Down |  |  |
|  |  | *Smim9* | Down |  |  |
|  |  | *Smlr1* | Down |  |  |
|  |  | *Smoc1* | Down |  |  |
|  |  | *Smpd2* | Down |  |  |
|  |  | *Smpdl3a* | Down |  |  |
|  |  | *Smpx* | Down |  |  |
|  |  | *Sms* | Down |  |  |
|  |  | *Smyd3* | Down |  |  |
|  |  | *Snca* | Down |  |  |
|  |  | *Snhg11* | Down |  |  |
|  |  | *Snx22* | Down |  |  |
|  |  | *Snx29* | Down |  |  |
|  |  | *Snx31* | Down |  |  |
|  |  | *Snx8* | Down |  |  |
|  |  | *Sod1* | Down |  |  |
|  |  | *Sod2* | Down |  |  |
|  |  | *Sod3* | Down |  |  |
|  |  | *Sord* | Down |  |  |
|  |  | *Sost* | Down |  |  |
|  |  | *Sostdc1* | Down |  |  |
|  |  | *Sowaha* | Down |  |  |
|  |  | *Sp5* | Down |  |  |
|  |  | *Spag8* | Down |  |  |
|  |  | *Spata17* | Down |  |  |
|  |  | *Spata22* | Down |  |  |
|  |  | *Spata2l* | Down |  |  |
|  |  | *Spats1* | Down |  |  |
|  |  | *Spef2* | Down |  |  |
|  |  | *Spns3* | Down |  |  |
|  |  | *Spock3* | Down |  |  |
|  |  | *Spp2* | Down |  |  |
|  |  | *Spsb4* | Down |  |  |
|  |  | *Sptlc3* | Down |  |  |
|  |  | *Srcin1* | Down |  |  |
|  |  | *Srd5a2* | Down |  |  |
|  |  | *Srl* | Down |  |  |
|  |  | *Srr* | Down |  |  |
|  |  | *Sspo* | Down |  |  |
|  |  | *Ssu2* | Down |  |  |
|  |  | *St8sia1* | Down |  |  |
|  |  | *Stab2* | Down |  |  |
|  |  | *Stard4* | Down |  |  |
|  |  | *Stard8* | Down |  |  |
|  |  | *Stk32b* | Down |  |  |
|  |  | *Stkld1* | Down |  |  |
|  |  | *Stpg2* | Down |  |  |
|  |  | *Stradb* | Down |  |  |
|  |  | *Stxbp4* | Down |  |  |
|  |  | *Stxbp5l* | Down |  |  |
|  |  | *Sucla2* | Down |  |  |
|  |  | *Suclg1* | Down |  |  |
|  |  | *Suclg2* | Down |  |  |
|  |  | *Sucnr1* | Down |  |  |
|  |  | *Sufu* | Down |  |  |
|  |  | *Sugct* | Down |  |  |
|  |  | *Sult1c2* | Down |  |  |
|  |  | *Sun3* | Down |  |  |
|  |  | *Suox* | Down |  |  |
|  |  | *Supt3* | Down |  |  |
|  |  | *Susd2* | Down |  |  |
|  |  | *Susd3* | Down |  |  |
|  |  | *Susd5* | Down |  |  |
|  |  | *Sv2a* | Down |  |  |
|  |  | *Svip* | Down |  |  |
|  |  | *Sycn* | Down |  |  |
|  |  | *Syn2* | Down |  |  |
|  |  | *Syn3* | Down |  |  |
|  |  | *Syne4* | Down |  |  |
|  |  | *Syngr1* | Down |  |  |
|  |  | *Synpo2l* | Down |  |  |
|  |  | *Syp* | Down |  |  |
|  |  | *Sypl2* | Down |  |  |
|  |  | *Syt1* | Down |  |  |
|  |  | *Syt15* | Down |  |  |
|  |  | *Syt3* | Down |  |  |
|  |  | *Syt7* | Down |  |  |
|  |  | *Syt9* | Down |  |  |
|  |  | *Tarsl2* | Down |  |  |
|  |  | *Tbc1d13* | Down |  |  |
|  |  | *Tbc1d16* | Down |  |  |
|  |  | *Tbc1d32* | Down |  |  |
|  |  | *Tbx10* | Down |  |  |
|  |  | *Tbx21* | Down |  |  |
|  |  | *Tbxa2r* | Down |  |  |
|  |  | *Tbxas1* | Down |  |  |
|  |  | *Tc2n* | Down |  |  |
|  |  | *Tcaim* | Down |  |  |
|  |  | *Tcea3* | Down |  |  |
|  |  | *Tcerg1l* | Down |  |  |
|  |  | *Tchhl1* | Down |  |  |
|  |  | *Tcn2* | Down |  |  |
|  |  | *Tdrd12* | Down |  |  |
|  |  | *Tecr* | Down |  |  |
|  |  | *Tef* | Down |  |  |
|  |  | *Tenm2* | Down |  |  |
|  |  | *Tenm4* | Down |  |  |
|  |  | *Tfap2b* | Down |  |  |
|  |  | *Tfb1m* | Down |  |  |
|  |  | *Tfdp2* | Down |  |  |
|  |  | *Tfec* | Down |  |  |
|  |  | *Tfr2* | Down |  |  |
|  |  | *Tgm4* | Down |  |  |
|  |  | *Tgm7* | Down |  |  |
|  |  | *Thbs4* | Down |  |  |
|  |  | *Them7* | Down |  |  |
|  |  | *Thnsl2* | Down |  |  |
|  |  | *Thrb* | Down |  |  |
|  |  | *Thsd7b* | Down |  |  |
|  |  | *Timm9* | Down |  |  |
|  |  | *Timp3* | Down |  |  |
|  |  | *Tinag* | Down |  |  |
|  |  | *Tkfc* | Down |  |  |
|  |  | *Tldc2* | Down |  |  |
|  |  | *Tle2* | Down |  |  |
|  |  | *Tln2* | Down |  |  |
|  |  | *Tlr11* | Down |  |  |
|  |  | *Tlr12* | Down |  |  |
|  |  | *Tm2d2* | Down |  |  |
|  |  | *Tm4sf4* | Down |  |  |
|  |  | *Tmbim6* | Down |  |  |
|  |  | *Tmc3* | Down |  |  |
|  |  | *Tmco3* | Down |  |  |
|  |  | *Tmem106a* | Down |  |  |
|  |  | *Tmem116* | Down |  |  |
|  |  | *Tmem117* | Down |  |  |
|  |  | *Tmem132b* | Down |  |  |
|  |  | *Tmem139* | Down |  |  |
|  |  | *Tmem143* | Down |  |  |
|  |  | *Tmem150a* | Down |  |  |
|  |  | *Tmem169* | Down |  |  |
|  |  | *Tmem174* | Down |  |  |
|  |  | *Tmem175* | Down |  |  |
|  |  | *Tmem178* | Down |  |  |
|  |  | *Tmem178b* | Down |  |  |
|  |  | *Tmem206* | Down |  |  |
|  |  | *Tmem207* | Down |  |  |
|  |  | *Tmem229a* | Down |  |  |
|  |  | *Tmem25* | Down |  |  |
|  |  | *Tmem255a* | Down |  |  |
|  |  | *Tmem255b* | Down |  |  |
|  |  | *Tmem26* | Down |  |  |
|  |  | *Tmem260* | Down |  |  |
|  |  | *Tmem27* | Down |  |  |
|  |  | *Tmem42* | Down |  |  |
|  |  | *Tmem52b* | Down |  |  |
|  |  | *Tmem53* | Down |  |  |
|  |  | *Tmem56* | Down |  |  |
|  |  | *Tmem61* | Down |  |  |
|  |  | *Tmem64* | Down |  |  |
|  |  | *Tmem72* | Down |  |  |
|  |  | *Tmem8* | Down |  |  |
|  |  | *Tmem86a* | Down |  |  |
|  |  | *Tmem8b* | Down |  |  |
|  |  | *Tmprss9* | Down |  |  |
|  |  | *Tmtc4* | Down |  |  |
|  |  | *Tnfaip8* | Down |  |  |
|  |  | *Tnfaip8l3* | Down |  |  |
|  |  | *Tnfrsf11b* | Down |  |  |
|  |  | *Tnfrsf13c* | Down |  |  |
|  |  | *Tnfsf10* | Down |  |  |
|  |  | *Tnn* | Down |  |  |
|  |  | *Tpk1* | Down |  |  |
|  |  | *Tpmt* | Down |  |  |
|  |  | *Tppp* | Down |  |  |
|  |  | *Tprkb* | Down |  |  |
|  |  | *Trabd2b* | Down |  |  |
|  |  | *Trap1* | Down |  |  |
|  |  | *Trappc9* | Down |  |  |
|  |  | *Trdn* | Down |  |  |
|  |  | *Treh* | Down |  |  |
|  |  | *Tril* | Down |  |  |
|  |  | *Trim60* | Down |  |  |
|  |  | *Trim61* | Down |  |  |
|  |  | *Trim63* | Down |  |  |
|  |  | *Trim7* | Down |  |  |
|  |  | *Trim75* | Down |  |  |
|  |  | *Triqk* | Down |  |  |
|  |  | *Trmt2b* | Down |  |  |
|  |  | *Trpc5* | Down |  |  |
|  |  | *Trpm1* | Down |  |  |
|  |  | *Trpm3* | Down |  |  |
|  |  | *Trps1* | Down |  |  |
|  |  | *Tsga10* | Down |  |  |
|  |  | *Tspan18* | Down |  |  |
|  |  | *Tspan3* | Down |  |  |
|  |  | *Tspyl4* | Down |  |  |
|  |  | *Tspyl5* | Down |  |  |
|  |  | *Tst* | Down |  |  |
|  |  | *Ttbk1* | Down |  |  |
|  |  | *Ttc30a2* | Down |  |  |
|  |  | *Ttc30b* | Down |  |  |
|  |  | *Ttc36* | Down |  |  |
|  |  | *Ttc38* | Down |  |  |
|  |  | *Ttc39c* | Down |  |  |
|  |  | *Ttc41* | Down |  |  |
|  |  | *Ttc8* | Down |  |  |
|  |  | *Ttn* | Down |  |  |
|  |  | *Ttr* | Down |  |  |
|  |  | *Ttyh1* | Down |  |  |
|  |  | *Tufm* | Down |  |  |
|  |  | *Txndc16* | Down |  |  |
|  |  | *Txnrd2* | Down |  |  |
|  |  | *Tysnd1* | Down |  |  |
|  |  | *Tyw3* | Down |  |  |
|  |  | *Ube2u* | Down |  |  |
|  |  | *Ubiad1* | Down |  |  |
|  |  | *Ubxn10* | Down |  |  |
|  |  | *Ubxn2b* | Down |  |  |
|  |  | *Ugt1a7c* | Down |  |  |
|  |  | *Ugt2a3* | Down |  |  |
|  |  | *Ugt2b34* | Down |  |  |
|  |  | *Ugt2b37* | Down |  |  |
|  |  | *Ugt2b38* | Down |  |  |
|  |  | *Ugt2b5* | Down |  |  |
|  |  | *Ugt3a1* | Down |  |  |
|  |  | *Ugt3a2* | Down |  |  |
|  |  | *Ugt8a* | Down |  |  |
|  |  | *Ulk4* | Down |  |  |
|  |  | *Umod* | Down |  |  |
|  |  | *Unc13c* | Down |  |  |
|  |  | *Unc5c* | Down |  |  |
|  |  | *Upb1* | Down |  |  |
|  |  | *Uqcr11* | Down |  |  |
|  |  | *Uqcrc1* | Down |  |  |
|  |  | *Uqcrc2* | Down |  |  |
|  |  | *Uqcrfs1* | Down |  |  |
|  |  | *Uros* | Down |  |  |
|  |  | *Ush1c* | Down |  |  |
|  |  | *Ushbp1* | Down |  |  |
|  |  | *Usp2* | Down |  |  |
|  |  | *Vdac1* | Down |  |  |
|  |  | *Vegfa* | Down |  |  |
|  |  | *Vill* | Down |  |  |
|  |  | *Vipr1* | Down |  |  |
|  |  | *Vkorc1* | Down |  |  |
|  |  | *Vldlr* | Down |  |  |
|  |  | *Vmn1r19* | Down |  |  |
|  |  | *Vmn1r20* | Down |  |  |
|  |  | *Vmn1r4* | Down |  |  |
|  |  | *Vmn2r57* | Down |  |  |
|  |  | *Vpreb1* | Down |  |  |
|  |  | *Vps8* | Down |  |  |
|  |  | *Vstm2a* | Down |  |  |
|  |  | *Vwa1* | Down |  |  |
|  |  | *Vwa2* | Down |  |  |
|  |  | *Vwa8* | Down |  |  |
|  |  | *Vwce* | Down |  |  |
|  |  | *Wdpcp* | Down |  |  |
|  |  | *Wdr17* | Down |  |  |
|  |  | *Wdr31* | Down |  |  |
|  |  | *Wdr81* | Down |  |  |
|  |  | *Wdr86* | Down |  |  |
|  |  | *Wdr93* | Down |  |  |
|  |  | *Wfdc15b* | Down |  |  |
|  |  | *Wfdc16* | Down |  |  |
|  |  | *Whrn* | Down |  |  |
|  |  | *Wnk2* | Down |  |  |
|  |  | *Wnk4* | Down |  |  |
|  |  | *Wnt11* | Down |  |  |
|  |  | *Wnt5b* | Down |  |  |
|  |  | *Wscd1* | Down |  |  |
|  |  | *Wscd2* | Down |  |  |
|  |  | *Wwox* | Down |  |  |
|  |  | *Xcl1* | Down |  |  |
|  |  | *Xcr1* | Down |  |  |
|  |  | *Xkr6* | Down |  |  |
|  |  | *Xkrx* | Down |  |  |
|  |  | *Xpnpep2* | Down |  |  |
|  |  | *Xpr1* | Down |  |  |
|  |  | *Xylb* | Down |  |  |
|  |  | *Xylt1* | Down |  |  |
|  |  | *Ybx2* | Down |  |  |
|  |  | *Zbtb20* | Down |  |  |
|  |  | *Zc2hc1c* | Down |  |  |
|  |  | *Zcchc16* | Down |  |  |
|  |  | *Zfp101* | Down |  |  |
|  |  | *Zfp354b* | Down |  |  |
|  |  | *Zfp383* | Down |  |  |
|  |  | *Zfp444* | Down |  |  |
|  |  | *Zfp454* | Down |  |  |
|  |  | *Zfp493* | Down |  |  |
|  |  | *Zfp503* | Down |  |  |
|  |  | *Zfp706* | Down |  |  |
|  |  | *Zfp768* | Down |  |  |
|  |  | *Zfp773* | Down |  |  |
|  |  | *Zfp784* | Down |  |  |
|  |  | *Zfp810* | Down |  |  |
|  |  | *Zfp831* | Down |  |  |
|  |  | *Zfp879* | Down |  |  |
|  |  | *Zfp971* | Down |  |  |
|  |  | *Zmat4* | Down |  |  |
|  |  | *Zmynd10* | Down |  |  |
|  |  | *Znrf2* | Down |  |  |
|  |  | *Zpbp* | Down |  |  |
|  |  | *Zpld1* | Down |  |  |

**Table S3.** Mass spectrometry identifying proteins co-immunoprecipitated with *circAASS*.

| **Gene names** | **Approved names** | **Unique peptides** | **Score** | **Intensity ciRProbe** | **Mol. weight [kDa]** |
| --- | --- | --- | --- | --- | --- |
| *PPARGC1A* | PPARG coactivator 1 alpha | 28 | 168.2 | 151500000 | 91.712 |
| *DOCK9* | dedicator of cytokinesis 9 | 1 | 9.3077 | 37618000 | 236.44 |
| *MYH15* | myosin heavy chain 15 | 1 | 7.0236 | 32592000 | 224.62 |
| *UBTF* | upstream binding transcription factor | 2 | 11.787 | 12523000 | 89.405 |
| *KIF20B* | kinesin family member 20B | 1 | 5.9644 | 7732000 | 210.63 |
| *WDR36* | WD repeat domain 36 | 2 | 11.486 | 7281300 | 105.32 |
| *COL1A1* | collagen type I alpha 1 chain | 1 | 6.6335 | 4696300 | 138.91 |
| *SAFB2* | scaffold attachment factor B2 | 1 | 5.8974 | 3263300 | 107.47 |
| *SYNE3* | spectrin repeat containing nuclear envelope family member 3 | 1 | 5.9372 | 3226000 | 112.22 |
| *ERAP1* | endoplasmic reticulum aminopeptidase 1 | 1 | 5.9064 | 3146400 | 107.23 |
| *TP53BP1* | tumor protein p53 binding protein 1 | 1 | 8.8773 | 0 | 213.57 |
| *EXOC4* | exocyst complex component 4 | 1 | 6.1482 | 0 | 110.5 |

**Table S4.** Clinical characteristics of healthy controls, AKI patients, and CKD patients.

| Normal group (n=10) | |
| --- | --- |
| Male, n (%) | 7 (70%) |
| Age (years) | 44.70 ± 7.82 |
| Serum creatinine (μmol/L) | 70.17 ± 10.59 |
| eGFR, ml/min/1.73 m^2^ | 105.70 ± 14.88 |
| AKI patients (n=17) | |
| Male, n (%) | 10 (59%) |
| Age at biopsy, years | 34.88 ± 11.04 |
| Serum creatinine (μmol/L) | 326.18 ± 212.57 |
| eGFR, ml/min/1.73 m^2^ | 30.41 ± 19.36 |
| Primary etiological event | N (%) |
| Septic shock | 9 (52.94%) |
| Post-cardiac surgery | 5 (29.41%) |
| Cardiogenic shock | 2 (11.76%) |
| Hemorrhagic shock | 1 (5.88%) |
| CKD patients with biopsy-proven IRI-induced kidney fibrosis (n=15) | |
| Male, n (%) | 10 (67%) |
| Age at biopsy, years | 42.20 ± 8.37 |
| Serum creatinine (μmol/L) | 162.59 ± 27.45 |
| eGFR, ml/min/1.73 m^2^ | 44.08 ± 10.13 |
| Primary etiological event | N (%) |
| Septic shock | 9 (60.00%) |
| Post-cardiac surgery | 4 (26.67%) |
| Cardiogenic shock | 1 (6.67%) |
| Hemorrhagic shock | 1 (6.67%) |

Values are expressed as mean ± SD.

**Table S5.** Primers, probes and oligos used in this study.

| Primer/Probes/oligos | Species | Sequence (5' to 3') | Amplified product (bp) |
| --- | --- | --- | --- |
| *circAASS* | Human | Forward: CGGGGATCCTGATGTGTTCC  Reverse: TGCAGCATCTTGACACCTGG | 124 |
| *circAass* | Mouse | Forward: AGCACAGCTTCCAATCGAGG  Reverse: GAGTGCCATCACTGGCTTGT | 166 |
| Linear *AASS* | Human | Forward: TTATCAGACGCGACACAGCC  Reverse: CCTTTCTCCTGGTGCCCATT | 166 |
| Pre mRNA-*AASS* | Human | Forward: AAATATATCCAGACACTCCGGG  Reverse: GGTTTCAGCCAGCCACTTAG | 197 |
| *ACTB* (divergent) | Human | Forward: CCAGGGCTTACCTGTACACTG  Reverse: CGCGGCGATATCATCATCCA | 167 |
| *ACTB* (convergent) | Human | Forward: GATTCCTATGTGGGCGACGA  Reverse: AGAAGGTGTGGTGCCAGATT | 121 |
| *ACTB* | Human | Forward: CGTCTTCCCCTCCATCGTG  Reverse: TCGATGGGGTACTTCAGGGT | 129 |
| *Actb* | Mouse | Forward: CGCCACCAGTTCGCCAT  Reverse: CTTCTGACCCATTCCCACCA | 165 |
| *Il1b* | Mouse | Forward: TGCCACCTTTTGACAGTGATG  Reverse: AAGGTCCACGGGAAAGACAC | 220 |
| *Ccl2* | Mouse | Forward: GATGCAGTTAACGCCCCACT  Reverse: TGGACCCATTCCTTCTTGGG | 176 |
| *Tnf* | Mouse | Forward: AAGAGGCACTCCCCCAAAAG  Reverse: CCACTTGGTGGTTTGTGAGTG | 216 |
| *IL1B* | Human | Forward: CAGGCTGCTCTGGGATTCTC  Reverse: CCTGGAAGGAGCACTTCATCT | 170 |
| *CCL2* | Human | Forward: TCTCGCCTCCAGCATGAAAG  Reverse: GGCATTGATTGCATCTGGCT | 103 |
| *TNF* | Human | Forward: CCCATGTTGTAGCAAACCCT  Reverse: GAGGTACAGGCCCTCTGATG | 131 |
| *PINK1* | Human | Forward: CCTGGAGTGTGAAACGCTCT  Reverse: CTCCCACCCTCACCATTCAC | 147 |
| primer pair a for *IGF2BP2* RIP assay | Mouse | Forward: AGCCAGAACTTCTCCCCAGT  Reverse: ATAGCCAGACCCAAGGACCA | 162 |
| primer pair b (intron 11, CSI11) for *IGF2BP2* RIP assay | Mouse | Forward: ACTCTTATTGAGTTCACACATCA  Reverse: ACATAAGAACTACATTAAACTCAT | 278 |
| primer pair c (intron 11) for *IGF2BP2* RIP assay | Mouse | Forward: AGTGCAACTTGAGCAGGGAA  Reverse: TGGTGCTGCTTTGACGATGA | 199 |
| primer pair d (exon 2-exon 11 divergent) for *IGF2BP2* RIP assay | Mouse | Forward: CAGCACAGCTTCCAATCGAG  Reverse: GAGTGCCATCACTGGCTTGT | 167 |
| primer pair e (intron 1) for *IGF2BP2* RIP assay | Mouse | Forward: GCAATATGCGGCTTCAAGGT  Reverse: TCCCAGTTCTCTCACTGCTG | 200 |
| primer pair f (intron 1) for *IGF2BP2* RIP assay | Mouse | Forward: CTGGAACTCACGAAGGACAGT  Reverse: TGCTGCCATGCAGTGATAAG | 146 |
| *circAASS* siRNA | Human | Sense: TGAAGAAATGTCACCAGGTAA  Antisense: TTACCTGGTGACATTTCTTCA |  |
| *circAass* siRNA | Mouse | Sense: GAAGAAATGCTACCACATTAA  Antisense: TTAATGTGGTAGCATTTCTTC |  |
| *PINK1* siRNA | Human | Sense: GGATGAAGGCAGACATCAACA  Antisense: TGTTGATGTCTGCCTTCATCC |  |
| *METTL3* siRNA | Human | Sense: GAGCTATTAAATACTACAACA  Antisense: TGTTGTAGTATTTAATAGCTC |  |
| *METTL14* siRNA | Human | Sense: GGATGAACTAGAAATGCAACA  Antisense: TGTTGCATTTCTAGTTCATCC |  |
| *IGF2BP2*  siRNA | Human | Sense: GGAACTTGTTAGAAATGTAAA  Antisense: TTTACATTTCTAACAAGTTCC |  |
| *RNF34* siRNA | Human | Sense: GGATGATGATGATGAAGAA  Antisense: TTCTTCATCATCATCATCC |  |
| *circAASS* sense probe for RNA affinity isolation | Human | 5'-Biotin-TGACACCTGGTGACATTTCTT-3' |  |
| *circAASS* antisense probe for RNA affinity isolation | Human | 5'-Biotin-AAGAAATGTCACCAGGTGTCA-3' |  |
| *circAASS* junction-site probe for FISH and ISH | Human | 5'-DIG-TGACACCTGGTGACATTTCTT-3' |  |
| *circAASS* junction-site probe for ISH | Mouse | 5'-DIG-TCAAATGTGGTAGCATTTCTT-3' |  |
| *RNU6* probe for ISH | Human | 5'-DIG-CACGAATTTGCGTGTCATCCTT-3' |  |
